# M1^hot^ tumor-associated macrophages boost tissue-resident memory T cells infiltration and survival in human lung cancer

**DOI:** 10.1136/jitc-2020-000778

**Published:** 2020-07-21

**Authors:** Eva M Garrido-Martin, Toby W P Mellows, James Clarke, Anusha-Preethi Ganesan, Oliver Wood, Angelica Cazaly, Gregory Seumois, Serena J Chee, Aiman Alzetani, Emma V King, Catherine C Hedrick, Gareth Thomas, Peter S Friedmann, Christian Hermann Ottensmeier, Pandurangan Vijayanand, Tilman Sanchez-Elsner

**Affiliations:** 1 Clinical and Experimental Sciences, University of Southampton Faculty of Medicine, Southampton, UK; 2 La Jolla Institute for Immunology, La Jolla, California, USA; 3 Cancer Sciences, University of Southampton Faculty of Medicine, Southampton, UK; 4 Southampton University Hospitals NHS Trust, Southampton, UK; 5 Inflammatory Biology, La Jolla Institute for Immunology, La Jolla, California, USA; 6 Division of Vaccine Discovery, La Jolla Institute for Immunology, La Jolla, California, USA

**Keywords:** immunity, immunity, innate, lung neoplasms, macrophages

## Abstract

**Background:**

The role of tumor-associated macrophages (TAMs) in determining the outcome between the antitumor effects of the adaptive immune system and the tumor’s anti-immunity stratagems, is controversial. Macrophages modulate their activities and phenotypes by integration of signals in the tumor microenvironment. Depending on how macrophages are activated, they may adopt so-called M1-like, antitumor or M2-like, protumor profiles. In many solid tumors, a dominance of M2-like macrophages is associated with poor outcomes but in some tumor types, strong M1-like profiles are linked to better outcomes. We aimed to investigate the interrelationship of these TAM populations to establish how they modulate the efficacy of the adaptive immune system in early lung cancer.

**Methods:**

Macrophages from matched lung (non-tumor-associated macrophages (NTAMs)) and tumor samples (TAMs) from resected lung cancers were assessed by bulk and single-cell transcriptomic analysis. Protein expression of genes characteristic of M1-like (chemokine (C-X-C motif) ligand 9) or M2-like (matrix metallopeptidase 12) functions was confirmed by confocal microscopy. Immunohistochemistry related the distribution of TAM transcriptomic signatures to density of CD8^+^ tissue-resident memory T cells (T_RM_) in tumors and survival data from an independent cohort of 393 patients with lung cancer.

**Results:**

TAMs have significantly different transcriptomic profiles from NTAMs with >1000 differentially expressed genes. TAMs displayed a strong M2-like signature with no significant variation between patients. However, single-cell RNA-sequencing supported by immuno-stained cells revealed that additionally, in 25% of patients the M2-like TAMs also co-expressed a strong/hot M1-like signature (M1^hot^). Importantly, there was a strong association between the density of M1^hot^ TAMs and T_RM_ cells in tumors that was in turn linked to better survival. Our data suggest a mechanism by which M1^hot^ TAMs may recruit T_RM_ cells via CXCL9 expression and sustain them by making available more of the essential fatty acids on which T_RM_ depend.

**Conclusions:**

We showed that in early lung cancer, expression of M1-like and M2-like gene signatures are not mutually exclusive since the same TAMs can simultaneously display both gene-expression profiles. The presence of M1^hot^ TAMs was associated with a strong T_RM_ tumor-infiltrate and better outcomes. Thus, therapeutic approaches to re-program TAMs to an M1^hot^ phenotype are likely to augment the adaptive antitumor responses.

## Introduction

Lung cancer is responsible for the largest number of cancer deaths (1.8 million, worldwide) and hence, remains a clearly unmet need with approximately 2 million new cases in 2018.^1^ The major determinant of the outcome in many solid cancers is the quality of the antitumor immune response. Thus, in lung cancer, we and others have shown that the most robust correlation of survival is with the magnitude of the intratumoral infiltration with CD8^+^ cytotoxic tissue-resident memory T cells (T_RM_).^2 3^ While infiltrating immune cells can control tumor progression, it is clear that tumors defend themselves by generating a microenvironment that impairs and attenuates the function of immune cells. There is accumulating evidence that tumor-associated macrophages (TAMs) play critical roles as coordinating intermediaries at the interface between the tumors’ anti-immune defenses and the would-be antitumor effector mechanisms of the immune system.^4 5^ Different pools of macrophages (tissue-resident interstitial macrophages and monocyte-derived macrophages) can contribute to generating TAMs.^6–8^ Macrophages are immune cells that are regarded as inherently plastic, and their activation state is driven by the integration of microenvironmental signals causing them to show different gene profiles and functionalities in response to cytokines or pathogen-recognition signals.^5 9 10^ Two major and distinct gene signatures have been associated with M1 and M2 gene profiles, initially defined by the responses of macrophages to activation by either lipopolysaccharide (LPS) or T helper (Th)1 type cytokines (interferon (IFN)-γ) or Th2 type cytokines (interleukin (IL)-4, IL-5, IL-10), respectively.^6^ These activation states represent two ends of a wide spectrum of contrasting functions where M1-like (expressing genes associated with M1 gene profiles) macrophages have pro-inflammatory, immunogenic and antitumor properties, whereas M2-like (M2 gene profiles) macrophages show anti-inflammatory, tolerogenic, angiogenic and protumor effects.^7–9 11 12^ The majority of clinical studies report that TAM infiltration in solid tumors is associated with the expression of genes associated with M2 gene profiles^10 12^; M2-like TAMs help angiogenesis and activate immune suppression.^4–7^ However, a few immunohistochemical studies propose that macrophage infiltration may be favorable for patients with prostate cancer,^13^ colorectal cancer^14^ and non-small cell lung cancer (NSCLC),^15^ although the mechanisms underlying this association are not known.

There is evidence that various stimuli including drugs/agonists can cause macrophages to alter their phenotype and hence to be re-programmed from, for example, an M2 orientation towards an M1 bias.^4 5 10 11^ It remains to be established whether the two phenotypic states are mutually exclusive at the individual cell level, or whether individual macrophages can be bifunctional. Unfortunately, the analysis of TAM phenotype and function has been severely hampered by dependence on a limited range of markers, by analyses in murine models or surrogate in vitro culture systems.

Here, we analyzed the transcriptome of human TAMs isolated from patients with NSCLC and compared it with that of macrophages from paired, adjacent lung tissue from the same patients. We evaluated the TAM transcriptome for enrichment of gene signatures that are linked to tumor progression (ie, angiogenic, immunosuppressive, prometastatic and fibrotic pathways), or conversely, whether there was enrichment of signatures linked to tumor clearance by promoting cytokine release, phagocytosis or indirectly, through regulation of the adaptive immune response. Finally, we assessed whether these signatures are associated with immunological and clinical outcomes such as T-cell tumor infiltration and, most importantly, patient survival. We find, first, that TAMs display a completely different transcriptional signature from that of tissue-resident macrophages in adjacent, non-malignant lung tissue. This suggests that TAMs are modified by the tumor microenvironment. Second, the M2 and M1 signatures are not mutually exclusive even in a single cell: while in all patients the TAMs exhibit an M2 signature, in some patients TAMs are plastic and simultaneously exhibit a strong (hot) M1 signature. The presence of these M1^hot^ TAMS is associated with a strong CD8^+^ T_RM_ tumor-infiltrate and better survival outcomes, suggesting that this feature is of high clinical relevance.

## Methods

### Study design and cohort characteristics

A total of 41 patients with early stage (I–III) NSCLC from subtypes adenocarcinoma (LUAD) and squamous cell carcinoma (LUSC) consented to tissue collection. A portion of their resected tumor and tumor-free lung tissue (same lobe for paired analysis) was collected. None of the patients received neoadjuvant chemoradiotherapy. Of note, from the 41 patients we isolated TAMs from 40, and collected tissue for non-tumor-associated macrophages (NTAMs) from matched lung from 34 (one of the patients did not yield enough NTAMs, so paired analysis was performed with n=33). Sample size based on previous studies,^2 16^ and demographic characteristics are presented in online supplementary table S1.

10.1136/jitc-2020-000778.supp1 Supplementary data

### Tissue disaggregation and macrophage isolation for purified population analysis

NSCLC tumor specimens and specimens from matched normal lung from the same patients were collected after general anesthesia and surgical resection. A representative portion of each specimen was immediately formalin-fixed and used for paraffin-embedding and generation of tissue microarrays (TMA), for determination of tumor infiltrating lymphocytes (TILs) score by CD8^+^ staining, as described in the Immunohistochemistry and TIL score determination. The rest was immediately processed for tissue disaggregation and macrophage isolation as follows. Briefly, samples were collected in cold RPMI (supplemented with penicillin/streptomycin, L-glutamine and Na^+^-pyruvate) and processed immediately after resection. Tissue was minced with a scalpel and digested enzymatically with 0.15 WU/mL of D-Liberase (Roche) and 800 U/mL of DNase I (Sigma-Aldrich) for 15 min at 37°C. Then it was disaggregated into a single-cell suspension by passing it through a 70 µm strainer and rinsing with cold buffer (1× phosphate-buffered saline (PBS), 2 mM EDTA, 0.5% bovine serum albumin (BSA)). Red blood cells were lysed using an ammonium chloride-based lysing reagent (BD Pharm Lyse solution, BD Pharmingen) for 15 min on ice. Cells were counted for viability using Trypan Blue (Sigma-Aldrich). Around 5 million cells, when available, were stained for Fluorescent Activated Cell Sorting (FACS) as follows: FcR non-specific binding was blocked using FcR block (Miltenyi) for 30 min on ice. Then a cocktail of prelabeled antibodies was added: glycophorin-A (Pacific Blue, BioLegend #349108, dilution 1/50), CD45 (fluorescein isothiocyanate, BioLegend #304038, dilution 1/66), CD14 (APC-H7, BD Biosciences #641394, MφP9, dilution 1/20), HLA-DR (Allophycocyanin-APC, BD Biosciences #347403, L243, dilution 1/80). Cells with antibodies were incubated on ice for 20 min. For FACS sorting, cells were stained with DAPI (Sigma) for live/dead gating. Gating strategy used was as follows: singlets ->glycophorin-A^–^ (to remove possible residual red blood cells)/DAPI^-^ (live cells) ->CD45^+^ ->HLA-DR^+^/CD14^+^ (online supplementary figure S1B). Sorting was performed using a BD FACS Aria II Cell Sorter (BD Biosciences). Purified macrophage populations were then collected in Trizol-LS (Sigma-Aldrich) and stored at −80°C until RNA extraction was performed.

### Purified population RNA extraction and quantification

Isolated macrophages in the range of 10,000–50,000 were subjected to total RNA extraction using miRNeasy RNA extraction micro kit (Qiagen) following manufacturer’s instructions. Absolute quantification using real-time quantitative PCR (qPCR) method was used to measure RNA concentration. A standard curve was created with serial dilutions of RNA from a known concentration obtained from monocytes isolated from fresh human blood by Ficoll gradients using CD14^+^-isolating MACS columns. Quantification was performed by beta-2-microglobulin presence using SyBR-Green primers (Sigma). RNA quality was assessed in the bioanalyzer using RNA 6000 pico chips (Agilent) and RNAs with a RIN quality value ≥8.50 were used for the pre-amplification and library preparation procedures.

### RNA pre-amplification

ERCC spike-in mix (Ambion, Life Technologies) was added to 15 ng of starting RNA material. Ribosomal RNA was eliminated from total RNA by poly-A RNA positive selection using Poly(A)Purist MAG kit (Ambion, Life Technologies) following manufacturer’s instructions. Poly-A RNA was then pre-amplified using SeqPlex RNA Amplification kit for Whole Transcriptome Amplification (WTA, Sigma-Aldrich). Briefly, annealing mix was added to the poly-A RNA that was then denatured at 70°C for 5 min. Then the library synthesis buffer and RT enzyme were added, and RT was performed. The cycles of optimum amplification were then optimized using a small aliquot of the sample and amplification-mix and enzyme in a real-time qPCR using ROX dye and GelGreen. The optimal number of amplification cycles were achieved by proceeding two cycles into the amplification plateau. The rest of the sample was then amplified during the optimized number of cycles, in a mix of amplification mix and DNA amplification enzyme (WTA). Amplified product was cleaned up with ZR96 DNA clean up kit (Zymo Research) following manufacturer’s instructions. Final DNA was measured with nanodrop, double stranded DNA (dsDNA) was measured with dsDNA Quantifluor (Promega). Ratio of dsDNA/DNA was >75%. Postadaptor removal by enzymatic digestion was performed to 1 µg of DNA during 60 min at 37°C following by enzyme deactivation. Size selection and clean up was performed using Agencourt AMPure XP beads (Beckman Coulter). Beads were added to the samples in a ratio 1:1 (beads:sample) followed by three washes with 80% ethanol and eluted in Tris-EDTA buffer (TE). Final amplified DNA samples were measured by Nanodrop and size was evaluated in the bioanalyzer using High Sensitivity DNA chips (Agilent). More than 75% of amplified samples had a size of 150–450 bp. Quantification of dsDNA by Nanodrop (total DNA) and dsDNA Quantifluor to assure that >85% of the DNA amplified is dsDNA. Aliquots were taken in every step of the process and different quality checks were performed.

### Library preparation for RNA-sequencing

Amplified dsDNA was subjected to library preparation using TruSeq Nano DNA library prep kit (Illumina). Samples were subjected to end-repair during 30 min at 30°C. Size selection was performed using Agencourt AMPure XP beads in two steps: removal of large DNA fragments (>450 bp) with a ratio 1.6:1 of diluted beads:sample; removal of small DNA fragments (<150 bp) with a ratio 1:3.1 of undiluted beads:sample. DNA of interest was recovered in TE; 3’ ends of 150–450 bp DNA fragments were adenylated using A-Tailing Mix during 30 min at 37°C followed by enzyme deactivation. Then, Illumina adapters were ligated using Ligation Mix 2, Resuspension Buffer and the appropriate barcoded adapter in each case. As a last step, two sets of clean ups were performed using AMPure XP Beads: first in a 1.2:1 ratio and next in a 1.1:1 ratio. DNA was eluted with TE and PCR amplified using PCR Primer Cocktail and Enhanced PCR Mix as follows: 3 min at 95°C, and 8 cycles of 20 s at 98°C, 15 s at 60°C and 30 s at 70°C. As a final step, the final amplified libraries were cleaned up using Sample Purification Beads. The size of the library was determined by HS-DNA Agilent Bioanalyzer (150–450 bp). The amount of library was determined by Nanodrop and dsDNA Quantifluor. Quality checks were performed comparing with aliquots taken from the >450 bp and <150 bp fractions.

### RNA-sequencing and data analysis

The single-end reads (50 bp length) generated by HiSeq2500 that passed Illumina filters were filtered for those aligning to tRNA, rRNA, adapter sequences and spike-in controls. The reads were then aligned to UCSC human genome (hg19) using TopHat (V.1.4.1).^17^ DUST scores were calculated with PRINSEQ Lite (V.0.20.3)^18^ and low-complexity reads (DUST >4) were removed from the BAM files. The alignment results were parsed via the SAMtools^19^ to generate SAM files. Read counts to each genomic feature were obtained with the HTSeq-count program (V.0.6.0)^20^ using the ‘union’ option. After removing absent features (zero counts in all samples), the raw counts were then imported to R/Bioconductor package DESeq2^21^ to identify differentially expressed genes (DEGs) among samples. DESeq2 normalizes counts by dividing each column of the count table (samples) by the size factor of this column. The size factor is calculated by dividing the samples by geometric means of the genes. This brings the count values to a common scale suitable for comparison. P values for differential expression are calculated using negative binomial test for differences between the base means of two conditions. These p values are then adjusted for multiple test correction using the Benjamini-Hochberg algorithm to control the false discovery rate (V.1.14.1). We considered genes differentially expressed between two groups of samples when the DESeq2 analysis resulted in an adjusted p value (FDR) ≤0.05 and the fold-change (FC) in gene expression was ≥2 or ≤1/2 (log_2_FC≥|1|), and only genes filtered by mean expression ≥10 normalized counts across the samples were considered. Cluster analyses including principal component analysis (PCA) and hierarchical clustering were performed using standard algorithms and metrics. The t-SNE was generated using the top 3000 hypervariable genes, as calculated in DESeq2 (V.1.16.1); this allowed for unbiased visualization of the DESeq2 normalized count data, using package Rtsne (V.0.13). In order to apply log_2_ base transformation, a pseudocount (+1) was added to all the countable before log transformation. This is a usual procedure so genes with value 0 return 0 after log. Adding one does not bias the initial non-zero counts since we are expressing RNA-sequencing (RNA-seq) data as proportions. Weighted correlation analysis was completed using WGCNA (V.1.61)^22^ from the Log_2_DESeq2 normalized count data matrix and the function TOMsimilarityfromExpr (beta=5) and exportNetworkToCytoscape, weighted=true, threshold=0.05. Networks were generated in Gephi (0.92)^23^ using Fruchterman Reingold and Noverlap functions. The size was scaled according to the average degree as calculated in Gephi. The color was then curated given a significant (defined by WGCNA) correlation with *STAT1*. Purified population analysis of bulk CD8^+^ cells was previously reported^2^ and converted to transcripts per million (TPM).^16^ Data were completed as above using the TPM, the TPM values are provided in online supplementary table S5. Cell cycle and cytotoxicity gene signatures were taken from murine signatures (cluster I and cluster III),^24^ and our human lung cancer studies.^3^ Murine transcripts were converted to a direct homolog if the gene symbol was identical, otherwise genes were discarded. Signatures are provided in online supplementary table S5. Differential expression was completed as above, correcting for a batch using DESeq2. Significant genes were selected based on adjusted p value (FDR) ≤0.05 and the FC in gene expression was ≥2 or ≤1/2 (log_2_FC ≥ |1|).

### RNA-seq data public availability

RNA-seq data have been deposited on Gene Expression Omnibus (https://www.ncbi.nlm.nih.gov/geo/) under accession number GSE116948.

### Molecular pathway analysis and gene set enrichment analysis

Analysis of molecular pathways affected by DEGs was performed using Ingenuity Pathway Analysis tool (IPA, Qiagen). Genes filtered by basemean ≥10, FDR ≤0.05 and log_2_FC ≥ |1| were loaded into IPA software. A total of 1038 DEGs were loaded in the TAMs/NTAMs comparison and 222 DEGs were loaded in the chemokine (C-X-C motif) ligand 9 (*CXCL9*) high versus *CXCL9* low TAMs comparison. No extra filters were applied. Canonical pathways and upstream regulators were analyzed. Upstream regulators were ordered only among cytokines and growth factors by lowest p value (Fisher’s exact t-test). Canonical pathways were ordered by lowest p value (Fisher’s exact t-test). Gene set enrichment analysis was completed as previously described,^16^ using Qlucore (V.3.2), using the SNR setting to compare between two biological groups.

### Confocal imaging

For the M1/M2 staining with CXCL9 and matrix metallopeptidase 12 (MMP12) in TAMs, disaggregated cell suspensions from tumor and normal lung specimens were plated in several wells of μ-slide 8-well tissue culture-treated sterile chambers (#IB-80826, Ibidi). Cells were incubated in free-serum RPMI medium at 37°C for 3 hours to allow the macrophages to attach. After washing, they were fixed and permeabilized with a solution of 0.1% Triton X-100, 4% paraformaldehyde (ethanol-free) in PBS for 10 min at 4°C. Cells were stained with CD68 (mouse monoclonal antihuman CD68, clone PG-M1, #M0876, Dako; dilution 1/100), MMP12 (rabbit polyclonal antihuman MMP12 #NBP1-31225, Novus Biologicals; diluted 1/100) and CXCL9 (goat polyclonal antihuman CXCL9 #AF392, Novus Biologicals; diluted 1/20) overnight at 4°C and after washing they were incubated with secondary (donkey antirabbit Alexa405, donkey antimouse Alexa488, donkey antigoat Alexa647; all at 1/250) for 1 hour at room temperature in darkness. Washed preparations were briefly incubated with Sytox Orange for nuclear staining in tris-buffered saline and preserved in glycerol:antifade PBS (8:2) until visualization.

For the CXCR3 and CXCL9 staining in T cells, disaggregated cell suspensions from tumor and normal lung specimens were mildly centrifuged in slides using a cytospin and fixed and permeabilized as above. Staining was performed with CXCR3 (mouse monoclonal antihuman CD183/CXCR3, #557183, clone 1C6/CXCR3, BD Biosciences, dilution 1/500) and CXCL9 (goat polyclonal antihuman CXCL9 #AF392, Novus Biologicals; diluted 1/20) followed by secondary (donkey antimouse Alexa-Fluor 488, donkey antigoat Alexa-Fluor 647; all at 1/250) and nuclear staining with DAPI. Washed preparations were then mounted in Mowiol and left dry overnight at room temperature in darkness. Confocal images were taken using a SP8 Leica Confocal Microscopy.

### Immunohistochemistry and TIL score determination

Density of TILs was assessed by immunohistochemistry (IHC) using formalin-fixed paraffin-embedded TMAs. Triplicate 1 mm areas representative from each tumor were selected by pathologist review and processed into TMAs using the Aphelys Minicore 2 system (Mitogen, UK). TMAs were performed for tumors from both the cohort of patients of NSCLC adenocarcinoma and squamous cell lung carcinomas source of the TAM’s RNA-seq data in the manuscript (n=40) and for the archive-cohort of patients of NSCLC adenocarcinoma (2007–2011, n=460, data available for n=393), both from Southampton University Hospital. Serial 4 µm TMA sections were stained for the immunological markers CD8 (clone C8/144B, # IR623, Dako, Denmark) and CD14 (clone EPR6353, #114-R15 Merck, USA), the chemokine CXCL9 (clone 49106, #MAB392, R&D systems, Bio-Techne), and associated ligand CXCR3 (clone 49801, #MAB160, R&D systems, Bio-Techne). Antibody detection and visualization was performed with either high pH (CD8), or low pH (CXCL9, CXCR3) heat induced epitope retrieval using EnVision FLEX+ system and DAB as the chromogenic substrate. Optimal antibody conditions were determined in a diagnostic IHC laboratory using automated Dako Link 48 platforms and standardized protocols. For CD14 IHC, the standard protocol for OptiView DAB IHC Detection Kit by Ventana Medical Systems (USA) was used. For the RNA-seq cohort (n=40), TILs (CD8^+^), CXCL9^+^ and CXCR3^+^ cells were quantified using an Olympus DotSlide, as positive cells per field using the average of nine high-power (×400) fields across representative areas of each tumor, to allow for intratumoral heterogeneity. For the larger adenocarcinoma archive cohort TILs score was assessed, instead of counting cells, as TIL^high^ (CD8^+^ was diffuse, present in >80% of tumor/stroma); TIL^intermediate^ (CD8^+^ patchy, present in 20%–80% of tumor/stroma) or TIL^low^ (CD8^+^ weak/absent, present in <20% of tumor/stroma). CXCL9^+^ cells were assessed using the same criteria. Pictures were taken on a Zeiss AxioCam MRc5 microscope (Zeiss, Cambridge, UK) and Zeiss Axiovision software (V.4.8.1.0; Zeiss).

### Survival analysis

Two independent lung adenocarcinoma patient cohorts consisting of n=460 patients with available data for n=393 (2007–2011 archive-cohort from Southampton University Hospital) and n=511 patients with available survival data for n=495 (The Cancer Genome Atlas (TCGA)-LUAD) patients were analyzed retrospectively for survival stratifying them by their CXCL9 expression. Expression of CXCL9 in Southampton archive cohort was assessed at the protein level, by IHC and scored, as explained above. Expression of *CXCL9* or *CXCR3* for the TCGA-LUAD cohort is referred to their RNA-seq data from whole tumor available from TCGA. In both cases, the patients in the top 10% percentile and bottom 10% percentile of expression (of *CXCL9* or *CXCR3*) were categorized as high and low, respectively. Percentile of 10% was selected after visualization of the data distribution in a plateau where the top 10% of the data points are outside the plateau, as well as the bottom 10% of the data points.

### The Cancer Genome Atlas consortium data

Validation of findings was assessed in the dataset of lung adenocarcinoma (LUAD, RNA-seq data n=511) from TCGA consortium. The RNA-seq methodology and processing have been described by TCGA^25 26^; mRNA data for *CXCR3* and *CXCL9* expression were plotted and statistical significance was evaluated by Spearman’s correlation analysis.

### Single-cell transcriptomic analysis

For single-cell transcriptomics, tumor and lung samples were first dispersed (as above) and cryopreserved in freezing media (50% complete RMPI (Fisher Scientific), 40% human decomplemented AB serum, 10% dimethyl sulfoxide (both Sigma)) for two patients (online supplementary table S1). Cryopreserved samples were thawed, washed and prepared for staining. Cells were first incubated at 4°C with FcR block (Miltenyi Biotec) for 10 min, prior to staining with a combination of anti-CD45-AlexaFluor700 (HI30; BioLegend, 1/20), anti-CD14-BV421 (M5E2, BioLegend, 1/20), anti-HLA-DR-BB515 (G46-6, BD Bioscience, 1/20), anti-CD3-APC-Cy7 (SK7; BioLegend, 1/20), anti-CD56-Pe-Cy7 (HCD56; BioLegend, 1/20), CD19/20-PE/Dazzle (HIB19/2H7; both 1/40, BioLegend), for 30 min at 4°C. Live/dead discrimination was completed with potassium iodide immediately prior to acquisition. Samples (online supplementary table S1) were sorted as described in online supplementary figure S3A into low retention tubes (Thermo Fisher Scientific #3434) containing 500 µL suspension containing 50% MACS buffer (PBS containing 2% fetal bovine serum (FBS) 2 mM EDTA), 50% FBS and 200 U of Recombinant RNase Inhibitor (Takara) using a 100 micron nozzle on a FACS Aria-II (BD Biosciences). Samples were then gently vortexed and maintained at 4°C. Samples were processed using 10× v2 chemistry as per manufacturer’s recommendations.^16^ Barcoded RNA was collected and processed following manufacturer’s recommendations, as described previously. Libraries were sequenced on a HiSeq2500 (Illumina) to obtain 100 and 32 bp paired-end reads using the following read length: read 1, 26 cycles; read 2, 98 cycles and i7 index, 8 cycles (online supplementary table S1). Raw 10× data (online supplementary table S1) was processed as previously described,^16^ merging multiple cell types with cellranger aggr (V.2.0.2). The merged data were transferred to the R statistical environment for analysis using the package Seurat^16 27^ (V.2.3.0). Only cells expressing >200 genes and genes expressed in at least 3 cells were included in the analysis. The data were then log-normalized and scaled per cell and variable genes were detected. Transcriptomic data from each cell was then further normalized by the number of UMI-detected and mitochondrial genes. A PCA was then run on variable genes, and the first six principal components (PCs) were selected for further analyses based on the SD of PCs, as determined by an ‘elbow plot’ in Seurat. Outlier cells representing contaminating cells were removed and the analysis recalculated. Cells were clustered and visualized using the FindClusters function in Seurat with default settings, resolution=0.6 and 6 PCs. Differential expression between clusters was determined by converting the data to counts per million and analyzing cluster-specific differences using MAST (q<0.05, V.1.2.1).^16 28 29^ A gene was considered significantly different, only if the gene was commonly positively enriched in every comparison for a singular cluster.^16 30^ Further visualizations of exported normalized data were generated using the Seurat package and custom R scripts. Average expression across a cell cluster was calculated using the *AverageExpression* function, and downsampling was achieved using the *SubsetData* function (both in Seurat). Average expression data were clustered using average linkage and heatmaps were visualized in Qlucore as above.

### Co-culture competition lipid uptake experiments

Monocytes and CD3^+^ T lymphocytes were extracted from peripheral blood mononuclear cell (PBMC) cones obtained from the blood bank, by sequential positive selection using CD14^+^ MACS beads (130-050-201) followed by CD3^+^ MACS selection (130-050-101) on the CD14^-^ fraction. Monocytes were resuspended in RPMI containing 10% FBS at 1 million per mL and cultured for 12 days in granulocyte-macrophage colony-stimulating factor (GM-CSF) at 50 U/mL. CD3^+^ isolated cells were frozen and stored at −80°C until the day before use. Macrophage activation and polarization was completed using either IFN-γ and LPS (20 and 20 ng/mL) or IL-4 (20 ng/mL) on days 13 and 15 and uptake experiments completed on day 16 (equating to 72 hours postinitial activation). CD3^+^ cells were defrosted and rested in RPMI containing 10% FBS for 24 hours before use.

### Competition experiments

Bodipy-FL C16 (ThermoFisher #D3821) was used in conjunction with flow cytometry to assess competitive uptake. Macrophage culture media was removed to limit cytokine carry over. CD3^+^ lymphocytes and differentiated macrophages were combined in a 1:1 ratio in fresh RPMI containing 10% FBS. Cells were treated at a final concentration of 500 nM Bodipy FL C16 (reconstituted in FA Free BSA) for 30 min at 37°C. Uptake was quenched using ice cold 10 μM fatty acid free BSA. Supernatants were transferred to 4 mL FACS tubes and adherent cells detached by trypsinization and gentle scraping. Cells were spun down and washed once in PBS.

### Staining and flow cytometry for lipid uptake analysis

Live/Dead staining was completed using zombie violet viability kit (#423113) and cells labeled for CD3 (#344827), CD8 (#344710), CD103 (#350216), HLA-DR (#307616) (all purchased from BioLegend) and CD14 (BD Biosciences: #557831) before proceeding to flow cytometry. T cells with T_RM_ markers were gated as CD14^-^/HLA-DR^-^, CD3^+^/CD8^+^/CD103^+^ as outlined in online supplementary figure S6.

### Small interfering RNA knockdown of FABP3,4, 5 in lipid uptake experiments

Monocytes extracted from blood were differentiated into macrophages using GM-CSF over 5 days; 100 nM of either Scramble (Qiagen 1027287) or 33 nM each of FABP3, FABP4, FABP5 (Qiagen 1027415) were transfected using Hyperfect (Qiagen). Bodipy treatments were completed 24 hours post-transfection using 10 μM Bodipy FL C16 (ThermoFisher; D3821) for 30 min. Uptake was quenched using ice cold PBS containing fatty acids free BSA and washes were completed using PBS. Cells were detached and Bodipy uptake assessed by flow cytometry using zombie violet for the exclusion of dead cells. Mean fluorescence intensities under each treatment were compared with small interfering RNA (siRNA) scramble control to assess the percentage knockdown.

### Statistical analysis

Comparison between two groups were assessed with a Mann-Whitney U test for non-parametric samples. Multigroup comparisons were assessed by one-way analysis of variance with Kruskal-Wallis test. Wilcoxon signed-rank two-tailed test was used for paired non-parametric analysis. Kaplan-Meier survival curves were tested statistically using the Mantel-Cox log-rank test for two groups comparison or log-rank test for trend when more than two groups were compared. GraphPad PRISM 7 was used for all statistical analysis. For RNA-seq analysis, p values for differential expression are calculated using the negative binomial test for differences between the base means of two conditions, and adjusted for multiple test correction using the Benjamini-Hochberg algorithm to control the false discovery rate using DESeq2. Visualizations including PCA, gene set enrichment analysis (GSEA), t-distributed Stochastic Neighbor Embedding (t-SNE), hierarchical clustering and heatmaps were performed using R V.3.3.2 and Qlucore (V.3.2). Flow cytometry data were calculated using Flowjo (V.10.4).

## Results

### TAMs were enriched for both M2 and M1 features

**Figure 1:**
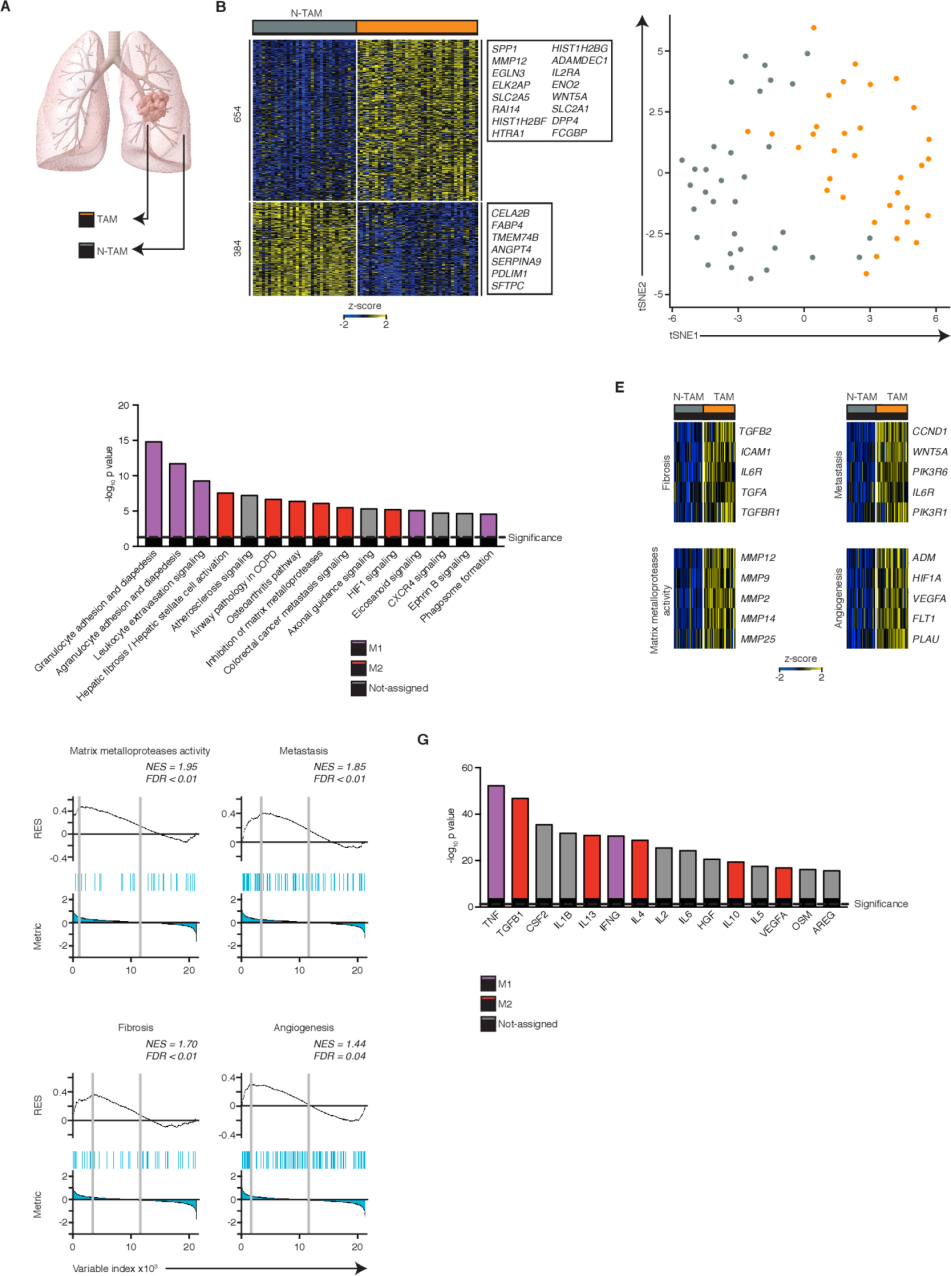
Tumor-associated macrophages (TAMs) are enriched for both M2 and M1 features. (A) Scheme of the study. (B) Heatmap showing normalized expression of 1038 differentially expressed genes (DEG) (FDR ≤0.05, log_2_fold-change (FC) ≥ |1|, basemean ≥10, n=74). (C) t-SNE analysis shows clustering of TAMs and paired non-tumor-associated macrophages (NTAMs), selecting for the 3000 hypervariable genes. (D) Top dysregulated canonical pathways based on the expression of 1038 DEGs. Statistical values displayed as –log_10_(p value). Purple columns indicate pathways related to M1 functions and red columns pathways related to M2 functions. Statistical significance baseline (dotted line) corresponds to a p value of 0.05. (E) Heatmaps showing expression of selected genes from M2 typical antitumor functions in TAMs versus NTAMs. (F) gene set enrichment analysis (GSEA) of various gene sets in the transcriptome of TAMs versus that of all NTAMs, presented as the running enrichment score for the gene set list of genes ranked to degree of over-representation. P values, Kolmogorov-Smirnov test. (G) Predicted upstream regulators (cytokines and growth factors) related to gene expression changes observed in (B). Purple columns indicate upstream regulators related to M1 functions and red columns to M2 functions. Statistical significance baseline (dotted line) corresponds to a p value of 0.05. Data are from n=41 donors (n=40 TAMs, n=34 NTAMs; n=33 paired, n=66 samples in total). Related to online supplementary figure S1 and tables S1, S2.

**Figure 3:**
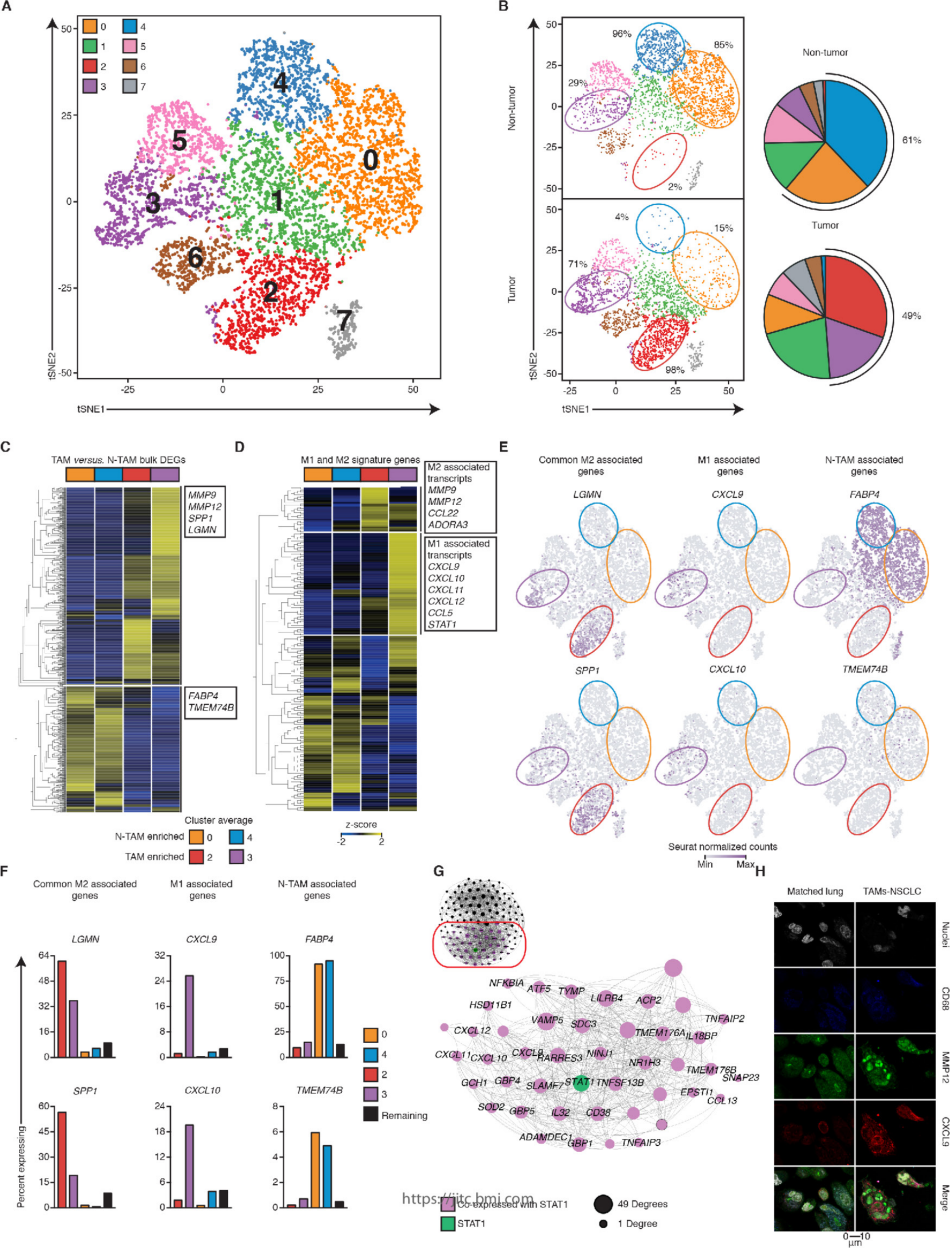
Single-cell RNA-sequencing (RNA-seq) reveals a tumor-associatedmacrophages (TAM) subset with dual M1 and M2 features. (A) t-SNE visualization of ~9000 CD19^-^CD20^-^CD56^-^CD3^-^CD45^+^ single cell transcriptomes obtained from two tumors and two matched lung samples. Each symbol represents a cell; color indicates Seurat clustering of cells identifying eight clusters. (B) Left, distribution of clusters in tumor and lung. Right, pie chart representing the relative proportions of cells in each tissue-resident memory T cells (T_RM_) cluster. (C) Single-cell RNA-seq analysis of genes differentially expressed between purified populations of TAM and non-tumor-associatedmacrophages (NTAM) (figure 1B) overlaid over the average expression of TAM-enriched and NTAM-enriched clusters. Horizontal breaks separate genes commonly upregulated or downregulated. Rows are clustered with average linkage. (D) Single-cell RNA-seq analysis of M1 and M2 associated signatures (figure 2A, B) as per (figure 3C). (E) Seurat normalized expression of indicated transcripts (cluster colored as per figure 3A), overlaid across the t-SNE plot, with expression levels represented by the color scale. (F) Percentage of cells expressing a given transcript in highlighted cluster, the median of the values from the other clusters (1, 5, 6, excluding 7) is shown. (G) WGCNA of the cluster 3 specific transcripts in 40 bulk tumor transcriptomes, visualized in Gephi the nodes are sized according to the number of edges (connections). (H) Confocal images of a representative TAM versus NTAM sample of a patient with non-small-cell lung cancer (NSCLC), showing co-expression of chemokine (C-X-C motif) ligand 9 (CXCL9) with matrix metallopeptidase 12 (MMP12). Scale bar refers to 10 µm. Related to online supplementary figure S3 and tables S1, S4.

We compared the genome-wide transcriptional profile of CD45^+^CD14^+^HLA-DR^+^ cells that includes predominantly macrophages (confirmed by single cell sequencing in figure 3) and to a lesser extent, monocytes and neutrophils as well as a recently discovered small population of mature cDC2 dendritic cells.^31^ We will refer to these cells as TAMs (we acknowledge that we may be excluding rare populations of macrophages that do not express any CD14), isolated from lung tumor and NTAMs from adjacent non-involved lung tissue samples (NTAMs) obtained from patients (paired analysis, n=33, online supplementary table S1) with treatment-naïve NSCLC (figure 1A and online supplementary figure S1A, B). This paired comparison is important to identify the molecular features specific to TAMs as they share with NTAMs the same genetic background, residency in the same end organ as well as any other potentially confounding factors (ie, gender, age, smoking, comorbidities, etc), which might otherwise bias our evaluation.

**Figure 2:**
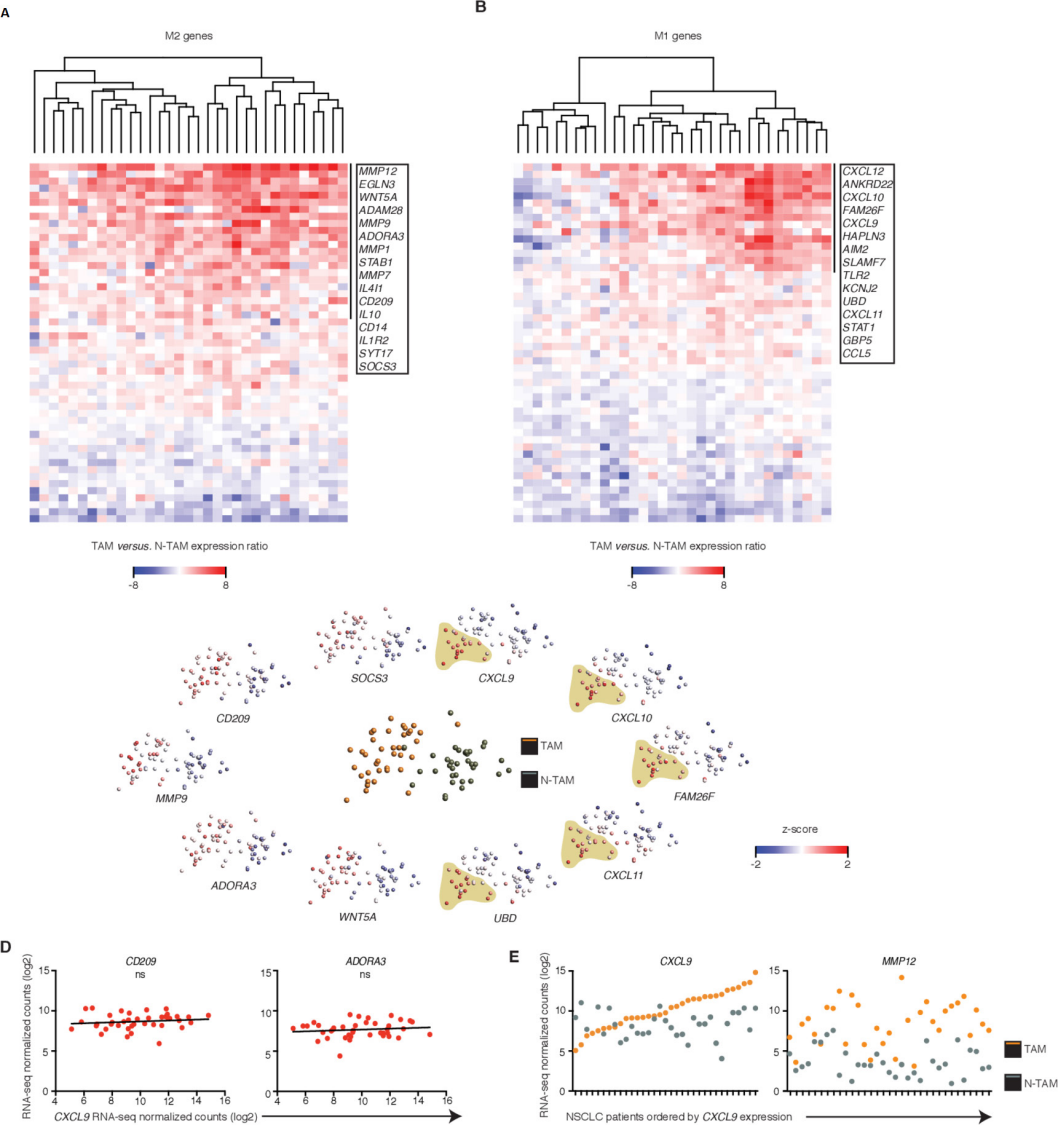
Uniform M2 but heterogeneous M1 features among patients. (A, B) Heatmaps showing changes in expression of M2 (A, 51 differentially expressed genes (DEGs)) or M1 (B, 50 DEGs) genes in tumor-associated macrophages (TAMs) relative to their paired non-tumor-associated macrophages (NTAMs) from n=33 patients with non-small-cell lung cancer (NSCLC). Data represented correspond to gene expression ratio between TAMs and NTAMs. Upregulation is shown in red and downregulation in blue. (C) Principal component analysis of TAMs and NTAMs from patients with NSCLC (middle plot), and expression of the transcripts encoding M1-related and M2-related products in TAMs and NTAMs (plots along perimeter; M2 left, M1 right). A population of TAMs enriched in high expression of M1 markers is highlighted. Each symbol represents an individual patient sample (one for TAMs and one for NTAMs). (D) Correlation plots showing expression of M2 markers CD209 and ADORA3 versus increasing expression of M1 canonical marker chemokine (C-X-C motif) ligand 9 (CXCL9) across TAM samples from patients with NSCLC (n=40). Statistical significance by Spearman’s correlation analysis. (E) Expression of CXCL9 (M1 canonical marker) and matrix metallopeptidase 12 (MMP12) (M2 canonical marker) across TAMs and paired NTAMs samples ordered by increasing expression of CXCL9 in TAMs (n=33, paired). ns, non-significant for p>0.05,. Related to online supplementary figure S2 and table S3.

10.1136/jitc-2020-000778.supp2 Supplementary data

Bulk RNA-seq analysis revealed major differences in the TAM molecular program; >1000 transcripts (1038) were expressed differentially in TAMs compared with NTAMs (figure 1B and online supplementary table S2), and t-SNE plots showed that TAMs clustered separately from NTAMs (figure 1C, online supplementary figure S1C). IPA revealed strong activation of both protumor and antitumor functions linked to the M2-like and M1-like macrophage gene profiles, respectively^32^ (figure 1D and online supplementary table S2). Transcripts associated with M2 protumor functions, such as angiogenesis (vascular endothelial growth factor signaling), metalloprotease activity, fibrosis and cancer metastasis, were expressed at higher levels in TAMs compared with NTAMs (figure 1E), which was further confirmed at a more global level, by GSEA (figure 1F and online supplementary table S2). As expected, M2-inducing cytokines like IL-4, IL-10, IL-13 and transforming growth factor-β were among the top upstream regulators of TAMs (compared with NTAMs) but surprisingly, so were typical M1-inducing cytokines such as IFN-γ and tumor necrosis factor (TNF)-α^33^ (IPA, figure 1G and online supplementary table S2). This finding might explain the simultaneous activation of both pro-inflammatory and antitumor pathways in TAMs, such as granulocyte adhesion and diapedesis and signaling of leukocyte extravasation, that were predicted by IPA (figure 1D). Together, these data indicate that TAMs in lung cancer expressed higher levels of transcripts linked to both an M2-gene profile, associated with protumor functions, and an M1-gene profile, involved in promoting antitumor T-cell responses.

### Uniform M2 but heterogeneous M1 features among patients

We wondered whether bulk RNA-seq allowed stratification of TAMs in tumor samples according to relative enrichment of M1-like or M2-like signatures (‘hot or cold’), in order to establish differential protumor or antitumor roles. The expression pattern of known M2 genes (n=122 Methods' section^34^) that were significantly upregulated in the TAM population (~40% of known M2 genes), which included *CD209, IL10, WNT5A and MMP12*, was homogeneous across all samples, indicating a uniform M2 signature in TAMs (figure 2A and C and online supplementary table S3). In contrast, the expression pattern of known M1 genes (n=116 Methods' section^34^) that were significantly upregulated in TAMs (~31% of known M1 genes), which included *CXCL9*, *CXCL10*, *CXCL11*, *CXCL12, STAT1* and *AIM2,* was highly heterogeneous and defined a clear subgroup of patients that exhibited ‘M1 hot’ features in their TAMs (figure 2B and C and online supplementary table S3).

We wondered whether in tumors with these M1^hot^ TAMs, there would be a reciprocal relationship with M2 TAMs which would show weaker M2 signatures. We selected *CXCL9* to define M1^hot^ TAMs. CXCL9 is a chemokine previously known as monokine induced by IFN-γ, overexpressed by macrophages after stimulation by IFN-γ (the key cytokine behind M1 signature activation) and is involved in recruitment of antitumor T cells as well as having anti-angiogenic roles and has been suggested as a novel target for cancer therapy.^35^ We thus correlated the expression of CXCL9, as one of the strongest M1 marker genes in macrophages, with key M2 marker genes (*CD209*, *ADORA3*, *STAT6*, *SOCS3*, *IL10* and *IRF4*) (figure 2D, online supplementary figure S2). As before, the M2 markers were homogeneously distributed across tumors, and showed no correlation to the *CXCL9* (M1 marker gene) expression. The expression pattern of *CXCL9* and *MMP12* (a top DEG in figure 2A, strongly associated with M2 signatures^36^) in TAMs was not linked to their expression in matched NTAMs (figure 2E). Overall, these results indicate that TAMs in all lung cancer tumors show a strong M2 gene profile. IPA analysis suggests that this is likely due to the presence of pro-M2/Th2 cytokines such as IL-4 and IL-13, in the tumor microenvironment (figure 1G). In some tumors (25%–35%), however, the concomitant presence of pro-M1/Th1 cytokines (TNF-α and IFN-γ, figure 1G) may also be driving the M1 gene profile overexpression in TAMs.

### Single-cell RNA-seq reveals a TAM subset with dual M1 and M2 features

Next, we wished to ascertain whether the TAM population was a mixture of different proportions of cells manifesting either an M1 or M2 phenotype (as suggested by most of previous literature) or whether individual TAM cells might express both the M1 and M2 signatures. Therefore, we performed single-cell RNA-seq analysis in purified macrophage populations (online supplementary figure S3A, table S1, Methods' section) isolated both from tumor and adjacent normal lung tissue from two additional patients with early stage lung cancer.

Analysis of the single-cell transcriptomic profile of ~9000 cells (Methods' section), revealed 8 distinct clusters (figure 3A and online supplementary figure S3B, C). As expected, transcripts with increased expression in bulk populations of NTAMs compared with TAMs (shown in figure 1B) were expressed at higher levels by single cells in the NTAM-enriched clusters (cluster 0 and 4), and the transcripts with increased expression in bulk populations of TAMs compared with NTAMs were expressed at higher levels by individual cells in the TAM-enriched clusters (cluster 2 and 3) (figure 3B, C and online supplementary figure S3C). While M2-like signature genes were expressed at higher levels (and in a higher proportion of cells) in both the TAM-enriched clusters (cluster 2 and 3), the M1 signature genes were expressed at higher levels (and in a higher proportion of cells) only in cells in cluster 3 (figure 3D–F and online supplementary table S4). The transcripts enriched in cluster 3 were also seen in our bulk population data, tightly co-expressed with *STAT1*, a known master regulator mediating the response to IFN-γ and activating the expression of M1-related genes (figure 3G). Overall, our single-cell transcriptome data confirm our bulk RNA-seq analysis but additionally, demonstrate that M1-related and M2-related genes can be strongly co-expressed in the same individual cells.

Confocal microscopy showed co-expression in TAMs of CXCL9 and MMP12 proteins, which we used as canonical markers of M1-like and M2-like phenotypes, respectively.^34^ NTAMs, expressed very low levels of both markers (figure 3H, online supplementary figure S3D). Together, these findings confirmed the existence of a TAM subset with dual M1-like and M2-like phenotype, overexpressing both gene profiles (M1^hot^), in addition to a TAM subset with exclusive M2-like phenotype, only overexpressing the M2 gene profile (M1^cold^).

### M1^hot^ TAMs are associated with robust T-cell responses in tumor

**Figure 4:**
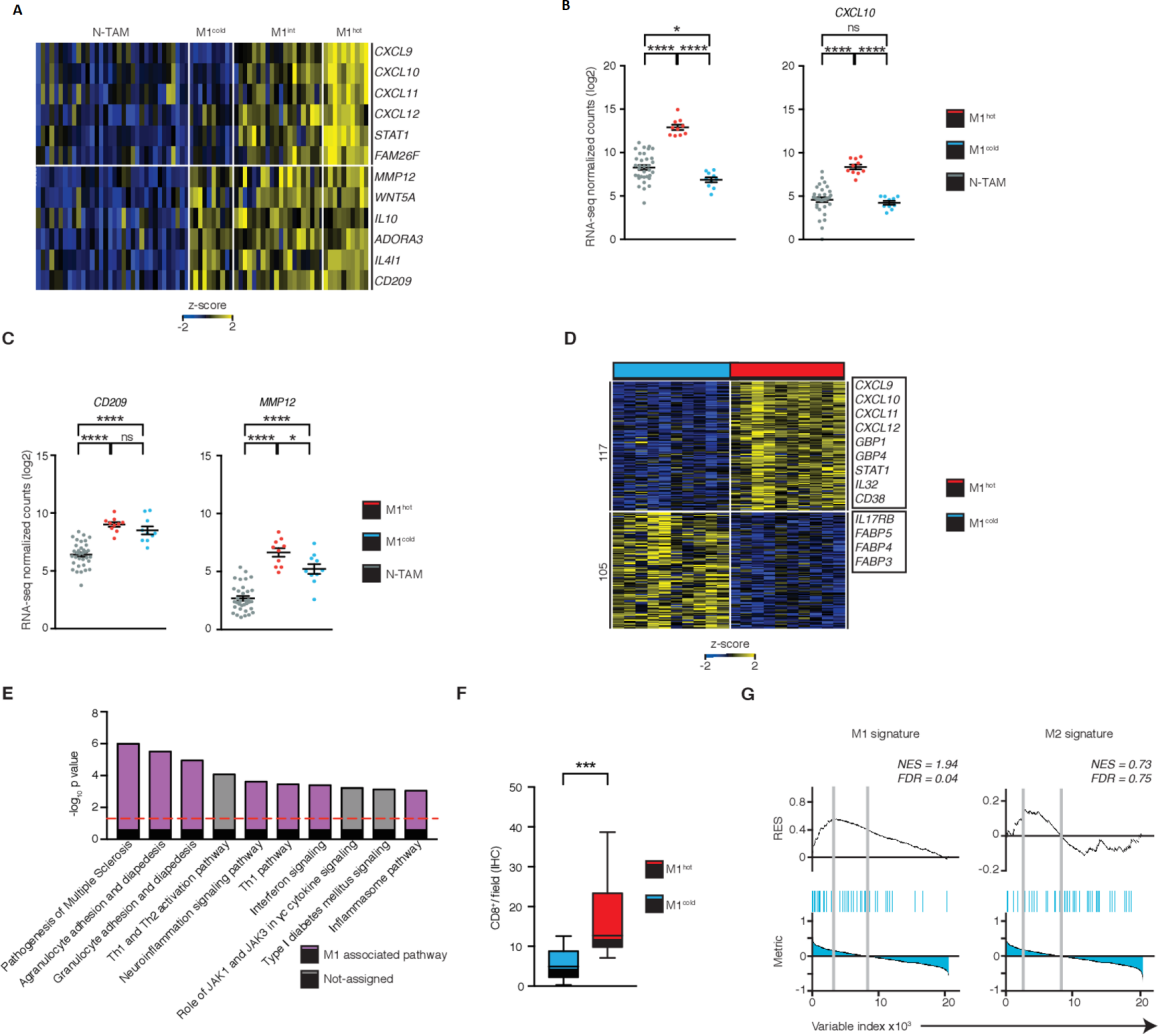
M1^hot^ tumor-associatedmacrophages (TAMs) are associated with robust T-cell responses in tumor. (A) Heatmap showing stratification of patient TAMs (n=40) into M1^cold^, M1 intermediate and M1^hot^, based on their expression of M1 markers chemokine (C-X-C motif) ligand (*CXCL)9*, *CXCL10*, *CXCL11*, *CXCL12*, *STAT1*, *FAM26F*. Expression of M2 markers matrix metallopeptidase 12 (*MMP12*), *WNT5A*, *IL10*, *ADORA3*, *IL4I1*, *CD209* is also shown. Expression of M1 (B) and M2 (C) genes in non-tumor-associatedmacrophages (NTAMs), M1^hot^ TAMs and M1^cold^ TAMs (categorized by expression of *CXCL9* in log_2_ normalized counts, by RNA-sequencing (RNA-seq)). Statistical significance by one-way analysis of variance. (D) Heatmap showing the differential gene expression (DEG) of 222, obtained by DESeq2 analysis between M1^hot^ TAMs (top 25% percentile of *CXCL9* expression by RNA-seq) versus M1^cold^ TAMs (bottom 25% percentile) represented by Row-Z-score across samples (FDR ≤0.05, log_2_fold-change (FC) ≥ |1|, basemean ≥10, n=20). Right margin, genes encoding M1 protumor functions and lipid uptake functions are indicated. Tumor subtype, cancer stage, gender and smoking history of each group were compared in online supplementary figure S4. (E) Top dysregulated canonical pathways based on the expression of 222 DEGs. Statistical values displayed as –log_10_(p value). Purple columns indicate pathways related to M1 functions. Statistical significance baseline (dotted line) corresponds to a p value of 0.05. (F) Box and whisker graph indicates CD8^+^ T-cell infiltration in non-small cell lungcancer (NSCLC) tumors with M1^hot^ TAMs (n=10) versus infiltration in NSCLC tumors with M1^cold^ TAMs (n=10). Statistical significance by Mann-Whitney U test. (G) Gene set enrichment analysis (GSEA) of M1 and M2 gene sets in the transcriptome of TAMs from NSCLC with TIL^high^ TAMs versus that of TIL^low^, presented as the running enrichment score. Statistical significance by Kolmogorov-Smirnov test. Statistical significance expressed as ns for non-significant (p>0.05), *p≤0.05, ***p≤0.001 and ****p≤0.00001. Related to online supplementary figure S4 and table S5.

To understand the potential functional impact of ‘M1^hot^ TAMs’ on antitumor immunity, we classified our cohort of cancer subjects based on the expression of M1 marker genes such as *CXCL9*, *CXCL10*, *CXCL11*, *CXCL12*, *STAT1*, *FAM26F* (heatmap, figure 4A), choosing *CXCL9* as the candidate gene to classify tumors into M1^hot^ (top 25th percentile) M1^intermediate^ (25th–75th percentile) and M1^cold^ (bottom 25th percentile) tumors (figure 4A). The expression levels of *CXCL9*, *CXCL10* and additional M1 marker genes were significantly higher in M1^hot^ TAMs (but not in M1^cold^ TAMs) than in NTAMs, while the transcript levels of *CD209*, *MMP12* and additional M2 marker genes were higher in both M1^hot^ and M1^cold^ TAMs, when compared with NTAMs (figure 4B, C and online supplementary figure S4A). In our patients, M1 status of TAMs (M1^hot^ or M1^cold^) was not related to gender, tumor stage or histological subtype of NSCLC and M1^hot^ status was not more frequent in ‘never-smokers’ (online supplementary figure S4B).

We next compared M1^hot^ TAMs with M1^cold^ TAMs to understand whether transcriptomic differences relate to outcomes in patients with lung cancer. We found that 222 transcripts were differentially expressed between M1^hot^ versus M1^cold^ TAMs (figure 4D); transcripts expressed at higher levels in M1^hot^ TAMs were involved in pathways linked to antitumor T-cell immune responses such as: the recruitment of T_H_1 T cells, by expressing more *CCL5*, *CXCL9*, *CXCL10* and *CXCL11*, antigen presentation, expansion of T cells, cytotoxicity and differentiation of effector T cells (IPA, figure 4E and online supplementary table S5). We found that M1^hot^ tumors had a significantly higher density of CD8^+^ TIL compared with M1^cold^ tumors (figure 4F, n=40). Furthermore, GSEA showed that expression of M1 signature genes in TAMs was strongly associated with a higher CD8^+^ T-cell density in tumors (q=0.04), whereas the expression of M2 signature genes in TAMs was not associated with either a higher or lower CD8^+^ T-cell density (q=0.75) (figure 4G). Taken together, our data indicate that while performing M2 functions, M1^hot^ TAMs appear to be involved in the recruitment and proliferation of T cells and therefore could shape the quality or magnitude of the antitumor response.

### M1^hot^ TAMs are associated with improved survival outcomes

**Figure 5:**
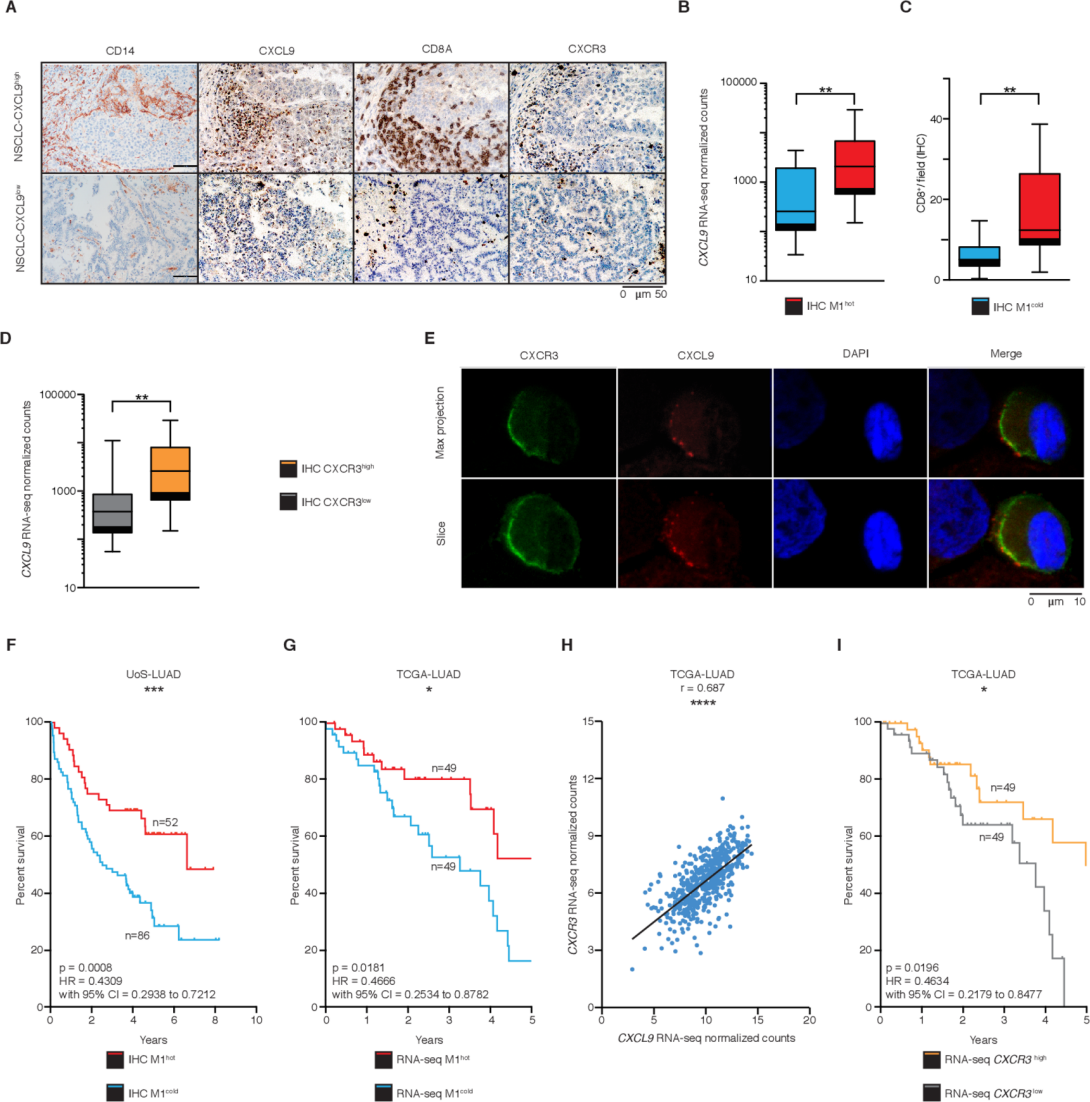
M1^hot^ tumor-associatedmacrophages (TAMs) are associated with improved survival outcomes. (A) Immunohistochemistry (IHC) analysis of CD14^+^, CXCL9^+^, CD8^+^ and CXCR3^+^ cells in two representative samples of non-small cell lungcancer (NSCLC) adenocarcinoma (M1^hot^ and M1^cold^). (B) Chemokine (C-X-C motif) ligand 9 (*CXCL9*) expression in TAMs (RNA-sequencing (RNA-seq) normalized counts), in NSCLC tumors with high CXCL9 tissue expression (IHC; n=17), versus NSCLC tumors with low CXCL9 tissue expression (n=16) (C) CD8^+^ T-cell infiltration in NSCLC tumors with high CXCL9 tissue expression (IHC; n=17), versus NSCLC tumors with low tissue CXCL9 expression (n=16). (D) *CXCL9* expression in TAMs by RNA-seq normalized counts, in NSCLC tumors with high CXCR3 tissue expression (IHC; n=16), versus NSCLC tumors with low CXCR3 tissue expression (n=11). (E) Representative tumor sample showing a T-cell stained for CXCL9 and CXCR3. Scale bar refers to 10 µm. (F) Kaplan-Meier overall survival curve of a retrospective cohort of patients with NSCLC-LUAD (Southampton University Hospital, n=393) stratified into M1^hot^ (samples with >80% positive cells; n=52) M1^intermediate^ (samples with 20%–80% positive cells; n=255) and M1^cold^ (samples with <20% positive cells; n=86) by histological evaluation of presence of CXCL9^+^-cells. (G) Kaplan-Meier overall survival curve of TCGA-LUAD cohort (n=495) comparing patients with high *CXCL9* expression (top 10%; n=49) versus low (bottom 10%; n=49), by RNA-seq. (H) Correlation analysis of *CXCR3* versus *CXCL9* mRNA gene expression in TCGA-LUAD cohort (n=511). Statistical significance by Spearman’s non-parametric test. (I) Kaplan-Meier overall survival curve of TCGA-LUAD cohort (n=495) comparing patients with high *CXCR3* expression (top 10%; n=49) versus low (bottom 10%; n=49), by RNA-seq. Statistical significance of Kaplan-Meier survival plots by Mantel-Cox log-rank test. Statistical significance in B, C and E by Mann-Whitney U test. *P≤0.05, **p≤0.01, ***p≤0.001 and ****p≤0.00001. Related to online supplementary figure S5 and table S6.

We next sought to determine the mechanism by which M1^hot^ TAMs could influence TIL density and hence, survival outcomes. Key defining transcripts of M1^hot^ TAMs encode for potent chemokines (CXCL9, CXCL10, CXCL11, CXCL12) that attract activated T_H_1 T cells.^37 38^ Therefore, we asked whether the presence of transcriptomically M1^hot^ TAMs was reflected in CXCL9 protein expression and whether the density of CXCL9 protein-expressing TAM correlated with a CD8^+^ T-cell density. Immunostaining on TMA of tumor samples (figure 5A) showed that high CXCL9 protein expression in TAM was indeed associated with higher expression of *CXCL9* mRNA by TAMs (ie, presence of M1^hot^ TAMs) (figure 5B) and a greater CD8^+^ T-cell density (figure 5C). CXCL9 is a known chemoattractant for T cells expressing CXCR3.^37^ We observed that a higher expression of *CXCL9* transcripts in TAMs was associated with a higher infiltration of CXCR3^+^ cells in the tumor (CXCR3^high^, figure 5D). Confocal microscopy on cells disaggregated from lung tumors showed that CXCR3 co-localizes with CXCL9 in the membrane of T cells (figure 5E). Taken together, these results suggested that there was a strong CXCL9-dependent infiltration of CXCR3-expressing CD8^+^ T cells in tumors (figure 5A and C–E) and that M1^hot^ TAMs were the main source of CXCL9 (figure 5B).

10.1136/jitc-2020-000778.supp4 Supplementary data

10.1136/jitc-2020-000778.supp3 Supplementary data

We hypothesized that M1^hot^ TAMs are associated with better prognosis for patients with lung cancer, since they link with higher CD8^+^ T-cell tumor infiltration. We assessed the survival outcome for patients whose tumors were classified by the density of cells expressing CXCL9 (a representative M1-like gene) into M1^hot^, M1^intermediate^ and M1^cold^ (Methods' section). This assessment was done in an independent, large cohort of predominantly early stage patients with lung cancer (total n=393; a majority of them stage I to IIIA, online supplementary table S6) who had been diagnosed from 2007 to 2011 and followed up until 2016, a cohort with similar characteristics to the one used to generate the transcriptomic analysis of this study. Consistent with our finding that M1^hot^ TAMs are linked to a stronger antitumor immune response, M1^hot^ tumors were associated with a 48% survival outcome at 10 years, compared with a 24% 10-year survival in M1^cold^ tumors. The two-group comparison (M1 hot vs M1 cold) is shown in figure 5F (n=138, p=0.0006, Methods' section; Kaplan-Meier plot with log-rank test p value), and the three group comparison (including intermediate group) is shown in online supplementary figure S5A (n=393, Methods' section; Kaplan-Meier plot with log-rank test p value).

In order to broaden the validity of this important finding, we interrogated the RNA-seq dataset published by TCGA. We classified tumors based on the expression of *CXCL9* transcripts (Methods' section), as M1^hot^ and M1^cold^; we then compared the survival of patients classified as having M1^hot^ or M1^cold^ tumors from both the TCGA lung cancer adenocarcinoma dataset (TCGA-LUAD, total n=495 patients with complete survival information; M1^hot^=49, M1^cold^=49) and from data available from our Southampton cohort (n=393). Importantly, the M1^hot^ tumors showed better survival outcomes in both TCGA and Southampton datasets (figure 5F, G and online supplementary table S6 including a univariate and multivariate analysis of overall survival). Using the TCGA database, we confirmed that in TCGA-LUAD, *CXCL9* transcript levels correlated strongly with *CXCR3* transcript levels, reflecting the presence of higher numbers of T cells (n=511, figure 5H), which validated our observation in figure 5D. Higher expression of *CXCR3* transcript levels was also linked to better survival (figure 5I). Overall, these results suggested that an M1^hot^ status of a tumor is positively linked to better survival outcomes in lung cancer through recruitment of a better antitumor TIL response.

### M1^hot^ TAMs support T_RM_ cell maintenance by allowing uptake of fatty acids

**Figure 6:**
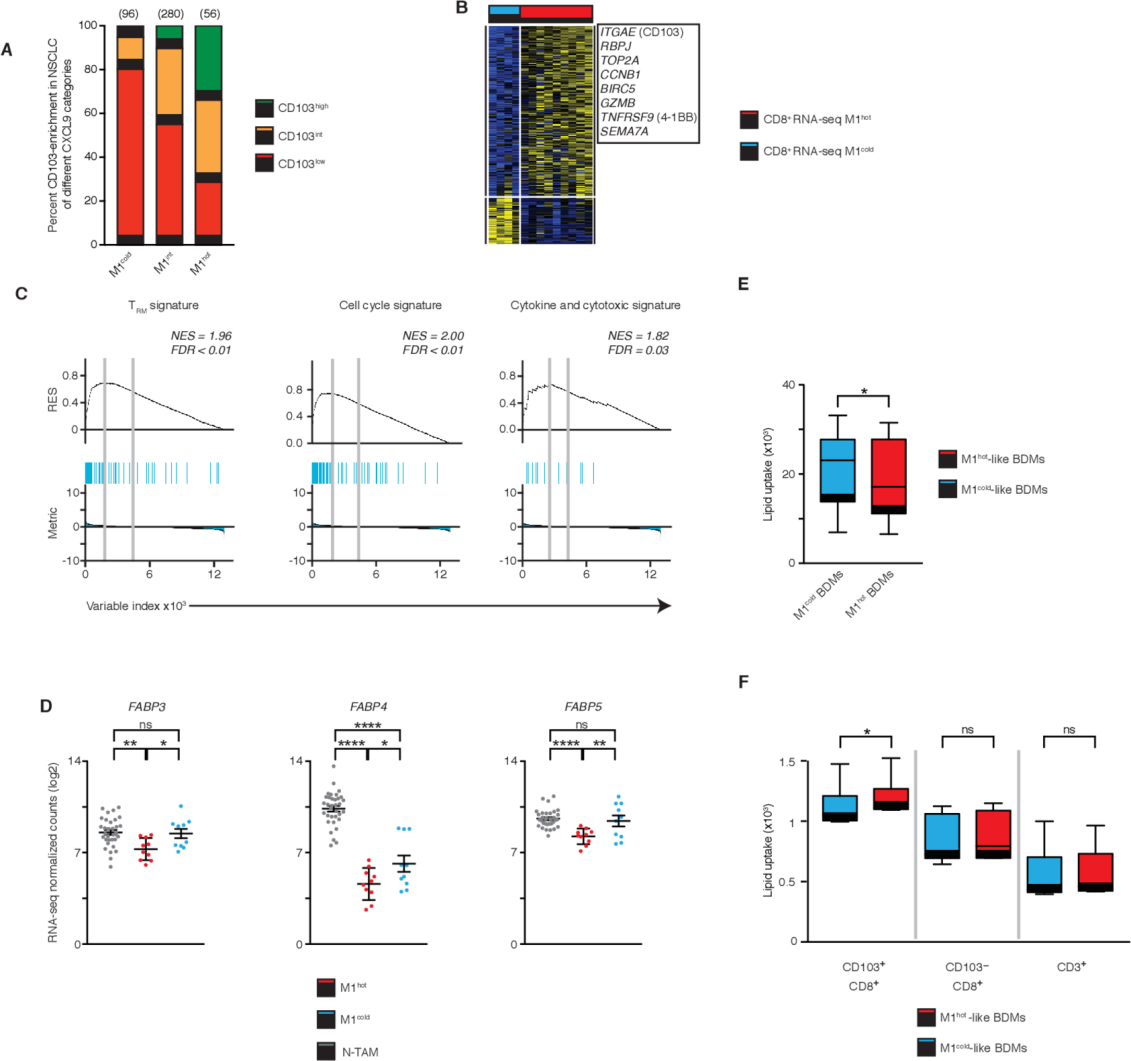
M1^hot^ tumor-associatedmacrophages (TAMs) support tissue-resident memory T cells (T_RM_) cell maintenance by allowing uptake of fatty acids. (A) Frequency of CD103^high^, CD103^intermediate^ and CD103^low^ tumors among those categorized by chemokine (C-X-C motif) ligand 9 (*CXCL9*) expression in TAMs (M1^hot^, M1^intermediate^, M1^cold^; numbers of patients above each bar). (B) Heatmap showing expression of genes related to Tissue Resident Memory features (T_RM_ phenotype) in CD8^+^ T cells isolated from the same tumors from patients with non-small cell lungcancer (NSCLC) were M1^hot^ TAMs and M1^cold^ TAMs were isolated and studied. (C) Gene set enrichment analysis (GSEA) of 246 gene sets in the transcriptome of CD8^+^ T cells from patients with NSCLC with M1^hot^ TAMs versus those with M1^cold^ TAMs, presented as the running enrichment score. Statistical significance by Kolmogorov-Smirnov test. (D) Expression of FABP3, FABP4 and FABP5 in non-tumor-associatedmacrophages (NTAMs), M1^hot^ TAMs and M1^cold^ TAMs (log_2_ normalized counts). Statistical significance by ordinary one-way analysis of variance. (E) Macrophages derived from blood (BDM) treated with interfern (INF)-γ/lipopolysaccharides (LPS) (M1^hot^-like BDM, red) or interleukin (IL)-4 (M1^cold^-like BDM, blue) cultured with bodipy lipid probe for 30 min (n=7). Lipid uptake measured by flow cytometry and expressed as mean fluorescence intensity. Wilcoxon test, two-tailed. *P≤0.05 (F) M1^hot^-like BDM (red) or M1^cold^-like BDM (blue) were co-cultured with CD3^+^ cells and bodipy lipid probe for 30 min (n=6). Uptake of lipid represented as Mean fluorescence intensities (MFI) in T_RM_ (CD8^+^CD103^+^), CD8^+^ (CD8^+^CD103^-^) and CD3^+^ (CD8^-^CD103^-^) cells was determined by flow cytometry. Wilcoxon test, two-tailed. *P≤0.05. ns, non-significant for p value >0.05, *p≤0.05, **p≤0.01 and ****p≤0.00001. Related to online supplementary figure S6 and tables S5, S6.

In patients with solid tumors, it has recently been shown that the density of intratumoral CD8^+^ T_RM_ is associated with better survival outcomes.^2^ Therefore, we asked if M1^hot^ tumors were also associated with a higher density of T_RM_ cells in tumors. In our historical cohort of patients with lung cancer (n=393), whose tumors were preclassified on the basis of the density of cells expressing CXCL9 (M1^hot^, M1^intermediate^, M1^cold^), we determined the density of cells expressing CD103, a marker of T_RM_ cells (T_RM_^high^, T_RM_^int^ or T_RM_^low^). We found a clear positive association in that T_RM_^high^ tumors were frequently also M1^hot^ tumors (34% vs 5% among M1^cold^ tumors). Similarly, T_RM_^low^ tumors were mainly also M1^cold^ tumors (80% vs 28% among M1^hot^ tumors) (figure 6A and online supplementary table S6). To directly assess the association between M1^hot^ TAMs and T_RM_ cells, we compared the transcriptome of tumor-infiltrating CD8^+^ T cells^2^ from the same patients with cancer in whom we had assessed the TAM phenotype as M1^hot^ and M1^cold^ (figure 6B and online supplementary table S5). Confirming our previous data, we found that in tumors with M1^hot^ TAMs compared with M1^cold^ TAMs, the tumor-infiltrating CD8^+^ T cells showed increased expression of genes strongly enriched for T_RM_ signature genes (eg, *ITGAE*, *RBPJ*) in addition to cell cycle and cytotoxic/cytokine signature genes (figure 6B, C). This result suggested that M1^hot^ TAMs were tightly linked to robust T_RM_ responses in the tumor.

We next asked how M1^hot^ TAMs might influence or modulate T_RM_ responses in the tumor microenvironment. M1^hot^ TAMs, through expression of CXCL9, 10 and 11, may enhance the local mobility of T_RM_ cells expressing CXCR3. However, further processes are required to sustain the antitumor activities of the recruited T_RM_ cells and a possible mechanism was suggested by our observation that the M1^hot^ TAMs showed reduced expression of transcripts encoding for the fatty acid binding proteins FABP3, FABP4 and FABP5, when compared with M1^cold^ TAMs (figures 4D and 6D). It is known that for survival in the tissues, T_RM_ cells depend on uptake of essential nutrient fatty acids through FABP4/5.^39^ Also, it has been shown that macrophages treated with IL-4, (‘M1^cold^’), increase their fatty acid uptake.^40^ Hence, we hypothesized that M1^cold^ TAMs will be more efficient than M1^hot^ TAMs in competing for fatty acids in the tumor microenvironment (ie, due to higher expression of FABP3, 4, 5), and hence will outcompete T_RM_ cells for this essential nutrient, thus compromising long-term maintenance of T_RM_ cells in tumors.

We tested this hypothesis experimentally by treating blood-derived macrophages (BDMs) with IFN-γ and LPS or with IL-4 in order to mimic M1^hot^ and M1^cold^ TAMs, respectively.^34^ M1^hot^-like BDMs (IFN-γ and LPS) showed a reduction in fatty acid uptake when compared with M1^cold^-like BDMs (IL-4) (figure 6E); similar results were observed when FABP3, 4 and 5 were knocked down using siRNA to mimic the expression pattern observed in M1^hot^ TAMs (online supplementary figure S6C). We then co-cultured M1^hot^-like BDMs or M1^cold^-like BDMs with T cells isolated from blood of the same donors and observed that CD8^+^CD103^+^ T cells showed a significantly increased uptake of fatty acids when co-cultured with M1^hot^-like BDM, compared with co-culture with M1^cold^-like BDM (figure 6F). CD8^+^CD103^-^ T cells and the rest of CD3^+^ T cells used as controls, did not show significant differences in lipid uptake, regardless of the type of BDM with which they were co-cultured.

Our experiments suggested that M1^cold^ TAMs may reduce the survival and maintenance of T_RM_ cells in tumors (80% of M1^cold^ TAM tumors are T_RM_^low^) and that a possible mechanism may be intercellular competition for lipid nutrients.

## Discussion

Our results have shown first that TAMs have a dramatically altered transcriptional program compared with their counterparts in normal lung (NTAMs) (>1000 DEGs). This is further confirmation of the recognized interaction between tumor-infiltrating macrophages and the complex matrix of stimuli present in the tumor microenvironment.^4 5 8^ The most significant findings in our data are first, that all NSCLC tumors contain TAMs expressing an M2-like signature and there is no significant variation in the strength of this M2-like signature across tumors, regardless of survival outcomes. Second, we have shown that in some patients (25%), these M2-like TAMs express simultaneously, a strong/hot signature of M1-like genes. Thus, the M1-like and M2-like functional signatures are not mutually exclusive but are in fact found in variable degrees in the same cells, reflecting plasticity in TAMs. The third critical finding we present is that the prognostic outcome for patients with NSCLC is not determined by the M2-like features in their tumors but by the balance between the M1^cold^ and M1^hot^ populations—the M1^hot^ tumors having a much stronger infiltration of T_RM_ cells.

While most studies on TAMs, based on limited surface markers and in vitro or in vivo mouse models, have suggested that TAMs exist as distinct subpopulations with non-concurrent M1/antitumor or M2/protumor activities,^41–43^ recent work suggests that M1 and M2 signatures may coexist, using single cells RNA-seq^44^ or CyTOF.^45 46^ The low number of samples in those studies (n=8, n=28, n=78, respectively, compared with n=393 in our study), precludes them from drawing robust conclusions regarding clinical outcomes as they can neither stratify TAMs to define their role in patient survival, nor are they able to reveal the link of TAMs with the infiltration of other immune cells. We are the first to establish a positive link to protective T cells responses. Our data further raise doubts about the dogma of an immune suppressive role of M2 TAMs and suggest a general presence of M2 TAMs in early lung cancer where the outcome is then modulated by the additional presence of M1^hot^ TAMs. In both the Southampton (n=393) and TCGA cohorts of lung adenocarcinoma patients (n=495), M1^hot^ TAMs are associated with better survival despite a concurrent background expression of an M2 gene signature. These findings concern predominantly lung adenocarcinoma and not all NSCLC. A key target for future research studies will be the elucidation of the factors in the tumor microenvironment that determine which tumors will develop an M1^hot^ or M1^cold^ signature.

There is now strong evidence that the density of tumor-infiltrating T_RM_ cells has a positive association with outcome in lung cancer.^2 47^ However, it is not known how T_RM_ cells are attracted and maintained in tumors. M1^hot^ TAMs expressed high levels of transcripts encoding for CXCL9, 10, 11 and 12 which are strong chemoattractants and activators of T cells, acting through CXCR3, a receptor highly expressed in CD4^+^ Th1 T cells and in antigen-specific T_RM_ cells.^48–50^ Confirming their potential role in attracting antitumor T cells, M1^hot^ TAMs were associated with higher infiltration of CD8^+^ and CXCR3^+^ T cells. Long-term residency and maintenance of active T_RM_ cells in normal lung has been shown to depend on persistent viral antigenic signals.^51 52^ M1^hot^ TAMs expressed higher levels of antigen-presenting molecules, which suggested that local presentation of tumor antigen(s) by M1^hot^ TAMs may help maintain antitumor T_RM_ cells.

T_RM_ cells seem to depend critically on adequate uptake of fatty acids, mediated by FABP4 and FABP5.^39^ M1^cold^ TAMs show upregulated expression of FABP3, 4 and 5, which may facilitate increased uptake of fatty acids. It is possible that significant intercellular competition for essential fatty acids in the tumor microenvironment could, in the long term, deprive T_RM_ cells of essential fatty acids needed for their survival. In a short-term in vitro intercellular competition assay, we showed reduction in fatty acid uptake by T_RM_ cells in the presence of macrophages. However, these experiments have been performed with CD103^+^ T cells from PBMCs, rather than those from tumor and thus further work is warranted to confirm these preliminary findings. Furthermore, in vivo experiments will be needed to assess the long-term impact of this on T_RM_ cell survival and antitumor activity as well as the role of different cells in the tumor microenvironment. The clinical association between M1^cold^ TAMs and the lack of T_RM_ is very robust; in patients with lung cancer, we found that 80% of the tumors infiltrated by M1^cold^ TAMs had a low density of intratumoral T_RM_ cells. Consistent with this, in M1^hot^ TAM tumors, the transcriptome of tumor-infiltrating CD8^+^ T cells showed enriched T_RM_ signatures. Together, this suggests that the TAM gene profile is likely to influence the magnitude of antitumor T_RM_ responses. Further studies are required to address whether exogenously inducing an M1 program in TAMs would enhance antitumor T_RM_ immune responses in lung cancer as well as other types of cancer.

## Conclusions

Our findings should lead to changes in the thinking about therapeutic approaches aimed to enhance the adaptive antitumor immune responses. Most of the current forms of cancer immunotherapy seek to target molecules on cytotoxic T cells such as cytotoxic T-lymphocyte-associated protein 4 or programmed cell death protein 1, aiming to remove inhibition and hence, boost activation of such antitumor effector cells. Additionally, therapeutic strategies are being explored which focus on eliminating TAMs, based on the presumed protumor nature of M2 activation.^53 54^ Our results clearly indicate that a re-think is called for and that a more productive approach will be to develop therapeutic approaches by which TAM populations can be re-programmed to generate a population of M1^hot^ TAMs. Examples of this approach have already been tried in murine cancer models.^11 55 56^ This appears likely to be accompanied by augmented recruitment and activation of CD8^+^ TILs and T_RM_ with enhanced antitumor activities. Again, evidence supporting this possibility has been generated in a murine model of breast cancer.^11 56^ Once this can be achieved, it opens the way for even greater augmentation of the efficacy of the antitumor adaptive immune responses induced by existing T-cell targeting immunotherapies.
