# Supplementary material for: M1hot tumor-associated macrophages boost tissue-resident memory T cells infiltration and survival in human lung cancer
Source: J Immunother Cancer. 2020 Jul 21;8(2):e000778. doi: 10.1136/jitc-2020-000778 (PMC7375465; doi:10.1136/jitc-2020-000778)
Supplement: Supplementary data [file jitc-2020-000778supp001.pdf]

Figure S1.

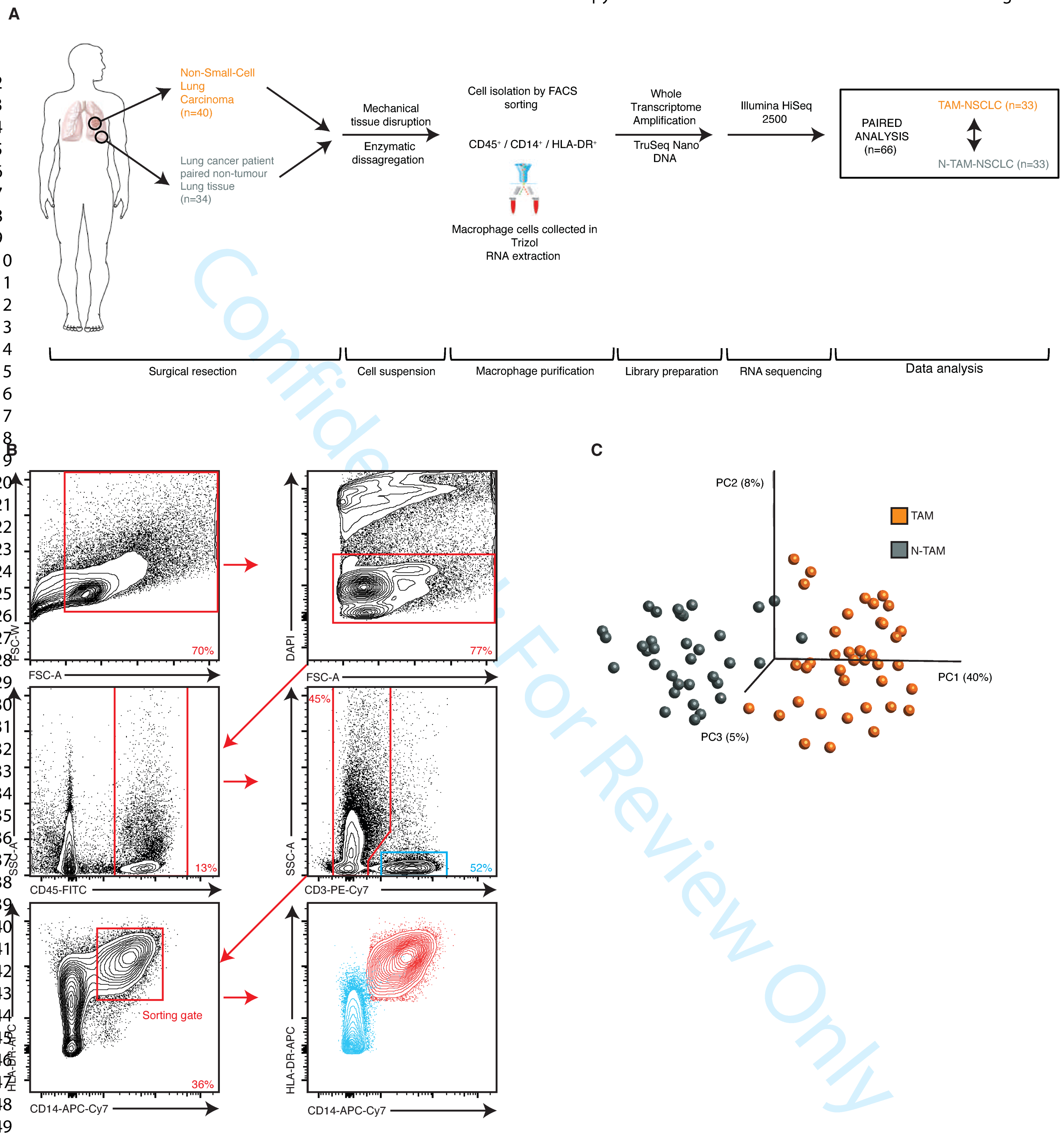

Confidential: For Review Only

Figure S2.

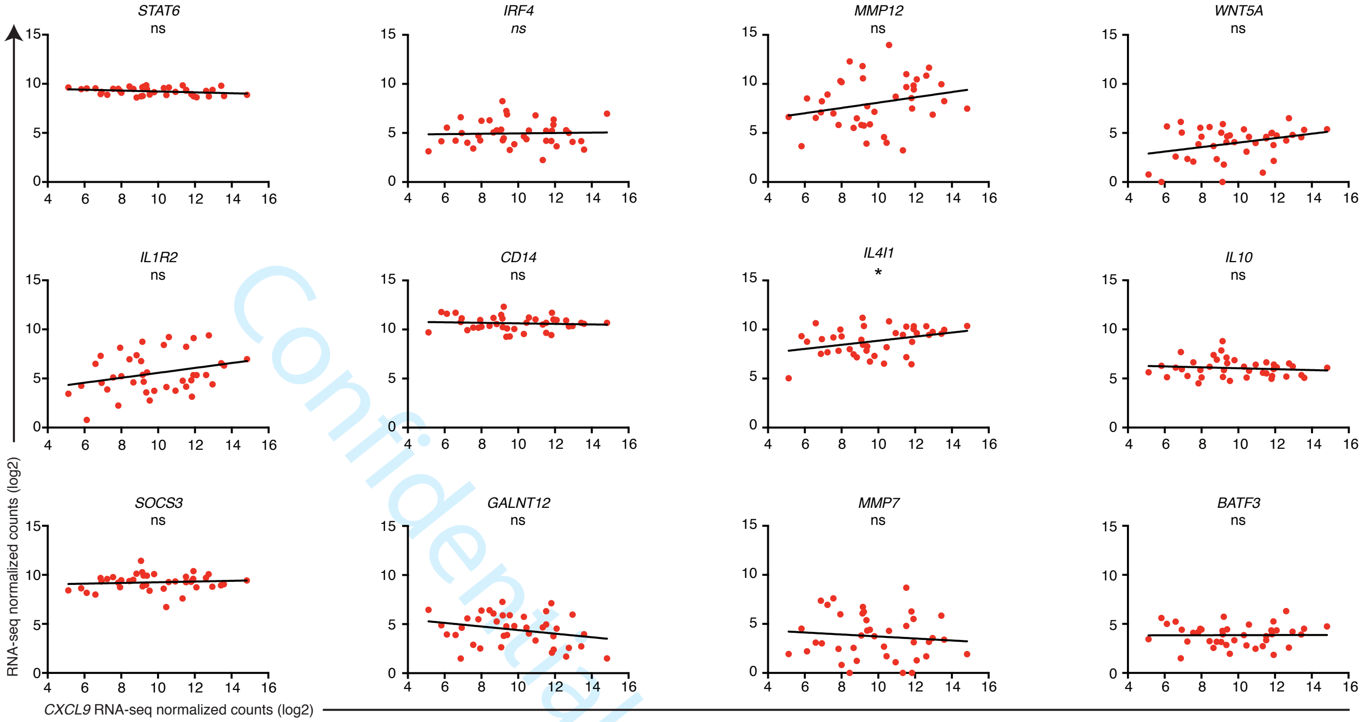

Confidential: For Review Only

Garrido-Martin EM, et al. J Immunother Cancer 2020; 8:e000778. doi: 10.1136/jitc-2020-000778

Figure S3

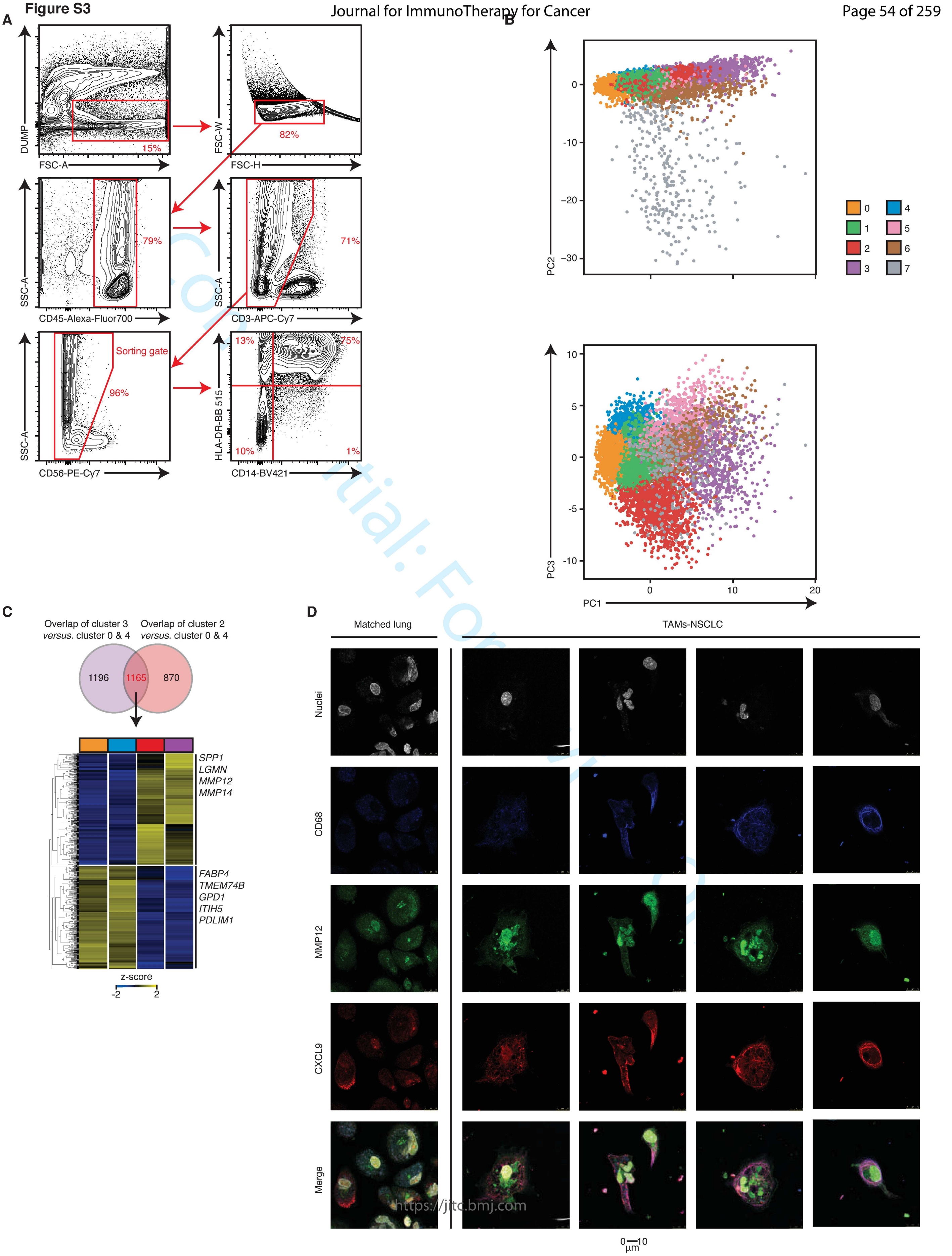

https://jitc.bmj.com

Figure S4.

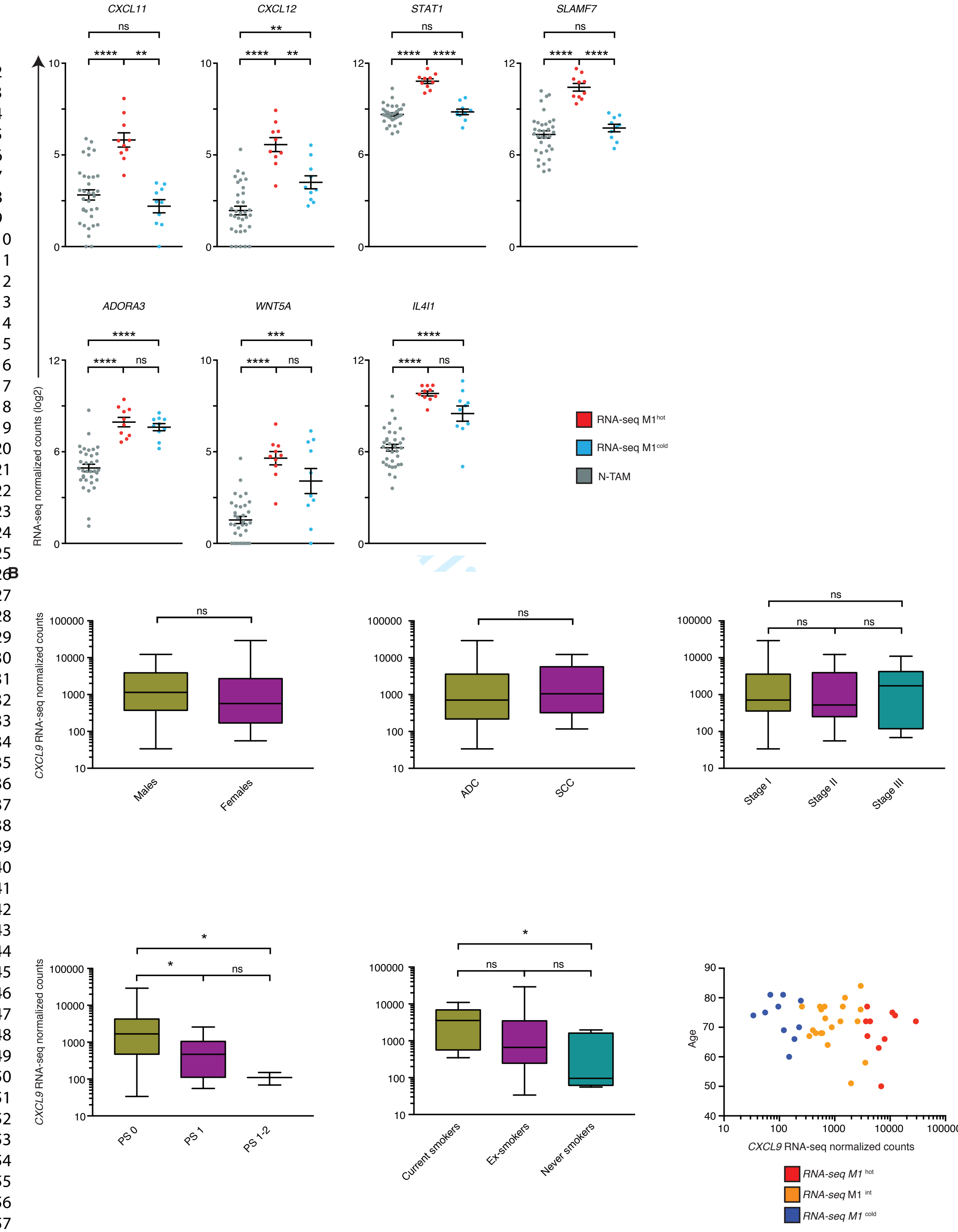

Garrido-Martin EM, *et al. J Immunother Cancer* 2020; 8:e000778. doi: 10.1136/jitc-2020-000778

Figure S5.

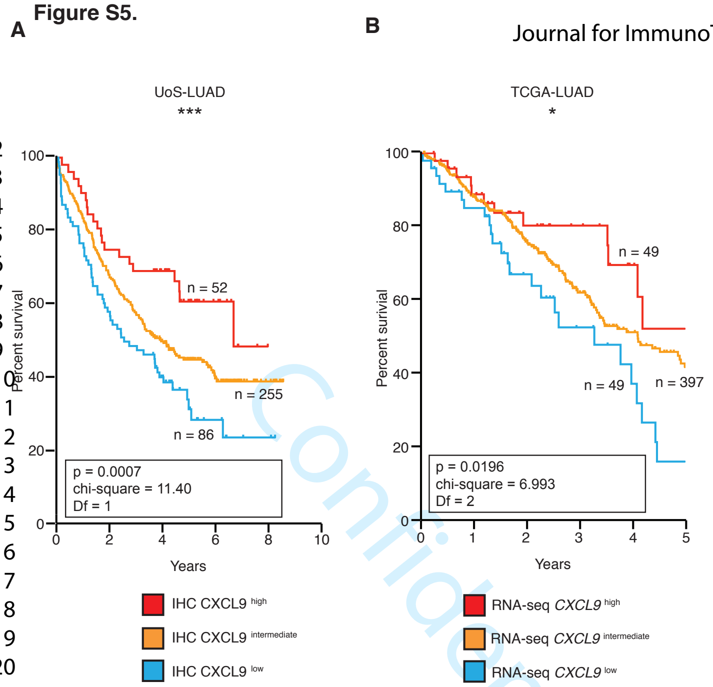

Confidential: For Review Only

Figure S6.

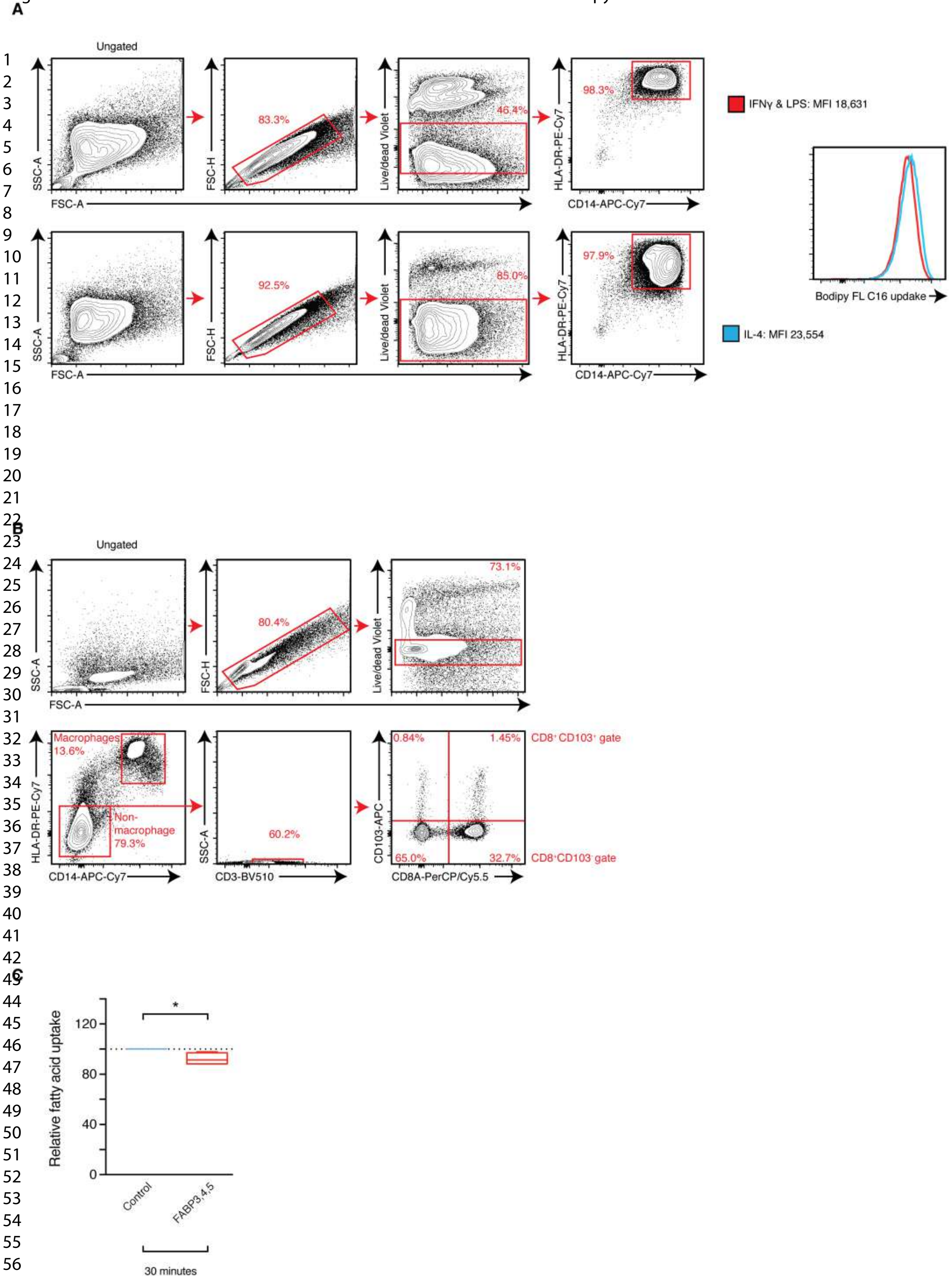

Garrido-Martin EM, *et al. J Immunother Cancer* 2020; 8:e000778. doi: 10.1136/jitc-2020-000778

Supplemental material
BMJ Publishing Group Limited (BMJ) disclaims all liability and responsibility arising from any reliance placed on this supplemental material which has been supplied by the author(s)
J Immunother Cancer

Demographics from whole cohort of NSCLC patients (n=41). From n=40 patients there was
n=33 patients were used in the paired analysis TAMs/NTAMs (availability of tumour and r

TargetLung ID	Used in paired analysis	Samples sequenced	Stage
52	No	only TAMs	IIB
62	Yes	TAMs and N-TAMs	IIA
68	Yes	TAMs and N-TAMs	IA
70	Yes	TAMs and N-TAMs	IIA
71	No	only TAMs	IB
72	No	only TAMs	IA
74	No	only TAMs	IB
80	Yes	TAMs and N-TAMs	IB
81	No	only TAMs	IB
88	Yes	TAMs and N-TAMs	IA
91	Yes	TAMs and N-TAMs	IIIB
96	Yes	TAMs and N-TAMs	IIIA
97	No	only TAMs	IA
99	Yes	TAMs and N-TAMs	IIA
103	Yes	TAMs and N-TAMs	IIA
105	Yes	TAMs and N-TAMs	IA
113	Yes	TAMs and N-TAMs	IB
116	No	only TAMs	IV
117	Yes	TAMs and N-TAMs	IIIA
121	Yes	TAMs and N-TAMs	IIIA
132	Yes	TAMs and N-TAMs	IIIA
135	Yes	TAMs and N-TAMs	IIB
137	Yes	TAMs and N-TAMs	IIB
149	Yes	TAMs and N-TAMs	IB
150	Yes	TAMs and N-TAMs	IA
153	Yes	TAMs and N-TAMs	IIA
156	Yes	TAMs and N-TAMs	IB
159	Yes	TAMs and N-TAMs	IA
161	Yes	TAMs and N-TAMs	IB
163	Yes	TAMs and N-TAMs	IB
164	Yes	TAMs and N-TAMs	IA
167	Yes	TAMs and N-TAMs	IB
179	Yes	TAMs and N-TAMs	IIIA
186	Yes	TAMs and N-TAMs	IA
192	Yes	TAMs and N-TAMs	IIIA
196	Yes	TAMs and N-TAMs	IIB
198	No	only N-TAMs	IA
203	Yes	TAMs and N-TAMs	IB
210	Yes	TAMs and N-TAMs	IA

Garrido-Martin EM, *et al. J Immunother Cancer* 2020; 8:e000778. doi: 10.1136/jitc-2020-000778

227	Yes	TAMs and N-TAMs	IIB
228	Yes	TAMs and N-TAMs	IA

WBC = White Blood Cell count

Confidential: For Review Only

Supplemental material BMJ Publishing Group Limited (BMJ) disclaims all liability and responsibility arising from any reliance placed on this supplemental material which has been supplied by the author(s) J Immunother Cancer

s tumour tissue available, from n=34 there was normal tissue available.
matched tumour-free lung tissue)

LUAD/LUSC	T status	N status	M status	Age	Gender
LUAD	3	0	0	75	F
LUAD	2b	0	0	72	F
LUAD	1a	0	0	66	M
LUAD	1b	1	0	84	F
LUAD	2a	0	0	72	F
LUAD	1b	0	0	72	M
LUAD	2a	0	0	77	F
LUAD	2a	0	0	77	F
LUSC	2a	0	0	67	M
LUSC	1a	0	0	81	M
LUAD	4	2	0	81	F
LUAD	4	0	0	72	F
LUSC	1b	0	0	68	M
LUSC	2b	0	0	68	M
LUAD	2a	1	0	68	F
LUAD	1b	0	0	69	M
LUAD	2a	0	0	63	M
LUAD	1a	0	1B	72	M
LUSC	3	1	0	75	M
LUAD	2b	2	0	66	M
LUAD	2b	2	0	77	F
LUSC	3	0	0	67	M
LUSC	3	0	0	79	M
LUSC	2a	0	0	80	M
LUAD	1a	0	0	58	F
LUAD	2a	1	0	77	F
LUAD	2a	0	0	51	F
LUAD	1b	0	0	70	F
LUAD	2a	0	0	73	M
LUAD	2a	0	0	69	F
LUAD	1a	0	0	77	M
LUAD	2a	0	0	76	M
LUAD	3	2	0	70	M
LUSC	1b	0	0	76	M
LUAD	2b	2	0	77	M
LUAD	2b	0	0	50	M
LUAD	1a	0	0	87	M
LUAD	2a	0	0	64	M
LUAD	1a	0	0	74	M

Garrido-Martin EM, *et al. J Immunother Cancer* 2020; 8:e000778. doi: 10.1136/jitc-2020-000778

LUSC	2b	1	0	74	M
LUAD	1b	0	0	60	F

Confidential: For Review Only

Performance status	Smoking status	Pack-year	Asbestos exposure
1	Never-smoker	0	No
0	Never-smoker	0	No
0	Ex-smoker	N/A	No
0	Ex-smoker	N/A	No
0	Ex-smoker	30	No
0	Ex-smoker	50	No
1	Ex-smoker	60	No
0	Ex-smoker	15	No
0	Ex-smoker	30	Yes
1	Ex-smoker	90	Yes
1-2	Never-smoker	0	No
0	Current-smoker	25	No
0	Current-smoker	12	No
0	Ex-smoker	63.75	No
1	Ex-smoker	50	No
0	Ex-smoker	5	No
0	Ex-smoker	63	No
1	Ex-smoker	30	No
0	Current-smoker	40	Yes
0	Ex-smoker	29	Yes
1	Never-smoker	0	No
1	Current-smoker	30.1	Yes
0	Ex-smoker	25	No
1	Ex-smoker	30	No
0	Current-smoker	40	No
0	Ex-smoker	8	No
0	Never-smoker	0	No
1	Ex-smoker	10	No
0	Ex-smoker	55	No
0	Ex-smoker	15	No
0	Ex-smoker	7.5	No
0	Ex-smoker	25	No
1	Ex-smoker	50	Yes
0	Current-smoker	60	No
0	Ex-smoker	55	Yes
0	Current-smoker	25	No
0	Ex-smoker	N/A	No
0	Ex-smoker	70	Yes
0	Ex-smoker	40	Yes

Garrido-Martin EM, *et al. J Immunother Cancer* 2020; 8:e000778. doi: 10.1136/jitc-2020-000778

0	Ex-smoker	50	No
1-2	Ex-smoker	25	No

Confidential: For Review Only

Garrido-Martin EM, et al. J Immunother Cancer 2020; 8:e000778. doi: 10.1136/jitc-2020-000778

Hb (g/L)	WBC (*E09/L)	Neutrophils (*E09/L)
121	10.4	7.3
124	10.1	6.7
158	9.2	7
145	8.2	5.1
101	12	9.9
100	10.1	8.1
135	14.7	10.5
130	7.8	4.2
139	9.8	6.8
133	5.9	3.8
121	5.5	3.1
161	12.8	8.7
162	10.1	6
107	22.5	17.5
127	8.9	6.6
136	8.8	6.9
140	9.1	5.4
158	5.3	3
95	12.2	9.7
141	10.7	7.5
148	6.4	4.2
142	6.4	3.6
148	16.8	10.6
125	19.5	16.2
145	69	3.6
128	6.4	3.4
135	5.4	3.1
128	5.4	2.5
123	139	11.2
126	3.8	2
135	6.4	3.8
86	11.3	9.9
95	10.5	8.5
125	9.6	5.7
130	6.5	4.8
134	10.8	5.9
130	9	6.4
131	7.7	5.3
129	10.1	5.5

w Only

140	11.1	6.2
133	11.5	7.7

Confidential: For Review Only

Lymphocytes (*E09/L)	Platelets (*E09/L)	Albumin (g/L)	ALK mut
2.2	430	37	NEG
2.7	338	36	NEG
1.4	247	42	NEG
2.2	223	37	NEG
1.3	350	27	NEG
0.7	434	20	NEG
3	402	35	NEG
2.6	75	40	NEG
2	287	5	NEG
1.3	342	43	Not done
1.8	272	37	NEG
2.1	205	37	NEG
3.2	256	46	NEG
1.9	405	23	NEG
1.6	246	46	NEG
1.2	265	41	Not done
2.5	288	Not recorded	NEG
1	229	39	NEG
1.4	24	29	NEG
2.2	332	39	NEG
1.4	261	38	NEG
1.7	261	38	NEG
4.3	245	39	NEG
2.3	32	457	NEG
2.6	257	40	NEG
2.3	261	40	NEG
1.7	334	37	NEG
2.1	117	Not recorded	NEG
1.7	323	46	NEG
1.3	240	31	NEG
1.9	247	25	NEG
1	156	25	NEG
0.9	471	18	NEG
3	307	43	NEG
1.3	192	31	NEG
3.9	400	35	NEG
1.6	231	Not recorded	NEG
1.5	274	25	NEG
3.8	313	43	Not done

Garrido-Martin EM, *et al. J Immunother Cancer* 2020; 8:e000778. doi: 10.1136/jitc-2020-000778

3.1	302	32	NEG
2.7	344	40	NEG

Confidential: For Review Only

EGFR mut	Mutation description	CD8+/field
NEG		6.26
NEG		28.07
NEG		10.11
NEG		11.44
NEG		38.70
NEG		15.56
YES	EGFR1 c2573 p.Leu858Arg	10.81
NEG		1.93
NEG		14.70
Not done		0.67
NEG		4.26
NEG		9.85
NEG		2.67
NEG		17.52
NEG		9.19
POS	EGFR1 deletion exon 19	2.81
NEG		21.22
NEG		10.29
NEG		9.74
NEG		9.56
YES	EGFR1 deletion exon 19	4.81
NEG		4.41
NEG		0.30
NEG		14
NEG		29.11
YES	EGFR1 deletion exon 19	6.33
YES	EGFR1 deletion exon 19	9.59
NEG		12.56
NEG		3.70
NEG		6.78
NEG		15.78
NEG		6.44
NEG		4.11
NEG		2.70
NEG		29.78
NEG		7.11
NEG		Not applicable (no tumour tissue available)
NEG		5.00
Not done		5.04

BMJ Publishing Group Limited (BMJ) disclaims all liability and responsibility arising from any reliance placed on this supplemental material which has been supplied by the author(s)

NEG		10.74
NEG		8.59

Confidential: For Review Only

Garrido-Martin EM, *et al. J Immunother Cancer* 2020; 8:e000778. doi: 10.1136/jitc-2020-000778

CD3+/field	CD4+/field
25.26	13.56
60.08	23.67
39.63	18.67
44.42	22.5
48.15	7.33
39.93	15.44
33.81	18.44
32.30	17.56
30.00	6.78
3.67	2.33
17.15	7.78
27.77	30.89
6.93	5
32.78	14.89
43.15	21.67
13.59	15.11
49.93	31.22
33.52	31.33
17.11	19.78
23.44	9.33
14.67	14.89
13.59	3.44
0.92	2.89
33	35.22
50.73	27.89
18.90	17.89
34.17	24.56
29.35	28.89
8.26	7.78
20.20	16.67
47.75	28.56
10.85	10.89
4.88	4.78
13.35	6.22
48.11	31.78
9.50	5.56
Not applicable (no tumour tissue available)	Not applicable (no tumour tissue available)
5.67	9.00
5.04	3.89

Supplemental material BMJ Publishing Group Limited (BMJ) disclaims all liability and responsibility arising from any reliance placed on this supplemental material which has been supplied by the author(s) J Immunother Cancer

11.27	11.56
11.74	9.67

Confidential: For Review Only

Garrido-Martin EM, et al. J Immunother Cancer 2020; 8:e000778. doi: 10.1136/jitc-2020-000778

CD20+/field	CD68+/field
4.22	7.56
18.11	9.67
3.56	8.22
3.67	17.83
6.11	20.33
0.22	18.33
8.00	5.11
10.33	11.56
0.33	10.11
2.89	2.44
0.33	7.00
4.44	16.00
0.33	7.89
7.89	9.56
3.33	12.89
1.56	8.00
11.22	14.67
6.22	3.44
0.31	22.44
8.89	7.89
0.11	5.33
1.09	6.78
0.11	3.89
2.22	18.11
50.09	6.67
8.56	3.22
15.09	2.67
2.02	16.11
10.27	9.33
1.09	8.78
6.69	10.33
1.09	7.11
0.47	3.00
2.49	6.11
16.44	30.56
2.02	11.89
Not applicable (no tumour tissue available)	Not applicable (no tumour tissue available)
0.22	8.00
0.47	1.44

Garrido-Martin EM, *et al. J Immunother Cancer* 2020; 8:e000778. doi: 10.1136/jitc-2020-000778

18.82	7.89
7.31	6.00

Confidential: For Review Only

BMJ Publishing Group Limited (BMJ) disclaims all liability and responsibility arising from any reliance placed on this supplemental material which has been supplied by the author(s)

Confidential: For Review Only

BMJ Publishing Group Limited (BMJ) disclaims all liability and responsibility arising from any reliance placed on this supplemental material which has been supplied by the author(s)

Confidential: For Review Only

Garrido-Martin EM, *et al. J Immunother Cancer* 2020; 8:e000778. doi: 10.1136/jitc-2020-000778

Gene
AATF
ADAR
AIM2
ALPK1
ANKRD22
APOBEC3A
APOL2
APOL3
APOL6
ARS2
BATF2
BTN3A1
BTN3A2
BTN3A3
C6ORF106
C9ORF109
CCL15
CCL20
CCL5
CD40
CD69
CFB
CLDN7
CXCL10
CXCL11
CXCL12
CXCL9
DCAF6
DYNLT1
ELF4
ETV7
FAM26F
GBP1
GBP2
GBP4
GBP5
HAPLN3
HCG4
HCP5
HLA-A
HLA-B
HLA-C
HLA-E
HLA-F
HLA-G

Confidential: For Review Only

Garrido-Martin EM, et al. J Immunother Cancer 2020; 8:e000778. doi: 10.1136/jitc-2020-000778

HLA-H
IL1F7
IL11
IL12A
IL12B
IL23A
IL27
IL6
IRF1
IRF5
IRF7
IRF9
ITGAL
JAK2
KCNJ2
LBA1
LOC400759
LRRCC1
MDK
MFAP1
MIA3
MOV10
MR1
NFIX
NLRC5
NMI
NOS2
NUP50
OAT
ORC3L
OSCAR
PARP14
PAXIP1
PLEKHA7
PML
PRPF3
PSMB10
PSMB8
PSMB9
RARRES3
RBCK1
SELT
SERPING1
SERPINI1
SLAMF7
SLC15A4

Confidential: For Review Only

Garrido-Martin EM, *et al. J Immunother Cancer* 2020; 8:e000778. doi: 10.1136/jitc-2020-000778

SLC6A12
SP100
SP140
STAT1
STAT2
TAP1
TAP2
TIFA
TLR2
TLR4
TM9SF2
TM9SF3
TMEM140
TNF
TNFSF10
TRAFD1
TUT1
UBD
UBE2L6
USP9X
VAMP5
VPS36
WARS
ZDHHC8
ZNF668

Confidential: For Review Only

Gene
ADAM15
ADAM28
ADORA3
ALDH5A1
ALOX15
ALOX5AP
ARG1
ARG2
ARHGAP23
ATG7
AUH
BAP1
BATF3
BCL3
C3AR1
C5AR1
CABIN1
CARD9
CBR3
CCDC6
CCL17
CCL18
CCL22
CCL26
CCRN4L
CD14
CD163
CD206
CD209
CLIC2
CRH
CTNNAL1
CXORF40A
CYBB
DAAM1
DAP
DHRS11
DLST
DUSP6
EGLN3
ESPNL
EXOC2
FOS
FOXD2
FOXQ1

Confidential: For Review Only

GALNT12
GALNTL4
GLS
GOLGA8B
GPD1L
GPR65
HARS2
HEMK1
HOMER2
HPS1
IL10
IL10RA
IL10RB
IL13RA1
IL17RB
IL1R1
IL1R2
IL4I1
IL4R
INTS3
IRF4
ISYNA1
ITPRIPL1
KIAA0182
KIAA1671
KTN1
LOC284998
LOC152195
LYZ
MACF1
MAOA
MAP1A
MLKL
MMP1
MMP12
MMP7
MMP9
MUTED
NAGPA
NLRP12
NMD3
PALLD
PELP1
PITPNA
PITRM1
PPP1R14A

Confidential: For Review Only

PPP1R3B
RAB33A
RAB40B
RAMP1
RAP1GAP
RASL10A
RASSF7
RPL28
RRP1B
RTKN
S100A8
S100A9
SNX8
SOCS1
SOCS3
SOX8
STAB1
STAT6
SUCNR1
SULF2
SYT17
TGFBI
TIGD5
TOR3A
TRIB2
TTC9C
UST
WDR33
WNT5A
WNT5B
ZNF317

Confidential: For Review Only

Supplemental material
BMJ Publishing Group Limited (BMJ) disclaims all liability and responsibility arising from any reliance placed on this supplemental material which has been supplied by the author(s)

Gene	baseMean	log2FoldChange	ratio	Fold change
CXCL12	19.51263125	2.489846196	5.61718063	5.61718063
ANKRD22	89.56258349	2.339358547	5.060775747	5.060775747
CXCL10	94.59583781	1.699363322	3.247576076	3.247576076
FAM26F	51.48535892	1.682373483	3.209555443	3.209555443
CXCL9	1390.332009	1.648872185	3.135883982	3.135883982
HAPLN3	44.68826456	1.615332581	3.063822208	3.063822208
AIM2	26.90522505	1.554165757	2.936638655	2.936638655
SLAMF7	461.3068025	1.38261185	2.607399868	2.607399868
TLR2	395.533988	1.35500951	2.557988024	2.557988024
KCNJ2	12.55091287	1.244461962	2.369301771	2.369301771
UBD	97.05301695	1.22187405	2.332495099	2.332495099
CXCL11	17.3031083	1.051506062	2.072692449	2.072692449
STAT1	690.9780365	0.958123103	1.942780759	1.942780759
GBP5	342.8407346	0.865903391	1.822480516	1.822480516
CCL5	147.3033654	0.812839618	1.75666564	1.75666564
USP9X	136.1824523	0.632556208	1.550309444	1.550309444
ZDHHC8	49.34648962	-0.679447059	0.624404544	-1.601525821
TNF	88.91998842	-0.698377192	0.616265019	-1.622678505
SLC6A12	88.50785058	-0.753287535	0.59325015	-1.685629578
ITGAL	819.1592004	-0.81644516	0.567839392	-1.761061339
MDK	58.41483068	-0.829116259	0.562873932	-1.776596753
OSCAR	212.3750855	-1.122001946	0.459455822	-2.176487819
CLDN7	58.18743931	-1.472364424	0.360391172	-2.774762751
SERPING1	4703.840371	-1.498495967	0.353922168	-2.82547998

For peer review Only

Garrido-Martin EM, *et al. J Immunother Cancer* 2020; 8:e000778. doi: 10.1136/jitc-2020-000778

lfcMLE	lfcSE	stat	pvalue	padj	log2ratio T/NL 62
2.901494743	0.296175957	8.406645224	4.22E-17	1.83E-15	1.498517216
2.521986278	0.274028265	8.536924266	1.38E-17	6.37E-16	0.082478085
1.641380525	0.304950028	5.572596045	2.51E-08	3.01E-07	0.109074652
1.837833442	0.288532028	5.830803238	5.52E-09	7.44E-08	2.023512456
1.495946795	0.336453948	4.900736621	9.55E-07	8.59E-06	-0.73818597
1.817391956	0.283403287	5.699766572	1.20E-08	1.53E-07	-2.567594602
1.588036669	0.269751323	5.761475934	8.34E-09	1.09E-07	1.841827458
1.525892975	0.25543981	5.412671783	6.21E-08	6.95E-07	-0.821860336
1.461509408	0.189804527	7.138973627	9.40E-13	2.19E-11	-0.774879989
1.403105198	0.31473418	3.954009582	7.69E-05	0.000445744	4.617959542
1.240676942	0.327186268	3.734490628	0.000188095	0.000974095	-1.605627653
1.229738134	0.301579614	3.486661609	0.00048909	0.002269192	-0.125161109
0.954904739	0.162169937	5.90814253	3.46E-09	4.83E-08	0.611857286
0.856237976	0.232384272	3.726170383	0.000194411	0.00100319	-0.633561931
0.780106681	0.202750545	4.009062556	6.10E-05	0.000362276	1.054847315
0.638259206	0.136030266	4.650113725	3.32E-06	2.67E-05	1.180567474
-0.713113129	0.154939258	-4.385247907	1.16E-05	8.31E-05	0.346555168
-0.709637996	0.233024618	-2.997010347	0.002726415	0.009873199	-1.166254622
-0.763307071	0.137331534	-5.48517529	4.13E-08	4.75E-07	1.321306951
-0.822980645	0.098012229	-8.33003357	8.08E-17	3.35E-15	-0.059834351
-0.935813471	0.18490372	-4.484043147	7.32E-06	5.50E-05	-1.8207842
-1.142368102	0.118330612	-9.48192463	2.50E-21	1.79E-19	-0.542185885
-1.53772971	0.16682362	-8.825875028	1.09E-18	5.56E-17	-2.325796213
-1.537185473	0.17014526	-8.807156684	1.28E-18	6.50E-17	-2.086953916

review Only

log2ratio T/NL 68	log2ratio T/NL 70	log2ratio T/NL 80	log2ratio T/NL 88	log2ratio T/NL 91	log2ratio T/NL 96
4.180379503	3.052158268	3.190213663	-1.412673177	0.491503945	4.633912016
3.023966014	3.279303025	-2.104609117	0.918686364	2.678197607	5.521474877
1.293243319	3.497366863	-0.254576864	-1.508233198	1.241715773	6.350127209
2.164481768	2.397433731	-1.11436417	-0.921961851	1.539744353	5.098396792
3.152369964	3.146610705	-1.283389601	-2.378176651	-1.194211857	5.587865945
2.283843167	3.554908549	-0.433869754	1.641796891	4.738033289	6.882452149
2.666621566	2.626070517	0.048102224	0.180315072	1.957006943	5.644564705
2.999141029	2.928085151	-0.873981238	-1.749159576	0.304108499	4.388176833
1.534448137	2.159500667	3.736684578	2.763950282	1.198666176	2.893215014
3.109450042	2.924705292	-2.8241056	0.212830158	0.7732887	3.578221627
4.205284291	2.537001995	-2.538054294	-2.254768743	-0.190010314	6.536945649
1.323617504	3.203789037	-0.794693188	-5.001351498	1.15374806	4.975947949
2.741898437	2.003305522	0.749378226	-0.370047319	-0.042836991	3.102567688
2.427371774	2.864533245	-1.092331494	-0.516604903	-0.167866669	4.548800285
1.592685512	2.937694967	1.168417462	0.716334928	1.404191176	3.351751672
0.578818113	1.440696009	0.119276497	0.267273903	1.456072708	1.799342692
0.187549396	-1.265583577	-3.211637068	1.078226134	-0.337278241	-2.034235652
-0.277189708	-2.951810767	-1.926197201	1.321649095	2.146123835	1.574421018
-0.259173232	-1.442478111	-3.330430429	-1.178816932	-0.340110033	-1.59430356
-0.361534439	-1.183438209	-1.522937571	-0.971476041	-0.417646399	-1.500823545
-0.456705083	-1.103938846	-1.948547895	-0.60723727	-0.599081623	-1.367107886
-1.419252255	-1.547720692	-2.025577018	-1.460669577	-1.644652761	-1.987533249
-2.564372706	-1.752441747	-0.470562065	-0.774883008	-1.755328922	-1.315271983
-0.161135434	-1.559504559	-2.312254604	-1.8766309	-2.881863195	-0.275880196

For Peer Review Only

Garrido-Martin EM, *et al. J Immunother Cancer* 2020; 8:e000778. doi: 10.1136/jitc-2020-000778

Supplemental material BMJ Publishing Group Limited (BMJ) disclaims all liability and responsibility arising from any reliance placed on this supplemental material which has been supplied by the author(s) J Immunother Cancer

log2ratio T/NL 99	log2ratio T/NL 103	log2ratio T/NL 105	log2ratio T/NL 113	log2ratio T/NL 117	log2ratio T/NL 121
3.18110689	3.940611452	2.632088572	3.031313587	3.288342116	2.098736602
0.654874614	1.781982361	2.430624226	3.498825578	3.871921785	1.970742497
1.388800259	2.734718032	-0.535936501	4.180626836	4.126327059	2.443129745
-0.638240796	2.269321505	1.421136548	3.574255175	3.299203854	1.970742497
4.676983173	1.116969713	-0.240379096	4.872749916	4.979384084	1.784735677
2.883908431	1.027394996	-0.201855932	2.505346734	2.578045117	3.984264923
-0.388530756	0.323823737	0.74247951	1.523516745	2.535489375	2.199065277
-0.111704697	1.486412678	-0.009483558	3.394272814	3.083441138	2.300446966
2.10186886	2.401259743	1.771173324	-0.095187496	0.367035749	3.647281381
-1.120899283	1.739714253	0.614758455	-0.695245061	2.520497867	-1.756952466
0.743719486	2.350934436	0.906253603	3.174345327	3.007465166	-1.12601728
1.319192334	0.836088519	-0.262195309	1.669135205	3.732637994	1.996019686
0.583010007	1.034877639	-0.935942398	2.0891398	2.107601189	1.146471528
-0.101639405	1.013065002	-0.125681653	1.647888011	2.092766469	1.986586502
1.955877517	-1.390794615	-0.677212562	2.371408134	3.051069896	-0.351061562
0.691573886	-0.116036576	0.458669805	1.170838639	0.689719071	2.580841841
-1.397249008	-1.021330352	-0.305419039	-1.095119226	-2.273241476	-2.098473726
-2.28369926	-0.795470527	-2.64739504	1.345983989	-2.02215487	-6.424497091
0.093241337	-1.039470412	-1.414971364	-0.86388149	0.043212166	-0.814713494
-0.741933616	-0.683318087	-0.47798044	-0.519615805	-0.111803532	-2.59973928
-2.44924169	-0.531251448	0.653193286	-0.929574246	0.360163973	-1.445694765
-1.289739052	-0.428269832	-0.993524315	-2.471073923	-0.339554364	-0.476054114
-2.635602886	-2.507041244	-1.771538671	-1.135638781	-0.636658036	-2.994346809
-2.53860485	-0.736943581	-2.243003516	-1.875311135	-0.440202235	-3.019557281

For Peer Review Only

Garrido-Martin EM, et al. J Immunother Cancer 2020; 8:e000778. doi: 10.1136/jitc-2020-000778

Supplemental material BMJ Publishing Group Limited (BMJ) disclaims all liability and responsibility arising from any reliance placed on this supplemental material which has been supplied by the author(s) J Immunother Cancer

log2ratio T/NL 132	log2ratio T/NL 135	log2ratio T/NL 137	log2ratio T/NL 149	log2ratio T/NL 150	log2ratio T/NL 153
1.537750248	-0.814132749	-0.293180431	3.904785506	4.33878523	-0.243957306
-1.658315208	0.433274011	4.453891232	3.692163358	3.622504817	2.864321486
-2.11752607	-0.377848043	0.332281554	0.309689159	3.272471572	-0.522018636
-2.393829277	-0.873812315	3.685152237	1.778240665	2.261901055	-0.766738541
-3.779532397	-2.073527974	1.856020498	0.211397612	4.804213335	-0.398412357
0.226620016	0.962011418	2.062828592	2.097677083	2.575292242	-0.691371153
-0.930110172	1.209174297	3.509632286	0.215290544	0.620418303	-1.372213749
-1.449947141	0.134905144	2.379568209	1.862918812	2.132977955	-0.470466049
-0.881752426	1.896425328	2.344176973	1.258519048	1.411321543	1.627016847
-1.222802806	-0.410703526	1.547638905	3.320380293	-0.019332422	-0.577931839
-3.445540808	-1.241585163	2.113926652	1.27342922	4.954516118	0.181192476
-1.697056596	-2.75459999	1.738781968	-0.486405099	3.197017002	1.333730975
-0.766367972	0.316830025	1.112366402	1.48527666	1.379480368	0.077611795
-1.72583312	0.698856873	2.750818057	0.427122424	1.993574019	-0.051275571
0.517682432	-0.585160665	-0.796488424	1.438841223	1.934319772	-0.577568819
0.683604221	0.697762406	0.045383808	-1.114232209	1.094212823	0.189391173
-1.022629884	-1.740375382	-0.972866547	0.602825911	0.23272373	-0.369757265
1.357582527	-0.161466352	-2.036688216	0.765630849	0.89036996	-0.082286602
-1.451475251	-1.313612463	-0.010174431	-1.210507121	-0.336148512	-0.494644509
-0.622478642	-0.792339618	-1.773275728	-0.472647697	-0.840398333	-0.084741644
0.014339203	-1.848179393	-3.170631737	-2.412997014	-0.783207416	-0.769843928
-0.858335118	-0.879599729	-0.847716878	-1.011577263	-1.200066806	-0.090824303
0.092720733	-0.282991775	-1.431807104	-1.8455067	-2.414924578	0.265712635
-0.404249669	-2.213249172	-2.33517576	-1.448769486	-0.400171764	-1.87353667

Review Only

Garrido-Martin EM, *et al. J Immunother Cancer* 2020; 8:e000778. doi: 10.1136/jitc-2020-000778

BMJ Publishing Group Limited (BMJ) disclaims all liability and responsibility arising from any reliance placed on this supplemental material which has been supplied by the author(s)

log2ratio T/NL 156	log2ratio T/NL 159	log2ratio T/NL 161	log2ratio T/NL 163	log2ratio T/NL 164	log2ratio T/NL 167
1.993884279	2.948946631	5.179086507	3.639482967	4.809560556	1.096439596
1.071038377	4.840898568	2.56631144	1.127160123	4.340313086	3.768886406
1.556878596	0.948648638	2.101066837	1.401967689	2.123110961	2.660224024
0.601222122	0.444717052	2.192819854	2.201433842	1.488578801	5.496957571
2.605826892	0.727588827	2.235451604	-0.364182361	2.513981702	1.908204085
2.014600099	1.384797688	1.644307352	0.927698776	1.13025234	1.019770348
3.129115048	1.579943016	5.347481515	0.51578063	3.644655545	1.894202045
1.21651988	0.721094306	2.284893597	2.204149948	2.762061225	3.026160186
0.891793482	3.565755433	2.126103637	0.749452049	2.545207263	3.307733591
2.503025336	3.694875349	4.21561643	2.906035847	2.498380519	2.427270576
2.176881029	-0.551164199	1.810450453	0.29324162	2.027321835	1.166753745
0.856684261	-0.565674732	1.178589074	-0.122325649	2.212206312	1.214714867
0.928671509	0.303410777	1.786387437	1.27020632	1.671998028	-0.039836733
1.419607859	-0.092512518	2.165803636	0.190147066	1.236401302	0.005252288
0.699578088	1.022088317	-0.591587547	1.113252271	1.788511096	1.636518483
1.089701081	0.239931858	1.928513209	0.547996511	-0.37312651	2.069184463
-0.053807491	-0.55840396	-0.129416052	0.066284066	-1.348343963	-2.074479249
-0.567515727	-0.473347405	-0.117859422	-0.74686428	-2.450846245	-0.589551137
-0.255212251	-1.360903479	-1.744495298	-0.492635962	-1.342757226	-1.951286881
-0.526821296	-0.041525304	-0.982046552	-1.133320254	-0.887645937	-1.447527087
0.947824302	-0.023171814	-1.971260998	-1.567788587	-1.503683349	-2.506524442
-0.713683846	-0.289153428	-2.619233356	-0.830694206	-1.697400456	-2.17047849
0.257179192	-1.497678899	-3.927330231	-2.277398632	-2.36387654	-1.782207494
-0.160164737	-2.43058195	-2.969978521	-1.697322265	-1.474630423	-4.69878326

review Only

log2ratio T/NL 179	log2ratio T/NL 186	log2ratio T/NL 192	log2ratio T/NL 196	log2ratio T/NL 203	log2ratio T/NL 210
2.094948875	2.69427697	1.289984071	4.525863708	4.602038638	-0.248928008
2.848150301	3.237437498	5.457822148	3.136503171	-0.300867536	-0.277604131
2.520899388	3.474153542	3.07968319	7.674819847	-0.993253085	-2.140666405
2.93224584	2.102607505	5.268711722	5.598121933	-1.102175624	-2.37222149
0.97811757	4.549470149	2.748105429	6.735066114	-1.071163229	-4.080247764
1.313870647	2.183315815	3.435363768	4.956564396	-1.911739637	-3.147279773
1.121726179	1.211330314	2.191002735	2.427921498	-0.343941358	-2.08173621
1.821645173	2.194426071	2.562506474	4.829876885	-1.172801546	-0.845127388
1.810492765	0.457761547	1.263604081	-0.646473315	0.145427482	-0.499790965
1.317197174	2.860569014	-0.100133336	0.475376921	0.136783035	-1.777863952
0.91872695	3.991764139	2.631087296	3.701181804	-1.418366531	-3.830092551
1.051249266	1.001681295	1.737522846	5.28774686	-0.886955246	0.124544554
-0.02618618	1.361331053	2.209169582	2.321143054	-0.267206596	-0.887045217
0.140912981	1.600040165	1.884486724	3.421758431	-1.477617664	-2.232299231
-0.837268536	0.134606492	1.537421396	-0.268457115	-0.856282887	-1.513665126
1.057225779	-0.497824942	-0.350345406	0.999670325	0.250143405	0.393559889
-1.400206456	-0.130497255	-0.439973096	-0.041550458	0.767820196	-0.776597084
-0.729705229	-0.473445195	1.719543192	-1.036204478	-2.678320024	-1.501126552
-1.184126907	-0.513370431	0.291827841	-0.965038305	-0.182141017	0.080560628
-1.024375974	-0.435668308	-0.30699092	-0.367283017	-1.198278001	-1.712814904
-2.162501117	-0.125033834	1.850089292	0.324810528	-1.932301333	-0.977653689
-1.913388172	-0.565461266	-0.841799799	-1.191500694	-0.75296904	-0.050471677
-2.837320565	-0.600682197	-0.821723216	-2.417818206	-0.532566748	-0.419820359
-2.274273825	0.955687447	-0.998577348	-0.439236358	-1.681732316	-1.59445756

review Only

Garrido-Martin EM, *et al. J Immunother Cancer* 2020; 8:e000778. doi: 10.1136/jitc-2020-000778

log2ratio T/NL 227	log2ratio T/NL 228
1.489468067	1.380133043
4.392277522	0.777596653
5.42579036	-0.550641319
4.535095243	0.729456026
5.916476361	-0.054485125
4.278405485	0.729456026
4.020950409	-1.305192397
4.396855182	1.238225941
1.077930085	-0.292757447
2.896915246	-1.631670657
6.551306815	0.61803738
4.700751196	0.66014023
2.158067246	0.235757887
2.681210936	-0.395663057
3.202977256	-0.53609824
-0.613251507	0.201408639
-0.845985941	-0.810364221
-1.057834976	-1.160495688
0.312040649	-0.544554968
-0.5889423	-0.956566626
0.632130793	-0.643048529
-2.329659651	-0.552508693
-1.762924935	-1.573234995
0.717642003	-1.286545191

Confidential: For Review Only

Gene	baseMean	log2FoldChange	ratio	Fold change	lfcMLE
MMP12	619.4816667	4.510936887	22.7996044	22.7996044	4.662037728
EGLN3	10.84181384	3.687850001	12.8870487	12.8870487	4.622150154
WNT5A	14.4120669	3.197318715	9.172523623	9.172523623	3.755334326
ADAM28	136.2307988	2.969442729	7.832336393	7.832336393	3.136442248
MMP9	709.8776221	2.953047801	7.743832788	7.743832788	2.993709956
ADORA3	161.7510606	2.703517572	6.51388194	6.51388194	2.862060832
MMP1	14.1233665	2.689277682	6.449903974	6.449903974	3.360448353
STAB1	628.1641159	2.449766404	5.463276361	5.463276361	2.631599763
MMP7	25.6851934	2.326990482	5.017575715	5.017575715	2.605286593
IL4I1	359.5301932	2.322887979	5.003327812	5.003327812	2.41483146
CD209	311.8945887	2.158789819	4.465401252	4.465401252	2.251471622
SULF2	344.44726	1.919140064	3.781975622	3.781975622	2.197874071
IL10	51.87247215	1.561824027	2.952268686	2.952268686	1.823590983
CD14	1179.964207	1.52456077	2.876991124	2.876991124	1.553227489
IL1R2	102.1495033	1.26968122	2.41108284	2.41108284	1.740245493
SYT17	15.17546521	1.150602432	2.220065792	2.220065792	1.298119304
SOCS3	519.0378287	1.094852168	2.135911939	2.135911939	1.133295804
TRIB2	11.12077508	1.087232046	2.12466008	2.12466008	1.215203011
MACF1	292.0586868	1.018158424	2.025332007	2.025332007	1.029434103
PALLD	96.53308326	0.974606148	1.965104655	1.965104655	1.02385414
PPP1R3B	56.65298822	0.927971433	1.902598879	1.902598879	0.956969119
IRF4	28.26617219	0.815489935	1.759895706	1.759895706	0.963225204
SUCNR1	17.43026275	0.680254985	1.602422946	1.602422946	0.763829076
FOS	1988.67157	0.643018625	1.561593145	1.561593145	0.640655023
CBR3	12.89416567	0.626843209	1.544182436	1.544182436	0.678039042
CLIC2	34.90845215	0.619590668	1.53643919	1.53643919	0.620570363
TGFBI	4425.620552	0.605233176	1.521224602	1.521224602	0.608319752
C3AR1	833.534316	0.590410088	1.505674677	1.505674677	0.6071536
NAGPA	99.71325713	-0.586114283	0.666134643	-1.501198009	-0.590733157
HOMER2	10.43916248	-0.924570724	0.526837256	-1.898119369	-0.938995656
ALOX5AP	6363.505702	-0.958198311	0.514699287	-1.942882039	-0.985957657
BATF3	37.67526449	-1.70571994	0.306568221	-3.261916701	-1.799684005
GALNT12	86.49749034	-2.121089279	0.229873286	-4.350222757	-2.337890527

lfcSE	stat	pvalue	padj	log2ratio T/NL 62	log2ratio T/NL 68
0.295104678	15.28588744	9.50E-53	6.76E-49	1.817187227	1.783688961
0.418330602	8.815635255	1.19E-18	6.07E-17	0	5.167067628
0.309528848	10.32963079	5.18E-25	6.30E-23	0.736560532	2.728622178
0.22546803	13.1701276	1.30E-39	1.55E-36	1.98502803	4.063150679
0.237700893	12.423377	1.95E-35	1.26E-32	2.922277896	2.032341516
0.187725447	14.40144429	5.07E-47	1.33E-43	2.39009469	2.376071274
0.532324294	5.051953695	4.37E-07	4.17E-06	1.886258801	0
0.185025386	13.24016373	5.14E-40	6.66E-37	2.242718598	2.172778652
0.33333039	6.981033087	2.93E-12	6.42E-11	0.715252882	1.016532561
0.212891343	10.91114341	1.02E-27	1.81E-25	0.374408901	3.229792802
0.174391163	12.37900924	3.40E-35	1.79E-32	2.108097718	1.015693113
0.258385902	7.427417848	1.11E-13	2.91E-12	0.980724741	3.878728223
0.219384281	7.119124576	1.09E-12	2.51E-11	-0.530828499	2.007514439
0.122792284	12.41577016	2.15E-35	1.33E-32	1.458875224	1.102467278
0.2988357	4.248760174	2.15E-05	0.000144308	-0.740959125	2.079122647
0.238101824	4.832396538	1.35E-06	1.17E-05	1.043796456	2.574362951
0.162323464	6.744879289	1.53E-11	3.06E-10	0.140705875	0.980453038
0.270598875	4.017873486	5.87E-05	0.00034948	1.385512441	0.6418619
0.144482036	7.04695513	1.83E-12	4.08E-11	1.467776584	1.624033054
0.170073157	5.730511298	1.00E-08	1.29E-07	0.859277085	1.475351943
0.15863395	5.84976564	4.92E-09	6.71E-08	-0.000528049	0.141834606
0.232150079	3.512770443	0.00044346	0.002088797	-0.058957917	0.925970171
0.215021514	3.163660103	0.001557986	0.006145199	-0.83314972	0.769281056
0.113421109	5.669302924	1.43E-08	1.80E-07	-0.084447353	0.603405724
0.250605883	2.501310823	0.012373453	0.035542787	1.060812968	0.942402457
0.154834283	4.001637468	6.29E-05	0.000372537	0.105284313	0.437906093
0.109789629	5.512662561	3.53E-08	4.11E-07	-0.188014491	-0.472151355
0.128873679	4.58130855	4.62E-06	3.62E-05	-0.284519786	0.766952851
0.134812432	-4.347627856	1.38E-05	9.71E-05	-0.081826418	-0.723233283
0.327379318	-2.824157397	0.00474051	0.015874911	-3.379398055	-0.848895894
0.137828037	-6.952129158	3.60E-12	7.83E-11	-1.316289228	-2.475170632
0.181611806	-9.392120356	5.88E-21	4.03E-19	-1.834325986	-0.907990377
0.2013818	-10.53267611	6.11E-26	8.05E-24	-0.549380797	-1.609833423

log2ratio T/NL 70	log2ratio T/NL 80	log2ratio T/NL 88	log2ratio T/NL 91	log2ratio T/NL 96	log2ratio T/NL 99
4.785718709	2.709253308	4.064215243	5.226393583	5.731673076	4.944561121
4.512800716	3.253575361	-1.024555882	0.783759824	5.607494731	1.22959072
2.469055949	2.762345338	5.111625244	5.115457718	3.698873713	2.331995687
3.820277676	-0.366402304	3.954645627	4.729691981	4.492884466	2.689686502
4.648469766	3.196701291	1.276103192	1.320827324	4.115711613	2.381964457
2.506275543	3.548120489	0.230502898	3.792400338	3.129132328	2.039048759
3.879901248	0	1.498055983	0.783759824	3.72255626	0
3.963406524	3.313370533	1.254577151	2.189919646	3.132957468	3.032971269
4.54379068	0.023909176	3.234862669	1.255332716	-1.603328744	0.260497024
3.937969862	0.777798487	-0.052666245	1.502235439	5.294246251	2.181776531
2.998921942	0.542795114	0.089068953	2.266145427	2.481909581	3.161737908
4.20845089	3.532663105	1.095625122	1.15215594	4.015413934	1.996091486
3.323717003	4.061940084	1.417873196	0.939100997	1.805696746	0.857976025
2.162353743	1.886461586	0.621635216	1.478749728	1.712469981	1.570741604
5.43450251	3.942521337	3.087061465	-3.923220816	3.691232699	3.518461181
2.207136502	0.526803425	-0.16869468	4.159314054	1.26090301	3.012306905
1.824070258	3.593512139	0.144649835	0.260724732	2.161638041	1.68063464
1.829163047	-2.475425107	-0.290088975	2.630192777	1.801119315	2.331995687
1.574899299	-0.270352763	1.018631275	1.18057327	1.891037355	1.799441935
1.813671391	1.73525014	0.467579321	-0.081634104	-0.622062685	2.04728428
0.975507755	1.437827179	0.684115429	-0.339876156	1.563334241	3.242506482
1.671807392	2.678477603	1.563153668	0.489405824	0.560157533	1.472885122
1.385328363	-2.263362818	-1.544646356	0.920752603	0.949555941	3.416968562
0.438463598	1.357530991	0.213778676	0.974903072	0.507978966	0.90482163
-0.219965332	-4.018311128	0.978558764	0.527892682	1.117537689	0.665158682
0.499907401	-2.104609117	0.934502335	0.420664055	2.081286366	1.451608578
0.694289924	1.834989352	0.331765307	0.077062666	0.883783626	0.321633489
0.935883503	-0.525020542	-0.863134469	1.622339872	1.612504208	0.589413635
-0.946864092	-4.218941355	-2.37450984	-0.689561453	-0.62479234	-0.94243047
3.056305566	-3.948988807	0	1.243452872	-2.709285673	-4.772639836
-1.876940233	0.164617526	0.456709608	-0.45929313	-1.267438791	-2.273914992
-2.133200819	-2.724364777	-4.835432945	-1.86685459	-0.061549559	-2.865853588
-3.057902475	-4.866519907	-3.975795457	-1.844528735	-3.688095567	-2.616279015

Garrido-Martin EM, *et al. J Immunother Cancer* 2020; 8:e000778. doi: 10.1136/jitc-2020-000778

log2ratio T/NL 103	log2ratio T/NL 105	log2ratio T/NL 113	log2ratio T/NL 117	log2ratio T/NL 121	log2ratio T/NL 132
4.03570892	4.898322621	7.675582185	3.651405888	4.473920558	1.617057436
3.193832082	3.926735545	1.719980042	0	4.251687275	3.4704772
1.784552062	4.033575276	4.234710366	2.520497867	2.078315822	1.89559662
2.539331887	4.845181323	2.100730298	2.638265782	2.689247065	-3.140953565
3.81159475	2.695558783	2.477728112	1.987585904	4.698258282	0.962576988
2.651431114	3.472650118	2.660944109	3.506491201	5.450479432	2.715453848
0.859999968	1.751874839	0	-2.855295387	6.383917919	0
3.353113841	4.054846771	3.490653186	1.877226288	2.92766698	2.127207755
1.903461056	1.421136548	1.690792962	1.624694565	6.476698071	1.292586253
3.519581209	2.777194176	3.54685047	1.704189614	4.047079128	1.00458348
2.659998045	2.256316857	4.308352677	2.433739158	1.56820658	2.545970489
3.75090991	1.910176026	0.022598694	0.165967029	2.915318086	1.293622285
2.220570276	1.063697514	2.874401298	1.70730707	4.881527781	-0.292709913
2.674522366	2.18461284	1.52244887	1.193093274	1.771708334	0.682248377
1.839881628	4.554319709	3.039946279	-0.003055755	1.559810422	2.067568298
-0.012477524	2.06412947	0.841312371	0.17475355	2.765884912	3.632093274
0.530925031	1.752471665	2.036840296	0.16982509	1.947434722	0.228898577
-1.827390759	4.22956463	0.229657272	-0.637257545	1.635008663	2.372009996
0.392596586	1.968799575	1.76271433	1.045582381	0.608879451	0.723805273
3.526056691	1.65121893	4.314814543	1.296786656	0.634489832	0.430420935
0.063683766	-0.101381337	0.177599428	0.727650641	2.310801483	1.274282326
-0.012477524	-0.642854927	1.194092009	-0.277783721	-1.071208787	0.340594612
2.090905271	1.312087729	1.970934618	0.026212557	0.30055948	0.013111631
-0.466986064	0.364690574	0.432763212	0.123618762	1.671882409	0.026379633
1.17272256	2.790750989	0.301188919	3.245823363	0	1.127784317
0.651097326	1.803619222	-0.452151407	-0.136753417	1.470117726	-0.586047372
0.848399297	0.451437003	1.070386343	1.387329586	1.605182927	1.580703421
2.481947968	1.359014666	1.314442735	0.693596123	0.645108178	0.469970635
0.645357617	-0.713228506	-1.127056281	0.076132874	-0.805714772	-0.331378414
0.041390345	-0.583528195	-2.321476014	-2.366721618	-1.768178368	1.514595915
-1.237087141	-0.852562006	-1.225629044	-1.448354748	-1.440086979	-0.633715954
-0.75292739	-1.345238309	-0.807810547	-1.56013428	-2.919683254	0.114436974
-3.261519861	-1.370467502	-3.1666424	-3.489976088	-4.341211933	-1.75825696

nly

log2ratio T/NL 135	log2ratio T/NL 137	log2ratio T/NL 149	log2ratio T/NL 150	log2ratio T/NL 153	log2ratio T/NL 156
6.192454263	8.022624568	7.009211227	5.149594554	6.38241489	1.235262987
5.033249463	5.941293909	5.198568212	0.836267051	4.901600538	0.056753498
5.603451022	4.344662749	1.878771893	4.124654707	3.112015319	2.854333865
2.297132549	3.937537847	2.072703594	2.965438209	3.322420678	1.908462314
2.159362304	4.260752337	6.438630442	4.890434232	2.386296559	1.285495873
0.770375274	3.531510833	2.865931365	1.605467618	1.505354757	1.251835178
7.646622399	6.027375011	5.980139975	3.475566892	1.714049886	0
3.009490791	2.724823903	2.824286464	3.540614654	0.032557721	0.754789493
0	5.984976389	4.295539754	2.757111828	0.815795393	-2.44333057
1.389857736	3.152055438	3.302585413	2.854326479	2.007523546	0.918914143
1.824745731	2.413165114	3.316404337	1.055718317	1.271504355	-0.213514831
3.169374537	3.241114872	4.443522095	2.188697042	2.721787683	-0.282268648
3.928600703	0.526303933	2.180365853	1.938282676	0.466059433	-0.966268704
1.668189582	1.686308147	2.739368315	1.308135522	0.72993136	-0.187588396
3.41333792	3.330441913	4.739476009	1.939440793	2.522600075	-0.376433204
0.755150256	2.090566695	0.07890777	0.300680409	1.107156629	-0.100754036
1.449519141	0.324871737	2.302949738	1.488072848	-0.051050185	-0.406887216
-2.26038839	2.383792468	1.68142726	1.379714966	4.208374633	0.868270153
2.453295289	2.031513943	0.213526351	1.1103393	0.6718744	0.30380921
0.279672878	0.718487975	-0.186923271	-0.433228788	0.525844138	0.051567789
1.380434633	1.115523978	2.192440378	0.175882981	1.778325317	0.379848317
3.632884854	-2.108062959	0.29134128	1.867070244	2.349818398	-0.083504169
0.943656233	0.749263385	-4.243471345	0.966140778	2.263437647	2.245620155
1.097234446	0.32990545	0.717548514	0.468803714	-0.374463883	0.289048619
-2.26038839	1.308835951	-0.669728901	-2.617680426	0.108827815	2.872640755
-0.884671839	0.307838382	-1.148461939	1.273398602	0.167128013	1.683246264
1.281115365	2.034081737	1.088542885	-0.081280761	0.50101714	-0.418561497
-0.043268667	0.412839462	-0.056964174	0.96036889	0.775919647	0.701480647
-0.172828465	-1.222216836	0.004945834	-0.246887367	-1.604760661	-0.991780936
0	-2.614075227	-1.877138398	2.599760039	-0.690916547	-0.646390773
-2.244258939	-1.704893685	-1.190946336	-0.602145442	0.294277907	-0.188131011
-2.721182535	-2.132024593	-0.316903767	-0.836319222	-1.432128962	-2.760830349
-1.640980869	-4.025636123	-2.94961654	-0.556084285	1.404852236	-1.59609571

Garrido-Martin EM, *et al. J Immunother Cancer* 2020; 8:e000778. doi: 10.1136/jitc-2020-000778

log2ratio T/NL 159	log2ratio T/NL 161	log2ratio T/NL 163	log2ratio T/NL 164	log2ratio T/NL 167	log2ratio T/NL 179
3.796946456	1.696876854	0.916249745	1.91311282	7.187975812	4.595780381
1.318313697	4.551362039	0	3.214313224	5.594361894	3.861282708
2.840805252	4.653875625	3.669452463	1.425249935	4.381419229	2.66926657
4.777840801	4.814916507	3.173797928	5.423723264	5.002541668	4.617437631
2.875033154	1.475058983	1.532728385	3.253217009	3.027138129	1.465475484
5.905396976	2.815610382	3.0830622	4.763136344	4.811767721	0.59621604
0	-1.964359749	0	-1.061977183	2.305332151	4.412770246
5.495580656	3.333644558	3.666513953	4.534262951	3.276380197	0.912284442
2.454500646	0.905381051	2.557017273	-0.387781637	6.736975138	2.881085863
2.525350296	1.52938469	2.080269014	3.687090298	3.39598135	1.327876651
4.251319371	1.840367857	3.155166238	3.45987761	5.029868182	1.271780473
4.03287436	1.628408323	2.38591714	1.359805979	7.592856634	2.577060685
2.901671401	2.515725225	4.215029376	0.729563172	4.199572374	0.945518748
1.980350363	0.766605063	2.762592509	2.873287924	3.044316402	1.29427046
0.225859445	-0.667797101	2.534445073	2.042424973	1.537397762	2.344591004
2.539704743	2.637865916	0.127826172	1.55260616	1.80202421	-1.05276941
1.399893141	1.565512951	1.965801006	-0.693301914	4.005543887	2.250028876
2.106288778	1.895695806	0.89347278	2.139952702	3.468292436	-0.048193499
0.593385371	3.293911525	2.26993783	0.794430894	1.495559858	1.311354623
1.166295047	1.444033584	2.79539834	0.084424654	1.213246024	2.222365816
1.017571712	2.246964241	0.997112761	0.556467304	2.537675227	1.343359924
3.121759641	2.611107486	2.511445421	2.270739441	2.72940186	1.511278257
0.745726043	2.192819854	0.36868206	0.2474847	1.088692539	1.128119312
1.549986703	1.403774483	1.512213609	-0.683417248	0.148009314	1.713169365
0.78092339	1.88100615	-1.059958704	-0.369796043	0.616026958	-0.470155737
1.084277501	0.56270613	0.100893212	0.903831081	1.284878557	0.845807046
0.618858867	0.225456279	0.136144516	0.655819605	-0.096937486	0.17868029
0.660149722	0.578075154	-0.131182359	2.557420376	1.121750688	0.454322717
0.038556245	-0.626839871	-0.483692244	-0.503749716	-0.781151805	-1.276070237
-0.909448218	-0.829188825	-0.788520411	-1.208482833	-4.616678528	-1.443360749
-0.087383562	-2.327376734	-2.404845974	-1.877796957	-0.401322353	-1.33731722
-1.297820313	-1.95477921	-2.460589797	-2.163335881	-1.663744826	-1.206610568
-1.786322796	-2.909318966	-1.175639276	-2.775358322	-1.758902559	-2.678544201

log2ratio T/NL 186	log2ratio T/NL 192	log2ratio T/NL 196	log2ratio T/NL 203	log2ratio T/NL 210	log2ratio T/NL 227
5.311776681	8.056498042	8.20250256	2.266167712	1.97321563	5.291358491
-0.954402969	4.378665625	4.394609219	4.194860566	-1.143527513	3.911177427
4.600664373	1.1972205	3.059219836	2.030279368	-1.441176584	5.315394864
1.65363083	2.650397923	3.345348889	2.777926575	0.632980987	3.344165726
2.31763851	6.468513936	4.320692647	2.431837295	-0.075552723	4.847025204
1.936664331	1.939228933	3.584685866	3.361472972	4.175465652	2.759314907
2.452485734	1.765648945	6.327678212	2.413717042	0.086876469	-0.241066014
1.271454656	2.507853663	0.922510298	3.100717148	-0.331443666	3.699629246
2.133981815	-0.025290482	3.172064124	4.405955105	1.932359861	1.97307275
2.172847859	3.724006916	3.902449544	0.688157585	-0.141877266	4.819064695
2.466098868	1.914004873	3.658353316	1.705956491	2.741932197	1.206817249
0.131014088	3.191078652	1.12052611	1.245201996	-2.121713911	3.63699559
1.168264556	1.063277848	0.170083393	1.608672921	1.921347073	1.826834997
0.51898657	1.851691747	1.093351405	0.652533534	1.283524038	2.009656049
-0.194067928	1.698846672	-1.639272417	-0.557330576	0.108933955	0.040158814
0.006349451	0.562428871	1.415049138	-1.51235383	0.702096651	0.602603693
0.661028057	1.087985277	0.381586948	-0.366253748	0.596636777	1.109660668
0.195385656	1.050749887	-0.753332604	-0.535475741	0.190739979	1.01263369
-0.673427397	-0.243133734	1.119762289	1.030653735	-0.322841482	-0.469099964
0.870681707	-0.634424747	1.181896548	1.082367855	0.861536739	0.051434473
1.032629998	-0.134873971	-0.852570957	0.407036107	0.499465607	1.845321865
-0.027236072	2.638429448	-0.700801262	1.146709169	-1.6893356	0.071454493
0.267452239	-0.374398602	2.428032524	0.357296895	-0.617490873	0.955102531
2.119135188	0.52240779	1.331212298	-0.066667887	0.655542476	1.301705232
0.513125072	1.167540394	-0.354062062	-0.136981322	3.01504153	2.055436073
0.799641207	0.089900623	0.728395737	-0.158131346	1.242319046	1.558268261
1.082191172	0.30218926	1.034757589	-0.20446765	0.649863948	0.975288264
0.747958287	-0.369341312	0.994466473	-0.649301723	1.036846413	0.019734578
-0.077568474	0.092699199	-0.899435477	0.270781952	0.701941284	-0.28014665
0.005307521	1.063952819	-0.66341619	0.634574707	0.106998667	0.302351849
0.111035448	-0.109864543	-0.813147845	-1.25928243	0.024213281	0.458887539
-1.538499142	0.008174258	-3.818046508	-3.371635897	-2.112258998	-1.56025804
-0.614892543	-3.40445581	-3.367989025	-1.159149875	-1.057682715	-3.373180161

nly

**log2ratio T/NL 228**
5.417098742
0.147124333
0.639676757
2.356903617
5.910828251
2.615814743
4.084894186
1.560439599
4.79817921
1.966208249
1.494516033
-0.648762736
2.503394107
1.160637185
-1.629699117
2.211982456
1.493397045
-0.207028851
0.13658811
1.999227853
0.650291289
-0.343520163
0.340008169
0.072807344
1.49155169
0.789312751
-0.236337068
-0.390771031
0.136795512
0.147124333
-1.016387133
-1.837195891
-1.982777824

Confidential: For Review Only

Table 4: TAM *versus.* N-TAM breakdown per cluster

Cluster ID	TAM Frequency	N-TAM Frequency	Sum	TAM percent	N-TAM Percent
0	279	1586	1865	15%	85%
1	907	563	1470	62%	38%
2	1273	31	1304	98%	2%
3	768	312	1080	71%	29%
4	45	972	1017	4%	96%
5	287	452	739	39%	61%
6	441	163	604	73%	27%
7	182	103	285	64%	36%

Note: Each sample was randomly downsampled to the same number of cells passing qual

Confidential: For Review Only

BMJ Publishing Group Limited (BMJ) disclaims all liability and responsibility arising from any reliance placed on this supplemental material which has been supplied by the author(s)

:

lity control following clustering

Confidential: For Review Only

Garrido-Martin EM, *et al. J Immunother Cancer* 2020; 8:e000778. doi: 10.1136/jitc-2020-000778

Table 4: 'Cluster 3 enriched transcripts'

Differentially expressed genes (MAST < 0.05) with concurrently directionality between T

TAM enriched	N-TAM enriched
CTD-2350C19.2	FABP4
GSDMA	INHBA
MYO10	SERPING1
REPS2	MCEMP1
PTPRN2	DEFB1
PDGFA	C1QA
BIRC7	CXCL3
CYB561A3	SERPINA1
MAOA	C1QB
S100B	RND3
HAAO	STXBP2
SH3RF1	CRIP1
HSD11B1	UBB
EMP2	SCD
ACSM5	ALDH2
FAM27C	MME
LPAR5	AQP3
ZNF703	RETN
TRERF1	TCF7L2
CHST15	CXCL8
RP11-556E13.1	MRC1
ZG16B	ALOX5AP
ST3GAL5	BHLHE41
NRIP3	LGALS3BP
SRGAP3	ARL4A
SLC12A5	RGCC
ST18	FBP1
CADM1	COX17
C1orf21	MIR3945HG
ZNF618	TREM1
LY9	PDLIM1
CCND2	CES1
HRH2	S100A13
RNU11	FN1
SRGAP1	PPARG
TMEM71	HBEGF
AK4	B3GNT5
SYNE3	IGFBP2
RGAG4	LRRFIP1
CRIP2	CEBPB
GNG2	THBS1
ACOT11	SPATS2L
COL4A2-AS2	GPATCH11
STK26	PLA2G16
ALOX15B	CXCL16
SYNE1	RNF149

Confidential: For Review Only

MRC2	LPL
L3MBTL4-AS1	MTPN
LRMP	CARD16
PTPN7	ANXA1
CHST13	RP5-839B4.8
C9orf91	ABCG1
HSPA8	LY6E
TPCN1	ALOX5
ADAMDEC1	SEPT11
AC133644.2	VMO1
FAM101B	CXCL5
DYSF	SPN
PXN	GLIPR2
IGF2BP2	ACO1
MGME1	GLDN
RPLP1	POR
SPHK1	RAB11FIP1
SLAMF7	THBD
GAS6	PPIC
HIST1H1D	SVIL
BATF	CD52
FZD1	TUBA1B
CYP2S1	ALDOA
BCL2	APIP
IER5L	GCA
SLC23A2	TGM2
S100A12	ATP6V1E1
FAM214A	PNPLA6
ETV5	PCOLCE2
STAB1	ENPP4
BMF	QSOX1
OAZ2	RHBDD2
IL10	PFDN2
CHD3	MACC1
TRAF3IP3	TKT
TRAF5	ATP2C1
HAUS1	TAGLN2
FGD6	SPI1
CHKA	ABHD5
PEAK1	MARCO
NME4	LCP1
PKIB	ANKRD28
TXNDC16	HPGD
INPP5D	DDX21
ALKBH7	GYPC
PTGS1	ADAM17
GATM	COMT
FAM13A	PYCARD
MTSS1	SGMS2

Confidential: For Review Only

STARD13	LMNA
SETDB2	S100A4
EVA1B	PRR13
TMEM163	EML4
F13A1	LAMTOR1
ECM1	H2AFZ
PXDC1	EGR2
ALK	CD44
NEK3	FHL1
P2RY11	ADGRE5
ATP8B4	PTPN12
PRDM1	SPINT2
SLC38A7	NMB
POU2F2	LYAR
APOBEC3A	DECR1
RAB3D	CD69
IL1R1	GPD1
SPTBN1	AMIGO2
TBC1D2B	CD55
CCR5	FAM89A
ARHGAP12	MYL12A
GPAT3	PHLDA3
KLHL5	CD151
PTGER2	TXN
CA11	LIMA1
SULF2	ATP1B1
CLEC10A	IFI27
YPEL3	DOCK8
SLC9A9	CPE
MAX	RP11-284N8.3
SLC25A37	OSCAR
ST6GAL1	OLR1
KCNJ5	EVL
CD93	SUN2
CCL13	IL1RN
RFTN1	NSRP1
TFEB	SECTM1
TMEM192	ARRDC4
CLCN7	ACOT7
FAM20A	CDC37
TRMT1	ECH1
TAGAP	HLA-DRB5
TMEM55A	MOB3B
ANO6	FLNA
PTMA	CYB5R3
CD72	ADAMTSL4
OLFML2B	S100A6
ZNF292	SELPLG
SERPINE1	RAC2

Confidential: For Review Only

CUX1	RUNX1
CMKLR1	AGRP
HMHA1	FPR2
KDM7A	CFDP1
RP11-69E11.4	TMEM173
RPS2	PAG1
ANKRD29	MSR1
MXI1	DSTN
IDH2	VSIG4
HPS3	PGD
G0S2	TPT1
RPL10	CCL20
MRPS6	FDFT1
UQCRC2	YWHAH
SLC26A11	UBASH3B
MBNL2	VASP
GPR155	CCND3
MATK	HMGN2
RPL27A	ARPC1B
ADAM8	HIPK2
ADAM28	PTPN6
SDSL	REEP3
AMDHD2	GNB2
RPL13A	UPP1
SPECC1	AKR1C3
GLA	NDUFV2
DNAJC5B	FBXL5
CD300A	IDS
RPS28	RTF1
PDE7A	PLAC8
MREG	ITGB8
RPS12	TCEAL3
SNAP23	PKM
WDR70	TSPAN3
DCK	SAP18
NBPF14	EFHD2
MMP2	ARL6IP1
TRPM2	HLA-DRB1
TMEM37	TSPO
TMEM87B	GGA2
UBAC2	STX11
GALNT7	HEATR5B
DOCK5	CLIC1
RHOC	BSG
ARMCX1	CAT
AP001055.6	KAT8
NUCB2	TRPV2
HTRA4	CDC42EP3
GABARAP	GRN

Confidential: For Review Only

SMPDL3A	ACER3
PFKFB3	RASAL2
CD2AP	PTMS
PAPSS2	IL1RAP
SCARB1	DENND5A
NAF1	MLPH
NBPF19	AGFG1
PLBD2	CETN2
RAD51AP1	RP11-598F7.3
TMIGD3	BLVRA
AMPD3	NDUFB1
ARHGEF40	C19orf43
RP11-160E2.6	DIAPH1
CDS2	CSTA
GOLGA4	MYADM
SATB1	NPTN
ANXA6	OASL
DFNA5	TOMM5
IFNAR2	ACTN4
HELZ	CD74
CYTL1	FCGRT
SRPK2	LAPTM5
PIK3IP1	CLDN7
RPS15A	LSAMP
MMP12	RPL36AL
GAS7	MYO6
ANKH	CYB5A
NLN	NNMT
RPL26	ACADVL
CD180	MYH9
UBE2E1	AES
WDR11	ATF4
ARID3A	COLEC12
LACC1	RTN3
SGTB	CKS1B
RPS13	EPAS1
ISCA2	STAC
GIMAP4	RBP4
HOMER3	FNDC3B
SGPL1	BATF3
DIP2A	ALAS1
CST6	JAML
NREP	ISG15
SNX29	MRPL55
PALLD	RPS20
DNMT1	RARA
NUP62	NDUFS5
STK38L	CD58
CREBRF	OSBPL11

Confidential: For Review Only

Garrido-Martin EM, *et al. J Immunother Cancer* 2020; 8:e000778. doi: 10.1136/jitc-2020-000778

ANTXR2	SLFN11
BLNK	HCAR2
PMFBP1	STX12
PLXND1	MYL12B
KLHL24	TMED5
CAMK1	EIF6
RBP1	SREK1IP1
RAB42	CDC42
MCOLN3	DST
ZNF385A	ERAP2
WIPI1	HP
ELK3	DOK2
LRRK2	SLC7A8
IGFBP7	CDC42BPB
ABCC5	VDAC2
CCL8	IGF1
TXNL1	MSN
MACF1	HMOX2
ABI3	TRGC1
ACBD3	CYSLTR1
LINC01010	TLR4
VCL	TLCD2
RNF145	BRD2
IL6R	RAD23B
ENTPD1	TMEM14A
IFI30	RGS19
MMP9	LAT2
CHD4	DERL1
RGL1	TANC2
CAPZB	PNPLA2
CTTNBP2NL	TNNI2
CDK6	TBC1D9
RPL35	GCC2
MYO5A	NT5C3A
RPS6	SIRPB1
CMTM7	RASGRP2
CISD2	ITIH5
ADORA3	RASSF5
TPST2	CD300LF
ERO1A	PECAM1
TCN2	SLC6A6
TNFRSF21	SPRY1
SLC18B1	ANOS1
ABHD12	DCTN6
LIMD2	PAPSS1
FXYD6	MT-ND2
PLEKHA2	FTH1
TSPAN4	YWHAG
TLE3	ZMAT2

Confidential: For Review Only

Garrido-Martin EM, *et al. J Immunother Cancer* 2020; 8:e000778. doi: 10.1136/jitc-2020-000778

BMJ Publishing Group Limited (BMJ) disclaims all liability and responsibility arising from any reliance placed on this supplemental material which has been supplied by the author(s)

RPS24	TRIQK
WWP1	OPN3
PHF20L1	GPA33
RENBP	ARHGEF28
ATP6V1C1	TMC6
RPS8	CORO2A
CCL7	HMGB1
RSRP1	PER2
PREX1	NCF4
EPB41L2	SLC19A3
HS3ST2	CGGBP1
QPCT	CAPZA2
NGFRAP1	POLD3
GLMP	CD46
GAL3ST4	ATP10A
TIMP3	GAA
DOCK4	MMP24-AS1
AC079767.4	SLC27A3
FAM105A	TXNIP
PPP1CC	MGAT3
PQLC3	DDAH2
DICER1	WDR1
SLC39A11	MYL6
TNFRSF1B	NECAP2
RNASE2	AGPAT2
GADD45G	SELM
JARID2	TMEM74B
EMB	FFAR4
HNRNPU-AS1	ANXA11
VMA21	RAC1
NINJ1	IL1A
RPL41	TMBIM1
LILRB2	RSPO3
LILRB3	ZMPSTE24
RASSF4	LARP4
TCF4	PLBD1
SLC36A1	MYD88
NRP2	DDA1
CLEC5A	BTK
SWAP70	ADGRE3
RPL23A	GALNT12
PHACTR4	MAN1A1
DCAF7	SKIL
RPS27A	APOBR
RPS9	TUBB4B
MTCH2	PROS1
JAK1	NUS1
MYCBP2	PAFAH1B2
SET	CDCP1

Confidential: For Review Only

Garrido-Martin EM, *et al. J Immunother Cancer* 2020; 8:e000778. doi: 10.1136/jitc-2020-000778

Supplemental material
BMJ Publishing Group Limited (BMJ) disclaims all liability and responsibility arising from any reliance placed on this supplemental material which has been supplied by the author(s)

DYNLT1	G3BP2
GIMAP7	MS4A7
NCOA4	KIAA1147
P2RX7	EEF1A1
FCN1	FOLR3
RPL21	PDCD6IP
TDP2	SEPT9
TNS3	USP25
METTL7A	UNC5B
CD86	HIPK1
LPAR6	ADGRE1
MT-ND4	NUP107
UTRN	UBA1
PEA15	FAM134B
ST8SIA4	STMN1
ENO1	SLC3A2
ADAP2	SERTAD1
MIR4435-2HG	TBC1D10C
ATF5	ARF6
ATP6V0A1	ATL1
FMN1	MSMO1
CHPT1	GPRIN3
APP	ITPK1
HGF	TRGC2
SLC4A7	TUBB6
PRDX1	PCMT1
IRF2BP2	MYCBP
AP1B1	RNPEPL1
RCSD1	FKBP1A
FAM20C	STON1
SLC40A1	USP30-AS1
GALM	ACKR3
DHRS9	MSRB1
KIAA0930	SLAIN2
SH3KBP1	GS1-600G8.5
SEP15	CMPK1
ATP6V1B2	HAUS2
PAK1	TMEM128
MMP14	DTX4
IL4I1	B3GNT7
RGS18	RP5-884M6.1
SGK3	USP32
SLAMF8	HP1BP3
NOTCH2	ACVRL1
CYBRD1	ST3GAL2
LILRB5	DYX1C1
LGALS2	CD101
EEF1B2	ZNF800
TMEM50A	PHTF2

Confidential: For Review Only

Supplemental material BMJ Publishing Group Limited (BMJ) disclaims all liability and responsibility arising from any reliance placed on this supplemental material which has been supplied by the author(s)

CREBL2	ARHGAP6
ARL4C	SLC44A2
RNASEH2B	PARP9
FAM49B	GNB1
NAIP	MPZL2
FOLR2	VIM-AS1
CTSS	HMCES
CMTM3	TRHDE
GGCT	OPTN
AP2A2	ICAM2
RPS10	SPOCD1
ITGAM	MMD
RASGRP3	SIK2
TPM4	CSNK1A1
SERINC5	PRSS21
TMEM107	RP6-159A1.4
HAVCR2	FGD5
SNX6	VGLL3
MPP1	SAMD9L
ME2	ITSN1
CTSK	EPB41L4A
CSF3R	TNF
OSBPL8	CCL23
CHST11	ADRBK2
QKI	HK3
MERTK	ZNFX1
FAM198B	MINK1
C1orf54	FAR2
ATP1B3	CLIC4
TNS1	STIM1
CHI3L1	TUBA1A
ZNF331	NCSTN
ATP6V0E1	TXNRD2
RASSF2	PROCR
IFNAR1	CSRP1
NAGK	HCAR3
CHCHD6	RFX2
UCP2	TRPC6
WIPF1	ATP1A1
PRKCB	ADRBK1
CSF1R	APBB2
CPVL	INTS6
RPS17	IKZF2
ZFYVE16	FLOT2
SDS	MT-ND3
MAP3K2	AKAP5
TLR5	KLHL2
SCARB2	KSR1
ATOX1	MSRA

Confidential: For Review Only

Garrido-Martin EM, *et al. J Immunother Cancer* 2020; 8:e000778. doi: 10.1136/jitc-2020-000778

CCL3L3	PDCL
SNCA	ARMC8
RP11-24F11.2	PKIA
RPL10A	EHD1
MGAT4A	TMEM53
SAT1	ECSCR.1
MTHFD2	MORF4L1
EIF4A3	RPS27L
SORL1	FOLR1
IGSF6	RAB11FIP4
HSPE1	AMFR
GNPDA1	IQSEC3
LINC00152	HEXIM1
SLC25A5	COLGALT1
ATP13A3	MFSD10
RPL22L1	TRIM25
PLTP	CEP44
IQGAP2	SAMM50
HM13	ADIPOR2
MT-ATP6	FLVCR2
HSBP1	SFTPC
FCHO2	PEX19
BMP2K	CENPV
ARL5A	GNG12
GPR34	PNP
RALA	KIAA0368
CORO1A	CACNB3
DMXL2	PLEC
PRCP	RARA-AS1
RPL7	MLF1
KDELR2	CATSPER1
DRAM2	MTMR12
FCGR2B	AC011899.9
ABCA1	TMSB4X
FGL2	FAM214B
CREG1	PDGFD
EVI2A	CHD7
TTYH3	MAPK7
CH17-373J23.1	ZDHHC3
CAPG	ATG16L2
RAB32	CTSW
SUB1	PELI3
CD14	MAN2B2
PLEKHO1	TRIM28
DNPH1	GATA3
NAP1L1	F11R
C4orf3	C8B
RP11-386I14.4	PTGER3
SEPP1	FRMD4A

Confidential: For Review Only

Garrido-Martin EM, et al. J Immunother Cancer 2020; 8:e000778. doi: 10.1136/jitc-2020-000778

ATP6AP2	DHCR24
CHMP1B	MDK
FUCA1	PARP10
ADAM9	CARS2
KCNMA1	KLHL29
RNF13	RP11-384F7.2
GM2A	KCNA3
EPSTI1	AKR1C2
PLPP3	ALPK3
SLC38A6	CTD-2135D7.5
HERPUD1	CYBA
PDIA3	BCAR1
TMEM70	RC3H1
RPS3A	TBC1D4
CLEC2B	PELO
SSR4	OR2A1-AS1
NPM1	SIDT2
ITGB2	AKT3
HNRNPU	UTP23
BCAT1	ICOS
CALR	C1orf228
TNFSF13B	NLK
PPT1	CLIP4
LHFPL2	ATN1
RARRES1	KCNQ1
PABPC4	MYB
NEAT1	RAB1B
RPL36A	GPR153
BASP1	PPP2R3A
PRMT9	EPN2
LILRB4	PVR
PLXNC1	AOC3
CD59	HMGCR
SLC1A3	RP13-143G15.4
MALAT1	EPB41L1
NR4A2	CYFIP2
CYP1B1	S100P
OSTC	PGM1
CTSA	OSBPL10
CCR1	ANGPT4
FNIP2	SIGLEC11
NPL	GTPBP2
SOAT1	CD5L
WBP5	AWAT2
CALM3	BPNT1
SEC61G	OVCH1
AP2S1	NEIL2
MAF	ANGPT2
GPX1	ANO5

Confidential: For Review Only

Garrido-Martin EM, *et al. J Immunother Cancer* 2020; 8:e000778. doi: 10.1136/jitc-2020-000778

CECR1	PCED1B
TPP1	ARHGEF35
BLVRB	CHRM3
PSAP	AMOTL1
GNB4	ODF3L1
FOS	ATP6AP1L
CTSZ	SMYD2
ID2	CDC42EP4
FRMD4B	AR
PMP22	RP11-246K15.1
RAP2B	CLCN4
RGS10	KAZN
HLA-DMB	LIMS2
IFI16	ZNF788
SMS	CD3D
LIMS1	CRACR2B
HAMP	ENPP5
LGALS1	FGF10
CD48	IGSF22
MEF2C	A4GALT
GLIPR1	HOXB6
GPR183	ARHGEF5
RNF130	RASSF8-AS1
FOSB	PYGB
PDIA6	ZBTB7C
CD84	
A2M	
HIF1A	
ZEB2	
IFITM3	
MAFB	
PLA2G7	
FYB	
FAM96A	
EMP1	
ASAH1	
PLD3	
RGS2	
CPM	
SDC2	
C15orf48	
MFSD1	
H2AFY	
LY96	
CHIT1	
CD36	
MS4A4A	
OTOA	
CTSB	

Confidential: For Review Only

APOE			
RNASE6			
AP1S2			
NPC2			
CCL18			
CCL3			
FPR3			
ZFP36L1			
RNASE1			
MS4A6A			
TREM2			
GPNMB			
CCL2			
SGK1			
RGS1			
LIPA			
MARCKS			
LGMN			
SPP1			

Confidential: For Review Only

TAM and N-TAM enriched clusters

Confidential: For Review Only

Table 4: Percentage cells expressing given gene in each cluster

	Cluster 0	Cluster 1	Cluster 2	Cluster 3	Cluster 4	Cluster 5
LGMN	3.41	13.02	60.73	35.67	5.80	6.06
SPP1	1.63	8.68	56.53	19.25	0.80	7.17
TMEM74B	5.95	3.43	0.23	0.71	4.91	0.49
FABP4	91.89	64.44	9.55	14.73	95.09	12.73
CXCL9	0.24	1.30	1.22	25.73	1.61	2.72
CXCL10	0.58	1.23	1.83	19.61	3.93	4.08

Note: cluster 7 comprised of cycling cells, consequently was removed from further analy

Confidential: For Review Only

Cluster 6	Cluster 7
9.02	21.14
12.56	24.16
0.16	3.69
6.92	60.74
5.80	0.67
6.12	3.02

Median of remaining clusters (excluding 7)
9.02
8.68
0.49
12.73
2.72
4.08

ysis

Confidential: For Review Only

Garrido-Martin EM, *et al. J Immunother Cancer* 2020; 8:e000778. doi: 10.1136/jitc-2020-000778

Table 4: 'Cluster 3 enriched transcripts'

Gene symbol	log2 fold change versus cluster(0)	log2 fold change versus cluster(1)	log2 fold change versus cluster(2)	log2 fold change versus cluster(4)
HSPA1A	4.08	3.78	4.51	2.91
B2M	0.62	0.56	0.61	0.40
SLC40A1	2.18	2.12	1.63	2.20
HSPA1B	5.08	4.77	5.18	4.19
SOD2	5.68	4.72	4.55	4.41
HLA-A	0.80	1.64	0.76	0.69
SEPP1	2.17	2.13	1.13	2.15
ABCA1	2.54	2.21	1.59	2.33
SDC3	1.23	1.12	1.07	1.15
NFKBIA	3.15	3.18	3.99	1.54
HSP90AA1	0.73	1.18	1.26	1.16
TMEM176A	2.69	2.65	2.63	2.59
TSC22D1	1.90	2.27	2.33	1.56
EGR1	2.38	2.06	2.11	1.47
TMEM176B	3.70	3.53	3.49	3.52
CXCL9	2.60	2.54	2.53	2.54
HSPB1	1.22	1.48	1.52	0.65
CYFIP1	1.54	1.52	1.16	1.25
SLC1A3	3.06	3.01	1.87	2.54
SMPDL3A	1.47	1.39	1.21	1.49
STAT1	3.50	3.42	2.78	2.59
FOLR2	1.45	1.43	0.85	1.42
CCL3	4.22	3.49	0.98	3.22
SDCBP	1.08	1.09	0.77	0.49
ATOX1	1.49	1.77	0.80	2.16
SGK1	5.10	3.51	0.76	3.83
MAFB	4.19	3.47	2.18	3.25
HLA-B	0.28	0.47	0.29	0.62
CXCL10	2.14	2.10	2.03	1.89
PDK4	2.13	1.20	1.94	0.58
HSPA5	1.25	1.35	1.16	0.75
KCNMA1	1.87	1.67	0.91	1.95
ATP1B3	1.81	1.60	1.11	1.60
RAB20	0.93	0.96	0.87	0.78
UBE2D1	1.05	1.37	0.92	1.21
CREG1	1.80	1.89	0.96	1.55
GBP1	1.35	1.62	1.87	0.52
TSPAN4	1.05	1.02	0.70	1.09
ARRDC3	1.43	1.31	1.24	0.63
PFKFB3	1.80	1.76	1.51	1.75
VAMP5	3.01	2.42	2.81	2.63

Confidential: For Review Only

HERPUD1	3.56	3.06	2.51	3.38
RGL1	1.04	0.99	0.68	1.05
CTSL	1.08	1.75	0.83	0.88
PABPC4	2.17	1.77	0.93	1.48
JUNB	1.79	1.44	1.48	1.71
CD14	2.26	2.12	1.22	2.40
TYMP	3.22	2.17	2.24	1.96
SNAP23	0.68	0.73	0.45	0.82
DNAJB1	3.41	3.23	3.61	2.84
ARL4C	3.73	3.42	3.14	3.54
APOC2	0.76	0.81	0.76	0.59
BNIP3L	1.13	0.95	0.94	1.12
IER3	1.42	1.47	1.17	1.31
CCL13	0.72	0.67	0.52	0.73
MGAT1	1.90	1.66	1.06	1.21
FNIP2	2.08	1.85	0.81	2.22
ELL2	1.72	1.37	1.28	0.89
SDS	1.38	1.31	0.65	1.38
HSPH1	2.04	2.24	1.97	2.18
ZFAND5	3.25	3.05	3.34	2.13
ICAM1	1.51	1.43	1.37	1.27
NDFIP1	0.78	0.65	0.56	0.91
GADD45B	2.75	2.15	1.92	1.74
UGCG	0.96	1.07	0.65	0.74
CECR1	2.27	1.70	0.85	1.59
ADAMDEC1	1.02	1.02	0.91	1.04
RARRES3	1.49	1.44	1.67	1.38
EPSTI1	1.78	1.87	0.81	1.28
KLF2	2.23	1.46	2.93	0.78
ADM	1.66	1.67	1.63	1.60
IL2RA	0.64	0.66	0.63	0.65
NR1H3	0.62	0.49	0.45	0.68
PTP4A1	0.93	0.84	0.90	0.39
HLA-E	1.71	1.79	1.75	1.20
ENPP2	1.11	1.10	1.08	1.11
ACP2	0.56	0.49	0.37	0.49
TNFAIP3	3.25	3.14	3.41	2.13
CD38	0.61	0.56	0.56	0.61
RASSF3	0.71	0.66	0.39	0.66
CXCR4	4.79	3.94	3.15	3.34
TNFRSF14	0.55	0.54	0.41	0.54
TNFAIP2	1.02	0.88	1.11	1.02
DNAJA4	0.68	0.74	0.73	0.60
IL18BP	0.68	0.70	0.52	0.64
HIF1A	4.22	3.49	2.37	3.22
HLA-DMB	3.11	2.71	1.47	2.87
DMXL2	1.43	1.19	0.55	1.10
LILRB4	1.79	1.61	0.61	1.86
CMKLR1	0.51	0.47	0.30	0.54

Confidential: For Review Only

Supplemental material BMJ Publishing Group Limited (BMJ) disclaims all liability and responsibility arising from any reliance placed on this supplemental material which has been supplied by the author(s) J Immunother Cancer

TGIF1	0.67	0.80	0.87	0.48
ATF3	2.54	1.94	2.32	1.77
FYB	3.47	2.64	1.40	3.04
PNRC1	3.21	2.79	3.23	1.62
ATF5	0.82	0.74	0.34	0.86
CD4	1.08	0.99	0.71	0.53
NRP2	0.92	0.69	0.48	0.89
NINJ1	1.07	1.01	0.63	1.06
CTSS	0.79	0.39	0.17	0.53
CLK1	1.88	1.45	1.69	1.34
CH25H	1.10	1.07	1.08	0.74
MMP14	0.69	0.60	0.14	0.62
ATP2A2	0.71	0.71	0.58	0.46
CXCL12	0.15	0.15	0.14	0.15
GBP5	0.75	0.73	0.82	0.60
KCTD12	1.46	1.36	0.51	0.75
GBP4	0.81	0.91	1.14	0.54
GLA	0.45	0.52	0.22	0.58
FMNL2	0.43	0.42	0.49	0.41
CLEC4E	1.15	1.05	1.16	1.29
HLA-F	0.47	0.71	0.97	0.47
PIK3R1	0.82	0.65	0.45	0.65
IFNGR1	0.90	0.93	0.53	0.17
A2M	2.10	1.72	0.23	2.14
LCP2	1.43	1.36	1.00	1.32
DNAJB4	1.39	1.37	1.30	1.40
FAM21A	0.47	0.45	0.40	0.43
EIF4A3	1.20	1.18	0.41	1.27
IL32	1.10	1.10	1.08	1.08
MS4A6A	5.71	3.46	2.01	4.83
SLAMF7	0.79	0.78	0.68	0.82
CCL4L2	1.51	1.46	1.05	1.28
NPL	1.74	1.59	0.46	1.59
TNFSF13B	2.33	1.70	1.28	2.00
HSD11B1	0.29	0.26	0.23	0.31
CXCL11	0.67	0.65	0.66	0.63
RASSF4	1.20	1.15	0.77	1.24
CYB561A3	0.25	0.32	0.20	0.33
IQGAP2	1.31	1.18	0.49	1.35
SLC16A10	0.68	0.57	0.37	0.16
RB1	1.09	0.72	0.72	0.98
PAPSS2	0.63	0.56	0.33	0.54
PLIN2	2.71	3.31	2.44	0.45
TPP1	1.69	1.42	0.33	1.59
IGSF6	1.84	2.36	0.93	3.14
TFDP2	0.27	0.34	0.27	0.33
GCH1	0.43	0.32	0.42	0.36
PMP22	1.80	1.44	0.24	1.47
RHOQ	0.83	0.76	0.41	0.58

Confidential: For Review Only

Garrido-Martin EM, *et al. J Immunother Cancer* 2020; 8:e000778. doi: 10.1136/jitc-2020-000778

TBC1D2B	0.32	0.28	0.15	0.32
TFEC	0.37	0.57	0.28	0.53
HNRNPU	1.72	1.10	0.58	0.94
ME1	0.32	0.31	0.35	0.22
DUSP1	2.84	1.85	2.25	0.57
MARCKS	5.21	4.60	0.22	5.10
HNRNPU-AS1	0.73	0.64	0.27	0.72
TRAM1	0.94	0.64	0.29	0.83
IRF2BP2	1.16	1.20	0.63	1.15

Confidential: For Review Only

log2 fold change versus cluster(5)	log2 fold change versus cluster(6)	Adjusted p value(0)	Adjusted p value(1)	Adjusted p value(2)	Adjusted p value(4)
4.17	3.24	1.52E-141	1.30E-121	1.84E-137	1.02E-72
0.69	0.88	1.18E-127	4.26E-88	3.57E-94	1.19E-42
1.93	1.75	2.96E-119	2.79E-82	1.14E-32	1.23E-76
4.58	3.13	8.62E-228	3.54E-169	6.96E-186	9.57E-107
2.89	5.28	9.90E-235	1.82E-147	1.09E-133	1.26E-111
1.53	1.94	6.81E-22	8.43E-60	7.79E-20	3.19E-27
2.11	2.11	2.29E-107	1.23E-80	2.16E-25	6.31E-67
1.91	2.24	3.56E-104	3.26E-56	3.90E-25	2.12E-52
1.12	1.14	3.76E-51	1.10E-30	1.61E-24	5.28E-26
1.57	2.23	2.84E-78	1.79E-66	2.59E-102	4.15E-24
2.25	0.96	1.93E-23	7.92E-39	2.53E-36	1.85E-32
1.68	1.68	3.19E-153	1.58E-112	3.38E-100	3.43E-78
1.65	1.92	5.74E-48	2.98E-66	2.57E-65	4.99E-22
2.12	2.15	1.94E-70	9.70E-47	5.44E-41	9.61E-22
2.00	2.24	9.22E-217	3.12E-140	5.18E-123	2.34E-107
2.13	1.84	1.50E-134	5.39E-95	1.97E-85	7.55E-74
3.79	2.46	7.29E-30	9.47E-31	7.43E-39	1.69E-18
1.36	1.63	7.17E-33	7.54E-31	3.76E-17	1.59E-18
1.70	2.40	5.31E-129	9.10E-102	1.65E-34	4.16E-53
1.09	1.28	4.04E-78	6.59E-48	3.24E-28	1.80E-48
2.49	2.12	6.50E-108	2.10E-89	2.10E-58	1.09E-48
1.21	1.44	1.53E-65	1.54E-49	2.46E-13	2.59E-39
3.34	3.09	2.35E-128	3.45E-74	2.15E-12	6.71E-45
1.43	3.61	2.99E-18	6.52E-17	3.21E-14	2.47E-12
2.26	2.11	1.36E-24	4.96E-35	4.54E-11	1.42E-44
1.27	1.04	4.88E-198	1.14E-84	8.58E-11	5.28E-95
1.66	3.53	3.34E-158	3.91E-89	3.06E-39	1.09E-67
0.34	1.11	1.32E-09	5.21E-19	6.41E-09	3.53E-28
1.49	1.35	2.14E-91	3.55E-68	1.51E-53	1.58E-34
0.99	2.31	3.25E-58	5.10E-18	5.05E-39	9.30E-09
1.13	1.15	1.19E-12	2.93E-14	2.71E-09	1.21E-08
1.50	2.01	3.28E-48	4.99E-31	1.87E-08	1.56E-37
0.44	1.64	1.56E-25	5.43E-19	9.37E-12	4.62E-22
0.71	0.92	5.16E-21	1.47E-21	6.61E-15	2.90E-09
0.96	1.10	1.17E-11	4.94E-21	5.14E-08	2.63E-13
1.83	1.99	1.26E-35	1.70E-34	5.42E-08	4.15E-20
1.01	0.98	2.26E-29	1.58E-35	8.44E-41	6.17E-08
0.79	0.86	7.24E-31	2.78E-22	9.42E-08	1.18E-21
0.57	1.00	5.08E-41	4.30E-30	1.43E-22	3.88E-06
0.74	1.23	2.30E-78	3.56E-60	1.44E-33	7.89E-48
1.03	1.85	6.93E-107	9.55E-52	6.10E-72	1.98E-48

Confidential: For Review Only

1.31	1.25	1.52E-118	4.05E-77	2.00E-51	5.40E-77
0.64	0.86	4.90E-61	9.88E-35	5.11E-11	5.63E-36
3.31	5.07	1.87E-17	4.49E-23	1.87E-07	4.83E-14
0.90	0.95	1.23E-52	1.23E-30	4.71E-08	9.21E-21
0.04	0.99	5.34E-22	4.66E-13	2.18E-15	6.38E-18
1.20	2.29	1.37E-47	1.78E-35	5.16E-12	9.32E-45
0.02	1.40	1.30E-83	9.13E-34	1.27E-36	9.95E-27
0.20	0.28	1.03E-09	2.04E-09	0.0002711	3.09E-10
2.79	1.41	4.46E-122	7.44E-94	1.64E-115	4.02E-59
2.06	1.37	3.13E-199	2.79E-119	8.53E-86	3.72E-108
0.69	0.63	8.32E-19	2.76E-19	1.62E-14	1.31E-06
0.37	1.01	1.16E-15	5.84E-13	2.58E-09	7.82E-12
0.85	0.98	9.61E-36	1.30E-31	5.81E-16	3.33E-21
0.48	0.59	2.21E-38	5.33E-20	5.55E-09	4.73E-22
0.47	1.46	7.87E-35	1.59E-23	3.10E-08	2.45E-11
0.96	0.87	2.04E-64	6.87E-39	4.46E-06	2.04E-53
0.69	1.47	6.25E-51	9.91E-27	5.49E-21	2.95E-08
0.79	0.88	9.04E-78	3.18E-48	6.37E-06	4.82E-48
2.33	1.27	1.16E-45	3.58E-52	4.02E-42	3.05E-44
1.33	3.12	4.10E-94	3.16E-80	5.86E-84	3.70E-35
1.01	0.88	3.31E-51	4.42E-35	1.98E-30	1.04E-19
0.38	0.61	3.06E-07	5.86E-05	0.0096815	1.60E-07
1.16	1.12	1.10E-89	2.20E-42	4.03E-30	6.02E-25
1.01	0.03	1.69E-15	8.11E-17	7.75E-05	1.36E-05
1.19	1.85	1.30E-54	2.02E-24	1.60E-05	8.49E-19
0.88	0.64	2.89E-57	5.96E-44	2.47E-24	9.49E-39
0.92	0.90	3.23E-42	1.48E-31	3.22E-45	2.67E-23
0.90	0.85	3.40E-36	2.96E-35	2.93E-05	2.62E-12
0.45	2.33	4.54E-44	2.60E-17	1.14E-65	3.00E-08
0.69	1.43	2.74E-78	5.41E-64	1.14E-52	2.25E-43
0.56	0.48	2.53E-28	1.31E-26	2.19E-19	3.30E-19
0.53	0.76	1.63E-11	2.01E-05	6.76E-05	1.80E-09
0.33	0.47	1.30E-11	9.18E-09	8.55E-09	0.0474222
0.65	2.26	2.36E-23	2.49E-25	2.47E-22	2.18E-10
0.94	0.65	3.24E-64	1.84E-45	6.69E-39	1.23E-35
0.59	0.59	7.20E-10	1.53E-05	9.74E-05	0.0002744
1.00	1.90	3.29E-114	2.78E-87	7.23E-100	6.89E-34
0.37	0.45	1.09E-26	1.23E-13	1.07E-13	5.35E-14
0.33	0.58	4.01E-11	1.68E-08	0.0129896	1.19E-06
0.59	0.27	3.34E-232	4.65E-109	4.06E-68	7.08E-63
0.29	0.31	1.68E-11	1.05E-07	0.0005777	1.07E-06
0.58	0.94	6.14E-13	2.88E-09	1.53E-13	5.42E-10
0.66	0.52	1.16E-19	5.62E-20	5.54E-18	8.60E-09
0.30	0.40	3.27E-31	4.53E-26	2.13E-08	5.04E-14
1.18	1.18	6.13E-165	1.25E-94	1.79E-41	1.80E-69
2.04	1.18	1.78E-75	9.75E-56	8.97E-16	9.23E-54
0.69	1.76	1.48E-31	4.95E-20	0.0005136	1.60E-12
0.84	1.00	9.02E-81	7.60E-43	0.00057	2.97E-54
0.35	0.44	1.15E-28	1.52E-14	0.0006206	9.97E-21

Confidential: For Review Only

0.41	0.81	2.09E-06	4.59E-08	1.40E-09	0.0126667
1.53	0.60	8.96E-65	4.57E-36	8.40E-44	5.20E-27
0.35	0.83	2.71E-118	5.96E-57	3.93E-17	3.79E-65
0.50	0.92	1.23E-84	2.81E-61	2.70E-70	7.56E-21
0.22	0.24	1.56E-29	4.02E-16	0.0402801	6.49E-23
0.75	0.86	1.35E-16	1.31E-12	4.76E-06	0.0009419
1.00	0.94	2.64E-14	1.66E-06	0.0009505	1.54E-10
0.31	0.65	6.36E-34	2.17E-22	7.01E-08	2.45E-21
0.18	2.61	5.93E-95	1.15E-23	0.001467	3.88E-39
0.68	0.75	1.52E-36	4.93E-19	5.26E-26	7.41E-14
0.44	0.93	3.36E-54	1.55E-37	2.53E-36	4.07E-09
0.63	0.58	2.27E-15	9.91E-12	0.0016146	2.97E-09
0.67	0.88	6.33E-09	2.94E-08	2.15E-06	0.0016833
0.13	0.13	4.54E-08	1.52E-06	8.32E-05	2.29E-05
0.41	0.57	1.55E-23	1.01E-19	1.09E-24	4.95E-08
0.93	1.32	6.42E-20	1.70E-15	0.003003	5.34E-06
0.38	0.36	2.64E-14	3.23E-17	2.41E-31	0.0011845
0.41	0.45	1.44E-05	7.94E-08	0.0031757	1.64E-07
0.60	0.80	0.00351516	0.00122432	0.0001644	0.0035734
0.23	1.08	5.72E-24	7.73E-16	2.42E-19	2.67E-21
0.39	0.75	0.00641622	1.65E-06	6.57E-13	0.0015082
0.39	0.42	1.17E-17	2.96E-10	9.44E-05	1.46E-06
0.19	0.20	5.24E-07	2.71E-07	0.0004527	0.0046501
1.89	1.23	9.61E-94	1.65E-42	0.0046813	7.83E-62
0.50	0.47	1.73E-28	7.51E-21	1.35E-11	3.38E-19
1.25	0.62	3.23E-49	3.81E-40	5.02E-33	2.51E-33
0.33	0.39	3.43E-08	8.89E-06	0.0004242	0.0005279
0.89	0.98	8.10E-23	5.96E-21	0.0057242	1.09E-18
0.85	0.42	2.70E-57	2.20E-45	1.62E-38	5.43E-31
1.77	0.69	2.96E-254	4.42E-81	3.23E-24	1.41E-140
0.59	0.39	4.69E-43	1.33E-29	1.68E-16	1.16E-28
1.20	0.47	6.52E-46	3.29E-36	1.80E-12	8.34E-19
1.36	1.67	1.07E-60	9.07E-42	0.0094879	7.20E-31
0.30	1.00	8.03E-54	1.33E-23	9.26E-13	6.40E-31
0.17	0.24	1.79E-15	6.38E-07	1.33E-05	6.24E-12
0.53	0.39	1.12E-35	4.19E-22	1.27E-21	1.56E-14
0.96	0.43	4.88E-31	5.55E-22	1.38E-07	8.72E-22
0.29	0.22	0.00049551	2.37E-06	0.0118959	1.90E-05
0.83	1.06	4.37E-25	2.58E-17	0.0128949	4.03E-22
0.38	0.49	5.57E-15	2.79E-08	0.0010036	0.0129595
0.25	0.49	1.25E-18	9.92E-08	6.05E-07	9.29E-12
0.32	0.58	2.25E-20	1.17E-10	0.0045176	4.44E-09
1.54	3.84	9.32E-52	1.34E-67	6.33E-37	0.0187405
1.36	1.45	1.10E-21	1.39E-14	0.0241397	1.77E-17
1.17	0.66	2.38E-32	1.62E-41	0.0004375	1.58E-63
0.20	0.08	0.00132815	5.59E-05	0.0052626	3.80E-05
0.05	0.36	7.39E-12	0.00033984	1.40E-08	6.99E-06
1.20	1.20	1.43E-61	3.45E-29	0.0306225	1.74E-22
0.49	0.79	1.62E-10	4.93E-08	0.0333805	1.39E-06

Confidential: For Review Only

0.25	0.28	1.94E-07	0.00089365	0.0346412	8.91E-05
0.61	0.76	0.04240019	9.50E-06	0.0353546	2.08E-05
0.71	0.89	8.01E-24	8.71E-12	0.0359354	1.23E-07
0.31	0.38	1.16E-06	9.22E-07	2.02E-06	0.0363689
0.47	2.97	1.09E-160	1.01E-74	2.28E-95	4.01E-14
2.42	2.75	2.26E-282	1.17E-164	0.0412413	4.49E-173
0.61	0.54	1.05E-15	6.49E-10	0.0465191	1.37E-12
0.64	0.35	4.77E-07	0.00444566	0.0476443	0.0001089
0.42	0.38	1.32E-19	5.71E-19	2.36E-05	8.79E-15

Confidential: For Review Only

Adjusted p value(5)	Adjusted p value(6)	Minimun fold change	mean fold change	maximun fold change	Cluster 0 mean	Cluster 1 mean	Cluster 2 mean	Cluster 3 mean
2.35E-62	2.02E-30	2.91	3.78	4.51	0.48	0.55	0.38	1.58
3.65E-88	1.89E-109	0.40	0.63	0.88	4.93	4.98	4.94	5.35
2.81E-38	1.36E-22	1.63	1.97	2.20	0.00	0.02	0.12	0.51
8.46E-84	3.69E-30	3.13	4.49	5.18	0.14	0.22	0.12	1.44
2.93E-28	4.56E-83	2.89	4.59	5.68	0.52	0.71	0.76	1.91
1.18E-26	4.51E-41	0.69	1.23	1.94	2.38	2.07	2.38	2.67
1.85E-50	2.05E-41	1.13	1.96	2.17	0.00	0.01	0.23	0.57
1.01E-26	4.84E-33	1.59	2.14	2.54	0.06	0.14	0.26	0.59
1.05E-22	2.57E-19	1.07	1.14	1.23	0.02	0.05	0.05	0.27
2.20E-08	8.61E-15	1.54	2.61	3.99	0.65	0.65	0.46	1.33
3.68E-27	8.73E-05	0.73	1.26	2.25	2.17	1.98	1.98	2.43
3.83E-22	9.15E-17	1.68	2.32	2.69	0.01	0.02	0.02	0.55
6.49E-20	1.48E-23	1.56	1.94	2.33	0.18	0.12	0.09	0.57
3.07E-27	2.44E-23	1.47	2.05	2.38	0.16	0.23	0.21	0.71
6.69E-21	4.35E-22	2.00	3.08	3.70	0.01	0.05	0.06	0.79
2.27E-36	1.72E-19	1.84	2.37	2.60	0.01	0.02	0.02	0.63
2.35E-42	5.05E-16	0.65	1.85	3.79	1.41	1.35	1.30	1.74
2.50E-14	3.28E-18	1.16	1.41	1.63	0.19	0.20	0.27	0.49
8.21E-17	1.80E-29	1.70	2.43	3.06	0.07	0.09	0.32	0.72
8.52E-17	2.01E-22	1.09	1.32	1.49	0.01	0.03	0.06	0.30
2.68E-22	1.03E-13	2.12	2.82	3.50	0.39	0.43	0.54	1.14
1.34E-19	9.29E-32	0.85	1.30	1.45	0.01	0.02	0.14	0.33
6.57E-39	1.85E-27	0.98	3.05	4.22	0.30	0.49	1.10	1.36
2.96E-07	6.08E-51	0.49	1.41	3.61	1.66	1.62	1.71	1.90
1.84E-14	3.23E-13	0.80	1.77	2.26	1.07	1.01	1.23	1.36
1.01E-06	0.0039907	0.76	2.58	5.10	0.45	0.79	1.48	1.69
6.03E-10	3.63E-48	1.66	3.05	4.19	0.20	0.36	0.65	1.17
1.24E-07	2.38E-32	0.28	0.52	1.11	2.86	2.71	2.85	2.99
5.17E-16	1.59E-09	1.35	1.83	2.14	0.01	0.03	0.05	0.61
1.49E-05	1.50E-32	0.58	1.53	2.31	0.17	0.38	0.21	0.67
4.18E-06	0.0001219	0.75	1.13	1.35	0.60	0.60	0.62	0.82
7.85E-18	2.74E-31	0.91	1.65	2.01	0.16	0.22	0.37	0.52
2.71E-08	1.41E-08	0.44	1.37	1.81	0.79	0.83	0.93	1.14
3.92E-08	6.63E-10	0.71	0.86	0.96	0.08	0.08	0.10	0.27
2.79E-10	4.56E-06	0.92	1.10	1.37	0.34	0.29	0.37	0.53
7.83E-25	2.10E-24	0.96	1.67	1.99	0.24	0.25	0.44	0.58
3.35E-06	8.59E-05	0.52	1.22	1.87	0.26	0.23	0.16	0.56
4.75E-11	6.07E-10	0.70	0.92	1.09	0.06	0.09	0.15	0.26
1.22E-07	3.49E-07	0.57	1.03	1.43	0.09	0.12	0.13	0.39
1.26E-07	4.90E-11	0.74	1.47	1.80	0.02	0.03	0.08	0.41
1.30E-07	2.33E-14	1.03	2.29	3.01	0.13	0.26	0.18	0.77

Confidential: For Review Only

1.41E-07	1.32E-07	1.25	2.51	3.56	0.24	0.34	0.47	1.04
1.44E-07	1.69E-12	0.64	0.88	1.05	0.00	0.01	0.08	0.21
6.38E-32	2.28E-78	0.83	2.15	5.07	1.34	1.21	1.41	1.57
1.88E-07	0.0005976	0.90	1.37	2.17	0.24	0.33	0.50	0.68
4.13E-07	0.0072161	0.04	1.24	1.79	0.86	0.93	0.90	1.23
4.58E-07	1.50E-20	1.20	1.92	2.40	0.40	0.44	0.62	0.89
4.73E-07	1.67E-05	0.02	1.83	3.22	0.49	0.72	0.71	1.17
4.82E-07	0.0455214	0.20	0.53	0.82	0.14	0.14	0.18	0.26
1.44E-36	8.74E-07	1.41	2.88	3.61	0.16	0.21	0.12	0.96
1.56E-23	1.20E-06	1.37	2.88	3.73	0.02	0.09	0.14	0.84
1.61E-12	1.32E-06	0.59	0.70	0.81	0.07	0.07	0.08	0.21
1.89E-06	2.73E-07	0.37	0.92	1.13	0.27	0.31	0.31	0.48
2.34E-06	3.20E-06	0.85	1.20	1.47	0.11	0.11	0.17	0.42
2.76E-06	1.28E-08	0.48	0.62	0.73	0.00	0.02	0.05	0.16
3.26E-06	2.99E-09	0.47	1.29	1.90	0.30	0.37	0.48	0.67
8.75E-09	0.0006903	0.81	1.47	2.22	0.11	0.17	0.39	0.54
6.02E-06	1.02E-15	0.69	1.24	1.72	0.09	0.17	0.19	0.45
9.46E-09	1.24E-07	0.65	1.07	1.38	0.00	0.02	0.16	0.30
1.62E-29	6.58E-06	1.27	2.01	2.33	0.33	0.32	0.35	0.79
7.22E-06	5.14E-32	1.33	2.70	3.34	0.74	0.80	0.71	1.51
3.63E-11	7.26E-06	0.88	1.25	1.51	0.06	0.08	0.09	0.36
7.40E-06	0.0131432	0.38	0.65	0.91	0.28	0.32	0.34	0.41
2.02E-07	9.78E-06	1.12	1.81	2.75	0.14	0.28	0.33	0.75
1.89E-13	1.31E-05	0.03	0.74	1.07	0.16	0.15	0.23	0.33
1.01E-09	2.03E-18	0.85	1.58	2.27	0.23	0.36	0.53	0.66
6.20E-18	2.02E-05	0.64	0.92	1.04	0.00	0.00	0.03	0.22
8.20E-08	2.22E-05	0.90	1.30	1.67	0.10	0.12	0.06	0.40
5.35E-06	0.000209	0.81	1.25	1.87	0.23	0.23	0.45	0.56
3.82E-05	4.66E-21	0.45	1.70	2.93	0.42	0.60	0.27	0.91
4.63E-05	2.24E-20	0.69	1.45	1.67	0.01	0.01	0.02	0.37
1.47E-09	5.09E-05	0.48	0.60	0.66	0.01	0.00	0.01	0.15
1.38E-06	8.46E-13	0.45	0.59	0.76	0.09	0.13	0.13	0.20
8.17E-05	0.023979	0.33	0.64	0.93	0.22	0.25	0.23	0.39
8.40E-05	1.21E-15	0.65	1.56	2.26	0.85	0.85	0.86	1.17
2.51E-19	9.56E-05	0.65	1.00	1.11	0.00	0.01	0.01	0.23
4.57E-08	3.65E-06	0.37	0.51	0.59	0.08	0.11	0.13	0.19
1.00E-04	1.64E-13	1.00	2.47	3.41	0.16	0.20	0.14	0.86
0.0001023	0.0001292	0.37	0.52	0.61	0.02	0.03	0.03	0.13
0.0001929	0.0032273	0.33	0.55	0.71	0.12	0.14	0.19	0.26
8.49E-11	0.0002298	0.27	2.68	4.79	0.07	0.27	0.42	1.17
0.0003075	0.0253316	0.29	0.44	0.55	0.06	0.08	0.10	0.17
0.0003287	1.41E-05	0.58	0.92	1.11	0.28	0.32	0.26	0.46
6.28E-10	0.0003584	0.52	0.65	0.74	0.03	0.03	0.03	0.18
0.0003918	0.001527	0.30	0.54	0.70	0.01	0.01	0.05	0.15
3.76E-06	0.0004834	1.18	2.61	4.22	0.18	0.33	0.59	1.14
1.31E-14	0.0004916	1.18	2.23	3.11	0.45	0.54	0.81	1.08
4.11E-06	9.55E-29	0.55	1.12	1.76	0.15	0.22	0.35	0.44
1.48E-08	7.88E-08	0.61	1.29	1.86	0.03	0.08	0.29	0.38
7.83E-06	2.91E-07	0.30	0.44	0.54	0.00	0.01	0.05	0.10

Confidential: For Review Only

BMJ Publishing Group Limited (BMJ) disclaims all liability and responsibility arising from any reliance placed on this supplemental material which has been supplied by the author(s)

0.0006918	2.03E-05	0.41	0.67	0.87	0.23	0.22	0.19	0.35
2.33E-10	0.0007375	0.60	1.78	2.54	0.28	0.41	0.33	0.86
0.0007588	0.0032373	0.35	1.96	3.47	0.20	0.37	0.66	0.95
0.0007777	0.0184927	0.50	2.04	3.23	0.45	0.56	0.46	1.15
0.0008916	0.0066309	0.22	0.54	0.86	0.02	0.05	0.13	0.19
1.89E-06	1.52E-05	0.53	0.82	1.08	0.20	0.23	0.28	0.40
2.77E-13	5.07E-08	0.48	0.82	1.00	0.16	0.22	0.25	0.33
0.0012081	0.0002745	0.31	0.79	1.07	0.05	0.07	0.14	0.26
0.012865	1.87E-150	0.17	0.78	2.61	2.89	3.13	3.30	3.38
0.0014685	0.027956	0.68	1.30	1.88	0.29	0.38	0.33	0.68
0.0015547	1.25E-14	0.44	0.89	1.10	0.01	0.02	0.01	0.24
1.80E-08	6.63E-05	0.14	0.54	0.69	0.07	0.10	0.19	0.21
1.79E-06	6.68E-08	0.46	0.67	0.88	0.17	0.18	0.20	0.29
0.0018799	0.0105858	0.13	0.14	0.15	0.00	0.00	0.00	0.03
0.0024161	5.01E-05	0.41	0.65	0.82	0.03	0.03	0.02	0.18
1.33E-06	9.33E-07	0.51	1.06	1.46	0.44	0.46	0.64	0.72
0.0032733	0.0030076	0.36	0.69	1.14	0.11	0.10	0.04	0.26
0.0060662	0.0088693	0.22	0.44	0.58	0.12	0.11	0.17	0.20
3.75E-05	2.09E-07	0.41	0.53	0.80	0.21	0.23	0.20	0.28
0.0036608	8.93E-11	0.23	0.99	1.29	0.12	0.16	0.13	0.35
0.0038776	9.61E-05	0.39	0.63	0.97	0.28	0.24	0.18	0.36
0.0040763	0.0352084	0.39	0.56	0.82	0.08	0.12	0.15	0.24
6.88E-07	0.0275689	0.17	0.49	0.93	0.61	0.62	0.69	0.76
1.13E-39	2.39E-09	0.23	1.55	2.14	0.03	0.11	0.43	0.47
5.41E-09	0.0048293	0.47	1.01	1.43	0.18	0.20	0.27	0.46
6.44E-18	0.0054038	0.62	1.22	1.40	0.04	0.05	0.06	0.36
0.0054329	0.0138206	0.33	0.41	0.47	0.07	0.08	0.08	0.16
6.29E-08	2.37E-06	0.41	0.99	1.27	0.16	0.17	0.33	0.41
1.40E-13	0.0057517	0.42	0.94	1.10	0.00	0.01	0.01	0.24
2.15E-10	0.0061729	0.69	3.08	5.71	0.22	0.70	1.10	1.55
5.04E-10	0.0078843	0.39	0.67	0.82	0.00	0.01	0.03	0.16
2.38E-14	0.0081091	0.47	1.16	1.51	0.07	0.09	0.18	0.42
5.16E-19	3.80E-28	0.46	1.40	1.74	0.06	0.10	0.34	0.41
0.010124	0.001383	0.30	1.44	2.33	0.29	0.43	0.53	0.76
0.0103408	8.05E-05	0.17	0.25	0.31	0.00	0.01	0.02	0.06
9.42E-08	0.0105952	0.39	0.59	0.67	0.00	0.01	0.01	0.16
1.89E-16	0.0118135	0.43	0.96	1.24	0.09	0.12	0.20	0.33
3.75E-05	0.0370407	0.20	0.27	0.33	0.04	0.02	0.05	0.08
2.48E-05	5.19E-07	0.49	1.04	1.35	0.19	0.23	0.37	0.44
0.0115825	0.0029259	0.16	0.44	0.68	0.06	0.08	0.12	0.20
0.0155963	0.0216354	0.25	0.71	1.09	0.18	0.26	0.26	0.41
0.0170457	6.43E-07	0.32	0.49	0.63	0.02	0.04	0.09	0.16
1.37E-13	8.63E-46	0.45	2.38	3.84	0.81	0.71	0.85	1.37
6.37E-07	1.35E-06	0.33	1.31	1.69	0.63	0.70	0.93	0.94
6.90E-05	0.0280085	0.66	1.68	3.14	1.03	0.91	1.27	1.43
0.0003561	0.0291914	0.08	0.25	0.34	0.06	0.05	0.06	0.11
0.0300385	0.0011396	0.05	0.33	0.43	0.02	0.05	0.03	0.11
1.77E-13	1.70E-09	0.24	1.22	1.80	0.07	0.15	0.40	0.44
8.58E-05	7.56E-05	0.41	0.64	0.83	0.20	0.23	0.30	0.35

Confidential: For Review Only

Garrido-Martin EM, et al. J Immunother Cancer 2020; 8:e000778. doi: 10.1136/jitc-2020-000778

0.0077521	0.0061328	0.15	0.27	0.32	0.03	0.04	0.06	0.09
0.0009672	0.0002289	0.28	0.52	0.76	0.30	0.27	0.33	0.36
1.04E-06	0.0029988	0.58	0.99	1.72	0.57	0.70	0.82	0.91
4.24E-07	7.17E-06	0.22	0.32	0.38	0.03	0.04	0.03	0.10
0.0376844	7.07E-71	0.47	1.82	2.97	1.70	1.99	1.87	2.71
2.99E-21	6.27E-26	0.22	3.38	5.21	0.04	0.16	1.20	1.24
7.96E-09	0.0032777	0.27	0.58	0.73	0.07	0.09	0.16	0.22
0.0001431	0.0299568	0.29	0.61	0.94	0.59	0.66	0.72	0.72
0.0092819	0.0490815	0.38	0.82	1.20	0.20	0.20	0.31	0.43

Confidential: For Review Only

Cluste r 4 mean	Cluste r 5 mean	Cluste r 6 mean	Percent cells expressing cluster 0	Percent cells expressing cluster 1	Percent cells expressing cluster 2	Percent cells expressing cluster 3
0.77	0.40	0.67	24.09	29.60	20.70	59.98
5.08	4.85	4.73	100.00	99.94	100.00	100.00
0.00	0.03	0.08	0.05	0.91	6.19	22.01
0.34	0.26	0.60	7.92	12.31	6.95	56.61
0.85	0.97	0.48	26.87	36.14	38.66	75.07
2.45	1.71	1.72	86.76	79.27	87.01	91.04
0.01	0.00	0.00	0.10	0.45	11.46	20.76
0.12	0.12	0.08	3.65	8.10	15.20	29.72
0.04	0.02	0.02	1.10	2.91	3.21	14.37
1.04	0.80	0.72	33.45	34.20	25.36	59.01
2.10	1.38	1.89	83.06	79.02	78.92	83.67
0.04	0.16	0.16	0.34	1.17	1.38	29.02
0.29	0.12	0.10	9.74	7.38	5.42	27.95
0.36	0.17	0.19	8.45	12.82	11.15	31.77
0.07	0.28	0.23	0.38	2.66	3.13	38.69
0.03	0.06	0.15	0.24	1.30	1.22	25.73
1.58	0.56	0.94	63.20	61.66	61.57	67.52
0.27	0.14	0.11	10.60	12.44	16.27	25.82
0.20	0.30	0.16	3.74	5.57	19.17	35.40
0.01	0.04	0.01	0.34	1.68	3.82	16.33
0.62	0.43	0.53	21.69	24.94	29.41	51.55
0.03	0.04	0.00	0.82	1.30	7.94	16.15
0.63	0.34	0.44	13.34	22.22	46.52	50.13
1.88	1.18	0.75	71.74	71.24	74.87	75.95
1.00	0.58	0.66	51.87	51.75	59.36	59.36
0.78	1.17	1.28	23.56	38.99	66.77	68.50
0.42	0.70	0.27	11.08	19.43	34.61	51.73
2.74	2.52	2.20	94.15	90.67	94.73	94.76
0.10	0.12	0.19	0.58	1.23	1.83	19.61
0.54	0.34	0.08	9.12	19.82	11.76	29.64
0.75	0.46	0.46	31.24	32.64	33.16	40.28
0.18	0.13	0.05	9.07	13.28	22.00	27.86
0.88	0.92	0.62	40.50	43.65	48.13	53.06
0.13	0.09	0.06	4.80	5.38	6.26	14.64
0.34	0.26	0.23	18.76	17.88	21.54	28.13
0.34	0.12	0.10	13.87	15.28	25.74	30.97
0.48	0.23	0.26	14.88	14.05	9.55	26.35
0.08	0.05	0.05	3.69	5.31	9.09	15.17
0.28	0.25	0.14	5.04	7.45	7.72	19.79
0.04	0.26	0.13	1.34	1.81	4.58	20.41
0.25	0.46	0.29	7.20	14.05	10.70	36.91

Confidential: For Review Only

0.29	0.70	0.73	13.58	19.82	26.81	48.00
0.00	0.06	0.02	0.00	0.84	4.66	11.45
1.48	0.55	0.26	59.74	55.18	62.34	63.62
0.42	0.43	0.41	14.16	19.24	28.19	34.87
0.92	1.13	0.90	42.13	46.63	45.68	56.61
0.39	0.54	0.29	21.40	23.70	32.24	42.15
0.82	1.00	0.72	26.87	38.08	38.50	54.84
0.13	0.19	0.17	7.68	8.48	11.61	14.64
0.28	0.27	0.59	8.83	12.37	7.49	42.24
0.07	0.35	0.48	1.15	4.73	7.94	39.75
0.13	0.02	0.04	3.79	4.40	4.43	12.16
0.30	0.37	0.23	15.36	19.49	18.41	25.64
0.14	0.23	0.20	6.24	5.83	8.86	20.85
0.01	0.02	0.01	0.05	0.84	2.75	7.63
0.47	0.51	0.30	16.55	21.24	27.04	34.87
0.11	0.29	0.30	6.43	10.04	22.00	27.77
0.29	0.27	0.10	5.18	10.17	11.15	22.89
0.00	0.12	0.09	0.00	0.78	8.25	14.64
0.33	0.20	0.44	18.47	19.11	20.93	37.18
1.05	1.01	0.62	38.29	42.55	37.66	64.33
0.13	0.10	0.13	3.17	4.79	5.58	19.25
0.29	0.26	0.22	16.12	19.37	19.79	22.89
0.37	0.46	0.48	8.01	15.22	17.57	35.67
0.23	0.09	0.32	8.97	9.20	13.60	18.54
0.39	0.33	0.22	12.43	20.60	29.49	34.69
0.00	0.01	0.05	0.00	0.13	1.30	10.83
0.14	0.14	0.16	5.61	7.19	3.82	20.94
0.38	0.26	0.31	12.57	14.12	25.52	29.81
0.77	0.72	0.33	22.12	31.54	15.05	43.03
0.03	0.21	0.04	0.67	0.91	1.22	18.10
0.01	0.01	0.04	0.29	0.13	0.46	7.10
0.10	0.04	0.02	5.23	8.55	8.63	11.89
0.36	0.28	0.24	12.24	15.03	13.60	21.30
1.05	0.87	0.54	43.91	45.60	45.91	55.90
0.01	0.02	0.08	0.00	0.32	0.46	12.07
0.12	0.04	0.03	4.94	6.99	8.40	10.91
0.44	0.53	0.37	9.40	11.79	8.02	40.99
0.02	0.04	0.03	0.82	1.81	1.68	7.72
0.15	0.17	0.11	7.01	8.61	11.54	14.20
0.40	1.03	1.09	3.89	13.54	22.69	51.82
0.09	0.08	0.08	3.89	4.92	6.42	10.03
0.31	0.26	0.22	15.69	18.91	15.58	24.58
0.06	0.02	0.06	2.06	1.62	1.60	9.23
0.03	0.07	0.05	0.58	0.71	2.98	8.34
0.40	0.80	0.78	9.98	18.39	32.01	51.29
0.55	0.48	0.67	24.18	30.38	42.63	51.91
0.25	0.25	0.04	8.97	13.54	20.02	23.34
0.04	0.16	0.14	1.63	4.66	16.73	20.94
0.00	0.02	0.01	0.05	0.71	2.98	5.86

Confidential: For Review Only

0.30	0.21	0.14	12.91	12.82	11.38	19.08
0.46	0.47	0.70	15.21	22.67	17.88	39.93
0.32	0.78	0.66	11.56	20.98	35.37	44.99
0.84	0.90	0.82	24.38	31.41	25.21	53.15
0.02	0.13	0.12	1.44	2.72	7.33	10.47
0.33	0.20	0.19	11.13	13.47	16.35	21.83
0.19	0.06	0.10	8.93	12.82	15.20	18.19
0.06	0.17	0.10	2.64	3.76	8.40	14.11
3.11	3.12	1.80	95.11	96.11	97.25	96.54
0.43	0.46	0.45	15.69	21.18	19.02	34.16
0.10	0.12	0.02	0.34	0.84	0.69	12.07
0.10	0.03	0.05	3.98	6.09	11.31	11.09
0.25	0.12	0.08	9.60	10.88	12.53	16.50
0.00	0.00	0.00	0.00	0.00	0.08	1.69
0.07	0.08	0.04	1.63	2.33	0.92	9.67
0.63	0.44	0.35	23.85	26.04	34.61	36.56
0.19	0.14	0.16	6.14	6.09	2.52	14.55
0.11	0.08	0.07	6.77	6.93	10.77	11.18
0.24	0.08	0.06	11.80	13.99	12.61	15.62
0.12	0.24	0.08	7.15	9.13	7.56	18.90
0.31	0.22	0.17	15.79	14.83	11.00	19.70
0.12	0.13	0.13	4.46	7.25	9.32	13.04
0.81	0.63	0.62	32.39	34.26	37.89	38.69
0.04	0.03	0.15	1.68	6.80	24.68	23.78
0.22	0.33	0.32	10.27	11.72	16.12	24.49
0.04	0.07	0.20	2.40	2.78	3.67	16.86
0.09	0.06	0.05	3.93	4.79	5.19	8.96
0.16	0.19	0.16	9.12	10.23	18.72	21.03
0.01	0.02	0.14	0.14	0.26	0.38	11.80
0.44	0.97	1.25	11.42	35.04	51.34	65.84
0.00	0.01	0.07	0.10	0.45	1.60	8.78
0.14	0.09	0.28	3.31	4.79	8.56	17.92
0.12	0.07	0.03	3.60	6.80	19.63	22.01
0.40	0.58	0.44	15.83	23.77	29.49	37.62
0.00	0.01	0.00	0.10	0.71	1.07	3.55
0.02	0.02	0.05	0.00	0.39	0.23	6.92
0.12	0.09	0.20	5.57	7.71	11.69	18.19
0.02	0.02	0.04	2.16	1.49	3.13	4.88
0.21	0.18	0.16	10.84	13.73	21.01	23.69
0.17	0.11	0.09	3.17	4.79	7.18	10.29
0.21	0.32	0.29	10.27	15.28	15.05	21.12
0.05	0.08	0.03	1.49	2.46	5.19	8.25
1.40	0.86	0.37	39.44	36.53	42.02	59.18
0.72	0.49	0.51	33.25	38.21	48.97	46.32
0.79	0.98	1.12	48.90	44.62	55.69	60.34
0.05	0.06	0.08	3.60	3.24	4.05	6.48
0.04	0.09	0.02	1.34	2.85	1.53	6.03
0.15	0.14	0.14	3.93	9.00	22.46	22.72
0.28	0.20	0.14	11.66	14.44	17.57	19.34

Confidential: For Review Only

0.03	0.02	0.03	1.49	2.33	3.97	5.06
0.30	0.16	0.13	17.08	16.26	19.25	19.52
0.77	0.66	0.63	30.47	38.67	42.78	44.54
0.06	0.01	0.01	1.97	2.72	1.76	5.50
2.50	2.39	1.47	72.70	79.66	77.54	91.75
0.08	0.61	0.51	2.02	8.35	55.92	53.77
0.07	0.10	0.10	3.93	5.25	9.47	11.62
0.68	0.44	0.54	31.72	36.66	39.72	37.18
0.22	0.28	0.30	11.23	12.18	17.95	22.63

Confidential: For Review Only

Supplemental material BMJ Publishing Group Limited (BMJ) disclaims all liability and responsibility arising from any reliance placed on this supplemental material which has been supplied by the author(s) J Immunother Cancer

Percent cells expressing cluster 4	Percent cells expressing cluster 5	Percent cells expressing cluster 6	Mean expression of expressing cells cluster 0	Mean expression of expressing cells cluster 1	Mean expression of expressing cells cluster
39.96	17.18	26.57	1.98	1.85	1.83
100.00	99.88	100.00	4.93	4.98	4.94
0.27	1.61	3.70	1.31	1.68	1.95
18.64	10.63	24.64	1.83	1.76	1.76
43.00	39.80	21.42	1.93	1.98	1.96
92.51	63.91	66.83	2.75	2.61	2.73
0.45	0.25	0.32	1.66	2.16	1.97
7.58	6.06	4.03	1.62	1.69	1.72
2.50	0.87	0.97	1.84	1.60	1.69
53.79	33.99	30.92	1.93	1.91	1.80
84.12	53.15	67.15	2.62	2.51	2.51
2.68	7.66	8.21	1.91	1.76	1.59
16.41	6.18	4.83	1.80	1.56	1.66
19.36	7.79	8.53	1.86	1.78	1.91
4.01	12.98	11.59	1.95	1.92	1.91
1.61	2.72	5.80	2.45	1.86	2.04
71.99	24.35	36.55	2.23	2.18	2.12
16.77	7.17	5.64	1.75	1.61	1.66
11.60	13.60	7.89	1.80	1.61	1.69
0.80	2.35	0.81	1.97	1.60	1.62
34.70	19.41	24.64	1.80	1.73	1.82
1.52	2.22	0.16	1.80	1.75	1.74
26.14	13.35	16.91	2.28	2.21	2.36
82.52	48.45	35.59	2.31	2.28	2.28
53.35	26.21	30.76	2.06	1.95	2.07
40.86	45.36	51.69	1.92	2.04	2.21
23.28	29.54	13.85	1.83	1.85	1.88
94.11	84.55	77.78	3.03	2.99	3.01
3.93	4.08	6.12	2.11	2.07	2.50
28.10	14.59	4.03	1.89	1.92	1.82
41.75	21.01	22.06	1.91	1.83	1.87
11.06	6.67	2.90	1.73	1.63	1.69
47.64	37.82	29.95	1.95	1.91	1.93
7.85	4.57	3.06	1.73	1.52	1.53
20.70	12.36	12.08	1.80	1.63	1.73
20.96	6.30	5.64	1.76	1.64	1.69
27.12	11.00	12.56	1.77	1.62	1.68
5.08	2.72	2.58	1.71	1.60	1.64
15.97	11.25	7.41	1.73	1.60	1.70
2.05	11.25	6.76	1.75	1.79	1.80
13.47	19.90	14.17	1.81	1.84	1.69

Confidential: For Review Only

Garrido-Martin EM, et al. J Immunother Cancer 2020; 8:e000778. doi: 10.1136/jitc-2020-000778

17.22	30.04	31.24	1.78	1.74	1.75
0.27	2.97	1.13	NA	1.77	1.61
67.62	23.73	12.72	2.23	2.20	2.27
24.89	19.53	20.13	1.72	1.70	1.79
47.90	45.98	40.74	2.03	2.00	1.97
22.39	23.86	14.49	1.86	1.84	1.92
44.25	42.27	33.17	1.84	1.88	1.84
7.76	8.90	8.53	1.76	1.60	1.57
15.97	11.74	24.96	1.81	1.68	1.55
3.75	15.33	22.71	1.70	1.84	1.79
8.30	1.24	2.25	1.77	1.51	1.70
17.93	16.81	11.43	1.74	1.61	1.68
8.21	10.51	9.66	1.79	1.82	1.89
0.36	1.36	0.48	1.21	1.96	1.78
26.76	22.99	14.98	1.83	1.72	1.79
6.87	13.10	14.81	1.74	1.71	1.78
16.41	11.99	5.48	1.80	1.64	1.68
0.18	5.32	4.83	NA	2.26	1.89
19.80	9.64	20.61	1.81	1.66	1.67
52.54	42.03	28.99	1.94	1.87	1.88
7.49	4.94	6.92	1.91	1.69	1.62
17.48	12.86	11.43	1.76	1.65	1.72
20.87	20.02	21.10	1.80	1.81	1.85
13.56	4.20	14.33	1.75	1.65	1.69
22.84	15.70	10.63	1.82	1.73	1.79
0.00	0.49	2.90	NA	1.59	1.96
8.74	7.17	8.21	1.69	1.64	1.64
21.68	12.98	15.14	1.84	1.66	1.76
40.86	30.90	15.62	1.90	1.89	1.80
1.96	9.27	2.09	1.99	1.60	1.77
0.36	0.74	1.61	1.92	1.61	1.63
6.51	2.47	0.81	1.70	1.57	1.55
21.14	13.23	12.40	1.80	1.66	1.70
54.50	37.58	26.25	1.94	1.86	1.88
0.54	0.74	4.03	NA	2.15	1.55
7.31	1.98	2.09	1.66	1.60	1.52
24.71	24.23	17.55	1.75	1.72	1.72
1.52	1.85	1.45	1.98	1.63	1.53
9.19	8.03	5.80	1.70	1.60	1.66
20.70	39.93	43.80	1.89	1.99	1.87
5.44	4.08	4.19	1.64	1.64	1.60
18.64	12.61	10.63	1.77	1.68	1.69
3.57	1.24	2.90	1.68	1.64	1.63
1.87	3.21	2.42	1.52	1.50	1.72
23.02	33.87	34.94	1.81	1.80	1.86
31.31	22.74	32.53	1.87	1.78	1.89
14.99	11.62	2.09	1.73	1.61	1.73
2.23	7.79	7.09	1.68	1.69	1.72
0.00	1.11	0.32	2.08	1.92	1.56

Confidential: For Review Only

Garrido-Martin EM, *et al. J Immunother Cancer* 2020; 8:e000778. doi: 10.1136/jitc-2020-000778

BMJ Publishing Group Limited (BMJ) disclaims all liability and responsibility arising from any reliance placed on this supplemental material which has been supplied by the author(s)

17.40	10.26	7.09	1.77	1.69	1.68
25.69	20.64	29.79	1.86	1.81	1.82
18.82	33.87	29.95	1.77	1.78	1.86
44.51	38.57	37.04	1.87	1.78	1.81
1.69	6.06	6.12	1.66	1.80	1.78
19.45	9.64	9.50	1.78	1.69	1.72
11.60	3.58	5.15	1.78	1.69	1.67
3.66	8.03	5.15	1.71	1.75	1.62
98.31	91.84	67.63	3.04	3.26	3.39
24.71	21.26	21.90	1.83	1.79	1.73
5.08	5.44	0.97	1.66	2.09	1.61
5.98	2.10	2.90	1.72	1.56	1.66
14.90	5.69	4.19	1.75	1.65	1.60
0.00	0.00	0.00	NA	NA	2.45
4.01	3.83	2.42	1.80	1.49	1.66
34.97	20.15	18.20	1.83	1.79	1.84
11.15	6.92	7.73	1.80	1.61	1.62
6.69	4.20	4.19	1.70	1.56	1.57
14.99	4.82	3.70	1.76	1.62	1.61
6.78	11.62	4.35	1.75	1.70	1.70
18.82	10.75	8.53	1.78	1.65	1.63
7.40	6.55	6.76	1.72	1.59	1.64
44.87	27.94	28.99	1.87	1.81	1.82
2.50	1.36	8.05	1.77	1.67	1.76
13.47	15.08	15.78	1.73	1.71	1.66
2.68	3.21	9.98	1.75	1.65	1.59
5.35	3.34	3.06	1.67	1.62	1.62
9.90	8.65	8.37	1.75	1.65	1.75
0.89	1.36	5.96	1.80	2.16	1.89
25.33	39.43	50.56	1.90	2.01	2.15
0.18	0.87	3.38	2.06	1.87	1.70
7.23	3.58	10.47	2.02	1.78	2.16
7.23	3.96	1.61	1.77	1.53	1.74
23.28	26.08	21.74	1.81	1.79	1.79
0.09	0.62	0.00	2.04	1.96	1.44
0.80	0.87	2.25	NA	1.93	2.29
7.31	4.08	9.82	1.69	1.60	1.68
1.52	0.87	1.77	1.75	1.64	1.49
13.02	9.64	8.70	1.71	1.65	1.75
9.99	5.07	4.35	1.82	1.73	1.72
12.22	15.08	13.85	1.75	1.70	1.71
3.03	3.83	1.45	1.58	1.73	1.69
64.23	33.50	17.07	2.05	1.94	2.02
39.79	22.99	24.80	1.90	1.84	1.90
41.12	39.80	45.09	2.11	2.03	2.29
3.84	2.72	4.03	1.68	1.54	1.50
2.85	4.20	1.13	1.75	1.72	1.72
8.47	6.92	7.57	1.72	1.66	1.77
17.93	9.52	7.25	1.73	1.61	1.69

Confidential: For Review Only

Garrido-Martin EM, *et al. J Immunother Cancer* 2020; 8:e000778. doi: 10.1136/jitc-2020-000778

2.05	1.24	1.29	1.73	1.66	1.54
18.64	8.41	7.57	1.76	1.65	1.69
42.11	29.05	29.15	1.87	1.81	1.92
3.75	0.74	0.48	1.71	1.44	1.66
92.51	80.47	59.90	2.34	2.50	2.41
4.37	25.96	23.35	1.85	1.90	2.14
4.91	4.20	5.15	1.72	1.67	1.72
37.73	20.64	25.76	1.85	1.81	1.81
13.11	13.47	14.49	1.75	1.66	1.73

Confidential: For Review Only

Garrido-Martin EM, *et al. J Immunother Cancer* 2020; 8:e000778. doi: 10.1136/jitc-2020-000778

Mean expression of expressing cells cluster 3	Mean expression of expressing cells cluster	Mean expression of expressing cells cluster 5	Mean expression of expressing cells cluster
2.64	1.93	2.33	2.52
5.35	5.08	4.86	4.73
2.34	1.44	1.85	2.03
2.55	1.84	2.41	2.44
2.55	1.99	2.44	2.25
2.94	2.65	2.68	2.57
2.77	1.41	1.58	1.45
1.99	1.63	1.93	2.05
1.88	1.75	1.89	1.69
2.26	1.94	2.34	2.34
2.91	2.50	2.59	2.82
1.91	1.65	2.05	1.99
2.05	1.74	1.98	2.08
2.23	1.84	2.15	2.20
2.03	1.77	2.17	1.97
2.46	2.05	2.19	2.59
2.57	2.20	2.28	2.58
1.91	1.63	1.97	1.99
2.03	1.69	2.23	1.98
1.85	1.70	1.88	1.59
2.21	1.80	2.21	2.15
2.03	1.74	1.86	1.23
2.70	2.41	2.57	2.58
2.50	2.27	2.44	2.12
2.28	1.88	2.21	2.16
2.46	1.90	2.57	2.47
2.27	1.80	2.36	1.94
3.16	2.91	2.98	2.82
3.10	2.66	2.97	3.04
2.25	1.92	2.33	2.06
2.04	1.81	2.19	2.06
1.86	1.60	1.93	1.66
2.14	1.84	2.42	2.06
1.82	1.64	1.98	1.89
1.89	1.66	2.13	1.92
1.87	1.63	1.96	1.79
2.11	1.78	2.13	2.04
1.73	1.57	1.84	1.80
1.96	1.74	2.27	1.93
2.02	1.74	2.33	1.94
2.08	1.85	2.31	2.04

Confidential: For Review Only

2.17	1.70	2.32	2.34
1.85	1.86	2.01	1.78
2.47	2.19	2.32	2.01
1.95	1.67	2.20	2.01
2.17	1.92	2.45	2.22
2.11	1.72	2.28	2.00
2.13	1.85	2.37	2.17
1.74	1.61	2.18	1.96
2.28	1.77	2.27	2.36
2.12	1.78	2.31	2.12
1.72	1.57	1.55	1.70
1.88	1.66	2.21	2.03
2.03	1.70	2.20	2.11
2.16	1.75	1.80	1.70
1.91	1.75	2.21	1.98
1.93	1.56	2.21	2.05
1.97	1.74	2.22	1.88
2.04	1.88	2.34	1.93
2.13	1.64	2.04	2.15
2.35	2.00	2.40	2.14
1.86	1.73	1.97	1.93
1.80	1.67	2.06	1.91
2.11	1.78	2.30	2.29
1.80	1.71	2.04	2.20
1.92	1.72	2.13	2.03
2.02	NA	2.05	1.87
1.89	1.63	1.98	1.91
1.88	1.73	2.03	2.07
2.10	1.88	2.32	2.12
2.07	1.62	2.30	1.93
2.07	1.46	1.78	2.22
1.71	1.56	1.59	2.03
1.85	1.70	2.11	1.95
2.10	1.92	2.31	2.04
1.90	1.41	2.21	1.91
1.74	1.64	1.86	1.52
2.11	1.77	2.21	2.09
1.72	1.60	1.93	1.75
1.80	1.62	2.13	1.84
2.25	1.94	2.58	2.49
1.66	1.66	1.92	1.87
1.89	1.69	2.10	2.05
1.95	1.68	1.89	2.05
1.75	1.64	2.12	1.96
2.23	1.76	2.36	2.23
2.08	1.76	2.11	2.07
1.88	1.68	2.15	1.70
1.83	1.71	2.01	1.99
1.76	NA	2.21	2.06

Confidential: For Review Only

Garrido-Martin EM, *et al. J Immunother Cancer* 2020; 8:e000778. doi: 10.1136/jitc-2020-000778

BMJ Publishing Group Limited (BMJ) disclaims all liability and responsibility arising from any reliance placed on this supplemental material which has been supplied by the author(s)

1.82	1.70	2.08	1.95
2.16	1.80	2.26	2.35
2.11	1.71	2.32	2.21
2.15	1.89	2.34	2.21
1.77	1.42	2.10	2.01
1.85	1.72	2.11	2.05
1.80	1.62	1.81	1.97
1.82	1.61	2.10	1.90
3.50	3.16	3.40	2.66
1.98	1.75	2.15	2.05
1.96	1.88	2.19	2.21
1.91	1.63	1.64	1.75
1.78	1.65	2.05	1.92
1.86	NA	NA	NA
1.91	1.71	2.08	1.79
1.98	1.79	2.20	1.93
1.80	1.71	2.01	2.01
1.80	1.59	1.92	1.77
1.77	1.63	1.72	1.75
1.83	1.74	2.07	1.92
1.81	1.64	2.00	1.97
1.86	1.67	2.02	1.96
1.97	1.81	2.24	2.12
1.96	1.59	1.85	1.92
1.88	1.61	2.22	2.06
2.12	1.60	2.03	2.01
1.73	1.66	1.88	1.65
1.95	1.63	2.15	1.89
2.01	1.58	1.79	2.30
2.36	1.75	2.46	2.47
1.86	2.09	1.70	2.09
2.36	1.99	2.43	2.65
1.87	1.63	1.88	1.73
2.01	1.70	2.22	2.01
1.76	1.91	1.47	NA
2.29	2.24	2.43	2.37
1.79	1.59	2.18	2.03
1.73	1.61	1.76	2.00
1.84	1.58	1.91	1.88
1.98	1.71	2.14	2.16
1.92	1.68	2.12	2.08
1.88	1.54	2.04	2.05
2.31	2.19	2.56	2.18
2.03	1.81	2.12	2.04
2.38	1.92	2.46	2.48
1.65	1.42	2.05	1.98
1.80	1.51	2.20	1.84
1.95	1.77	2.07	1.85
1.81	1.58	2.08	1.88

Confidential: For Review Only

1.75	1.57	1.67	2.04
1.83	1.62	1.95	1.78
2.04	1.82	2.27	2.16
1.78	1.67	1.17	1.73
2.95	2.70	2.98	2.46
2.31	1.81	2.35	2.17
1.91	1.52	2.31	1.92
1.94	1.79	2.13	2.09
1.88	1.67	2.08	2.07

Confidential: For Review Only

Garrido-Martin EM, et al. J Immunother Cancer 2020; 8:e000778. doi: 10.1136/jitc-2020-000778

Table 4: Weighted co-expression analysis of 'cluster 3 enriched transcripts' in purif

Edges

Source	Target	weight	Type
HSPA1A	HSPA1B	0.42	undirected
HSPA1A	HSPB1	0.09	undirected
HSPA1A	ARRDC3	0.09	undirected
HSPA1A	PFKFB3	0.05	undirected
HSPA1A	DNAJB1	0.38	undirected
HSPA1A	BNIP3L	0.07	undirected
HSPA1A	HSPH1	0.08	undirected
HSPA1A	GADD45B	0.11	undirected
HSPA1A	ADM	0.21	undirected
HSPA1A	IL2RA	0.05	undirected
HSPA1A	CXCR4	0.06	undirected
HSPA1A	DNAJA4	0.07	undirected
HSPA1A	EIF4A3	0.10	undirected
HSPA1A	PLIN2	0.06	undirected
B2M	HLA-A	0.11	undirected
B2M	STAT1	0.07	undirected
B2M	ATOX1	0.06	undirected
B2M	HLA-B	0.07	undirected
B2M	GBP1	0.05	undirected
B2M	VAMP5	0.09	undirected
B2M	RARRES3	0.06	undirected
B2M	HLA-E	0.06	undirected
B2M	LILRB4	0.06	undirected
B2M	HLA-F	0.07	undirected
B2M	SLAMF7	0.05	undirected
B2M	TNFSF13B	0.06	undirected
SLC40A1	CXCL12	0.05	undirected
HSPA1B	HSPB1	0.10	undirected
HSPA1B	ARRDC3	0.09	undirected
HSPA1B	PFKFB3	0.06	undirected
HSPA1B	DNAJB1	0.42	undirected
HSPA1B	BNIP3L	0.06	undirected
HSPA1B	HSPH1	0.07	undirected
HSPA1B	GADD45B	0.12	undirected
HSPA1B	ADM	0.20	undirected
HSPA1B	DNAJA4	0.06	undirected
HSPA1B	EIF4A3	0.08	undirected
HSPA1B	PLIN2	0.06	undirected
SOD2	CXCL9	0.16	undirected
SOD2	STAT1	0.14	undirected
SOD2	CXCL10	0.10	undirected
SOD2	GBP1	0.16	undirected
SOD2	VAMP5	0.05	undirected
SOD2	CCL13	0.07	undirected
SOD2	ADAMDEC1	0.12	undirected

Noc

Node
HSPA1A
B2M
SLC40A1
HSPA1B
SOD2
HLA-A
SEPP1
SDC3
NFKBIA
HSP90AA1
TMEM176A
TSC22D1
TMEM176B
CXCL9
HSPB1
SLC1A3
SMPDL3A
STAT1
FOLR2
CCL3
SDCBP
ATOX1
SGK1
MAFB
HLA-B
CXCL10
PDK4
HSPA5
ATP1B3
UBE2D1
CREG1
GBP1
TSPAN4
ARRDC3
PFKFB3
VAMP5
HERPUD1
RGL1
JUNB
CD14
TYMP
SNAP23
DNAJB1
ARL4C
BNIP3L

Confidential: For Review Only

SOD2	RARRES3	0.08	undirected
SOD2	TNFAIP3	0.06	undirected
SOD2	CD38	0.06	undirected
SOD2	CH25H	0.08	undirected
SOD2	GBP5	0.18	undirected
SOD2	GBP4	0.13	undirected
SOD2	SLAMF7	0.12	undirected
SOD2	CXCL11	0.07	undirected
SOD2	GCH1	0.09	undirected
HLA-A	SDC3	0.10	undirected
HLA-A	TMEM176A	0.14	undirected
HLA-A	TMEM176B	0.11	undirected
HLA-A	HSPB1	0.06	undirected
HLA-A	STAT1	0.09	undirected
HLA-A	ATOX1	0.15	undirected
HLA-A	MAFB	0.06	undirected
HLA-A	HLA-B	0.21	undirected
HLA-A	TSPAN4	0.11	undirected
HLA-A	VAMP5	0.18	undirected
HLA-A	CD14	0.09	undirected
HLA-A	TYMP	0.10	undirected
HLA-A	MGAT1	0.12	undirected
HLA-A	ZFAND5	0.06	undirected
HLA-A	ICAM1	0.08	undirected
HLA-A	CECR1	0.08	undirected
HLA-A	RARRES3	0.09	undirected
HLA-A	EPSTI1	0.06	undirected
HLA-A	NR1H3	0.11	undirected
HLA-A	HLA-E	0.18	undirected
HLA-A	ACP2	0.14	undirected
HLA-A	CD38	0.08	undirected
HLA-A	TNFAIP2	0.07	undirected
HLA-A	IL18BP	0.12	undirected
HLA-A	HLA-DMB	0.07	undirected
HLA-A	LILRB4	0.16	undirected
HLA-A	CMKLR1	0.05	undirected
HLA-A	ATF5	0.09	undirected
HLA-A	CD4	0.09	undirected
HLA-A	NINJ1	0.08	undirected
HLA-A	MMP14	0.06	undirected
HLA-A	HLA-F	0.19	undirected
HLA-A	PIK3R1	0.09	undirected
HLA-A	IL32	0.08	undirected
HLA-A	SLAMF7	0.06	undirected
HLA-A	TNFSF13B	0.12	undirected
HLA-A	RASSF4	0.12	undirected
HLA-A	SLC16A10	0.05	undirected
HLA-A	TFEC	0.05	undirected
SEPP1	FOLR2	0.08	undirected

IER3
CCL13
MGAT1
FNIP2
ELL2
SDS
HSPH1
ZFAND5
ICAM1
NDFIP1
GADD45B
UGCG
CECR1
ADAMDEC1
RARRES3
EPSTI1
KLF2
ADM
IL2RA
NR1H3
PTP4A1
HLA-E
ENPP2
ACP2
TNFAIP3
CD38
CXCR4
TNFRSF14
TNFAIP2
DNAJA4
IL18BP
HIF1A
HLA-DMB
DMXL2
LILRB4
CMKLR1
TGIF1
ATF3
FYB
PNRC1
ATF5
CD4
NINJ1
CTSS
CLK1
CH25H
MMP14
ATP2A2
CXCL12

Confidential: For Review Only

Garrido-Martin EM, et al. J Immunother Cancer 2020; 8:e000778. doi: 10.1136/jitc-2020-000778

SDC3	NFKBIA	0.05	undirected
SDC3	TMEM176A	0.12	undirected
SDC3	TMEM176B	0.09	undirected
SDC3	CXCL9	0.06	undirected
SDC3	STAT1	0.17	undirected
SDC3	ATOX1	0.08	undirected
SDC3	MAFB	0.05	undirected
SDC3	HLA-B	0.06	undirected
SDC3	GBP1	0.10	undirected
SDC3	VAMP5	0.20	undirected
SDC3	CD14	0.06	undirected
SDC3	TYMP	0.11	undirected
SDC3	MGAT1	0.05	undirected
SDC3	ICAM1	0.10	undirected
SDC3	RARRES3	0.13	undirected
SDC3	EPSTI1	0.06	undirected
SDC3	NR1H3	0.12	undirected
SDC3	HLA-E	0.10	undirected
SDC3	ACP2	0.11	undirected
SDC3	CD38	0.11	undirected
SDC3	TNFAIP2	0.07	undirected
SDC3	IL18BP	0.09	undirected
SDC3	LILRB4	0.14	undirected
SDC3	CMKLR1	0.11	undirected
SDC3	ATF5	0.08	undirected
SDC3	CD4	0.05	undirected
SDC3	NINJ1	0.12	undirected
SDC3	MMP14	0.08	undirected
SDC3	CXCL12	0.06	undirected
SDC3	GBP5	0.08	undirected
SDC3	GBP4	0.07	undirected
SDC3	HLA-F	0.12	undirected
SDC3	IL32	0.12	undirected
SDC3	SLAMF7	0.11	undirected
SDC3	TNFSF13B	0.14	undirected
SDC3	RASSF4	0.14	undirected
NFKBIA	STAT1	0.05	undirected
NFKBIA	PFKFB3	0.06	undirected
NFKBIA	VAMP5	0.10	undirected
NFKBIA	JUNB	0.07	undirected
NFKBIA	TYMP	0.07	undirected
NFKBIA	ICAM1	0.05	undirected
NFKBIA	ACP2	0.06	undirected
NFKBIA	LILRB4	0.11	undirected
NFKBIA	PNRC1	0.09	undirected
NFKBIA	NINJ1	0.10	undirected
NFKBIA	HLA-F	0.07	undirected
NFKBIA	TNFSF13B	0.06	undirected
NFKBIA	DUSP1	0.08	undirected

GBP5
KCTD12
GBP4
GLA
FMNL2
CLEC4E
HLA-F
PIK3R1
A2M
LCP2
DNAJB4
FAM21A
EIF4A3
IL32
MS4A6A
SLAMF7
NPL
TNFSF13B
HSD11B1
CXCL11
RASSF4
IQGAP2
SLC16A10
RB1
PAPSS2
PLIN2
TPP1
GCH1
TFEC
HNRNPU
ME1
DUSP1
HNRNPU-AS1

Confidential: For Review Only

HSP90AA1	SLC1A3	0.10	undirected
HSP90AA1	CCL3	0.05	undirected
HSP90AA1	SDCBP	0.08	undirected
HSP90AA1	ATOX1	0.06	undirected
HSP90AA1	SGK1	0.05	undirected
HSP90AA1	HSPA5	0.15	undirected
HSP90AA1	ATP1B3	0.14	undirected
HSP90AA1	CREG1	0.11	undirected
HSP90AA1	HERPUD1	0.06	undirected
HSP90AA1	RGL1	0.10	undirected
HSP90AA1	JUNB	0.10	undirected
HSP90AA1	MGAT1	0.05	undirected
HSP90AA1	ELL2	0.06	undirected
HSP90AA1	HSPH1	0.15	undirected
HSP90AA1	ZFAND5	0.12	undirected
HSP90AA1	NDFIP1	0.09	undirected
HSP90AA1	UGCG	0.11	undirected
HSP90AA1	PTP4A1	0.17	undirected
HSP90AA1	ENPP2	0.09	undirected
HSP90AA1	TNFRSF14	0.05	undirected
HSP90AA1	HIF1A	0.08	undirected
HSP90AA1	DMXL2	0.16	undirected
HSP90AA1	PNRC1	0.06	undirected
HSP90AA1	CTSS	0.05	undirected
HSP90AA1	CLK1	0.09	undirected
HSP90AA1	ATP2A2	0.13	undirected
HSP90AA1	FMNL2	0.07	undirected
HSP90AA1	DNAJB4	0.12	undirected
HSP90AA1	EIF4A3	0.07	undirected
HSP90AA1	MS4A6A	0.08	undirected
HSP90AA1	NPL	0.06	undirected
HSP90AA1	IQGAP2	0.14	undirected
HSP90AA1	SLC16A10	0.06	undirected
HSP90AA1	RB1	0.16	undirected
HSP90AA1	TPP1	0.10	undirected
HSP90AA1	TFEC	0.07	undirected
HSP90AA1	HNRNPU	0.11	undirected
HSP90AA1	ME1	0.09	undirected
HSP90AA1	HNRNPU-AS1	0.08	undirected
TMEM176A	TMEM176B	0.26	undirected
TMEM176A	STAT1	0.11	undirected
TMEM176A	ATOX1	0.10	undirected
TMEM176A	MAFB	0.05	undirected
TMEM176A	HLA-B	0.12	undirected
TMEM176A	GBP1	0.06	undirected
TMEM176A	TSPAN4	0.07	undirected
TMEM176A	VAMP5	0.18	undirected
TMEM176A	CD14	0.08	undirected
TMEM176A	TYMP	0.10	undirected

Confidential: For Review Only

TMEM176A	MGAT1	0.07	undirected
TMEM176A	ICAM1	0.12	undirected
TMEM176A	CECR1	0.05	undirected
TMEM176A	RARRES3	0.10	undirected
TMEM176A	EPSTI1	0.06	undirected
TMEM176A	NR1H3	0.11	undirected
TMEM176A	HLA-E	0.13	undirected
TMEM176A	ACP2	0.11	undirected
TMEM176A	CD38	0.10	undirected
TMEM176A	TNFAIP2	0.06	undirected
TMEM176A	IL18BP	0.14	undirected
TMEM176A	HLA-DMB	0.09	undirected
TMEM176A	LILRB4	0.12	undirected
TMEM176A	CMKLR1	0.10	undirected
TMEM176A	ATF5	0.09	undirected
TMEM176A	CD4	0.16	undirected
TMEM176A	NINJ1	0.09	undirected
TMEM176A	MMP14	0.08	undirected
TMEM176A	HLA-F	0.14	undirected
TMEM176A	PIK3R1	0.06	undirected
TMEM176A	IL32	0.13	undirected
TMEM176A	SLAMF7	0.08	undirected
TMEM176A	TNFSF13B	0.13	undirected
TMEM176A	RASSF4	0.18	undirected
TSC22D1	ELL2	0.07	undirected
TSC22D1	CTSS	0.06	undirected
TSC22D1	GLA	0.06	undirected
TMEM176B	STAT1	0.11	undirected
TMEM176B	ATOX1	0.06	undirected
TMEM176B	HLA-B	0.07	undirected
TMEM176B	GBP1	0.07	undirected
TMEM176B	VAMP5	0.14	undirected
TMEM176B	CD14	0.05	undirected
TMEM176B	TYMP	0.05	undirected
TMEM176B	ICAM1	0.07	undirected
TMEM176B	RARRES3	0.10	undirected
TMEM176B	NR1H3	0.06	undirected
TMEM176B	HLA-E	0.11	undirected
TMEM176B	ACP2	0.07	undirected
TMEM176B	CD38	0.06	undirected
TMEM176B	IL18BP	0.08	undirected
TMEM176B	HLA-DMB	0.08	undirected
TMEM176B	LILRB4	0.07	undirected
TMEM176B	CMKLR1	0.07	undirected
TMEM176B	CD4	0.12	undirected
TMEM176B	HLA-F	0.11	undirected
TMEM176B	IL32	0.11	undirected
TMEM176B	SLAMF7	0.08	undirected
TMEM176B	TNFSF13B	0.07	undirected

Confidential: For Review Only

Garrido-Martin EM, et al. J Immunother Cancer 2020; 8:e000778. doi: 10.1136/jitc-2020-000778

Supplemental material BMJ Publishing Group Limited (BMJ) disclaims all liability and responsibility arising from any reliance placed on this supplemental material which has been supplied by the author(s) J Immunother Cancer

TMEM176B	RASSF4	0.14	undirected
CXCL9	SMPDL3A	0.09	undirected
CXCL9	STAT1	0.27	undirected
CXCL9	CXCL10	0.32	undirected
CXCL9	GBP1	0.29	undirected
CXCL9	VAMP5	0.07	undirected
CXCL9	CCL13	0.05	undirected
CXCL9	ICAM1	0.05	undirected
CXCL9	ADAMDEC1	0.25	undirected
CXCL9	RARRES3	0.17	undirected
CXCL9	NR1H3	0.06	undirected
CXCL9	TNFAIP3	0.10	undirected
CXCL9	CD38	0.15	undirected
CXCL9	CMKLR1	0.06	undirected
CXCL9	CXCL12	0.11	undirected
CXCL9	GBP5	0.26	undirected
CXCL9	GBP4	0.31	undirected
CXCL9	IL32	0.12	undirected
CXCL9	SLAMF7	0.22	undirected
CXCL9	HSD11B1	0.10	undirected
CXCL9	CXCL11	0.29	undirected
CXCL9	GCH1	0.27	undirected
HSPB1	DNAJB1	0.09	undirected
HSPB1	NR1H3	0.05	undirected
HSPB1	ACP2	0.06	undirected
HSPB1	IL18BP	0.05	undirected
SLC1A3	SDCBP	0.06	undirected
SLC1A3	ATOX1	0.05	undirected
SLC1A3	ATP1B3	0.12	undirected
SLC1A3	CREG1	0.07	undirected
SLC1A3	HERPUD1	0.09	undirected
SLC1A3	JUNB	0.09	undirected
SLC1A3	CD14	0.05	undirected
SLC1A3	MGAT1	0.06	undirected
SLC1A3	ZFAND5	0.08	undirected
SLC1A3	UGCG	0.07	undirected
SLC1A3	PTP4A1	0.11	undirected
SLC1A3	DMXL2	0.12	undirected
SLC1A3	PNRC1	0.08	undirected
SLC1A3	ATP2A2	0.07	undirected
SLC1A3	FMNL2	0.08	undirected
SLC1A3	A2M	0.11	undirected
SLC1A3	DNAJB4	0.06	undirected
SLC1A3	MS4A6A	0.10	undirected
SLC1A3	NPL	0.13	undirected
SLC1A3	IQGAP2	0.11	undirected
SLC1A3	RB1	0.06	undirected
SLC1A3	TPP1	0.09	undirected
SLC1A3	ME1	0.10	undirected

Confidential: For Review Only

Garrido-Martin EM, et al. J Immunother Cancer 2020; 8:e000778. doi: 10.1136/jitc-2020-000778

SMPDL3A	STAT1	0.05	undirected
SMPDL3A	CXCL10	0.07	undirected
SMPDL3A	GBP1	0.06	undirected
SMPDL3A	HSD11B1	0.05	undirected
SMPDL3A	CXCL11	0.10	undirected
STAT1	HLA-B	0.11	undirected
STAT1	CXCL10	0.27	undirected
STAT1	GBP1	0.32	undirected
STAT1	VAMP5	0.17	undirected
STAT1	TYMP	0.12	undirected
STAT1	SNAP23	0.05	undirected
STAT1	CCL13	0.05	undirected
STAT1	ICAM1	0.12	undirected
STAT1	ADAMDEC1	0.20	undirected
STAT1	RARRES3	0.28	undirected
STAT1	EPSTI1	0.10	undirected
STAT1	NR1H3	0.15	undirected
STAT1	HLA-E	0.06	undirected
STAT1	ACP2	0.09	undirected
STAT1	TNFAIP3	0.08	undirected
STAT1	CD38	0.24	undirected
STAT1	TNFAIP2	0.05	undirected
STAT1	IL18BP	0.09	undirected
STAT1	LILRB4	0.10	undirected
STAT1	CMKLR1	0.12	undirected
STAT1	ATF5	0.05	undirected
STAT1	NINJ1	0.11	undirected
STAT1	MMP14	0.08	undirected
STAT1	CXCL12	0.13	undirected
STAT1	GBP5	0.29	undirected
STAT1	GBP4	0.30	undirected
STAT1	HLA-F	0.11	undirected
STAT1	IL32	0.21	undirected
STAT1	SLAMF7	0.30	undirected
STAT1	TNFSF13B	0.15	undirected
STAT1	HSD11B1	0.10	undirected
STAT1	CXCL11	0.22	undirected
STAT1	RASSF4	0.13	undirected
STAT1	GCH1	0.19	undirected
FOLR2	TSPAN4	0.09	undirected
FOLR2	CD14	0.07	undirected
FOLR2	CCL13	0.07	undirected
FOLR2	CLEC4E	0.09	undirected
CCL3	PDK4	0.10	undirected
CCL3	IER3	0.06	undirected
CCL3	PTP4A1	0.07	undirected
CCL3	HLA-E	0.07	undirected
CCL3	DMXL2	0.10	undirected
CCL3	CLK1	0.08	undirected

Confidential: For Review Only

CCL3	KCTD12	0.06	undirected
CCL3	IQGAP2	0.09	undirected
CCL3	SLC16A10	0.05	undirected
SDCBP	ATP1B3	0.16	undirected
SDCBP	UBE2D1	0.06	undirected
SDCBP	ZFAND5	0.06	undirected
SDCBP	NDFIP1	0.06	undirected
SDCBP	UGCG	0.06	undirected
SDCBP	PTP4A1	0.12	undirected
SDCBP	DMXL2	0.07	undirected
SDCBP	CTSS	0.10	undirected
SDCBP	NPL	0.07	undirected
SDCBP	IQGAP2	0.06	undirected
SDCBP	TPP1	0.07	undirected
SDCBP	ME1	0.05	undirected
ATOX1	SGK1	0.08	undirected
ATOX1	MAFB	0.15	undirected
ATOX1	HLA-B	0.07	undirected
ATOX1	ATP1B3	0.06	undirected
ATOX1	CREG1	0.07	undirected
ATOX1	TSPAN4	0.14	undirected
ATOX1	VAMP5	0.14	undirected
ATOX1	HERPUD1	0.07	undirected
ATOX1	JUNB	0.07	undirected
ATOX1	CD14	0.14	undirected
ATOX1	TYMP	0.09	undirected
ATOX1	MGAT1	0.21	undirected
ATOX1	ZFAND5	0.10	undirected
ATOX1	ICAM1	0.05	undirected
ATOX1	RARRES3	0.05	undirected
ATOX1	EPSTI1	0.05	undirected
ATOX1	NR1H3	0.10	undirected
ATOX1	PTP4A1	0.07	undirected
ATOX1	HLA-E	0.16	undirected
ATOX1	ENPP2	0.06	undirected
ATOX1	ACP2	0.14	undirected
ATOX1	CD38	0.05	undirected
ATOX1	TNFRSF14	0.08	undirected
ATOX1	TNFAIP2	0.07	undirected
ATOX1	IL18BP	0.09	undirected
ATOX1	HLA-DMB	0.06	undirected
ATOX1	DMXL2	0.09	undirected
ATOX1	LILRB4	0.15	undirected
ATOX1	ATF3	0.06	undirected
ATOX1	PNRC1	0.07	undirected
ATOX1	ATF5	0.11	undirected
ATOX1	CD4	0.06	undirected
ATOX1	NINJ1	0.09	undirected
ATOX1	CLK1	0.08	undirected

Confidential: For Review Only

ATOX1	ATP2A2	0.10	undirected
ATOX1	HLA-F	0.15	undirected
ATOX1	PIK3R1	0.14	undirected
ATOX1	DNAJB4	0.06	undirected
ATOX1	MS4A6A	0.06	undirected
ATOX1	TNFSF13B	0.12	undirected
ATOX1	RASSF4	0.09	undirected
ATOX1	IQGAP2	0.10	undirected
ATOX1	SLC16A10	0.08	undirected
ATOX1	RB1	0.07	undirected
ATOX1	TFEC	0.14	undirected
ATOX1	HNRNPU	0.11	undirected
ATOX1	HNRNPU-AS1	0.09	undirected
SGK1	MAFB	0.05	undirected
SGK1	ATP1B3	0.05	undirected
SGK1	CREG1	0.09	undirected
SGK1	HERPUD1	0.07	undirected
SGK1	MGAT1	0.07	undirected
SGK1	PTP4A1	0.05	undirected
SGK1	DMXL2	0.06	undirected
SGK1	ATF3	0.16	undirected
SGK1	CLK1	0.08	undirected
SGK1	ATP2A2	0.10	undirected
SGK1	GLA	0.05	undirected
SGK1	PIK3R1	0.05	undirected
SGK1	IQGAP2	0.07	undirected
SGK1	TPP1	0.05	undirected
SGK1	TFEC	0.15	undirected
MAFB	CREG1	0.05	undirected
MAFB	VAMP5	0.11	undirected
MAFB	CD14	0.08	undirected
MAFB	MGAT1	0.15	undirected
MAFB	HLA-E	0.09	undirected
MAFB	ACP2	0.05	undirected
MAFB	DMXL2	0.05	undirected
MAFB	LILRB4	0.12	undirected
MAFB	ATP2A2	0.06	undirected
MAFB	HLA-F	0.08	undirected
MAFB	TNFSF13B	0.10	undirected
MAFB	IQGAP2	0.05	undirected
MAFB	TFEC	0.09	undirected
HLA-B	GBP1	0.08	undirected
HLA-B	VAMP5	0.13	undirected
HLA-B	TYMP	0.06	undirected
HLA-B	MGAT1	0.05	undirected
HLA-B	ICAM1	0.05	undirected
HLA-B	RARRES3	0.09	undirected
HLA-B	NR1H3	0.06	undirected
HLA-B	HLA-E	0.12	undirected

Confidential: For Review Only

HLA-B	ACP2	0.08	undirected
HLA-B	CD38	0.06	undirected
HLA-B	IL18BP	0.07	undirected
HLA-B	LILRB4	0.09	undirected
HLA-B	GBP5	0.05	undirected
HLA-B	HLA-F	0.13	undirected
HLA-B	IL32	0.07	undirected
HLA-B	SLAMF7	0.07	undirected
HLA-B	TNFSF13B	0.07	undirected
HLA-B	RASSF4	0.06	undirected
CXCL10	GBP1	0.31	undirected
CXCL10	VAMP5	0.07	undirected
CXCL10	ADAMDEC1	0.16	undirected
CXCL10	RARRES3	0.17	undirected
CXCL10	TNFAIP3	0.05	undirected
CXCL10	CD38	0.11	undirected
CXCL10	CXCL12	0.08	undirected
CXCL10	GBP5	0.24	undirected
CXCL10	GBP4	0.24	undirected
CXCL10	IL32	0.10	undirected
CXCL10	SLAMF7	0.22	undirected
CXCL10	HSD11B1	0.07	undirected
CXCL10	CXCL11	0.24	undirected
CXCL10	GCH1	0.24	undirected
PDK4	CD14	0.07	undirected
PDK4	ZFAND5	0.05	undirected
PDK4	ICAM1	0.05	undirected
PDK4	HLA-E	0.05	undirected
PDK4	DMXL2	0.07	undirected
PDK4	IQGAP2	0.05	undirected
HSPA5	ATP1B3	0.09	undirected
HSPA5	CREG1	0.06	undirected
HSPA5	JUNB	0.06	undirected
HSPA5	HSPH1	0.07	undirected
HSPA5	ZFAND5	0.07	undirected
HSPA5	UGCG	0.07	undirected
HSPA5	PTP4A1	0.15	undirected
HSPA5	HIF1A	0.06	undirected
HSPA5	DMXL2	0.11	undirected
HSPA5	CLK1	0.06	undirected
HSPA5	ATP2A2	0.07	undirected
HSPA5	IQGAP2	0.09	undirected
HSPA5	RB1	0.09	undirected
HSPA5	HNRNPU	0.06	undirected
ATP1B3	CREG1	0.08	undirected
ATP1B3	HERPUD1	0.06	undirected
ATP1B3	JUNB	0.10	undirected
ATP1B3	CD14	0.05	undirected
ATP1B3	MGAT1	0.05	undirected

Confidential: For Review Only

Garrido-Martin EM, *et al. J Immunother Cancer* 2020; 8:e000778. doi: 10.1136/jitc-2020-000778

BMJ Publishing Group Limited (BMJ) disclaims all liability and responsibility arising from any reliance placed on this supplemental material which has been supplied by the author(s)

ATP1B3	ELL2	0.05	undirected
ATP1B3	HSPH1	0.06	undirected
ATP1B3	ZFAND5	0.11	undirected
ATP1B3	NDFIP1	0.13	undirected
ATP1B3	UGCG	0.11	undirected
ATP1B3	PTP4A1	0.17	undirected
ATP1B3	TNFRSF14	0.09	undirected
ATP1B3	DMXL2	0.12	undirected
ATP1B3	PNRC1	0.08	undirected
ATP1B3	CTSS	0.08	undirected
ATP1B3	ATP2A2	0.11	undirected
ATP1B3	DNAJB4	0.06	undirected
ATP1B3	FAM21A	0.06	undirected
ATP1B3	MS4A6A	0.09	undirected
ATP1B3	NPL	0.09	undirected
ATP1B3	IQGAP2	0.11	undirected
ATP1B3	RB1	0.10	undirected
ATP1B3	TPP1	0.13	undirected
ATP1B3	HNRNPU	0.09	undirected
ATP1B3	ME1	0.12	undirected
CREG1	HERPUD1	0.11	undirected
CREG1	RGL1	0.05	undirected
CREG1	JUNB	0.06	undirected
CREG1	MGAT1	0.06	undirected
CREG1	ELL2	0.05	undirected
CREG1	HSPH1	0.06	undirected
CREG1	ZFAND5	0.07	undirected
CREG1	PTP4A1	0.09	undirected
CREG1	ENPP2	0.07	undirected
CREG1	HIF1A	0.05	undirected
CREG1	DMXL2	0.11	undirected
CREG1	ATF3	0.09	undirected
CREG1	CLK1	0.09	undirected
CREG1	ATP2A2	0.11	undirected
CREG1	GLA	0.06	undirected
CREG1	CLEC4E	0.08	undirected
CREG1	PIK3R1	0.06	undirected
CREG1	DNAJB4	0.06	undirected
CREG1	MS4A6A	0.08	undirected
CREG1	NPL	0.05	undirected
CREG1	IQGAP2	0.12	undirected
CREG1	SLC16A10	0.08	undirected
CREG1	RB1	0.06	undirected
CREG1	PAPSS2	0.07	undirected
CREG1	TPP1	0.09	undirected
CREG1	TFEC	0.11	undirected
CREG1	HNRNPU	0.05	undirected
GBP1	VAMP5	0.12	undirected
GBP1	TYMP	0.07	undirected

Confidential: For Review Only

GBP1	CCL13	0.05	undirected
GBP1	ICAM1	0.08	undirected
GBP1	ADAMDEC1	0.21	undirected
GBP1	RARRES3	0.23	undirected
GBP1	EPSTI1	0.06	undirected
GBP1	NR1H3	0.09	undirected
GBP1	TNFAIP3	0.08	undirected
GBP1	CD38	0.18	undirected
GBP1	LILRB4	0.05	undirected
GBP1	CMKLR1	0.08	undirected
GBP1	NINJ1	0.06	undirected
GBP1	CXCL12	0.12	undirected
GBP1	GBP5	0.29	undirected
GBP1	GBP4	0.31	undirected
GBP1	HLA-F	0.06	undirected
GBP1	IL32	0.16	undirected
GBP1	SLAMF7	0.28	undirected
GBP1	TNFSF13B	0.09	undirected
GBP1	HSD11B1	0.09	undirected
GBP1	CXCL11	0.25	undirected
GBP1	RASSF4	0.07	undirected
GBP1	GCH1	0.24	undirected
TSPAN4	VAMP5	0.07	undirected
TSPAN4	CD14	0.07	undirected
TSPAN4	MGAT1	0.11	undirected
TSPAN4	HLA-E	0.15	undirected
TSPAN4	ACP2	0.09	undirected
TSPAN4	HLA-DMB	0.07	undirected
TSPAN4	DMXL2	0.06	undirected
TSPAN4	LILRB4	0.08	undirected
TSPAN4	CLK1	0.06	undirected
TSPAN4	HLA-F	0.11	undirected
TSPAN4	PIK3R1	0.09	undirected
TSPAN4	IQGAP2	0.06	undirected
TSPAN4	SLC16A10	0.08	undirected
TSPAN4	TFEC	0.08	undirected
TSPAN4	HNRNPU	0.06	undirected
ARRDC3	DNAJB1	0.09	undirected
ARRDC3	BNIP3L	0.07	undirected
ARRDC3	ELL2	0.06	undirected
ARRDC3	ADM	0.10	undirected
ARRDC3	IL2RA	0.05	undirected
ARRDC3	CXCR4	0.08	undirected
ARRDC3	HIF1A	0.10	undirected
ARRDC3	CLK1	0.06	undirected
ARRDC3	SLC16A10	0.13	undirected
ARRDC3	PAPSS2	0.08	undirected
PFKFB3	DNAJB1	0.07	undirected
PFKFB3	ARL4C	0.07	undirected

Confidential: For Review Only

PFKFB3	SDS	0.08	undirected
PFKFB3	GADD45B	0.06	undirected
PFKFB3	TGIF1	0.06	undirected
VAMP5	CD14	0.11	undirected
VAMP5	TYMP	0.20	undirected
VAMP5	ARL4C	0.07	undirected
VAMP5	IER3	0.06	undirected
VAMP5	MGAT1	0.12	undirected
VAMP5	ZFAND5	0.08	undirected
VAMP5	ICAM1	0.15	undirected
VAMP5	RARRES3	0.19	undirected
VAMP5	EPSTI1	0.15	undirected
VAMP5	NR1H3	0.19	undirected
VAMP5	HLA-E	0.15	undirected
VAMP5	ACP2	0.19	undirected
VAMP5	CD38	0.18	undirected
VAMP5	TNFRSF14	0.06	undirected
VAMP5	TNFAIP2	0.09	undirected
VAMP5	IL18BP	0.16	undirected
VAMP5	LILRB4	0.22	undirected
VAMP5	CMKLR1	0.10	undirected
VAMP5	PNRC1	0.06	undirected
VAMP5	ATF5	0.14	undirected
VAMP5	CD4	0.08	undirected
VAMP5	NINJ1	0.19	undirected
VAMP5	MMP14	0.11	undirected
VAMP5	CXCL12	0.06	undirected
VAMP5	GBP5	0.11	undirected
VAMP5	GBP4	0.08	undirected
VAMP5	HLA-F	0.20	undirected
VAMP5	PIK3R1	0.08	undirected
VAMP5	IL32	0.18	undirected
VAMP5	SLAMF7	0.15	undirected
VAMP5	TNFSF13B	0.26	undirected
VAMP5	HSD11B1	0.05	undirected
VAMP5	CXCL11	0.05	undirected
VAMP5	RASSF4	0.18	undirected
HERPUD1	CD14	0.05	undirected
HERPUD1	MGAT1	0.08	undirected
HERPUD1	ZFAND5	0.06	undirected
HERPUD1	PTP4A1	0.05	undirected
HERPUD1	DMXL2	0.06	undirected
HERPUD1	ATF3	0.06	undirected
HERPUD1	CLK1	0.06	undirected
HERPUD1	ATP2A2	0.07	undirected
HERPUD1	PIK3R1	0.05	undirected
HERPUD1	A2M	0.08	undirected
HERPUD1	MS4A6A	0.12	undirected
HERPUD1	IQGAP2	0.07	undirected

Confidential: For Review Only

BMJ Publishing Group Limited (BMJ) disclaims all liability and responsibility arising from any reliance placed on this supplemental material which has been supplied by the author(s)

HERPUD1	TFEC	0.09	undirected
RGL1	HSPH1	0.05	undirected
RGL1	IL2RA	0.05	undirected
RGL1	PTP4A1	0.06	undirected
RGL1	ENPP2	0.10	undirected
RGL1	DMXL2	0.08	undirected
RGL1	CLK1	0.07	undirected
RGL1	IQGAP2	0.08	undirected
JUNB	CD14	0.05	undirected
JUNB	MGAT1	0.07	undirected
JUNB	ZFAND5	0.07	undirected
JUNB	NDFIP1	0.12	undirected
JUNB	UGCG	0.05	undirected
JUNB	PTP4A1	0.11	undirected
JUNB	DMXL2	0.10	undirected
JUNB	LILRB4	0.06	undirected
JUNB	PNRC1	0.20	undirected
JUNB	ATP2A2	0.10	undirected
JUNB	FAM21A	0.05	undirected
JUNB	MS4A6A	0.06	undirected
JUNB	IQGAP2	0.09	undirected
JUNB	RB1	0.13	undirected
JUNB	TPP1	0.11	undirected
JUNB	DUSP1	0.12	undirected
JUNB	HNRNPU-AS1	0.05	undirected
CD14	TYMP	0.05	undirected
CD14	IER3	0.05	undirected
CD14	MGAT1	0.13	undirected
CD14	ZFAND5	0.08	undirected
CD14	ICAM1	0.06	undirected
CD14	PTP4A1	0.06	undirected
CD14	HLA-E	0.12	undirected
CD14	ACP2	0.07	undirected
CD14	IL18BP	0.07	undirected
CD14	DMXL2	0.10	undirected
CD14	LILRB4	0.11	undirected
CD14	ATF5	0.09	undirected
CD14	CD4	0.07	undirected
CD14	NINJ1	0.06	undirected
CD14	ATP2A2	0.06	undirected
CD14	HLA-F	0.09	undirected
CD14	PIK3R1	0.07	undirected
CD14	TNFSF13B	0.07	undirected
CD14	RASSF4	0.06	undirected
CD14	IQGAP2	0.09	undirected
CD14	SLC16A10	0.05	undirected
CD14	TFEC	0.08	undirected
CD14	HNRNPU	0.06	undirected
CD14	HNRNPU-AS1	0.06	undirected

Confidential: For Review Only

Garrido-Martin EM, et al. J Immunother Cancer 2020; 8:e000778. doi: 10.1136/jitc-2020-000778

BMJ Publishing Group Limited (BMJ) disclaims all liability and responsibility arising from any reliance placed on this supplemental material which has been supplied by the author(s)

TYMP	IER3	0.06	undirected
TYMP	MGAT1	0.05	undirected
TYMP	ZFAND5	0.06	undirected
TYMP	ICAM1	0.09	undirected
TYMP	RARRES3	0.10	undirected
TYMP	NR1H3	0.12	undirected
TYMP	HLA-E	0.08	undirected
TYMP	ACP2	0.14	undirected
TYMP	CD38	0.09	undirected
TYMP	TNFRSF14	0.06	undirected
TYMP	TNFAIP2	0.05	undirected
TYMP	IL18BP	0.11	undirected
TYMP	LILRB4	0.15	undirected
TYMP	ATF5	0.09	undirected
TYMP	NINJ1	0.13	undirected
TYMP	MMP14	0.10	undirected
TYMP	GBP5	0.07	undirected
TYMP	HLA-F	0.11	undirected
TYMP	IL32	0.11	undirected
TYMP	SLAMF7	0.08	undirected
TYMP	TNFSF13B	0.11	undirected
TYMP	RASSF4	0.08	undirected
DNAJB1	BNIP3L	0.08	undirected
DNAJB1	HSPH1	0.07	undirected
DNAJB1	GADD45B	0.14	undirected
DNAJB1	ADM	0.22	undirected
DNAJB1	IL2RA	0.05	undirected
DNAJB1	CXCR4	0.06	undirected
DNAJB1	DNAJA4	0.07	undirected
DNAJB1	EIF4A3	0.09	undirected
DNAJB1	PLIN2	0.07	undirected
ARL4C	SDS	0.06	undirected
ARL4C	LILRB4	0.07	undirected
ARL4C	NINJ1	0.10	undirected
BNIP3L	ADM	0.14	undirected
BNIP3L	CXCR4	0.06	undirected
BNIP3L	SLC16A10	0.08	undirected
BNIP3L	PLIN2	0.17	undirected
IER3	LILRB4	0.06	undirected
IER3	ATF5	0.09	undirected
CCL13	TNFAIP3	0.05	undirected
CCL13	GBP5	0.08	undirected
CCL13	CLEC4E	0.05	undirected
MGAT1	ZFAND5	0.09	undirected
MGAT1	NR1H3	0.06	undirected
MGAT1	PTP4A1	0.06	undirected
MGAT1	HLA-E	0.15	undirected
MGAT1	ENPP2	0.05	undirected
MGAT1	ACP2	0.09	undirected

Confidential: For Review Only

Garrido-Martin EM, et al. J Immunother Cancer 2020; 8:e000778. doi: 10.1136/jitc-2020-000778

MGAT1	TNFRSF14	0.05	undirected
MGAT1	TNFAIP2	0.05	undirected
MGAT1	IL18BP	0.06	undirected
MGAT1	DMXL2	0.09	undirected
MGAT1	LILRB4	0.14	undirected
MGAT1	ATF3	0.05	undirected
MGAT1	PNRC1	0.07	undirected
MGAT1	ATF5	0.09	undirected
MGAT1	NINJ1	0.06	undirected
MGAT1	CLK1	0.06	undirected
MGAT1	ATP2A2	0.09	undirected
MGAT1	HLA-F	0.14	undirected
MGAT1	PIK3R1	0.10	undirected
MGAT1	DNAJB4	0.05	undirected
MGAT1	MS4A6A	0.08	undirected
MGAT1	TNFSF13B	0.09	undirected
MGAT1	RASSF4	0.06	undirected
MGAT1	IQGAP2	0.09	undirected
MGAT1	SLC16A10	0.06	undirected
MGAT1	RB1	0.07	undirected
MGAT1	TFEC	0.12	undirected
MGAT1	HNRNPU	0.08	undirected
MGAT1	HNRNPU-AS1	0.09	undirected
FNIP2	PTP4A1	0.05	undirected
FNIP2	HLA-E	0.07	undirected
FNIP2	HIF1A	0.07	undirected
FNIP2	DMXL2	0.11	undirected
FNIP2	CD4	0.06	undirected
FNIP2	CLK1	0.12	undirected
FNIP2	CLEC4E	0.06	undirected
FNIP2	PIK3R1	0.08	undirected
FNIP2	IQGAP2	0.09	undirected
FNIP2	SLC16A10	0.09	undirected
FNIP2	TFEC	0.06	undirected
FNIP2	HNRNPU	0.06	undirected
ELL2	HSPH1	0.06	undirected
ELL2	UGCG	0.05	undirected
ELL2	PTP4A1	0.08	undirected
ELL2	HIF1A	0.13	undirected
ELL2	DMXL2	0.11	undirected
ELL2	CTSS	0.07	undirected
ELL2	CLK1	0.09	undirected
ELL2	GLA	0.08	undirected
ELL2	IQGAP2	0.10	undirected
ELL2	SLC16A10	0.12	undirected
ELL2	PAPSS2	0.07	undirected
SDS	LILRB4	0.06	undirected
SDS	PNRC1	0.06	undirected
HSPH1	ZFAND5	0.05	undirected

Confidential: For Review Only

Garrido-Martin EM, et al. J Immunother Cancer 2020; 8:e000778. doi: 10.1136/jitc-2020-000778

HSPH1	PTP4A1	0.10	undirected
HSPH1	DNAJA4	0.07	undirected
HSPH1	HIF1A	0.08	undirected
HSPH1	DMXL2	0.12	undirected
HSPH1	CLK1	0.09	undirected
HSPH1	ATP2A2	0.06	undirected
HSPH1	DNAJB4	0.06	undirected
HSPH1	EIF4A3	0.09	undirected
HSPH1	IQGAP2	0.11	undirected
HSPH1	SLC16A10	0.08	undirected
HSPH1	RB1	0.05	undirected
HSPH1	HNRNPU	0.06	undirected
ZFAND5	NDFIP1	0.06	undirected
ZFAND5	UGCG	0.07	undirected
ZFAND5	PTP4A1	0.13	undirected
ZFAND5	HLA-E	0.08	undirected
ZFAND5	ACP2	0.06	undirected
ZFAND5	TNFRSF14	0.05	undirected
ZFAND5	DMXL2	0.13	undirected
ZFAND5	LILRB4	0.07	undirected
ZFAND5	ATF5	0.06	undirected
ZFAND5	CLK1	0.06	undirected
ZFAND5	ATP2A2	0.09	undirected
ZFAND5	HLA-F	0.06	undirected
ZFAND5	PIK3R1	0.06	undirected
ZFAND5	DNAJB4	0.05	undirected
ZFAND5	MS4A6A	0.07	undirected
ZFAND5	IQGAP2	0.12	undirected
ZFAND5	SLC16A10	0.05	undirected
ZFAND5	RB1	0.09	undirected
ZFAND5	TPP1	0.07	undirected
ZFAND5	TFEC	0.06	undirected
ZFAND5	HNRNPU	0.08	undirected
ZFAND5	ME1	0.08	undirected
ZFAND5	HNRNPU-AS1	0.07	undirected
ICAM1	RARRES3	0.09	undirected
ICAM1	NR1H3	0.08	undirected
ICAM1	HLA-E	0.07	undirected
ICAM1	ACP2	0.09	undirected
ICAM1	CD38	0.07	undirected
ICAM1	IL18BP	0.10	undirected
ICAM1	LILRB4	0.10	undirected
ICAM1	ATF5	0.07	undirected
ICAM1	CD4	0.07	undirected
ICAM1	NINJ1	0.10	undirected
ICAM1	MMP14	0.10	undirected
ICAM1	GBP5	0.06	undirected
ICAM1	HLA-F	0.09	undirected
ICAM1	IL32	0.08	undirected

Confidential: For Review Only

ICAM1	SLAMF7	0.07	undirected
ICAM1	TNFSF13B	0.08	undirected
ICAM1	RASSF4	0.07	undirected
NDFIP1	PTP4A1	0.12	undirected
NDFIP1	DMXL2	0.05	undirected
NDFIP1	PNRC1	0.09	undirected
NDFIP1	ATP2A2	0.08	undirected
NDFIP1	RB1	0.10	undirected
NDFIP1	TPP1	0.10	undirected
GADD45B	ADM	0.05	undirected
UGCG	PTP4A1	0.17	undirected
UGCG	HIF1A	0.09	undirected
UGCG	DMXL2	0.19	undirected
UGCG	CTSS	0.05	undirected
UGCG	CLK1	0.09	undirected
UGCG	ATP2A2	0.06	undirected
UGCG	DNAJB4	0.07	undirected
UGCG	NPL	0.08	undirected
UGCG	IQGAP2	0.14	undirected
UGCG	SLC16A10	0.06	undirected
UGCG	RB1	0.05	undirected
UGCG	HNRNPU	0.12	undirected
UGCG	ME1	0.08	undirected
CECR1	HLA-E	0.07	undirected
CECR1	HLA-F	0.05	undirected
ADAMDEC1	RARRES3	0.10	undirected
ADAMDEC1	TNFAIP3	0.08	undirected
ADAMDEC1	CD38	0.07	undirected
ADAMDEC1	CXCL12	0.05	undirected
ADAMDEC1	GBP5	0.22	undirected
ADAMDEC1	GBP4	0.21	undirected
ADAMDEC1	IL32	0.07	undirected
ADAMDEC1	SLAMF7	0.15	undirected
ADAMDEC1	CXCL11	0.12	undirected
ADAMDEC1	GCH1	0.12	undirected
RARRES3	EPSTI1	0.09	undirected
RARRES3	NR1H3	0.11	undirected
RARRES3	HLA-E	0.07	undirected
RARRES3	ACP2	0.08	undirected
RARRES3	CD38	0.19	undirected
RARRES3	IL18BP	0.08	undirected
RARRES3	LILRB4	0.10	undirected
RARRES3	CMKLR1	0.09	undirected
RARRES3	ATF5	0.05	undirected
RARRES3	NINJ1	0.08	undirected
RARRES3	MMP14	0.05	undirected
RARRES3	CXCL12	0.08	undirected
RARRES3	GBP5	0.18	undirected
RARRES3	GBP4	0.18	undirected

Confidential: For Review Only

RARRES3	HLA-F	0.11	undirected
RARRES3	IL32	0.18	undirected
RARRES3	SLAMF7	0.22	undirected
RARRES3	TNFSF13B	0.12	undirected
RARRES3	HSD11B1	0.07	undirected
RARRES3	CXCL11	0.14	undirected
RARRES3	RASSF4	0.11	undirected
RARRES3	GCH1	0.11	undirected
EPSTI1	HLA-E	0.05	undirected
EPSTI1	CD38	0.09	undirected
EPSTI1	LILRB4	0.09	undirected
EPSTI1	HLA-F	0.07	undirected
EPSTI1	SLAMF7	0.06	undirected
EPSTI1	TNFSF13B	0.14	undirected
KLF2	PNRC1	0.05	undirected
KLF2	DUSP1	0.07	undirected
ADM	CXCR4	0.08	undirected
ADM	PLIN2	0.17	undirected
IL2RA	ENPP2	0.10	undirected
NR1H3	HLA-E	0.10	undirected
NR1H3	ACP2	0.18	undirected
NR1H3	CD38	0.08	undirected
NR1H3	TNFAIP2	0.08	undirected
NR1H3	IL18BP	0.09	undirected
NR1H3	LILRB4	0.16	undirected
NR1H3	ATF5	0.07	undirected
NR1H3	NINJ1	0.11	undirected
NR1H3	MMP14	0.08	undirected
NR1H3	GBP5	0.07	undirected
NR1H3	GBP4	0.05	undirected
NR1H3	HLA-F	0.14	undirected
NR1H3	IL32	0.10	undirected
NR1H3	SLAMF7	0.10	undirected
NR1H3	TNFSF13B	0.11	undirected
NR1H3	HSD11B1	0.06	undirected
NR1H3	RASSF4	0.09	undirected
PTP4A1	ENPP2	0.05	undirected
PTP4A1	TNFRSF14	0.09	undirected
PTP4A1	HIF1A	0.11	undirected
PTP4A1	DMXL2	0.17	undirected
PTP4A1	PNRC1	0.07	undirected
PTP4A1	CTSS	0.08	undirected
PTP4A1	CLK1	0.09	undirected
PTP4A1	ATP2A2	0.13	undirected
PTP4A1	FMNL2	0.06	undirected
PTP4A1	PIK3R1	0.06	undirected
PTP4A1	DNAJB4	0.11	undirected
PTP4A1	MS4A6A	0.08	undirected
PTP4A1	NPL	0.09	undirected

Confidential: For Review Only

PTP4A1	IQGAP2	0.14	undirected
PTP4A1	SLC16A10	0.07	undirected
PTP4A1	RB1	0.15	undirected
PTP4A1	TPP1	0.11	undirected
PTP4A1	TFEC	0.06	undirected
PTP4A1	HNRNPU	0.14	undirected
PTP4A1	ME1	0.13	undirected
PTP4A1	HNRNPU-AS1	0.08	undirected
HLA-E	ACP2	0.12	undirected
HLA-E	CD38	0.06	undirected
HLA-E	TNFRSF14	0.05	undirected
HLA-E	TNFAIP2	0.08	undirected
HLA-E	IL18BP	0.11	undirected
HLA-E	HIF1A	0.05	undirected
HLA-E	HLA-DMB	0.10	undirected
HLA-E	DMXL2	0.07	undirected
HLA-E	LILRB4	0.14	undirected
HLA-E	CMKLR1	0.05	undirected
HLA-E	ATF5	0.11	undirected
HLA-E	CD4	0.11	undirected
HLA-E	NINJ1	0.07	undirected
HLA-E	CLK1	0.08	undirected
HLA-E	ATP2A2	0.05	undirected
HLA-E	HLA-F	0.18	undirected
HLA-E	PIK3R1	0.12	undirected
HLA-E	DNAJB4	0.05	undirected
HLA-E	IL32	0.07	undirected
HLA-E	TNFSF13B	0.11	undirected
HLA-E	RASSF4	0.11	undirected
HLA-E	IQGAP2	0.07	undirected
HLA-E	SLC16A10	0.09	undirected
HLA-E	TFEC	0.09	undirected
HLA-E	HNRNPU	0.07	undirected
HLA-E	HNRNPU-AS1	0.05	undirected
ENPP2	DMXL2	0.06	undirected
ENPP2	CLK1	0.06	undirected
ENPP2	ATP2A2	0.09	undirected
ENPP2	MS4A6A	0.06	undirected
ENPP2	IQGAP2	0.07	undirected
ENPP2	RB1	0.05	undirected
ENPP2	TFEC	0.06	undirected
ACP2	CD38	0.08	undirected
ACP2	TNFRSF14	0.07	undirected
ACP2	TNFAIP2	0.07	undirected
ACP2	IL18BP	0.13	undirected
ACP2	LILRB4	0.17	undirected
ACP2	ATF5	0.11	undirected
ACP2	CD4	0.05	undirected
ACP2	NINJ1	0.13	undirected

Confidential: For Review Only

Garrido-Martin EM, et al. J Immunother Cancer 2020; 8:e000778. doi: 10.1136/jitc-2020-000778

ACP2	MMP14	0.10	undirected
ACP2	HLA-F	0.15	undirected
ACP2	PIK3R1	0.07	undirected
ACP2	IL32	0.09	undirected
ACP2	SLAMF7	0.06	undirected
ACP2	TNFSF13B	0.11	undirected
ACP2	RASSF4	0.09	undirected
TNFAIP3	ATF3	0.05	undirected
TNFAIP3	GBP5	0.13	undirected
TNFAIP3	GBP4	0.11	undirected
TNFAIP3	LCP2	0.07	undirected
TNFAIP3	SLAMF7	0.06	undirected
CD38	IL18BP	0.08	undirected
CD38	LILRB4	0.10	undirected
CD38	CMKLR1	0.08	undirected
CD38	NINJ1	0.07	undirected
CD38	CXCL12	0.07	undirected
CD38	GBP5	0.14	undirected
CD38	GBP4	0.12	undirected
CD38	HLA-F	0.10	undirected
CD38	IL32	0.13	undirected
CD38	SLAMF7	0.17	undirected
CD38	TNFSF13B	0.11	undirected
CD38	HSD11B1	0.05	undirected
CD38	CXCL11	0.11	undirected
CD38	RASSF4	0.07	undirected
CD38	GCH1	0.07	undirected
CXCR4	HIF1A	0.07	undirected
CXCR4	CLK1	0.07	undirected
CXCR4	CLEC4E	0.07	undirected
CXCR4	SLC16A10	0.09	undirected
CXCR4	PLIN2	0.06	undirected
TNFRSF14	DMXL2	0.06	undirected
TNFRSF14	LILRB4	0.05	undirected
TNFRSF14	ATF5	0.06	undirected
TNFRSF14	IQGAP2	0.06	undirected
TNFRSF14	HNRNPU	0.05	undirected
TNFAIP2	LILRB4	0.11	undirected
TNFAIP2	HLA-F	0.11	undirected
TNFAIP2	TNFSF13B	0.05	undirected
TNFAIP2	RASSF4	0.06	undirected
DNAJA4	CLK1	0.06	undirected
IL18BP	LILRB4	0.12	undirected
IL18BP	CMKLR1	0.05	undirected
IL18BP	ATF5	0.11	undirected
IL18BP	CD4	0.08	undirected
IL18BP	NINJ1	0.10	undirected
IL18BP	MMP14	0.08	undirected
IL18BP	HLA-F	0.12	undirected

Confidential: For Review Only

IL18BP	IL32	0.09	undirected
IL18BP	SLAMF7	0.06	undirected
IL18BP	TNFSF13B	0.08	undirected
IL18BP	RASSF4	0.07	undirected
HIF1A	DMXL2	0.14	undirected
HIF1A	CLK1	0.12	undirected
HIF1A	DNAJB4	0.05	undirected
HIF1A	IQGAP2	0.12	undirected
HIF1A	SLC16A10	0.14	undirected
HIF1A	PAPSS2	0.08	undirected
HIF1A	TFEC	0.05	undirected
HIF1A	HNRNPU	0.07	undirected
HLA-DMB	CD4	0.06	undirected
HLA-DMB	HLA-F	0.07	undirected
HLA-DMB	PIK3R1	0.06	undirected
HLA-DMB	SLC16A10	0.10	undirected
DMXL2	ATF3	0.07	undirected
DMXL2	PNRC1	0.05	undirected
DMXL2	CD4	0.07	undirected
DMXL2	CTSS	0.07	undirected
DMXL2	CLK1	0.15	undirected
DMXL2	ATP2A2	0.12	undirected
DMXL2	FMNL2	0.14	undirected
DMXL2	CLEC4E	0.06	undirected
DMXL2	PIK3R1	0.10	undirected
DMXL2	DNAJB4	0.14	undirected
DMXL2	MS4A6A	0.07	undirected
DMXL2	NPL	0.11	undirected
DMXL2	IQGAP2	0.19	undirected
DMXL2	SLC16A10	0.11	undirected
DMXL2	RB1	0.13	undirected
DMXL2	PAPSS2	0.07	undirected
DMXL2	TPP1	0.08	undirected
DMXL2	TFEC	0.11	undirected
DMXL2	HNRNPU	0.19	undirected
DMXL2	ME1	0.13	undirected
DMXL2	HNRNPU-AS1	0.15	undirected
LILRB4	PNRC1	0.08	undirected
LILRB4	ATF5	0.12	undirected
LILRB4	CD4	0.05	undirected
LILRB4	NINJ1	0.16	undirected
LILRB4	MMP14	0.08	undirected
LILRB4	HLA-F	0.19	undirected
LILRB4	PIK3R1	0.08	undirected
LILRB4	IL32	0.09	undirected
LILRB4	SLAMF7	0.07	undirected
LILRB4	TNFSF13B	0.18	undirected
LILRB4	RASSF4	0.12	undirected
LILRB4	TFEC	0.06	undirected

Confidential: For Review Only

Garrido-Martin EM, et al. J Immunother Cancer 2020; 8:e000778. doi: 10.1136/jitc-2020-000778

CMKLR1	GBP5	0.06	undirected
CMKLR1	GBP4	0.05	undirected
CMKLR1	HLA-F	0.06	undirected
CMKLR1	IL32	0.12	undirected
CMKLR1	SLAMF7	0.10	undirected
CMKLR1	TNFSF13B	0.05	undirected
CMKLR1	RASSF4	0.07	undirected
ATF3	CLK1	0.11	undirected
ATF3	ATP2A2	0.07	undirected
ATF3	CLEC4E	0.07	undirected
ATF3	EIF4A3	0.05	undirected
ATF3	IQGAP2	0.07	undirected
ATF3	SLC16A10	0.07	undirected
ATF3	TFEC	0.15	undirected
FYB	LCP2	0.06	undirected
PNRC1	NINJ1	0.05	undirected
PNRC1	ATP2A2	0.08	undirected
PNRC1	FAM21A	0.07	undirected
PNRC1	IQGAP2	0.05	undirected
PNRC1	RB1	0.07	undirected
PNRC1	TPP1	0.09	undirected
PNRC1	DUSP1	0.14	undirected
ATF5	NINJ1	0.09	undirected
ATF5	MMP14	0.06	undirected
ATF5	HLA-F	0.10	undirected
ATF5	IL32	0.06	undirected
ATF5	TNFSF13B	0.07	undirected
ATF5	RASSF4	0.05	undirected
CD4	HLA-F	0.07	undirected
CD4	RASSF4	0.07	undirected
CD4	IQGAP2	0.06	undirected
CD4	SLC16A10	0.07	undirected
NINJ1	MMP14	0.09	undirected
NINJ1	GBP5	0.06	undirected
NINJ1	HLA-F	0.11	undirected
NINJ1	IL32	0.08	undirected
NINJ1	SLAMF7	0.07	undirected
NINJ1	TNFSF13B	0.12	undirected
NINJ1	RASSF4	0.07	undirected
CTSS	NPL	0.08	undirected
CTSS	IQGAP2	0.06	undirected
CLK1	ATP2A2	0.07	undirected
CLK1	CLEC4E	0.08	undirected
CLK1	PIK3R1	0.09	undirected
CLK1	DNAJB4	0.12	undirected
CLK1	NPL	0.05	undirected
CLK1	IQGAP2	0.14	undirected
CLK1	SLC16A10	0.12	undirected
CLK1	PAPSS2	0.07	undirected

Confidential: For Review Only

Supplemental material BMJ Publishing Group Limited (BMJ) disclaims all liability and responsibility arising from any reliance placed on this supplemental material which has been supplied by the author(s) J Immunother Cancer

CLK1	TFEC	0.10	undirected
CLK1	HNRNPU	0.11	undirected
CLK1	HNRNPU-AS1	0.06	undirected
CH25H	GBP5	0.06	undirected
MMP14	HLA-F	0.06	undirected
MMP14	IL32	0.06	undirected
MMP14	TNFSF13B	0.06	undirected
MMP14	RASSF4	0.05	undirected
ATP2A2	PIK3R1	0.06	undirected
ATP2A2	DNAJB4	0.06	undirected
ATP2A2	EIF4A3	0.05	undirected
ATP2A2	MS4A6A	0.08	undirected
ATP2A2	IQGAP2	0.13	undirected
ATP2A2	SLC16A10	0.05	undirected
ATP2A2	RB1	0.10	undirected
ATP2A2	TPP1	0.10	undirected
ATP2A2	TFEC	0.10	undirected
ATP2A2	HNRNPU	0.07	undirected
ATP2A2	ME1	0.05	undirected
ATP2A2	HNRNPU-AS1	0.07	undirected
CXCL12	GBP5	0.09	undirected
CXCL12	GBP4	0.09	undirected
CXCL12	IL32	0.05	undirected
CXCL12	SLAMF7	0.10	undirected
CXCL12	CXCL11	0.08	undirected
GBP5	GBP4	0.29	undirected
GBP5	LCP2	0.06	undirected
GBP5	IL32	0.14	undirected
GBP5	SLAMF7	0.23	undirected
GBP5	TNFSF13B	0.07	undirected
GBP5	HSD11B1	0.08	undirected
GBP5	CXCL11	0.18	undirected
GBP5	GCH1	0.19	undirected
GBP4	LCP2	0.07	undirected
GBP4	IL32	0.12	undirected
GBP4	SLAMF7	0.22	undirected
GBP4	HSD11B1	0.07	undirected
GBP4	CXCL11	0.20	undirected
GBP4	GCH1	0.20	undirected
GLA	SLC16A10	0.06	undirected
GLA	PAPSS2	0.05	undirected
GLA	TPP1	0.06	undirected
FMNL2	IQGAP2	0.11	undirected
FMNL2	HNRNPU	0.05	undirected
FMNL2	HNRNPU-AS1	0.05	undirected
CLEC4E	IQGAP2	0.06	undirected
CLEC4E	SLC16A10	0.08	undirected
CLEC4E	TFEC	0.09	undirected
HLA-F	PIK3R1	0.10	undirected

Confidential: For Review Only

Garrido-Martin EM, et al. J Immunother Cancer 2020; 8:e000778. doi: 10.1136/jitc-2020-000778

HLA-F	IL32	0.09	undirected
HLA-F	SLAMF7	0.07	undirected
HLA-F	TNFSF13B	0.14	undirected
HLA-F	RASSF4	0.13	undirected
HLA-F	SLC16A10	0.05	undirected
HLA-F	TFEC	0.06	undirected
PIK3R1	DNAJB4	0.06	undirected
PIK3R1	IQGAP2	0.12	undirected
PIK3R1	SLC16A10	0.10	undirected
PIK3R1	PAPSS2	0.05	undirected
PIK3R1	TFEC	0.10	undirected
PIK3R1	HNRNPU	0.07	undirected
PIK3R1	HNRNPU-AS1	0.05	undirected
A2M	MS4A6A	0.09	undirected
DNAJB4	IQGAP2	0.12	undirected
DNAJB4	SLC16A10	0.07	undirected
DNAJB4	TFEC	0.06	undirected
DNAJB4	HNRNPU	0.07	undirected
FAM21A	MS4A6A	0.09	undirected
IL32	SLAMF7	0.17	undirected
IL32	TNFSF13B	0.09	undirected
IL32	HSD11B1	0.06	undirected
IL32	CXCL11	0.07	undirected
IL32	RASSF4	0.10	undirected
IL32	GCH1	0.05	undirected
MS4A6A	IQGAP2	0.07	undirected
MS4A6A	RB1	0.05	undirected
MS4A6A	TPP1	0.06	undirected
SLAMF7	TNFSF13B	0.09	undirected
SLAMF7	HSD11B1	0.08	undirected
SLAMF7	CXCL11	0.16	undirected
SLAMF7	RASSF4	0.08	undirected
SLAMF7	GCH1	0.15	undirected
NPL	IQGAP2	0.10	undirected
NPL	TPP1	0.06	undirected
NPL	ME1	0.07	undirected
TNFSF13B	RASSF4	0.10	undirected
HSD11B1	CXCL11	0.07	undirected
CXCL11	GCH1	0.21	undirected
IQGAP2	SLC16A10	0.11	undirected
IQGAP2	RB1	0.10	undirected
IQGAP2	PAPSS2	0.09	undirected
IQGAP2	TPP1	0.08	undirected
IQGAP2	TFEC	0.12	undirected
IQGAP2	HNRNPU	0.15	undirected
IQGAP2	ME1	0.11	undirected
IQGAP2	HNRNPU-AS1	0.11	undirected
SLC16A10	PAPSS2	0.17	undirected
SLC16A10	TFEC	0.09	undirected

Confidential: For Review Only

SLC16A10	HNRNPU	0.08	undirected
RB1	TPP1	0.06	undirected
RB1	HNRNPU	0.06	undirected
PAPSS2	TFEC	0.06	undirected
TFEC	HNRNPU	0.07	undirected
TFEC	HNRNPU-AS1	0.07	undirected
HNRNPU	ME1	0.06	undirected
HNRNPU	HNRNPU-AS1	0.08	undirected

Confidential: For Review Only

Supplemental material
BMJ Publishing Group Limited (BMJ) disclaims all liability and responsibility arising from any reliance placed on this supplemental material which has been supplied by the author(s)

fied population analysis of NSCLC TAMs

des

Label
HSPA1A
B2M
SLC40A1
HSPA1B
SOD2
HLA-A
SEPP1
SDC3
NFKBIA
HSP90AA1
TMEM176A
TSC22D1
TMEM176B
CXCL9
HSPB1
SLC1A3
SMPDL3A
STAT1
FOLR2
CCL3
SDCBP
ATOX1
SGK1
MAFB
HLA-B
CXCL10
PDK4
HSPA5
ATP1B3
UBE2D1
CREG1
GBP1
TSPAN4
ARRDC3
PFKFB3
VAMP5
HERPUD1
RGL1
JUNB
CD14
TYMP
SNAP23
DNAJB1
ARL4C
BNIP3L

Confidential: For Review Only

Garrido-Martin EM, et al. J Immunother Cancer 2020; 8:e000778. doi: 10.1136/jitc-2020-000778

BMJ Publishing Group Limited (BMJ) disclaims all liability and responsibility arising from any reliance placed on this supplemental material which has been supplied by the author(s)

IER3
CCL13
MGAT1
FNIP2
ELL2
SDS
HSPH1
ZFAND5
ICAM1
NDFIP1
GADD45B
UGCG
CECR1
ADAMDEC1
RARRES3
EPSTI1
KLF2
ADM
IL2RA
NR1H3
PTP4A1
HLA-E
ENPP2
ACP2
TNFAIP3
CD38
CXCR4
TNFRSF14
TNFAIP2
DNAJA4
IL18BP
HIF1A
HLA-DMB
DMXL2
LILRB4
CMKLR1
TGIF1
ATF3
FYB
PNRC1
ATF5
CD4
NINJ1
CTSS
CLK1
CH25H
MMP14
ATP2A2
CXCL12

Confidential: For Review Only

GBP5
KCTD12
GBP4
GLA
FMNL2
CLEC4E
HLA-F
PIK3R1
A2M
LCP2
DNAJB4
FAM21A
EIF4A3
IL32
MS4A6A
SLAMF7
NPL
TNFSF13B
HSD11B1
CXCL11
RASSF4
IQGAP2
SLC16A10
RB1
PAPSS2
PLIN2
TPP1
GCH1
TFEC
HNRNPU
ME1
DUSP1
HNRNPU-AS1

Confidential: For Review Only

Gene	baseMean	log2FoldChange	lfcMLE	lfcSE	stat
CXCL9	4512.43678	5.170507467	6.103390762	0.327520205	15.78683509
CXCL10	190.1423226	3.727586265	4.267824379	0.303558173	12.27964387
UBD	240.7025689	4.034269428	5.055830536	0.363603001	11.09525889
GBP1	500.7827492	2.670932013	2.915565845	0.255221328	10.46515993
FAM26F	116.383238	3.187019923	3.849342572	0.344093893	9.262064766
SLAMF7	904.6340018	2.387629611	2.692383167	0.291424295	8.192966932
TNFRSF9	45.96779735	2.261271899	2.520129674	0.27832711	8.124511825
GBP4	356.5740555	2.40838949	2.746443017	0.302091422	7.972386227
STAT1	1191.464254	1.84907342	1.98426701	0.232094175	7.966910054
CXCL11	41.6841633	3.057087909	4.005585349	0.386137937	7.9170877
IDO1	96.08202552	3.131046377	4.364571416	0.408261326	7.669221097
PSTPIP2	185.9441512	1.816466287	1.958183846	0.238587276	7.613424811
GCH1	73.23338882	2.447898457	2.897330027	0.332613684	7.359584326
GBP5	721.3940221	2.447920405	2.915710628	0.337077873	7.262180639
KCNJ10	49.33578881	2.915854168	4.224063797	0.419170342	6.95625114
TAP1	523.6490667	1.526447841	1.625935752	0.220755291	6.914660267
AIM2	46.86936296	2.609173918	3.391857251	0.385585116	6.766791063
CP	15.11263423	2.883503815	4.522480022	0.43237743	6.668950822
RARRES3	387.3089009	1.953402826	2.225281979	0.301321565	6.482784683
IL32	97.89584969	2.260212137	2.751816486	0.350864503	6.441837575
CD96	11.07934691	2.756758539	4.482989866	0.434591422	6.343333991
APOL3	154.5613584	1.816569715	2.047288839	0.290900716	6.244638174
ANKRD22	232.1423049	2.281670746	2.849107409	0.366338972	6.228304717
ZBTB7C	14.6151735	-2.510569835	-3.826254317	0.426942271	-5.880349658
CD274	84.17610501	1.953247466	2.311220736	0.332787275	5.869357437
IRF1	339.323677	1.662265963	1.862481201	0.285137577	5.82969801
PPA1	143.4716337	1.616536447	1.797786214	0.27738706	5.827728393
GBP2	357.9714831	1.624720684	1.812957835	0.281014805	5.781619533
C19orf59	1553.906602	-2.031442279	-2.48463862	0.354940633	-5.723329735
TNNI2	38.70602634	-1.682185783	-1.92438981	0.302907975	-5.553454924
IL17RB	46.92695951	-2.26312732	-3.154301581	0.410267265	-5.51622689
DNM1	12.84992552	-2.290348082	-3.293524863	0.417286034	-5.488676576
SOD2	1544.752564	1.685174525	1.931119413	0.306984685	5.489441682
GZMA	17.03863268	2.44929692	4.196864672	0.446924037	5.480342787
HIC1	22.48479022	2.099456151	2.736309023	0.38402325	5.467002712
SIGLEC10	480.8902563	1.828362011	2.169919851	0.334975915	5.458189469
GRIN3A	19.93831121	2.042544305	2.60796801	0.377777318	5.40674151
WARS	1054.205733	1.338911867	1.454213031	0.248928578	5.378698904
MYOF	603.1487488	1.214423605	1.303389896	0.232438722	5.224704355
AGRP	75.52732232	-1.970343632	-2.519032705	0.377698898	-5.216704739
FAM46C	11.83062945	2.308347775	3.886663865	0.445690114	5.179266275
IL2RA	320.0182979	1.905708343	2.385019864	0.368207407	5.175638256
BIRC7	75.86123373	-2.115946613	-2.936846511	0.41008673	-5.159753916
KLHDC7B	47.3769355	1.634031365	1.904762386	0.320952908	5.091187289
LAP3	782.2716115	1.144086271	1.22308826	0.226532345	5.050432286

GZMB	28.60759796	2.313894752	5.386371668	0.460678276	5.022799798
PDE4B	278.9907876	1.62727787	1.910619185	0.327092462	4.974978199
TNFSF10	144.7946533	1.197403078	1.293000353	0.24086858	4.971188338
CXCR2P1	78.15582347	2.041095635	2.836645634	0.411003767	4.966123907
CD9	968.674048	-1.743618331	-2.120321571	0.351638153	-4.95855844
PDCD1LG2	124.0522743	1.700957946	2.049946524	0.346080943	4.91491363
DUSP5	54.74381251	1.361792163	1.517728385	0.279583669	4.870785794
HSPH1	281.4794656	1.637693911	1.945527486	0.336025977	4.873712222
CCL5	246.2479485	1.893662012	2.478313099	0.38955793	4.861053693
BAG3	194.9175561	1.519322049	1.752585387	0.31279148	4.857299978
CD38	87.33807137	1.837932931	2.367994747	0.382389186	4.80644589
CEACAM8	41.89050637	-2.077501545	-3.182856037	0.434641376	-4.779806205
CD3E	12.19496552	2.140425131	3.779927206	0.448703052	4.770248656
PAQR5	18.44890216	-1.739508511	-2.182050371	0.365993134	-4.752844658
SNORD104	47.97400821	-1.552318517	-1.827524078	0.327550311	-4.739175832
ADAMDEC1	338.0778081	2.027009411	3.023331534	0.430250496	4.711230851
CD52	411.8459839	-1.698523791	-2.101352577	0.361620792	-4.696974923
SIK1	223.5149145	1.453363748	1.67010195	0.3094927	4.695954863
SLC39A8	49.30189053	1.667253858	2.041985416	0.35659855	4.675436444
PLEK	997.4261377	1.043377175	1.113258112	0.223547969	4.667352503
MMP25	59.3999198	1.85996473	2.524648297	0.402945816	4.615917714
ENPP2	174.0567939	1.463515525	1.700067792	0.3190211	4.587519527
MIR34A	52.27568979	-1.828643847	-2.46293461	0.399933269	-4.572372417
LY6E	2404.770296	-1.402767944	-1.616170439	0.311760643	-4.499502983
C1orf54	86.07037922	1.283798204	1.441665644	0.288097032	4.456131311
CHDH	24.5476939	-1.801738098	-2.458350119	0.404394043	-4.455402177
HS3ST2	107.1838257	-1.970981011	-3.139185229	0.44187893	-4.460454834
TBL1X	124.6461316	-1.161558021	-1.273383737	0.26081208	-4.453620478
NRN1	43.99958441	1.881511185	2.72844844	0.423512858	4.442630609
C17orf108	37.39068162	-1.376967639	-1.587753098	0.312574694	-4.405243501
MT1H	184.2869427	1.779044777	2.414060374	0.404093087	4.40256177
CD8A	15.12770548	1.995208416	3.741558114	0.455014713	4.38493165
ST6GALNAC6	77.82829391	-1.301757689	-1.478258946	0.297949737	-4.369051314
VAMP5	528.8158705	1.372778911	1.586096622	0.314150862	4.369807874
ATP6V0D2	157.0498307	-1.725975057	-2.293121208	0.395715284	-4.361658814
CDC42SE2	125.5355136	1.197894456	1.328779582	0.27485791	4.358231704
LRRK2	34.78095582	1.605114042	2.00526104	0.36779484	4.364155954
MT2A	1209.71935	1.536958794	1.871175	0.352499819	4.360169029
MAPRE3	24.91344589	-1.286837298	-1.459961368	0.295712188	-4.351654588
STAT4	16.62665086	1.88170985	2.892851765	0.435755081	4.318274028
CXCL12	37.92469444	1.643980389	2.118244664	0.382030986	4.303264524
KLF8	15.54149381	-1.722778876	-2.332100063	0.400660338	-4.29984881
GDF15	38.94252144	-1.663552019	-2.169859396	0.387574131	-4.292216338
HSD11B1	77.42781838	1.895123333	3.001402798	0.441716252	4.290363608
CDC42BPA	13.6982526	-1.647360004	-2.136337223	0.385401302	-4.274401761
CYGB	31.04467308	-1.964712183	-4.015683942	0.459880306	-4.27222509

PLA2G2D	109.5312785	1.763716448	2.461637789	0.413350236	4.266881437
GPA33	20.074732	-1.797225017	-2.592964504	0.422064886	-4.258172329
FAIM	12.17614835	-1.610419491	-2.064581715	0.37886071	-4.25069016
EMR3	35.63183757	-1.714234096	-2.342124521	0.405693736	-4.22543891
TMEM91	95.2425393	-1.34607504	-1.568863687	0.321264045	-4.189933672
BATF2	31.77379546	1.362084415	1.597428289	0.326218503	4.175374493
NTRK3	10.85255794	-1.916180613	-4.357017511	0.460783586	-4.158526195
TLN2	36.65177328	1.497956833	1.852290677	0.36128621	4.146177718
IL1B	365.4624561	1.530731403	1.919053129	0.369593579	4.141661248
LRFN4	54.42784885	-1.114733582	-1.231747793	0.269706167	-4.133140865
IL21R	117.919771	1.569097085	2.01690221	0.382003964	4.107541372
HAPLN3	104.8505968	1.544536053	1.966579232	0.377064127	4.096215846
USP53	38.42799967	-1.532371517	-1.949601425	0.377029279	-4.06433028
CAPN3	91.897339	-1.484926744	-1.851003011	0.365542729	-4.062252171
EPHB3	16.04321053	1.713150509	2.505226601	0.425123443	4.029771909
MT1G	229.2205578	1.598210025	2.128964957	0.397462612	4.021032365
IL15RA	59.65779829	1.187577887	1.345951251	0.296237846	4.0088662
FABP5	651.1523843	-1.34778259	-1.602750154	0.336712182	-4.002773474
HPCAL1	299.0343808	-1.119391336	-1.247699834	0.279630128	-4.003114207
DPEP2	159.1087223	-1.28478646	-1.497746601	0.321159137	-4.000466783
SPN	864.368385	-1.040935645	-1.141067125	0.26064361	-3.993712512
ME3	14.27058955	-1.665542537	-2.373729552	0.418253806	-3.982133604
SMCR7	20.82517801	-1.493421207	-1.908434374	0.375978283	-3.972094331
LFNG	137.6601483	-1.270725548	-1.479367116	0.320326492	-3.966969886
SDHAP1	11.0307236	-1.352388557	-1.630018644	0.341033727	-3.965556627
KCNQ1	68.43407129	-1.084377198	-1.202619957	0.274183171	-3.954937113
RETN	185.6122813	-1.73801383	-2.705437982	0.439412919	-3.955308901
ZBED3	14.76532506	-1.659357388	-2.39580425	0.419959478	-3.951232142
AGPAT4	120.7876177	-1.04492469	-1.14963983	0.264837777	-3.945527342
FRAT2	80.67946471	-1.067034186	-1.182101602	0.272627639	-3.913888516
MEGF6	13.56411613	-1.40114029	-1.740935712	0.359711719	-3.895175539
AOC3	17.32511481	-1.707562265	-2.764422382	0.441509835	-3.867552049
PRAM1	199.6594122	-1.036969321	-1.144111003	0.268405093	-3.863448748
MMP2	104.4945018	-1.131589482	-1.278722696	0.293487575	-3.855664014
NENF	185.2985659	-1.202599637	-1.386716423	0.312453562	-3.848890796
IGJ	25.03426785	1.71786273	2.842587141	0.447144592	3.841850621
LYPD5	11.76862347	1.431623228	1.801045311	0.372756413	3.840640101
TRAF1	39.07495406	1.179502759	1.353741848	0.307873172	3.831131994
BAHCC1	11.08338558	-1.424306698	-1.813790257	0.372394177	-3.824728702
CSTB	5125.845831	-1.096553251	-1.229957929	0.286513486	-3.82723085
TNFAIP3	549.3857207	1.357195012	1.658290414	0.354818922	3.82503561
B4GALT4	24.08728419	-1.044208888	-1.158562376	0.274408674	-3.805305686
EPB41L4A-AS	107.2574279	-1.036465322	-1.149014178	0.272991289	-3.79669742
NLRP12	28.26961502	-1.211329148	-1.411054079	0.318789652	-3.799775621
RGS16	22.65632699	1.383606081	1.716949494	0.364476374	3.796147515
DNAJB1	1690.007652	1.315862602	1.588047407	0.347011561	3.791984904

Garrido-Martin EM, et al. J Immunother Cancer 2020; 8:e000778. doi: 10.1136/jitc-2020-000778

KPNA2	104.7604481	1.071882838	1.198860744	0.284102682	3.772871234
LOC284837	16.73846423	-1.580344855	-2.278049599	0.418927178	-3.772361735
ADTRP	26.90965128	-1.625210188	-2.483034162	0.434754915	-3.73822154
CFD	314.7313074	-1.218506624	-1.428252055	0.325922461	-3.738639613
TPD52	77.77927497	1.162417828	1.338019199	0.310828783	3.739736768
TCEA3	13.18082252	-1.652160472	-2.638841273	0.442809935	-3.731082661
CFB	53.64032029	1.31677967	1.607309332	0.353567321	3.724268597
SCMH1	41.56829122	-1.260150241	-1.503772579	0.338204441	-3.726001459
ACSS1	212.5864378	-1.087601935	-1.230498619	0.294850197	-3.688659353
PCOLCE2	116.0935723	-1.482794737	-2.007493868	0.403415967	-3.675597546
SPOCK2	36.43365789	1.579793894	2.353902999	0.42952817	3.677975049
EPB41L1	76.72906749	-1.428159977	-1.869424133	0.389551634	-3.666163484
CYP27A1	634.0234076	-1.30791622	-1.603533052	0.3569423	-3.664223093
CHCHD6	61.28845067	-1.553920909	-2.265955139	0.425966879	-3.647985289
RBP7	12.33291279	-1.533830689	-2.207925016	0.421534111	-3.638687001
FXYD6	122.0656734	-1.141653001	-1.318912896	0.314296896	-3.632403041
CORO2A	88.75755228	-1.118281432	-1.28625508	0.309464911	-3.613596869
SLC38A1	14.93963659	1.427307746	1.901610915	0.397634207	3.589499399
ZNF267	53.92606871	1.15361397	1.343766349	0.321637805	3.586686491
SLC9A9	24.61650662	1.094753715	1.251205052	0.30590015	3.578794305
DNAJA4	54.96172049	1.346448282	1.714664564	0.377085903	3.570667247
HIST1H2AG	12.62391438	1.534625542	2.286465597	0.429959031	3.569236673
ITIH5	18.01380904	-1.56050212	-2.430488669	0.437283954	-3.568624247
RGS1	1290.386358	1.213757973	1.450097489	0.340156181	3.568237297
TCTN2	36.97239021	-1.089437612	-1.248258427	0.305691864	-3.563842354
PMFBP1	17.55474313	-1.316757358	-1.659715536	0.369912651	-3.559644025
FARP1	138.7932985	-1.209209704	-1.445885514	0.340426826	-3.552040007
PPAP2B	141.7971196	-1.346442106	-1.71891297	0.378979937	-3.552805766
TFCP2L1	31.78874217	-1.294740835	-1.614020272	0.364962417	-3.547600451
SVIL	75.06386479	-1.182455914	-1.399469772	0.333586426	-3.544676347
BCL11A	36.64326627	-1.026677978	-1.155217445	0.28983202	-3.542320752
SLC5A3	22.25607233	1.347137206	1.725796454	0.381362491	3.532432367
HTRA1	66.06242209	-1.412048326	-1.892837754	0.400244311	-3.527966014
SLC30A1	30.56666234	1.325937924	1.686438193	0.376568622	3.521105712
GIMAP8	107.1657938	1.001748586	1.122338434	0.284945563	3.515578815
TRIM45	13.57986465	-1.243580849	-1.531240991	0.35448948	-3.508089573
CD72	86.94751182	1.043052331	1.182647951	0.297469493	3.506417824
PGM5	12.59704999	-1.54218198	-2.438561388	0.441463372	-3.493340732
SIGLEC8	42.50725379	1.334351034	1.71537547	0.381816187	3.494747155
AGPAT5	17.11981552	1.041876433	1.182593953	0.299409118	3.47977523
IGFBP2	30.50841736	-1.476491621	-2.15509708	0.425588473	-3.469294203
SNX10	339.436605	1.294962281	1.634213353	0.373428261	3.467767212
FABP4	96.0442691	-1.52404285	-2.380617348	0.440023273	-3.46355055
NLRP1	115.6950543	-1.082403175	-1.250673586	0.314068206	-3.446395252
FABP3	324.5236739	-1.174692937	-1.40572001	0.341303295	-3.441786098
HSPA6	40.88002351	1.288178687	1.629873972	0.375057829	3.434613513

DTX4	52.56719135	-1.450558876	-2.093861592	0.422924002	-3.429833419
FAM73B	20.42321467	-1.230126267	-1.515470832	0.358459803	-3.43169933
JAKMIP2	34.04764597	-1.408276105	-1.948705502	0.410604977	-3.429758977
JAK2	28.59247861	1.134460025	1.339278036	0.331424872	3.422977938
YPEL3	89.4986381	-1.002338089	-1.131866517	0.292666849	-3.424843272
TSC22D1	144.8975664	1.221076884	1.497533709	0.357080677	3.419610639
RMI2	13.61250719	1.374464041	1.848886935	0.402772466	3.41250745
SOX13	30.74870767	-1.358773174	-1.822580199	0.398301018	-3.411422802
FLJ41484	12.20979767	-1.307896324	-1.701371983	0.383734557	-3.408336052
KCNJ2	21.07829524	1.391193501	1.91442768	0.408319702	3.407118234
CDA	30.69818753	-1.248509857	-1.556314221	0.367127667	-3.400751206
PI3	10.62057208	1.549756349	2.843644909	0.45697273	3.391354115
LILRA2	73.98162077	-1.055048756	-1.215356963	0.31183313	-3.38337609
BIRC3	35.57862445	1.395495244	1.933844089	0.412630667	3.381947483
CAMK2D	77.02188316	1.089227138	1.270164513	0.322241398	3.380158927
ITPKB	61.44798586	-1.048868815	-1.207874502	0.310275952	-3.380438636
RNF144B	143.1558786	1.044012029	1.199489282	0.309569437	3.372464799
TBC1D10C	21.52038086	-1.260794601	-1.59737483	0.373971333	-3.371366971
PRDM1	176.5352971	1.031400809	1.181449561	0.306447697	3.365666695
C2orf65	22.19151159	-1.138025139	-1.363142296	0.340587051	-3.341363494
CCL17	110.1324763	-1.539850365	-3.269285792	0.460828377	-3.341483386
RTN4R	27.27968072	-1.499282949	-2.494073946	0.448455408	-3.34321523
ATP13A2	156.3152114	1.099032875	1.295044546	0.329747653	3.332951322
LILRA3	112.7163504	1.374592241	1.90802333	0.412497806	3.332362552
AQP3	93.33761251	-1.138097295	-1.362434876	0.341867197	-3.329062582
ALK	13.07262688	-1.458579898	-2.284124122	0.440065333	-3.314462166
ST5	111.4008208	-1.264873851	-1.630377011	0.383662202	-3.296842495
MAZ	312.1177835	-1.009698689	-1.156931892	0.30687219	-3.290290622
CCL8	91.38932758	1.204827915	1.502548037	0.367099592	3.28201922
C5orf20	18.85723214	1.380454234	1.982193557	0.422159922	3.269979363
RPH3AL	15.51679569	-1.18044595	-1.459609736	0.361191643	-3.268198397
MT1E	143.6920815	1.275875463	1.67816484	0.392724255	3.248781933
FNDC5	10.03290764	-1.483089497	-2.727978991	0.457166824	-3.244088197
FAM109A	25.92667614	-1.221773486	-1.558411076	0.377964007	-3.232512787
CLEC4D	11.17575785	1.405265307	2.135775731	0.435649371	3.225679639
MXD1	644.8226231	1.112380195	1.339899727	0.345843137	3.216429866
ROBO3	13.92355387	-1.211520819	-1.537446839	0.377090718	-3.212809968
MTRNR2L1	3112.626686	1.213544475	1.546476247	0.378327743	3.207653939
SAMSN1	291.8312363	1.136243227	1.386598158	0.354215437	3.207774445

Garrido-Martin EM, *et al. J Immunother Cancer* 2020; 8:e000778. doi: 10.1136/jitc-2020-000778

pvalue	padj	MeanDeseq_CXCL9_high	MeanDeseq_CXCL9_low
3.83E-56	5.03E-52	8931.253952	129.9474363
1.17E-34	7.64E-31	362.653433	18.87513211
1.32E-28	5.78E-25	469.0639106	14.27243298
1.25E-25	4.09E-22	887.086958	117.3602467
2.01E-20	5.26E-17	218.1285083	15.28487986
2.55E-16	5.57E-13	1572.883721	243.3785739
4.49E-16	8.42E-13	78.72209506	13.67591364
1.56E-15	2.37E-12	623.3214266	93.01157323
1.63E-15	2.37E-12	1910.566817	482.5146254
2.43E-15	3.19E-12	78.76455671	4.79327975
1.73E-14	2.06E-11	183.417647	8.956364075
2.67E-14	2.92E-11	296.6097972	76.55721744
1.84E-13	1.86E-10	129.3953405	17.26486854
3.81E-13	3.57E-10	1276.682507	170.0370891
3.49E-12	3.06E-09	93.97819487	4.945684436
4.69E-12	3.84E-09	794.2342996	257.2032584
1.32E-11	1.02E-08	86.2313745	7.973781225
2.58E-11	1.88E-08	29.05301136	1.197963918
9.00E-11	6.22E-08	641.1126445	136.6729022
1.18E-10	7.74E-08	171.0414439	25.49346005
2.25E-10	1.40E-07	21.13493106	1.079875634
4.25E-10	2.53E-07	250.1741804	60.34375268
4.72E-10	2.69E-07	408.3997937	57.18830988
4.09E-09	2.15E-06	2.164659735	27.20568014
4.37E-09	2.21E-06	140.2466297	28.28805057
5.55E-09	2.63E-06	533.6056298	147.186586
5.62E-09	2.63E-06	223.1041636	64.43696858
7.40E-09	3.35E-06	559.8334827	159.2777837
1.04E-08	4.57E-06	473.6987247	2637.305024
2.80E-08	1.18E-05	16.65635457	60.91643656
3.46E-08	1.42E-05	9.705280855	84.0346252
4.05E-08	1.56E-05	2.37134521	23.53526015
4.03E-08	1.56E-05	2452.173241	643.6401289
4.25E-08	1.59E-05	32.39768733	1.813304783
4.58E-08	1.67E-05	39.18497155	15.45696904
4.81E-08	1.71E-05	790.070783	176.1970815
6.42E-08	2.22E-05	34.43932016	5.61668341
7.50E-08	2.52E-05	1548.739024	565.5251216
1.74E-07	5.72E-05	861.5967427	349.7904694
1.82E-07	5.83E-05	23.01448799	128.0887811
2.23E-07	6.93E-05	22.15932748	1.522678996
2.27E-07	6.93E-05	539.3828977	103.4391181
2.47E-07	7.37E-05	17.83556102	133.842376
3.56E-07	0.00010145	75.21458257	20.00102635
4.41E-07	0.000123005	1098.490454	472.0867588

Garrido-Martin EM, *et al. J Immunother Cancer* 2020; 8:e000778. doi: 10.1136/jitc-2020-000778

5.09E-07	0.000139138	56.0742846	1.41388568
6.53E-07	0.000171122	441.0847306	118.1193525
6.65E-07	0.000171122	205.9755019	84.64054682
6.83E-07	0.000172271	137.6423802	19.33218546
7.10E-07	0.000175736	364.5785107	1571.702065
8.88E-07	0.000215721	200.4272978	48.28051918
1.11E-06	0.000255755	81.07839357	28.69986334
1.10E-06	0.000255755	448.3653085	116.3395745
1.17E-06	0.000264024	419.5517616	75.11301688
1.19E-06	0.000264517	301.5347805	89.8054913
1.54E-06	0.000335825	146.570295	28.51152268
1.75E-06	0.000377248	8.431428906	75.73844144
1.84E-06	0.000389216	22.53742458	1.942993904
2.01E-06	0.000417545	7.154944651	29.92229029
2.15E-06	0.00043974	21.56891439	74.79003518
2.46E-06	0.000496807	605.2063304	74.60585285
2.64E-06	0.00051944	157.6140814	666.6710615
2.65E-06	0.00051944	341.212712	107.5863369
2.93E-06	0.000565737	79.55438517	19.25533485
3.05E-06	0.000579922	1367.546097	633.9038677
3.91E-06	0.000722917	101.4506293	17.91629366
4.49E-06	0.000817034	267.5423658	82.3400184
4.82E-06	0.000866368	16.33662231	88.8977497
6.81E-06	0.001207156	1190.130734	3625.089967
8.35E-06	0.001401716	126.3231439	46.74310943
8.37E-06	0.001401716	7.713486096	41.40582259
8.18E-06	0.001401716	22.15008908	192.5431818
8.44E-06	0.001401716	73.08825596	177.4002464
8.89E-06	0.001438856	76.71924611	11.61659077
1.06E-05	0.001689998	18.51121445	56.5835373
1.07E-05	0.001690415	311.1260831	58.69650968
1.16E-05	0.001811466	27.94264646	2.425445129
1.25E-05	0.001903009	41.49817759	114.371628
1.24E-05	0.001903009	795.5673296	264.8290894
1.29E-05	0.001910674	54.0988692	260.02061
1.31E-05	0.001910674	179.7489578	71.9692963
1.28E-05	0.001910674	55.83149502	13.92882861
1.30E-05	0.001910674	1905.63352	521.849677
1.35E-05	0.001947275	13.66390629	36.32354584
1.57E-05	0.002241726	29.35115683	3.989981084
1.68E-05	0.002373388	61.85174854	14.27897391
1.71E-05	0.002384623	5.232447646	26.04430978
1.77E-05	0.002436942	14.35202272	63.55345446
1.78E-05	0.002436942	138.1289149	17.28685068
1.92E-05	0.002589973	5.073712166	22.39830902
1.94E-05	0.002589973	3.702262088	58.37593472

1.98E-05	0.002625973	187.1868766	33.75703547
2.06E-05	0.002676305	5.730280819	34.51225258
2.13E-05	0.002713567	4.682607852	19.81605945
2.38E-05	0.002978673	11.83423157	59.74160009
2.79E-05	0.003420147	48.14503969	142.5606043
2.97E-05	0.003612651	47.90118691	15.80469036
3.20E-05	0.003818939	1.123732583	20.50737678
3.38E-05	0.003994421	57.72186331	15.92327108
3.45E-05	0.004037539	579.2322509	153.6649518
3.58E-05	0.004116721	32.73840297	76.34639128
4.00E-05	0.004521206	189.9715865	47.22601005
4.20E-05	0.004707482	167.3483093	43.1335003
4.82E-05	0.005353874	15.67126699	61.77172246
4.86E-05	0.005356379	40.55261462	143.8609419
5.58E-05	0.006001833	27.42636359	4.851117394
5.79E-05	0.006128475	374.16049	85.88002774
6.10E-05	0.006401271	86.28139903	33.57960081
6.26E-05	0.006464997	324.3770739	977.5728946
6.25E-05	0.006464997	178.5542572	421.3580102
6.32E-05	0.006477341	84.11032787	234.5295164
6.50E-05	0.006613084	542.6797495	1193.444903
6.83E-05	0.006890355	4.691142907	23.90014536
7.12E-05	0.007078473	9.138286735	32.55599116
7.28E-05	0.007166747	73.0229619	203.1865367
7.32E-05	0.007166747	5.816513472	16.31065712
7.66E-05	0.007266625	41.51847657	95.67093787
7.64E-05	0.007266625	49.44628869	322.4800456
7.77E-05	0.007283496	4.970093069	24.62603187
7.96E-05	0.007406212	75.94137236	166.9102258
9.08E-05	0.008388204	49.48186273	112.292455
9.81E-05	0.008999608	6.879913443	20.41768124
0.000109933	0.009943284	5.039564106	29.74729274
0.000111797	0.010042619	124.6338628	275.5420644
0.000115416	0.010297141	61.69607997	147.6943995
0.000118654	0.010514498	102.9200886	267.4325959
0.00012211	0.010658232	489.1089394	6.364289681
0.000122714	0.010658232	18.39058641	5.216721631
0.000127555	0.010862886	56.43732083	22.0257548
0.000130916	0.010868443	5.257462403	16.98011581
0.000129593	0.010868443	3079.406187	7184.056136
0.000130753	0.010868443	837.1290634	266.2930779
0.000141629	0.011465843	14.84967457	33.46506985
0.000146637	0.011511383	67.48085476	147.3413613
0.000144827	0.011511383	15.78482415	41.04643251
0.000146962	0.011511383	34.71162527	10.77392638
0.000149448	0.011597695	2544.483018	846.8397632

Garrido-Martin EM, *et al. J Immunother Cancer* 2020; 8:e000778. doi: 10.1136/jitc-2020-000778

0.00016138	0.012402463	146.4304118	63.86089727
0.00016171	0.012402463	6.124263629	27.59638533
0.000185327	0.013731969	8.516591832	45.53421974
0.000185019	0.013731969	171.3941014	458.3649208
0.000184213	0.013731969	111.9038974	44.2841096
0.000190659	0.013969208	3.665350067	22.76930208
0.000195882	0.014038231	80.78070516	26.92339291
0.000194541	0.014038231	21.83526645	61.67012955
0.000225439	0.015857575	128.2089245	298.4768308
0.000237293	0.016379486	47.01260964	185.7974747
0.000235093	0.016379486	61.20072553	12.11190206
0.000246217	0.016818386	33.74272631	120.2957116
0.00024809	0.016858583	316.1372207	952.4672428
0.000264305	0.01777619	21.15807689	101.1828755
0.000274032	0.018243272	4.335957641	20.31710693
0.000280794	0.018599063	69.83490008	174.3838286
0.000301978	0.019802238	52.58648208	125.6780025
0.000331314	0.021195989	23.64276486	17.86603792
0.000334907	0.021321843	77.54053226	30.43773834
0.000345183	0.021869928	34.88062187	14.58644567
0.000356073	0.02220686	84.07730228	26.16153948
0.000358023	0.02220686	21.06314279	65.07590846
0.000358861	0.02220686	6.056773802	30.15704382
0.000359391	0.02220686	1899.062693	696.6974957
0.000365465	0.02239757	22.00917368	52.24821499
0.000371358	0.022652828	8.653851554	26.60110088
0.000382257	0.023102752	75.30622952	203.1602286
0.000381146	0.023102752	66.44182138	217.5135555
0.000388757	0.023387864	16.18111315	47.93255351
0.000393095	0.023540853	41.39999856	109.6931973
0.000396623	0.023644132	22.5095697	51.05323649
0.000411756	0.024325107	34.41187204	10.30526631
0.000418766	0.024454319	28.11915801	105.1121494
0.000429751	0.024699831	46.66618483	14.66640003
0.000438796	0.024810321	147.2047532	68.24050409
0.000451337	0.025404659	7.388303335	19.92961948
0.000454182	0.025455523	121.657761	53.03578418
0.000477017	0.026176082	3.977620347	21.29224477
0.000474511	0.026176082	65.84734925	19.79928727
0.000501835	0.027196533	24.07754606	10.27493665
0.000521828	0.028163663	11.43839543	49.83951959
0.000524802	0.028208091	514.6876492	165.9713542
0.000533096	0.028420974	31.54243544	161.0706729
0.000568119	0.02980351	68.8488098	163.9911292
0.000577887	0.030195172	178.8295173	471.6846153
0.000593399	0.030882676	62.06009454	20.07462523

0.000603952	0.030982114	20.46449851	84.70269217
0.000599812	0.030982114	10.44927026	30.55182391
0.000604118	0.030982114	14.24484439	54.05539947
0.000619391	0.031295175	41.18519397	16.27713103
0.000615154	0.031295175	56.53404882	123.2123819
0.000627108	0.031511589	214.5752098	76.05847167
0.000643682	0.032220936	21.54929745	5.819827624
0.000646248	0.032226396	14.13067493	47.51351066
0.000653603	0.032469733	6.094632269	18.38875371
0.000656527	0.032491883	33.32074603	9.035659916
0.00067201	0.033009011	15.36743926	46.01868015
0.000695482	0.03321626	18.67935288	2.568264425
0.000716005	0.033900385	44.35311707	104.0076924
0.000719739	0.033932218	56.55444463	14.63326713
0.000724439	0.033932218	109.4626683	45.20938528
0.000723702	0.033932218	37.71619419	85.58458021
0.000744986	0.034403143	200.3665423	87.53627815
0.000747962	0.034419352	10.55191564	32.63630164
0.000763589	0.035015635	245.8508088	109.1128949
0.00083368	0.036690313	12.65574921	32.01060265
0.00083332	0.036690313	20.71272823	199.2499568
0.000828136	0.036690313	8.465151118	46.02210539
0.0008593	0.037520226	222.8118673	90.72425698
0.00086112	0.037520226	178.4844601	47.5185724
0.000871388	0.037726906	52.38998748	135.3684824
0.000918196	0.039535524	4.460042808	21.6878275
0.000977783	0.041500409	54.79371165	168.6547309
0.00100084	0.042070547	194.4370486	430.7406562
0.001030666	0.042699909	135.1770314	48.00007521
0.001075553	0.04367677	30.41247174	7.554368973
0.001082345	0.04367677	8.306034063	22.82054444
0.001159003	0.04577228	219.6864467	68.85943166
0.001178273	0.045991204	2.602686162	17.43944975
0.001227066	0.047193479	13.23657321	38.73787146
0.001256739	0.048052879	18.20290273	4.148035674
0.001297963	0.048916031	926.3109425	366.8499935
0.001314432	0.049253655	6.999002758	21.08528689
0.001338224	0.049860257	12207.32039	4131.402345
0.001337664	0.049860257	424.2267501	162.033007

© 2000-2018 QIAGEN. All rights reserved.

Ingenuity Canonical Pathways	-log(p-value)	Ratio
Pathogenesis of Multiple Sclerosis	5.96E+00	4.44E-01
Agranulocyte Adhesion and Diapedesis	5.45E+00	5.70E-02
Granulocyte Adhesion and Diapedesis	4.89E+00	5.52E-02
Th1 and Th2 Activation Pathway	4.03E+00	4.86E-02
Neuroinflammation Signaling Pathway	3.56E+00	3.54E-02
Th1 Pathway	3.41E+00	5.19E-02
Interferon Signaling	3.38E+00	1.11E-01
Type I Diabetes Mellitus Signaling	3.09E+00	5.41E-02
Inflammasome pathway	3.02E+00	1.50E-01
Role of JAK1 and JAK3 in Œ≥c Cytokine Signaling	3.01E+00	6.49E-02
IL-17A Signaling in Gastric Cells	2.74E+00	1.20E-01
IL-15 Production	2.59E+00	1.07E-01
Superoxide Radicals Degradation	2.59E+00	2.50E-01
TNFR2 Signaling	2.50E+00	1.00E-01
T Helper Cell Differentiation	2.23E+00	5.48E-02
Toll-like Receptor Signaling	2.17E+00	5.26E-02
Oncostatin M Signaling	2.15E+00	7.50E-02
Role of MAPK Signaling in the Pathogenesis of Influenza	2.13E+00	5.13E-02
Acute Phase Response Signaling	2.09E+00	3.41E-02
Role of Hypercytokinemia/hyperchemokinemia in the Pathogenesis o	2.06E+00	6.98E-02
iNOS Signaling	2.01E+00	6.67E-02
Triacylglycerol Biosynthesis	1.98E+00	6.52E-02
Crosstalk between Dendritic Cells and Natural Killer Cells	1.94E+00	4.49E-02
Primary Immunodeficiency Signaling	1.91E+00	6.12E-02
Differential Regulation of Cytokine Production in Macrophages and T	1.88E+00	1.11E-01
Communication between Innate and Adaptive Immune Cells	1.84E+00	4.21E-02
Protein Ubiquitination Pathway	1.79E+00	2.64E-02
Th2 Pathway	1.78E+00	3.33E-02
STAT3 Pathway	1.72E+00	3.88E-02
Choline Degradation I	1.71E+00	5.00E-01
Differential Regulation of Cytokine Production in Intestinal Epithelial (	1.67E+00	8.70E-02
Role of JAK1, JAK2 and TYK2 in Interferon Signaling	1.64E+00	8.33E-02
CDP-diacylglycerol Biosynthesis I	1.64E+00	8.33E-02
Role of JAK family kinases in IL-6-type Cytokine Signaling	1.60E+00	8.00E-02
Ethanol Degradation IV	1.60E+00	8.00E-02
Phosphatidylglycerol Biosynthesis II (Non-plastidic)	1.57E+00	7.69E-02
Eicosanoid Signaling	1.55E+00	4.48E-02
Tetrahydrobiopterin Biosynthesis I	1.53E+00	3.33E-01
Tetrahydrobiopterin Biosynthesis II	1.53E+00	3.33E-01
Neuroprotective Role of THOP1 in Alzheimer's Disease	1.51E+00	3.33E-02
Atherosclerosis Signaling	1.43E+00	3.15E-02
TREM1 Signaling	1.42E+00	4.00E-02
Hepatic Fibrosis / Hepatic Stellate Cell Activation	1.42E+00	2.67E-02

Retinoate Biosynthesis II	1.41E+00	2.50E-01
Phenylethylamine Degradation I	1.41E+00	2.50E-01
Acetate Conversion to Acetyl-CoA	1.41E+00	2.50E-01
Cytotoxic T Lymphocyte-mediated Apoptosis of Target Cells	1.40E+00	6.25E-02
4-1BB Signaling in T Lymphocytes	1.40E+00	6.25E-02
Role of Macrophages, Fibroblasts and Endothelial Cells in Rheumatoi	1.40E+00	2.19E-02
Chemokine Signaling	1.39E+00	3.90E-02
VDR/RXR Activation	1.38E+00	3.85E-02
GM-CSF Signaling	1.37E+00	3.80E-02
Dendritic Cell Maturation	1.36E+00	2.58E-02
Role of JAK2 in Hormone-like Cytokine Signaling	1.36E+00	5.88E-02
TWEAK Signaling	1.33E+00	5.71E-02
Iron homeostasis signaling pathway	1.33E+00	2.92E-02
Complement System	1.29E+00	5.41E-02
Notch Signaling	1.27E+00	5.26E-02
Inhibition of Matrix Metalloproteases	1.25E+00	5.13E-02
Prolactin Signaling	1.24E+00	3.37E-02
JAK/Stat Signaling	1.24E+00	3.37E-02
IL-17 Signaling	1.22E+00	3.30E-02
Role of PKR in Interferon Induction and Antiviral Response	1.21E+00	4.88E-02
LPS/IL-1 Mediated Inhibition of RXR Function	1.16E+00	2.25E-02
CTLA4 Signaling in Cytotoxic T Lymphocytes	1.13E+00	3.03E-02
Thyroid Cancer Signaling	1.12E+00	4.35E-02
Role of IL-17F in Allergic Inflammatory Airway Diseases	1.12E+00	4.35E-02
Airway Pathology in Chronic Obstructive Pulmonary Disease	1.12E+00	1.25E-01
Tryptophan Degradation to 2-amino-3-carboxymuconate Semialdehy	1.12E+00	1.25E-01
Salvage Pathways of Pyrimidine Deoxyribonucleotides	1.12E+00	1.25E-01
nNOS Signaling in Neurons	1.11E+00	4.26E-02
Graft-versus-Host Disease Signaling	1.09E+00	4.17E-02
Hematopoiesis from Pluripotent Stem Cells	1.09E+00	4.17E-02
TNFR1 Signaling	1.06E+00	4.00E-02
Tec Kinase Signaling	1.06E+00	2.35E-02
Antioxidant Action of Vitamin C	1.04E+00	2.78E-02
GDP-glucose Biosynthesis	1.03E+00	1.00E-01
Amyotrophic Lateral Sclerosis Signaling	1.01E+00	2.70E-02
Role of Cytokines in Mediating Communication between Immune Cell	1.00E+00	3.70E-02
Ephrin Receptor Signaling	9.97E-01	2.23E-02
Huntington's Disease Signaling	9.94E-01	2.00E-02
Glucose and Glucose-1-phosphate Degradation	9.87E-01	9.09E-02
Unfolded protein response	9.78E-01	3.57E-02
Glutamate Receptor Signaling	9.66E-01	3.51E-02
p38 MAPK Signaling	9.39E-01	2.50E-02
Leukotriene Biosynthesis	9.19E-01	7.69E-02
Glycogen Degradation II	9.19E-01	7.69E-02
Bile Acid Biosynthesis, Neutral Pathway	9.19E-01	7.69E-02
Induction of Apoptosis by HIV1	9.17E-01	3.28E-02

iCOS-iCOSL Signaling in T Helper Cells	9.16E-01	2.44E-02
Retinoic acid Mediated Apoptosis Signaling	9.05E-01	3.23E-02
Phospholipases	9.05E-01	3.23E-02
Glucocorticoid Receptor Signaling	8.99E-01	1.74E-02
Regulation of Cellular Mechanics by Calpain Protease	8.94E-01	3.17E-02
Sperm Motility	8.86E-01	2.36E-02
Renin-Angiotensin Signaling	8.79E-01	2.34E-02
NAD biosynthesis II (from tryptophan)	8.61E-01	6.67E-02
Glycogen Degradation III	8.61E-01	6.67E-02
Granzyme B Signaling	8.35E-01	6.25E-02
Remodeling of Epithelial Adherens Junctions	8.31E-01	2.90E-02
CNTF Signaling	8.21E-01	2.86E-02
Thrombopoietin Signaling	8.11E-01	2.82E-02
RAN Signaling	8.10E-01	5.88E-02
Leukocyte Extravasation Signaling	8.09E-01	1.90E-02
Ephrin B Signaling	7.92E-01	2.74E-02
1D-myo-inositol Hexakisphosphate Biosynthesis II (Mammalian)	7.66E-01	5.26E-02
D-myo-inositol (1,3,4)-trisphosphate Biosynthesis	7.66E-01	5.26E-02
IL-12 Signaling and Production in Macrophages	7.59E-01	2.05E-02
Granzyme A Signaling	7.46E-01	5.00E-02
The Visual Cycle	7.46E-01	5.00E-02
CD40 Signaling	7.39E-01	2.53E-02
Oxidative Ethanol Degradation III	7.27E-01	4.76E-02
IL-15 Signaling	7.14E-01	2.44E-02
Polyamine Regulation in Colon Cancer	7.09E-01	4.55E-02
Growth Hormone Signaling	6.91E-01	2.35E-02
Leptin Signaling in Obesity	6.83E-01	2.33E-02
IL-22 Signaling	6.75E-01	4.17E-02
Tumoricidal Function of Hepatic Natural Killer Cells	6.75E-01	4.17E-02
Superpathway of D-myo-inositol (1,4,5)-trisphosphate Metabolism	6.75E-01	4.17E-02
IL-3 Signaling	6.61E-01	2.25E-02
Tryptophan Degradation III (Eukaryotic)	6.59E-01	4.00E-02
Dopamine-DARPP32 Feedback in cAMP Signaling	6.59E-01	1.83E-02
Opioid Signaling Pathway	6.45E-01	1.62E-02
Lipid Antigen Presentation by CD1	6.44E-01	3.85E-02
Gluconeogenesis I	6.44E-01	3.85E-02
Aldosterone Signaling in Epithelial Cells	6.39E-01	1.79E-02
FLT3 Signaling in Hematopoietic Progenitor Cells	6.33E-01	2.15E-02
Death Receptor Signaling	6.33E-01	2.15E-02
Bladder Cancer Signaling	6.26E-01	2.13E-02
CCR5 Signaling in Macrophages	6.20E-01	2.11E-02
GABA Receptor Signaling	6.20E-01	2.11E-02
Colorectal Cancer Metastasis Signaling	6.18E-01	1.57E-02
Apoptosis Signaling	6.13E-01	2.08E-02
PDGF Signaling	6.13E-01	2.08E-02
G Protein Signaling Mediated by Tubby	5.66E-01	3.12E-02

FAK Signaling	5.58E-01	1.90E-02
NF-ŒʃB Signaling	5.53E-01	1.60E-02
Circadian Rhythm Signaling	5.44E-01	2.94E-02
Retinoate Biosynthesis I	5.44E-01	2.94E-02
MIF-mediated Glucocorticoid Regulation	5.34E-01	2.86E-02
Axonal Guidance Signaling	5.30E-01	1.31E-02
Production of Nitric Oxide and Reactive Oxygen Species in Macrophag	5.25E-01	1.55E-02
B Cell Development	5.23E-01	2.78E-02
Regulation of the Epithelial-Mesenchymal Transition Pathway	5.21E-01	1.54E-02
IGF-1 Signaling	5.20E-01	1.79E-02
Ethanol Degradation II	5.13E-01	2.70E-02
NRF2-mediated Oxidative Stress Response	5.06E-01	1.51E-02
Neuropathic Pain Signaling In Dorsal Horn Neurons	5.05E-01	1.74E-02
T Cell Receptor Signaling	5.05E-01	1.74E-02
Antigen Presentation Pathway	5.04E-01	2.63E-02
April Mediated Signaling	4.95E-01	2.56E-02
tRNA Charging	4.95E-01	2.56E-02
ERK/MAPK Signaling	4.88E-01	1.47E-02
Calcium Signaling	4.81E-01	1.46E-02
Pancreatic Adenocarcinoma Signaling	4.80E-01	1.67E-02
B Cell Activating Factor Signaling	4.77E-01	2.44E-02
nNOS Signaling in Skeletal Muscle Cells	4.77E-01	2.44E-02
tRNA Splicing	4.68E-01	2.38E-02
Retinol Biosynthesis	4.68E-01	2.38E-02
HIF1Œ± Signaling	4.62E-01	1.61E-02
MIF Regulation of Innate Immunity	4.60E-01	2.33E-02
Serotonin Receptor Signaling	4.60E-01	2.33E-02
Osteoarthritis Pathway	4.60E-01	1.42E-02
Synaptic Long Term Potentiation	4.53E-01	1.59E-02
FXR/RXR Activation	4.53E-01	1.59E-02
IL-9 Signaling	4.44E-01	2.22E-02
Dermatan Sulfate Biosynthesis (Late Stages)	4.37E-01	2.17E-02
Role of Tissue Factor in Cancer	4.36E-01	1.54E-02
PI3K/AKT Signaling	4.32E-01	1.53E-02
Autoimmune Thyroid Disease Signaling	4.22E-01	2.08E-02
IL-6 Signaling	4.20E-01	1.49E-02
Chondroitin Sulfate Biosynthesis (Late Stages)	4.15E-01	2.04E-02
Role of Pattern Recognition Receptors in Recognition of Bacteria and	4.08E-01	1.46E-02
Amyloid Processing	4.02E-01	1.96E-02
Docosahexaenoic Acid (DHA) Signaling	3.95E-01	1.92E-02
Systemic Lupus Erythematosus Signaling	3.95E-01	1.29E-02
Phagosome Maturation	3.68E-01	1.35E-02
Chondroitin Sulfate Biosynthesis	3.65E-01	1.75E-02
Nur77 Signaling in T Lymphocytes	3.54E-01	1.69E-02
Dermatan Sulfate Biosynthesis	3.54E-01	1.69E-02
Activation of IRF by Cytosolic Pattern Recognition Receptors	3.33E-01	1.59E-02

Hepatic Cholestasis	3.30E-01	1.25E-02
Calcium-induced T Lymphocyte Apoptosis	3.19E-01	1.52E-02
PKCŒ∏ Signaling in T Lymphocytes	3.15E-01	1.21E-02
Lymphotoxin Œ≤ Receptor Signaling	3.14E-01	1.49E-02
EGF Signaling	3.10E-01	1.47E-02
Cdc42 Signaling	3.09E-01	1.20E-02
IL-10 Signaling	3.05E-01	1.45E-02
Role of IL-17A in Arthritis	3.05E-01	1.45E-02
IL-2 Signaling	3.01E-01	1.43E-02
Mitochondrial Dysfunction	2.98E-01	1.17E-02
GNRH Signaling	2.98E-01	1.17E-02
eNOS Signaling	2.96E-01	1.16E-02
Wnt/Œ≤-catenin Signaling	2.96E-01	1.16E-02
Melatonin Signaling	2.93E-01	1.39E-02
MSP-RON Signaling Pathway	2.93E-01	1.39E-02
G-Protein Coupled Receptor Signaling	2.79E-01	1.06E-02
Glioma Invasiveness Signaling	2.77E-01	1.32E-02
Heparan Sulfate Biosynthesis (Late Stages)	2.77E-01	1.32E-02
Synaptic Long Term Depression	2.75E-01	1.11E-02
Dopamine Receptor Signaling	2.73E-01	1.30E-02
IL-17A Signaling in Airway Cells	2.69E-01	1.28E-02
Role of PI3K/AKT Signaling in the Pathogenesis of Influenza	2.65E-01	1.27E-02
Role of BRCA1 in DNA Damage Response	2.62E-01	1.25E-02
PPARŒ±/RXRŒ± Activation	2.61E-01	1.08E-02
Protein Kinase A Signaling	2.53E-01	9.98E-03
Xenobiotic Metabolism Signaling	2.52E-01	1.01E-02
RAR Activation	2.52E-01	1.05E-02
Heparan Sulfate Biosynthesis	2.51E-01	1.20E-02
Neurotrophin/TRK Signaling	2.51E-01	1.20E-02
Small Cell Lung Cancer Signaling	2.45E-01	1.18E-02
Allograft Rejection Signaling	2.45E-01	1.18E-02
Regulation of IL-2 Expression in Activated and Anergic T Lymphocytes	2.42E-01	1.16E-02
Endothelin-1 Signaling	2.39E-01	1.02E-02
Erythropoietin Signaling	2.38E-01	1.15E-02
Renal Cell Carcinoma Signaling	2.35E-01	1.14E-02
Adrenomedullin signaling pathway	2.31E-01	1.00E-02
Altered T Cell and B Cell Signaling in Rheumatoid Arthritis	2.29E-01	1.11E-02
OX40 Signaling Pathway	2.26E-01	1.10E-02
IL-7 Signaling Pathway	2.26E-01	1.10E-02
IL-8 Signaling	2.25E-01	9.85E-03
FcŒ≥ Receptor-mediated Phagocytosis in Macrophages and Monocyt	2.20E-01	1.08E-02
PEDF Signaling	2.20E-01	1.08E-02
HER-2 Signaling in Breast Cancer	2.18E-01	1.06E-02
VEGF Family Ligand-Receptor Interactions	2.18E-01	1.06E-02
IL-4 Signaling	2.15E-01	1.05E-02
Salvage Pathways of Pyrimidine Ribonucleotides	2.09E-01	1.03E-02

PPAR Signaling	1.99E-01	9.90E-03
Fc Epsilon RI Signaling	0.00E+00	8.00E-03
Actin Cytoskeleton Signaling	0.00E+00	4.29E-03
Aryl Hydrocarbon Receptor Signaling	0.00E+00	7.09E-03
LXR/RXR Activation	0.00E+00	8.26E-03
Clathrin-mediated Endocytosis Signaling	0.00E+00	9.66E-03
Role of NFAT in Regulation of the Immune Response	0.00E+00	5.21E-03
CXCR4 Signaling	0.00E+00	5.85E-03
CCR3 Signaling in Eosinophils	0.00E+00	7.35E-03
CD28 Signaling in T Helper Cells	0.00E+00	7.58E-03
Virus Entry via Endocytic Pathways	0.00E+00	8.62E-03
Cellular Effects of Sildenafil (Viagra)	0.00E+00	7.63E-03
Relaxin Signaling	0.00E+00	6.33E-03
Thrombin Signaling	0.00E+00	4.76E-03
Molecular Mechanisms of Cancer	0.00E+00	7.61E-03
HMGB1 Signaling	0.00E+00	7.19E-03
Cholecystokinin/Gastrin-mediated Signaling	0.00E+00	9.35E-03
Human Embryonic Stem Cell Pluripotency	0.00E+00	6.99E-03
Androgen Signaling	0.00E+00	7.30E-03
Role of NANOG in Mammalian Embryonic Stem Cell Pluripotency	0.00E+00	7.81E-03
CREB Signaling in Neurons	0.00E+00	4.59E-03
Glioma Signaling	0.00E+00	8.33E-03
EIF2 Signaling	0.00E+00	4.41E-03
PAK Signaling	0.00E+00	9.35E-03
Hereditary Breast Cancer Signaling	0.00E+00	6.67E-03
Role of Osteoblasts, Osteoclasts and Chondrocytes in Rheumatoid Art	0.00E+00	8.58E-03
Phospholipase C Signaling	0.00E+00	8.20E-03
Ovarian Cancer Signaling	0.00E+00	6.67E-03
Role of NFAT in Cardiac Hypertrophy	0.00E+00	4.44E-03
Breast Cancer Regulation by Stathmin1	0.00E+00	4.74E-03
RANK Signaling in Osteoclasts	0.00E+00	9.80E-03
PI3K Signaling in B Lymphocytes	0.00E+00	7.35E-03
Paxillin Signaling	0.00E+00	8.40E-03
Telomerase Signaling	0.00E+00	8.55E-03
Mouse Embryonic Stem Cell Pluripotency	0.00E+00	8.93E-03
D-myo-inositol-5-phosphate Metabolism	0.00E+00	6.17E-03
D-myo-inositol (1,4,5,6)-Tetrakisphosphate Biosynthesis	0.00E+00	6.94E-03
Superpathway of Inositol Phosphate Compounds	0.00E+00	8.47E-03
D-myo-inositol (3,4,5,6)-tetrakisphosphate Biosynthesis	0.00E+00	6.94E-03
3-phosphoinositide Degradation	0.00E+00	6.33E-03
3-phosphoinositide Biosynthesis	0.00E+00	4.98E-03
GŒ±q Signaling	0.00E+00	6.21E-03
UVA-Induced MAPK Signaling	0.00E+00	8.93E-03
Adipogenesis pathway	0.00E+00	7.46E-03
PTEN Signaling	0.00E+00	8.00E-03
Cardiac Œ≤-adrenergic Signaling	0.00E+00	7.09E-03

B Cell Receptor Signaling	0.00E+00	5.15E-03
Insulin Receptor Signaling	0.00E+00	6.80E-03
Integrin Signaling	0.00E+00	9.13E-03
cAMP-mediated signaling	0.00E+00	8.77E-03
Gustation Pathway	0.00E+00	6.49E-03
Sirtuin Signaling Pathway	0.00E+00	3.42E-03

Confidential: For Review Only

Garrido-Martin EM, *et al. J Immunother Cancer* 2020; 8:e000778. doi: 10.1136/jitc-2020-000778

z-score	Molecules
NaN	CXCL10,CXCL11,CCL5,CXCL9
NaN	AOC3,CXCL10,CXCL11,CCL8,CXCL12,CCL17,IL1B,MMP2,CCL5,CXCL9,MMP25
NaN	CXCL10,CXCL11,CCL8,CXCL12,CCL17,IL1B,MMP2,CCL5,CXCL9,MMP25
NaN	STAT4,IL17RB,CD3E,CD274,IL2RA,JAK2,STAT1,CD8A,IRF1
2.111	CXCL10,BIRC7,SOD2,PLA2G2D,CXCL12,IL1B,JAK2,CCL5,STAT1,BIRC3,GRIN3A
1.633	STAT4,CD3E,CD274,JAK2,STAT1,CD8A,IRF1
2	JAK2,STAT1,TAP1,IRF1
2.449	CD3E,GZMB,IL1B,JAK2,STAT1,IRF1
NaN	NLRP1,AIM2,IL1B
NaN	IL15RA,IL21R,IL2RA,JAK2,STAT1
NaN	CXCL10,CXCL11,CCL5
NaN	JAK2,STAT1,IRF1
NaN	SOD2,CYGB
NaN	TNFAIP3,BIRC3,TRAF1
NaN	STAT4,IL21R,IL2RA,STAT1
NaN	UBD,TNFAIP3,IL1B,TRAF1
NaN	MT2A,JAK2,STAT1
NaN	CXCL10,PLA2G2D,RARRES3,CCL5
2.236	RBP7,SOD2,CFB,IL1B,CP,JAK2
NaN	CXCL10,IL1B,CCL5
NaN	JAK2,STAT1,IRF1
NaN	AGPAT4,AGPAT5,PLPP3
NaN	IL15RA,TLN2,CAMK2D,TNFSF10
NaN	CD3E,CD8A,TAP1
NaN	IL1B,CCL5
NaN	CXCL10,IL1B,CCL5,CD8A
NaN	UBD,USP53,HSPH1,HSPA6,DNAJB1,BIRC3,TAP1
0.447	STAT4,IL17RB,CD3E,IL2RA,JAK2
0	IL17RB,NTRK3,IL1B,JAK2
NaN	CHDH
NaN	IL1B,CCL5
NaN	JAK2,STAT1
NaN	AGPAT4,AGPAT5
NaN	JAK2,STAT1
NaN	ACSS1,CYGB
NaN	AGPAT4,AGPAT5
NaN	PLA2G2D,RARRES3,DPEP2
NaN	GCH1
NaN	GCH1
0	GZMA,CFD,GZMB,HTRA1
NaN	PLA2G2D,CXCL12,RARRES3,IL1B
NaN	NLRP12,IL1B,JAK2
NaN	IL1B,MMP2,CCL5,STAT1,CXCL9

Supplemental material
BMJ Publishing Group Limited (BMJ) disclaims all liability and responsibility arising from any reliance placed on this supplemental material which has been supplied by the author(s)

NaN	RBP7
NaN	AOC3
NaN	ACSS1
NaN	CD3E,GZMB
NaN	TNFRSF9,TRAF1
NaN	CAMK2D,IL32,CXCL12,IL1B,JAK2,CCL5,TRAF1
NaN	CAMK2D,CXCL12,CCL5
NaN	CXCL10,MXD1,CCL5
NaN	CAMK2D,JAK2,STAT1
2.236	STAT4,IL32,IL1B,JAK2,STAT1
NaN	JAK2,STAT1
NaN	BIRC3,TRAF1
NaN	GDF15,CP,ATP6V0D2,JAK2
NaN	CFD,CFB
NaN	DTX4,LFNG
NaN	MMP2,MMP25
NaN	JAK2,STAT1,IRF1
NaN	STAT4,JAK2,STAT1
NaN	CXCL10,CXCL11,JAK2
NaN	STAT1,IRF1
NaN	HS3ST2,FABP4,IL1B,FABP5,FABP3
NaN	CD3E,JAK2,CD8A
NaN	NTRK3,CXCL12
NaN	CXCL10,IL1B
NaN	MMP2
NaN	IDO1
NaN	CDA
NaN	GRIN3A,CAPN3
NaN	GZMB,IL1B
NaN	CD3E,CD8A
NaN	TNFAIP3,BIRC3
NaN	STAT4,TNFSF10,JAK2,STAT1
NaN	PLA2G2D,RARRES3,JAK2
NaN	PGM5
NaN	BIRC3,GRIN3A,CAPN3
NaN	IL32,IL1B
NaN	CXCL12,EPHB3,JAK2,GRIN3A
NaN	DNM1,UBD,HSPA6,DNAJB1,CAPN3
NaN	PGM5
NaN	HSPH1,HSPA6
NaN	SLC38A1,GRIN3A
NaN	PLA2G2D,IL1B,STAT1
NaN	DPEP2
NaN	PGM5
NaN	CYP27A1
NaN	BIRC3,TRAF1

Garrido-Martin EM, *et al. J Immunother Cancer* 2020; 8:e000778. doi: 10.1136/jitc-2020-000778

NaN	CAMK2D,CD3E,IL2RA
NaN	TNFSF10,IRF1
NaN	PLA2G2D,RARRES3
NaN	CD3E,HSPA6,IL1B,JAK2,CCL5,STAT1
NaN	TLN2,CAPN3
NaN	PLA2G2D,RARRES3,PDE4B
NaN	JAK2,CCL5,STAT1
NaN	IDO1
NaN	PGM5
NaN	GZMB
NaN	DNM1,MAPRE3
NaN	JAK2,STAT1
NaN	JAK2,STAT1
NaN	KPNA2
NaN	SPN,CXCL12,MMP2,MMP25
NaN	CXCL12,EPHB3
NaN	ITPKB
NaN	ITPKB
NaN	STAT4,STAT1,IRF1
NaN	GZMA
NaN	RBP7
NaN	TNFAIP3,TRAF1
NaN	ACSS1
NaN	IL15RA,JAK2
NaN	MXD1
NaN	JAK2,STAT1
NaN	AGRP,JAK2
NaN	STAT1
NaN	GZMB
NaN	ITPKB
NaN	JAK2,STAT1
NaN	IDO1
NaN	KCNJ2,KCNJ10,GRIN3A
0	RGS1,CAMK2D,RGS16,GRIN3A
NaN	CD3E
NaN	ME3
NaN	HSPH1,HSPA6,DNAJB1
NaN	STAT4,STAT1
NaN	TNFSF10,BIRC3
NaN	MMP2,MMP25
NaN	CD3E,CCL5
NaN	DNM1,UBD
1	MMP2,JAK2,STAT1,MMP25
NaN	BIRC3,CAPN3
NaN	JAK2,STAT1
NaN	JAK2

Garrido-Martin EM, *et al. J Immunother Cancer* 2020; 8:e000778. doi: 10.1136/jitc-2020-000778

NaN	TLN2,CAPN3
NaN	NTRK3,TNFAIP3,IL1B
NaN	GRIN3A
NaN	RBP7
NaN	PLA2G2D
NaN	NTRK3,CXCL12,EPHB3,MMP2,ROBO3,RTN4R
NaN	JAK2,STAT1,IRF1
NaN	SPN
NaN	MMP2,JAK2,mir-34
NaN	JAK2,IGFBP2
NaN	ACSS1
NaN	SOD2,DNAJA4,DNAJB1
NaN	CAMK2D,GRIN3A
NaN	CD3E,CD8A
NaN	TAP1
NaN	TRAF1
NaN	WARS
NaN	TLN2,PLA2G2D,STAT1
NaN	CAMK2D,TNNI2,GRIN3A
NaN	JAK2,STAT1
NaN	TRAF1
NaN	CAPN3
NaN	PDE4B
NaN	RBP7
NaN	MMP2,MMP25
NaN	PLA2G2D
NaN	GCH1
NaN	IL1B,HTRA1,SLC39A8
NaN	CAMK2D,GRIN3A
NaN	CYP27A1,IL1B
NaN	STAT1
NaN	HS3ST2
NaN	IL1B,JAK2
NaN	GDF15,JAK2
NaN	GZMB
NaN	IL1B,JAK2
NaN	HS3ST2
NaN	IL1B,CCL5
NaN	CAPN3
NaN	IL1B
NaN	CD3E,CD72,IL1B
NaN	ATP6V0D2,TAP1
NaN	HS3ST2
NaN	CD3E
NaN	HS3ST2
NaN	STAT1

Garrido-Martin EM, *et al. J Immunother Cancer* 2020; 8:e000778. doi: 10.1136/jitc-2020-000778

NaN	CYP27A1,IL1B
NaN	CD3E
NaN	CAMK2D,CD3E
NaN	TRAF1
NaN	STAT1
NaN	CDC42BPA,CD3E
NaN	IL1B
NaN	CCL5
NaN	IL2RA
NaN	SOD2,LRRK2
NaN	CAMK2D,MMP2
NaN	AQP3,HSPA6
NaN	UBD,SOX13
NaN	CAMK2D
NaN	JAK2
NaN	CAMK2D,RGS16,PDE4B
NaN	MMP2
NaN	HS3ST2
NaN	PLA2G2D,RARRES3
NaN	GCH1
NaN	JAK2
NaN	CCL5
NaN	STAT1
NaN	IL1B,JAK2
NaN	DUSP5,CAMK2D,TNNI2,PDE4B
NaN	CAMK2D,HS3ST2,IL1B
NaN	RBP7,JAK2
NaN	HS3ST2
NaN	NTRK3
NaN	TRAF1
NaN	GZMB
NaN	CD3E
NaN	PLA2G2D,RARRES3
NaN	JAK2
NaN	UBD
NaN	IL1B,MMP2
NaN	IL1B
NaN	CD3E
NaN	STAT1
NaN	MMP2,CSTB
NaN	TLN2
NaN	SOD2
NaN	MMP2
NaN	PLA2G2D
NaN	JAK2
NaN	CDA

Garrido-Martin EM, *et al. J Immunother Cancer* 2020; 8:e000778. doi: 10.1136/jitc-2020-000778

NaN	IL1B
NaN	PLA2G2D
NaN	TLN2
NaN	IL1B
NaN	IL1B
NaN	DNM1,UBD
NaN	CD3E
NaN	CXCL12
NaN	PLA2G2D
NaN	CD3E
NaN	DNM1
NaN	PDE4B
NaN	PDE4B
NaN	CAMK2D
NaN	CAMK2D,JAK2,BIRC3
NaN	IL1B
NaN	IL1B
NaN	NTRK3
NaN	DNAJB1
NaN	JAK2
NaN	CAMK2D
NaN	CAMK2D
NaN	WARS
NaN	EPHB3
NaN	UBD
NaN	IL1B,BIRC3
NaN	CD3E,PLA2G2D
NaN	MMP2
NaN	CAMK2D
NaN	CAMK2D
NaN	BIRC3
NaN	CAMK2D
NaN	TLN2
NaN	IL2RA
NaN	JAK2
NaN	DUSP5
NaN	DUSP5
NaN	DUSP5,ITPKB
NaN	DUSP5
NaN	DUSP5
NaN	DUSP5
NaN	RGS16
NaN	STAT1
NaN	FABP4
NaN	NTRK3
NaN	PDE4B

Garrido-Martin EM, *et al. J Immunother Cancer* 2020; 8:e000778. doi: 10.1136/jitc-2020-000778

NaN	CAMK2D
NaN	JAK2
NaN	TLN2,CAPN3
NaN	CAMK2D,PDE4B
NaN	PDE4B
NaN	SOD2

Confidential: For Review Only

M1 geneset	M2 geneset
AATF	ADAM15
ADAR	ADAM28
AIM2	ADORA3
ALPK1	ALDH5A1
ANKRD22	ALOX15
APOBEC3A	ALOX5AP
APOL2	ARG1
APOL3	ARG2
APOL6	ARHGAP23
ARS2	ATG7
BATF2	AUH
BTN3A1	BAP1
BTN3A2	BATF3
BTN3A3	BCL3
C6ORF106	C3AR1
C9ORF109	C5AR1
CCL15	CABIN1
CCL20	CARD9
CCL5	CBR3
CD40	CCDC6
CD69	CCL17
CFB	CCL18
CLDN7	CCL22
CXCL10	CCL26
CXCL11	CCRN4L
CXCL12	CD14
CXCL9	CD163
DCAF6	CD206
DYNLT1	CD209
ELF4	CLIC2
ETV7	CRH
FAM26F	CTNNAL1
GBP1	CXORF40A
GBP2	CYBB
GBP4	DAAM1
GBP5	DAP
HAPLN3	DHRS11
HCG4	DLST
HCP5	DUSP6
HLA-A	EGLN3
HLA-B	ESPNL
HLA-C	EXOC2
HLA-E	FOS
HLA-F	FOXD2
HLA-G	FOXQ1

Confidential: For Review Only

HLA-H	GALNT12
IL1F7	GALNTL4
IL11	GLS
IL12A	GOLGA8B
IL12B	GPD1L
IL23A	GPR65
IL27	HARS2
IL6	HEMK1
IRF1	HOMER2
IRF5	HPS1
IRF7	IL10
IRF9	IL10RA
ITGAL	IL10RB
JAK2	IL13RA1
KCNJ2	IL17RB
LBA1	IL1R1
LOC400759	IL1R2
LRRCC1	IL4I1
MDK	IL4R
MFAP1	INTS3
MIA3	IRF4
MOV10	ISYNA1
MR1	ITPRIPL1
NFIX	KIAA0182
NLRC5	KIAA1671
NMI	KTN1
NOS2	LOC284998
NUP50	LOC152195
OAT	LYZ
ORC3L	MACF1
OSCAR	MAOA
PARP14	MAP1A
PAXIP1	MLKL
PLEKHA7	MMP1
PML	MMP12
PRPF3	MMP7
PSMB10	MMP9
PSMB8	MUTED
PSMB9	NAGPA
RARRES3	NLRP12
RBCK1	NMD3
SELT	PALLD
SERPING1	PELP1
SERPINI1	PITPNA
SLAMF7	PITRM1
SLC15A4	PPP1R14A

Confidential: For Review Only

SLC6A12	PPP1R3B
SP100	RAB33A
SP140	RAB40B
STAT1	RAMP1
STAT2	RAP1GAP
TAP1	RASL10A
TAP2	RASSF7
TIFA	RPL28
TLR2	RRP1B
TLR4	RTKN
TM9SF2	S100A8
TM9SF3	S100A9
TMEM140	SNX8
TNF	SOCS1
TNFSF10	SOCS3
TRAFD1	SOX8
TUT1	STAB1
UBD	STAT6
UBE2L6	SUCNR1
USP9X	SULF2
VAMP5	SYT17
VPS36	TGFBI
WARS	TIGD5
ZDHHC8	TOR3A
ZNF668	TRIB2
	TTC9C
	UST
	WDR33
	WNT5A
	WNT5B
	ZNF317

Confidential: For Review Only

Supplementary Table 6: Data of overall survival in a NSCLC adenocar

Anonimized ID	Gender	Age	Dead/Alive
LUAD-2007-001	M	51	1
LUAD-2007-002	F	69	1
LUAD-2007-003	M	42	1
LUAD-2007-004	M	89	1
LUAD-2007-005	F	56	1
LUAD-2007-006	F	73	1
LUAD-2007-007	M	74	0
LUAD-2007-008	M	73	1
LUAD-2007-009	M	66	0
LUAD-2007-010	F	71	1
LUAD-2007-011	M	59	NA
LUAD-2007-012	M	53	1
LUAD-2007-013	M	77	1
LUAD-2007-014	F	60	1
LUAD-2007-015	F	76	0
LUAD-2007-016	M	61	1
LUAD-2007-017	M	71	1
LUAD-2007-018	M	52	0
LUAD-2007-019	F	74	1
LUAD-2007-020	M	65	1
LUAD-2007-021	M	50	1
LUAD-2007-022	F	67	1
LUAD-2007-023	F	74	1
LUAD-2007-024	M	72	1
LUAD-2007-025	M	74	1
LUAD-2007-026	M	63	1
LUAD-2007-027	F	79	0
LUAD-2007-028	F	66	0
LUAD-2007-029	M	61	0
LUAD-2007-030	M	65	0
LUAD-2007-031	M	38	0
LUAD-2007-033	M	83	1
LUAD-2007-034	F	67	1
LUAD-2007-035	F	53	1
LUAD-2007-036	F	63	0
LUAD-2007-037	M	71	1
LUAD-2007-038	M	76	1
LUAD-2007-039	M	79	1
LUAD-2007-040	M	80	1
LUAD-2007-041	F	67	0
LUAD-2007-042	M	71	0
LUAD-2007-044	M	74	1
LUAD-2007-045	M	63	1
LUAD-2007-046	F	47	1
LUAD-2007-047	F	64	1
LUAD-2007-048	M	64	1

Confidential: For Review Only

LUAD-2007-049	F	66	1
LUAD-2007-050	F	62	0
LUAD-2007-051	F	76	1
LUAD-2007-052	M	70	1
LUAD-2007-053	M	69	0
LUAD-2007-054	M	68	1
LUAD-2007-055	M	61	1
LUAD-2007-056	M	43	1
LUAD-2007-057	M	71	NA
LUAD-2007-058	F	51	1
LUAD-2007-059	F	60	0
LUAD-2007-060	F	42	1
LUAD-2008-061	M	63	1
LUAD-2008-062	F	60	1
LUAD-2008-063	F	79	1
LUAD-2008-064	M	73	1
LUAD-2008-065	F	49	NA
LUAD-2008-066	F	63	1
LUAD-2008-067	F	54	1
LUAD-2008-068	F	84	0
LUAD-2008-069	F	71	0
LUAD-2008-070	F	71	0
LUAD-2008-071	M	67	1
LUAD-2008-072	M	68	1
LUAD-2008-073	F	65	0
LUAD-2008-074	F	65	1
LUAD-2008-075	F	63	0
LUAD-2008-077	F	70	0
LUAD-2008-078	M	53	0
LUAD-2008-079	F	75	0
LUAD-2008-080	F	58	NA
LUAD-2008-081	M	47	1
LUAD-2008-082	F	81	1
LUAD-2008-083	F	71	1
LUAD-2008-084	M	55	1
LUAD-2008-085	F	82	1
LUAD-2008-086	F	70	1
LUAD-2008-087	M	67	1
LUAD-2008-088	M	58	1
LUAD-2008-089	M	74	1
LUAD-2008-090	F	62	0
LUAD-2008-091	F	69	0
LUAD-2008-092	M	72	1
LUAD-2008-093	F	63	1
LUAD-2008-094	M	76	1
LUAD-2008-095	F	79	1
LUAD-2008-096	F	69	0
LUAD-2008-097	F	71	0
LUAD-2008-098	M	51	1

Confidential: For Review Only

LUAD-2008-099	F	54	0
LUAD-2008-101	M	73	1
LUAD-2008-102	M	75	0
LUAD-2008-103	M	58	1
LUAD-2008-104	M	71	1
LUAD-2008-105	F	76	1
LUAD-2008-106	M	81	1
LUAD-2008-107	F	64	0
LUAD-2008-108	F	77	1
LUAD-2008-109	M	59	0
LUAD-2008-110	F	55	1
LUAD-2008-111	F	75	0
LUAD-2008-112	F	69	0
LUAD-2008-113	M	73	1
LUAD-2008-114	F	67	1
LUAD-2008-116	M	63	1
LUAD-2008-117	F	49	0
LUAD-2008-118	F	67	0
LUAD-2008-119	F	73	0
LUAD-2008-120	M	62	1
LUAD-2008-121	F	79	1
LUAD-2008-122	F	67	0
LUAD-2008-123	F	70	0
LUAD-2008-124	M	72	0
LUAD-2008-125	M	61	1
LUAD-2008-126	M	72	1
LUAD-2008-127	F	64	1
LUAD-2008-128	M	83	1
LUAD-2008-129	F	63	1
LUAD-2008-130	F	58	0
LUAD-2008-131	M	73	1
LUAD-2008-132	M	78	1
LUAD-2008-133	F	58	0
LUAD-2008-134	M	44	1
LUAD-2008-135	M	70	0
LUAD-2008-136	M	78	1
LUAD-2008-137	F	76	1
LUAD-2008-138	F	59	1
LUAD-2008-139	F	75	0
LUAD-2008-140	M	69	1
LUAD-2008-141	M	61	1
LUAD-2008-143	F	76	1
LUAD-2009-144	M	70	1
LUAD-2009-145	M	63	0
LUAD-2009-146	F	51	0
LUAD-2009-147	M	59	1
LUAD-2009-148	M	56	1
LUAD-2009-149	F	58	0
LUAD-2009-150	F	61	1

Confidential: For Review Only

LUAD-2009-151	M	72	1
LUAD-2009-152	F	72	1
LUAD-2009-153	F	60	0
LUAD-2009-154	F	54	1
LUAD-2009-155	M	88	1
LUAD-2009-156	M	54	1
LUAD-2009-157	F	46	0
LUAD-2009-159	M	57	0
LUAD-2009-160	F	59	1
LUAD-2009-161	M	51	0
LUAD-2009-162	F	60	0
LUAD-2009-163	F	63	0
LUAD-2009-164	M	84	1
LUAD-2009-165	F	60	0
LUAD-2009-166	M	62	0
LUAD-2009-167	M	72	0
LUAD-2009-168	F	81	1
LUAD-2009-169	M	81	1
LUAD-2009-171	M	76	1
LUAD-2009-172	M	73	0
LUAD-2009-173	F	68	0
LUAD-2009-174	M	66	0
LUAD-2009-175	M	56	0
LUAD-2009-176	M	64	1
LUAD-2009-177	F	62	0
LUAD-2009-178	M	69	1
LUAD-2009-179	M	77	0
LUAD-2009-180	M	61	0
LUAD-2009-182	F	70	1
LUAD-2009-183	M	67	1
LUAD-2009-184	M	81	1
LUAD-2009-185	M	71	1
LUAD-2009-186	M	68	0
LUAD-2009-187	F	67	1
LUAD-2009-188	M	76	0
LUAD-2009-189	F	83	0
LUAD-2009-190	M	82	1
LUAD-2009-191	F	62	0
LUAD-2009-192	M	80	1
LUAD-2009-193	M	65	1
LUAD-2009-194	F	69	1
LUAD-2009-195	F	55	0
LUAD-2009-196	F	55	0
LUAD-2009-197	F	65	1
LUAD-2009-198	M	61	0
LUAD-2009-200	F	84	0
LUAD-2009-201	F	88	1
LUAD-2009-202	F	60	0
LUAD-2009-203	M	71	0

Confidential: For Review Only

Garrido-Martin EM, *et al. J Immunother Cancer* 2020; 8:e000778. doi: 10.1136/jitc-2020-000778

LUAD-2009-204	M	71	0
LUAD-2009-205	F	70	0
LUAD-2009-206	F	58	0
LUAD-2009-207	F	83	0
LUAD-2009-208	M	70	1
LUAD-2009-209	M	61	1
LUAD-2009-210	F	74	1
LUAD-2009-211	M	72	1
LUAD-2009-212	M	77	1
LUAD-2009-213	M	66	1
LUAD-2009-214	F	66	1
LUAD-2009-215	M	71	1
LUAD-2009-217	F	78	1
LUAD-2009-218	M	77	1
LUAD-2009-219	F	78	0
LUAD-2009-220	F	61	1
LUAD-2009-221	F	66	1
LUAD-2009-222	M	67	1
LUAD-2009-223	F	70	1
LUAD-2009-224	M	63	1
LUAD-2009-225	M	57	1
LUAD-2009-226	M	56	1
LUAD-2009-227	M	75	0
LUAD-2009-229	F	63	1
LUAD-2009-230	F	61	NA
LUAD-2009-231	F	59	0
LUAD-2009-232	F	59	1
LUAD-2009-233	M	73	NA
LUAD-2009-234	M	66	1
LUAD-2009-235	NA	NA	NA
LUAD-2010-236	F	74	1
LUAD-2010-237	M	55	0
LUAD-2010-238	M	65	0
LUAD-2010-239	M	76	0
LUAD-2010-240	F	78	0
LUAD-2010-241	M	79	0
LUAD-2010-242	M	72	0
LUAD-2010-243	F	45	1
LUAD-2010-245	F	69	1
LUAD-2010-246	M	67	0
LUAD-2010-247	M	68	0
LUAD-2010-248	M	60	1
LUAD-2010-249	M	75	0
LUAD-2010-250	F	71	0
LUAD-2010-251	F	69	1
LUAD-2010-252	M	72	1
LUAD-2010-253	F	85	1
LUAD-2010-254	F	75	0
LUAD-2010-255	F	61	1

Confidential: For Review Only

LUAD-2010-256	M	60	1
LUAD-2010-257	M	65	1
LUAD-2010-258	F	78	0
LUAD-2010-259	F	55	1
LUAD-2010-260	F	77	0
LUAD-2010-261	F	52	0
LUAD-2010-262	F	67	0
LUAD-2010-263	F	66	0
LUAD-2010-264	M	79	0
LUAD-2010-265	F	62	0
LUAD-2010-266	F	67	1
LUAD-2010-267	F	56	1
LUAD-2010-268	M	75	1
LUAD-2010-269	M	67	1
LUAD-2010-270	M	65	1
LUAD-2010-271	M	79	0
LUAD-2010-272	M	75	0
LUAD-2010-273	M	68	0
LUAD-2010-274	F	81	1
LUAD-2010-275	F	77	0
LUAD-2010-276	M	60	1
LUAD-2010-277	F	70	0
LUAD-2010-278	F	78	1
LUAD-2010-279	F	78	0
LUAD-2010-280	F	80	0
LUAD-2010-282	F	73	0
LUAD-2010-283	F	64	1
LUAD-2010-284	M	67	1
LUAD-2010-285	M	65	1
LUAD-2010-286	F	71	0
LUAD-2010-287	M	57	1
LUAD-2010-288	M	62	0
LUAD-2010-289	F	83	0
LUAD-2010-290	F	70	0
LUAD-2010-291	F	63	1
LUAD-2010-292	F	64	1
LUAD-2010-293	F	69	0
LUAD-2010-294	M	73	1
LUAD-2010-295	F	56	0
LUAD-2010-296	F	81	0
LUAD-2010-297	M	59	0
LUAD-2010-299	M	75	NA
LUAD-2010-300	F	70	1
LUAD-2010-301	M	59	0
LUAD-2010-302	F	70	0
LUAD-2010-303	M	51	1
LUAD-2010-304	M	69	1
LUAD-2010-305	M	74	0
LUAD-2010-306	M	61	0

Confidential: For Review Only

LUAD-2010-307	F	70	0
LUAD-2010-308	F	78	1
LUAD-2010-310	F	67	0
LUAD-2010-312	M	73	1
LUAD-2010-313	M	54	0
LUAD-2010-314	M	76	1
LUAD-2010-315	F	64	0
LUAD-2010-316	F	64	0
LUAD-2010-317	M	63	0
LUAD-2010-318	M	60	0
LUAD-2010-319	M	72	0
LUAD-2010-320	M	61	1
LUAD-2010-321	F	58	0
LUAD-2010-322	F	71	0
LUAD-2010-323	M	54	1
LUAD-2010-325	M	51	1
LUAD-2010-326	M	79	1
LUAD-2010-327	M	76	1
LUAD-2010-328	M	64	1
LUAD-2010-329	M	60	0
LUAD-2010-330	M	59	1
LUAD-2010-331	M	56	0
LUAD-2010-332	M	67	0
LUAD-2010-333	F	66	0
LUAD-2010-334	F	72	1
LUAD-2010-335	F	63	1
LUAD-2010-336	F	56	1
LUAD-2010-337	F	66	1
LUAD-2010-338	M	49	0
LUAD-2010-339	F	79	1
LUAD-2010-340	F	66	0
LUAD-2010-341	M	63	0
LUAD-2010-342	M	65	1
LUAD-2010-343	M	71	0
LUAD-2011-344	M	76	0
LUAD-2011-345	F	63	NA
LUAD-2011-347	M	63	1
LUAD-2011-348	F	51	1
LUAD-2011-349	M	72	1
LUAD-2011-350	F	72	0
LUAD-2011-351	F	56	1
LUAD-2011-352	F	45	1
LUAD-2011-353	M	63	1
LUAD-2011-354	F	50	0
LUAD-2011-355	F	79	1
LUAD-2011-356	F	73	1
LUAD-2011-357	M	72	1
LUAD-2011-360	F	63	1
LUAD-2011-361	M	68	0

Confidential: For Review Only

LUAD-2011-362	M	68	0
LUAD-2011-363	F	64	0
LUAD-2011-364	M	68	0
LUAD-2011-365	F	69	1
LUAD-2011-366	F	61	1
LUAD-2011-367	M	66	1
LUAD-2011-368	M	50	0
LUAD-2011-369	M	60	0
LUAD-2011-370	M	68	NA
LUAD-2011-371	M	62	0
LUAD-2011-372	M	76	0
LUAD-2011-373	F	54	0
LUAD-2011-374	F	68	1
LUAD-2011-375	F	74	0
LUAD-2011-376	M	69	0
LUAD-2011-377	F	71	0
LUAD-2011-378	F	70	0
LUAD-2011-379	F	49	1
LUAD-2011-380	M	80	0
LUAD-2011-381	F	68	0
LUAD-2011-382	F	71	1
LUAD-2011-383	M	50	0
LUAD-2011-384	F	57	1
LUAD-2011-385	M	57	0
LUAD-2011-386	M	54	1
LUAD-2011-387	M	73	NA
LUAD-2011-388	M	92	1
LUAD-2011-389	M	52	0
LUAD-2011-390	F	63	1
LUAD-2011-391	F	65	0
LUAD-2011-392	F	74	NA
LUAD-2011-393	M	54	1
LUAD-2011-394	M	75	0
LUAD-2011-396	F	77	1
LUAD-2011-397	M	69	1
LUAD-2011-398	M	65	1
LUAD-2011-399	F	66	0
LUAD-2011-400	F	73	0
LUAD-2011-401	F	72	0
LUAD-2011-402	M	81	0
LUAD-2011-403	M	56	0
LUAD-2011-404	M	72	0
LUAD-2011-405	F	68	0
LUAD-2011-406	F	59	0
LUAD-2011-407	M	62	0
LUAD-2011-408	F	79	NA
LUAD-2011-409	M	78	1
LUAD-2011-410	F	65	1
LUAD-2011-411	M	57	0

Confidential: For Review Only

Supplemental material BMJ Publishing Group Limited (BMJ) disclaims all liability and responsibility arising from any reliance placed on this supplemental material which has been supplied by the author(s) J Immunother Cancer

LUAD-2011-412	F	69	1
LUAD-2011-413	F	64	1
LUAD-2011-414	M	40	1
LUAD-2011-415	F	62	0
LUAD-2011-416	M	54	1
LUAD-2011-417	M	75	0
LUAD-2011-418	M	78	1
LUAD-2011-419	F	61	0
LUAD-2011-420	F	68	1
LUAD-2011-421	M	70	0
LUAD-2011-422	F	76	0
LUAD-2011-423	F	74	0
LUAD-2011-425	M	76	1
LUAD-2011-426	M	62	0
LUAD-2011-427	F	76	0
LUAD-2011-428	F	70	1
LUAD-2011-429	M	69	0
LUAD-2011-430	M	63	0
LUAD-2011-432	F	51	0
LUAD-2011-433	M	69	0
LUAD-2011-434	F	62	1
LUAD-2011-435	F	64	1
LUAD-2011-438	M	63	1
LUAD-2011-439	F	65	1
LUAD-2011-440	M	62	0
LUAD-2011-441	F	60	0
LUAD-2011-442	M	64	0
LUAD-2011-444	M	64	0
LUAD-2011-445	F	50	0
LUAD-2011-446	M	70	0
LUAD-2011-447	F	62	0
LUAD-2011-448	M	73	0
LUAD-2011-450	M	67	0
LUAD-2011-451	M	74	1
LUAD-2011-452	M	72	0
LUAD-2011-453	M	72	0
LUAD-2011-454	F	56	1
LUAD-2011-455	F	56	0
LUAD-2011-456	M	61	0
LUAD-2011-457	F	64	0
LUAD-2011-458	M	57	1
LUAD-2011-459	F	59	0

Confidential: For Review Only

Garrido-Martin EM, et al. J Immunother Cancer 2020; 8:e000778. doi: 10.1136/jitc-2020-000778

rcinoma (LUAD) retrospective cohort of University of Southampton UoS/Southl

Combined survival	CXCL9 score
367	1
417	2
342	2
614	2
161	2
996	2
3065	2
511	2
3060	2
263	2
617	2
253	2
1108	2
1153	2
3032	2
710	2
544	1
3018	2
1611	2
NA	2
1172	1
1390	2
386	3
36	2
10	2
2111	2
2975	2
2963	2
2960	2
2955	1
NA	1
544	3
908	2
1482	2
2934	2
81	2
9	2
136	2
371	2
2900	1
2897	2
496	2
151	2
390	2
171	2
493	2

Confidential: For Review Only

Garrido-Martin EM, *et al. J Immunother Cancer* 2020; 8:e000778. doi: 10.1136/jitc-2020-000778

327	2
2857	3
1565	1
1050	2
2829	2
359	2
1587	2
2047	2
750	2
2164	2
2764	2
1441	1
1	2
299	1
473	2
34	2
2067	2
289	2
355	1
2715	3
2711	2
2703	2
684	1
709	2
2681	2
1713	2
2676	2
2669	2
NA	1
2667	2
1589	1
472	2
1326	2
215	2
522	2
664	2
2393	3
688	2
394	2
1818	1
2571	2
NA	2
337	2
1183	2
1485	2
206	2
2536	2
2535	2
2090	2

Confidential: For Review Only

2533	2
2132	2
2522	1
1161	2
1261	2
1970	2
895	2
2500	2
605	3
2492	2
62	3
2470	2
2466	2
730	2
385	2
31	1
2469	2
2457	3
2450	2
569	2
494	2
2449	3
2445	2
NA	2
2248	1
209	1
15	1
1209	2
979	3
2420	2
144	1
779	2
2408	2
1662	3
2401	2
279	2
288	3
61	1
2389	2
6	2
35	2
2139	2
2026	2
2364	2
2360	3
89	2
467	1
2346	2
411	3

Confidential: For Review Only

BMJ Publishing Group Limited (BMJ) disclaims all liability and responsibility arising from any reliance placed on this supplemental material which has been supplied by the author(s)

51	1
1386	1
2322	2
1187	2
7	2
1073	2
2304	2
2302	3
863	1
2296	1
2295	2
2294	2
1184	2
2288	1
2284	2
2284	2
814	1
1441	2
44	2
2260	2
2253	2
NA	2
2247	2
398	2
2234	1
345	2
2226	2
2225	2
368	1
1187	2
1265	2
96	2
2210	3
1409	2
2185	3
2184	2
1656	3
2170	2
48	1
1328	1
1110	2
2154	2
2149	2
115	1
2143	2
2137	2
1784	1
2128	2
2121	2

Confidential: For Review Only

Garrido-Martin EM, *et al. J Immunother Cancer* 2020; 8:e000778. doi: 10.1136/jitc-2020-000778

2121	2
NA	1
2119	3
NA	1
540	2
639	3
287	1
324	3
22	1
1514	2
50	1
480	1
862	2
146	2
2074	2
593	3
1030	3
908	1
1360	1
547	1
1598	2
292	2
1468	2
834	2
35	2
2035	2
178	1
652	2
1019	2
0	2
1763	1
1990	1
1990	2
1987	2
1986	3
1982	2
NA	1
237	2
402	2
1946	2
NA	2
1080	1
NA	1
1926	3
911	2
589	2
299	1
1919	2
285	2

Confidential: For Review Only

814	2
646	1
NA	1
494	2
1909	1
1905	2
NA	2
1898	3
1897	3
1896	1
175	2
290	1
154	2
836	2
752	2
1874	2
1871	3
NA	1
483	1
1850	2
1282	2
1848	2
1592	3
NA	2
1836	2
1833	2
969	2
696	2
788	2
1819	3
620	2
1815	3
1814	2
NA	2
691	2
663	2
1807	2
140	1
1804	3
1801	1
1800	2
38	2
393	1
NA	2
1780	3
785	2
1650	2
1777	2
1771	2

Confidential: For Review Only

1764	2
1325	1
1737	2
149	3
NA	3
841	3
1723	2
NA	2
1720	2
NA	1
1715	3
473	2
1713	3
NA	3
996	2
630	1
489	3
750	1
460	1
NA	2
598	2
NA	2
NA	2
1678	3
553	2
1134	2
715	1
774	2
1661	1
423	1
1660	2
1660	1
866	1
1657	2
NA	2
71	3
1020	2
1159	2
618	2
1619	2
808	2
538	2
431	2
1612	2
35	2
847	2
470	2
222	2
1580	2

Confidential: For Review Only

1570	2
1568	1
1567	1
1320	1
402	3
464	1
1555	1
1555	2
19	2
1546	1
1540	2
1539	2
1304	2
1534	2
1533	2
1533	2
1533	1
30	1
1527	1
1526	1
577	2
1520	1
565	2
1518	3
357	2
27	2
1514	2
1511	3
226	3
1506	2
437	1
615	1
1496	3
975	1
20	2
89	2
1478	3
1477	2
1476	2
1476	1
1475	2
1475	1
1470	3
1462	3
1455	2
42	2
315	2
721	1
1443	1

Confidential: For Review Only

948	2
1016	2
490	2
1436	1
663	2
1429	1
8	2
1423	2
1028	2
1414	1
1409	2
1409	3
275	2
1401	2
1399	2
1068	2
1394	2
1393	3
1387	2
1387	2
139	2
482	2
237	2
114	2
1353	2
1350	3
1349	2
1345	1
1342	2
1332	2
1331	3
1325	1
1322	2
52	1
1317	2
1310	3
66	1
1300	1
1297	2
1293	2
48	1
1280	2

Confidential: For Review Only

hampton Univ. Hospital

Confidential: For Review Only

Supplementary Table 6: Data of overall survival in the NSCLC adenocarcinoma (LU

Patient ID	T/NL	Tumour.type
TCGA-50-6591	T	LUAD
TCGA-55-7726	T	LUAD
TCGA-62-A46O	T	LUAD
TCGA-71-6725	T	LUAD
TCGA-97-8171	T	LUAD
TCGA-91-6847	T	LUAD
TCGA-64-1678	T	LUAD
TCGA-50-5931	T	LUAD
TCGA-NJ-A7XG	T	LUAD
TCGA-50-5946	T	LUAD
TCGA-64-5775	T	LUAD
TCGA-55-7815	T	LUAD
TCGA-86-8054	T	LUAD
TCGA-86-7955	T	LUAD
TCGA-78-7633	T	LUAD
TCGA-86-8279	T	LUAD
TCGA-73-4670	T	LUAD
TCGA-49-AAR9	T	LUAD
TCGA-55-7724	T	LUAD
TCGA-MP-A4SY	T	LUAD
TCGA-69-7979	T	LUAD
TCGA-73-4659	T	LUAD
TCGA-44-7671	T	LUAD
TCGA-93-8067	T	LUAD
TCGA-05-4420	T	LUAD
TCGA-55-6972	T	LUAD
TCGA-55-8512	T	LUAD
TCGA-86-7714	T	LUAD
TCGA-05-5428	T	LUAD
TCGA-55-7727	T	LUAD
TCGA-J2-8194	T	LUAD
TCGA-55-8087	T	LUAD
TCGA-97-8179	T	LUAD
TCGA-78-7155	T	LUAD
TCGA-55-6981	T	LUAD
TCGA-64-1680	T	LUAD
TCGA-97-7937	T	LUAD
TCGA-86-8674	T	LUAD
TCGA-55-7725	T	LUAD
TCGA-55-8505	T	LUAD
TCGA-55-7913	T	LUAD
TCGA-62-A46P	T	LUAD
TCGA-50-5932	T	LUAD
TCGA-50-5072	T	LUAD
TCGA-95-7043	T	LUAD
TCGA-78-7150	T	LUAD

Confidential: For Review Only

TCGA-44-2665	T	LUAD
TCGA-05-4397	T	LUAD
TCGA-05-5429	T	LUAD
TCGA-64-5774	T	LUAD
TCGA-55-8094	T	LUAD
TCGA-NJ-A4YF	T	LUAD
TCGA-67-6215	T	LUAD
TCGA-05-4390	T	LUAD
TCGA-97-7938	T	LUAD
TCGA-78-7158	T	LUAD
TCGA-MP-A5C7	T	LUAD
TCGA-05-5715	T	LUAD
TCGA-44-6776	T	LUAD
TCGA-50-5944	T	LUAD
TCGA-55-7910	T	LUAD
TCGA-NJ-A4YI	T	LUAD
TCGA-J2-A4AD	T	LUAD
TCGA-95-8494	T	LUAD
TCGA-44-7670	T	LUAD
TCGA-91-6836	T	LUAD
TCGA-62-A46V	T	LUAD
TCGA-38-4631	T	LUAD
TCGA-62-A470	T	LUAD
TCGA-38-4628	T	LUAD
TCGA-78-7156	T	LUAD
TCGA-55-7570	T	LUAD
TCGA-78-7154	T	LUAD
TCGA-35-3615	T	LUAD
TCGA-55-8507	T	LUAD
TCGA-49-4486	T	LUAD
TCGA-44-7667	T	LUAD
TCGA-86-7711	T	LUAD
TCGA-62-A471	T	LUAD
TCGA-78-7167	T	LUAD
TCGA-69-7764	T	LUAD
TCGA-44-2666	T	LUAD
TCGA-73-4677	T	LUAD
TCGA-97-A4M3	T	LUAD
TCGA-J2-8192	T	LUAD
TCGA-62-8397	T	LUAD
TCGA-55-A57B	T	LUAD
TCGA-73-4676	T	LUAD
TCGA-62-8399	T	LUAD
TCGA-78-7220	T	LUAD
TCGA-97-8175	T	LUAD
TCGA-55-8090	T	LUAD
TCGA-91-6829	T	LUAD
TCGA-44-A47B	T	LUAD
TCGA-73-A9RS	T	LUAD

Confidential: For Review Only

TCGA-73-4668	T	LUAD
TCGA-78-8662	T	LUAD
TCGA-91-A4BD	T	LUAD
TCGA-05-4396	T	LUAD
TCGA-38-4630	T	LUAD
TCGA-69-7973	T	LUAD
TCGA-99-8025	T	LUAD
TCGA-44-5644	T	LUAD
TCGA-73-4675	T	LUAD
TCGA-44-2662	T	LUAD
TCGA-05-4395	T	LUAD
TCGA-67-4679	T	LUAD
TCGA-55-8614	T	LUAD
TCGA-97-8547	T	LUAD
TCGA-69-8253	T	LUAD
TCGA-78-7149	T	LUAD
TCGA-86-7713	T	LUAD
TCGA-67-3770	T	LUAD
TCGA-55-1592	T	LUAD
TCGA-55-6984	T	LUAD
TCGA-86-8055	T	LUAD
TCGA-L9-A5IP	T	LUAD
TCGA-91-8499	T	LUAD
TCGA-55-8207	T	LUAD
TCGA-55-6975	T	LUAD
TCGA-44-7660	T	LUAD
TCGA-78-7542	T	LUAD
TCGA-99-8032	T	LUAD
TCGA-49-4510	T	LUAD
TCGA-38-4626	T	LUAD
TCGA-86-8358	T	LUAD
TCGA-55-A48Y	T	LUAD
TCGA-62-8398	T	LUAD
TCGA-91-8496	T	LUAD
TCGA-05-4427	T	LUAD
TCGA-86-A4D0	T	LUAD
TCGA-93-A4JP	T	LUAD
TCGA-38-6178	T	LUAD
TCGA-78-7537	T	LUAD
TCGA-05-4426	T	LUAD
TCGA-50-6673	T	LUAD
TCGA-50-5936	T	LUAD
TCGA-05-4384	T	LUAD
TCGA-44-8119	T	LUAD
TCGA-53-7624	T	LUAD
TCGA-49-6742	T	LUAD
TCGA-44-4112	T	LUAD
TCGA-71-8520	T	LUAD
TCGA-97-8176	T	LUAD

Confidential: For Review Only

TCGA-86-8056	T	LUAD
TCGA-55-6543	T	LUAD
TCGA-53-7813	T	LUAD
TCGA-95-7562	T	LUAD
TCGA-78-7161	T	LUAD
TCGA-44-3398	T	LUAD
TCGA-35-5375	T	LUAD
TCGA-55-1596	T	LUAD
TCGA-86-A456	T	LUAD
TCGA-86-8075	T	LUAD
TCGA-64-1676	T	LUAD
TCGA-78-7163	T	LUAD
TCGA-55-8615	T	LUAD
TCGA-50-5939	T	LUAD
TCGA-44-6148	T	LUAD
TCGA-49-4494	T	LUAD
TCGA-05-4405	T	LUAD
TCGA-05-4415	T	LUAD
TCGA-4B-A93V	T	LUAD
TCGA-78-7535	T	LUAD
TCGA-95-7948	T	LUAD
TCGA-78-7536	T	LUAD
TCGA-62-8395	T	LUAD
TCGA-69-7763	T	LUAD
TCGA-44-7669	T	LUAD
TCGA-64-1679	T	LUAD
TCGA-L9-A443	T	LUAD
TCGA-55-8513	T	LUAD
TCGA-64-5815	T	LUAD
TCGA-44-7659	T	LUAD
TCGA-91-6828	T	LUAD
TCGA-55-8206	T	LUAD
TCGA-38-4627	T	LUAD
TCGA-55-A492	T	LUAD
TCGA-44-8120	T	LUAD
TCGA-05-4424	T	LUAD
TCGA-62-A46S	T	LUAD
TCGA-50-5051	T	LUAD
TCGA-44-2655	T	LUAD
TCGA-78-7166	T	LUAD
TCGA-86-6562	T	LUAD
TCGA-NJ-A55R	T	LUAD
TCGA-55-6986	T	LUAD
TCGA-44-A47A	T	LUAD
TCGA-50-5930	T	LUAD
TCGA-75-5146	T	LUAD
TCGA-75-6212	T	LUAD
TCGA-86-A4JF	T	LUAD
TCGA-78-7159	T	LUAD

Confidential: For Review Only

TCGA-J2-A4AE	T	LUAD
TCGA-86-8074	T	LUAD
TCGA-50-5942	T	LUAD
TCGA-55-8091	T	LUAD
TCGA-86-8073	T	LUAD
TCGA-64-1681	T	LUAD
TCGA-55-8096	T	LUAD
TCGA-97-7547	T	LUAD
TCGA-O1-A52J	T	LUAD
TCGA-50-6593	T	LUAD
TCGA-55-8616	T	LUAD
TCGA-MP-A4TF	T	LUAD
TCGA-55-8204	T	LUAD
TCGA-62-8394	T	LUAD
TCGA-NJ-A55O	T	LUAD
TCGA-67-6216	T	LUAD
TCGA-93-A4JN	T	LUAD
TCGA-05-4249	T	LUAD
TCGA-05-4403	T	LUAD
TCGA-49-4512	T	LUAD
TCGA-80-5611	T	LUAD
TCGA-49-6743	T	LUAD
TCGA-MP-A4TA	T	LUAD
TCGA-67-3771	T	LUAD
TCGA-MP-A4T7	T	LUAD
TCGA-55-A494	T	LUAD
TCGA-49-AARR	T	LUAD
TCGA-99-8033	T	LUAD
TCGA-44-5643	T	LUAD
TCGA-91-6831	T	LUAD
TCGA-44-6775	T	LUAD
TCGA-49-4506	T	LUAD
TCGA-55-1594	T	LUAD
TCGA-05-4402	T	LUAD
TCGA-75-7025	T	LUAD
TCGA-97-7554	T	LUAD
TCGA-50-5933	T	LUAD
TCGA-78-8640	T	LUAD
TCGA-MN-A4N4	T	LUAD
TCGA-73-4658	T	LUAD
TCGA-64-5779	T	LUAD
TCGA-95-A4VP	T	LUAD
TCGA-50-6595	T	LUAD
TCGA-44-6146	T	LUAD
TCGA-86-7953	T	LUAD
TCGA-55-5899	T	LUAD
TCGA-67-6217	T	LUAD
TCGA-44-7662	T	LUAD
TCGA-MN-A4N1	T	LUAD

Confidential: For Review Only

TCGA-55-7576	T	LUAD
TCGA-55-1595	T	LUAD
TCGA-97-8174	T	LUAD
TCGA-64-1677	T	LUAD
TCGA-97-A4M5	T	LUAD
TCGA-05-4418	T	LUAD
TCGA-97-8552	T	LUAD
TCGA-55-8097	T	LUAD
TCGA-55-8514	T	LUAD
TCGA-97-8177	T	LUAD
TCGA-44-6774	T	LUAD
TCGA-91-6849	T	LUAD
TCGA-44-6147	T	LUAD
TCGA-91-6830	T	LUAD
TCGA-67-3773	T	LUAD
TCGA-62-A472	T	LUAD
TCGA-93-7348	T	LUAD
TCGA-49-4488	T	LUAD
TCGA-05-4430	T	LUAD
TCGA-78-7148	T	LUAD
TCGA-97-7546	T	LUAD
TCGA-95-7947	T	LUAD
TCGA-44-8117	T	LUAD
TCGA-86-8278	T	LUAD
TCGA-97-A4M1	T	LUAD
TCGA-97-A4M7	T	LUAD
TCGA-49-4501	T	LUAD
TCGA-05-5423	T	LUAD
TCGA-38-4632	T	LUAD
TCGA-55-6970	T	LUAD
TCGA-62-A46Y	T	LUAD
TCGA-86-8669	T	LUAD
TCGA-62-A46R	T	LUAD
TCGA-44-7661	T	LUAD
TCGA-50-6592	T	LUAD
TCGA-86-8668	T	LUAD
TCGA-91-6840	T	LUAD
TCGA-69-8254	T	LUAD
TCGA-05-4432	T	LUAD
TCGA-MP-A4T8	T	LUAD
TCGA-J2-A4AG	T	LUAD
TCGA-78-7540	T	LUAD
TCGA-55-7283	T	LUAD
TCGA-50-5068	T	LUAD
TCGA-55-A4DF	T	LUAD
TCGA-69-8453	T	LUAD
TCGA-MP-A4TC	T	LUAD
TCGA-38-4625	T	LUAD
TCGA-78-7152	T	LUAD

Confidential: For Review Only

TCGA-50-5044	T	LUAD
TCGA-78-7143	T	LUAD
TCGA-49-AARN	T	LUAD
TCGA-78-7147	T	LUAD
TCGA-69-7760	T	LUAD
TCGA-NJ-A4YP	T	LUAD
TCGA-91-6848	T	LUAD
TCGA-55-8506	T	LUAD
TCGA-05-4433	T	LUAD
TCGA-05-4382	T	LUAD
TCGA-50-5066	T	LUAD
TCGA-73-7498	T	LUAD
TCGA-95-7039	T	LUAD
TCGA-MP-A4TD	T	LUAD
TCGA-95-8039	T	LUAD
TCGA-97-7941	T	LUAD
TCGA-75-5147	T	LUAD
TCGA-MP-A4TK	T	LUAD
TCGA-55-A4DG	T	LUAD
TCGA-69-A59K	T	LUAD
TCGA-67-3772	T	LUAD
TCGA-50-8460	T	LUAD
TCGA-67-3774	T	LUAD
TCGA-69-7974	T	LUAD
TCGA-86-8585	T	LUAD
TCGA-55-6642	T	LUAD
TCGA-49-4505	T	LUAD
TCGA-L4-A4E6	T	LUAD
TCGA-78-7162	T	LUAD
TCGA-44-3396	T	LUAD
TCGA-78-8655	T	LUAD
TCGA-MP-A4T6	T	LUAD
TCGA-38-4629	T	LUAD
TCGA-49-AARO	T	LUAD
TCGA-95-A4VK	T	LUAD
TCGA-86-8673	T	LUAD
TCGA-86-8359	T	LUAD
TCGA-05-4425	T	LUAD
TCGA-73-4662	T	LUAD
TCGA-95-7567	T	LUAD
TCGA-50-7109	T	LUAD
TCGA-55-8085	T	LUAD
TCGA-69-7765	T	LUAD
TCGA-49-AAR0	T	LUAD
TCGA-49-AAQV	T	LUAD
TCGA-55-7903	T	LUAD
TCGA-44-A4SS	T	LUAD
TCGA-55-6712	T	LUAD
TCGA-55-6983	T	LUAD

Confidential: For Review Only

Garrido-Martin EM, *et al. J Immunother Cancer* 2020; 8:e000778. doi: 10.1136/jitc-2020-000778

TCGA-44-6145	T	LUAD
TCGA-49-4490	T	LUAD
TCGA-55-8511	T	LUAD
TCGA-83-5908	T	LUAD
TCGA-86-8672	T	LUAD
TCGA-05-5425	T	LUAD
TCGA-49-AAR2	T	LUAD
TCGA-55-7227	T	LUAD
TCGA-78-7153	T	LUAD
TCGA-78-7160	T	LUAD
TCGA-55-8092	T	LUAD
TCGA-L9-A7SV	T	LUAD
TCGA-49-6744	T	LUAD
TCGA-44-A4SU	T	LUAD
TCGA-55-6985	T	LUAD
TCGA-69-7761	T	LUAD
TCGA-55-7911	T	LUAD
TCGA-55-7574	T	LUAD
TCGA-55-6968	T	LUAD
TCGA-91-8497	T	LUAD
TCGA-55-7573	T	LUAD
TCGA-86-A4P7	T	LUAD
TCGA-75-5125	T	LUAD
TCGA-91-7771	T	LUAD
TCGA-86-8280	T	LUAD
TCGA-55-A491	T	LUAD
TCGA-97-7553	T	LUAD
TCGA-49-AARQ	T	LUAD
TCGA-78-7145	T	LUAD
TCGA-44-6777	T	LUAD
TCGA-55-8508	T	LUAD
TCGA-44-A479	T	LUAD
TCGA-50-8457	T	LUAD
TCGA-55-7914	T	LUAD
TCGA-05-4417	T	LUAD
TCGA-53-7626	T	LUAD
TCGA-64-5781	T	LUAD
TCGA-35-4122	T	LUAD
TCGA-69-7980	T	LUAD
TCGA-97-A4M0	T	LUAD
TCGA-50-6590	T	LUAD
TCGA-44-5645	T	LUAD
TCGA-86-A4P8	T	LUAD
TCGA-55-A48X	T	LUAD
TCGA-44-2668	T	LUAD
TCGA-50-5935	T	LUAD
TCGA-05-4398	T	LUAD
TCGA-S2-AA1A	T	LUAD
TCGA-53-A4EZ	T	LUAD

Confidential: For Review Only

TCGA-55-8299	T	LUAD
TCGA-NJ-A55A	T	LUAD
TCGA-05-4434	T	LUAD
TCGA-95-A4VN	T	LUAD
TCGA-MP-A4SV	T	LUAD
TCGA-55-8621	T	LUAD
TCGA-38-A44F	T	LUAD
TCGA-55-8205	T	LUAD
TCGA-55-8620	T	LUAD
TCGA-MP-A4T9	T	LUAD
TCGA-55-A48Z	T	LUAD
TCGA-44-6779	T	LUAD
TCGA-L4-A4E5	T	LUAD
TCGA-62-8402	T	LUAD
TCGA-86-7701	T	LUAD
TCGA-97-A4M2	T	LUAD
TCGA-44-A47G	T	LUAD
TCGA-44-2656	T	LUAD
TCGA-05-4422	T	LUAD
TCGA-44-6778	T	LUAD
TCGA-78-7539	T	LUAD
TCGA-50-8459	T	LUAD
TCGA-55-8203	T	LUAD
TCGA-86-7954	T	LUAD
TCGA-55-7284	T	LUAD
TCGA-97-8172	T	LUAD
TCGA-05-5420	T	LUAD
TCGA-49-AARE	T	LUAD
TCGA-99-7458	T	LUAD
TCGA-78-8648	T	LUAD
TCGA-55-6980	T	LUAD
TCGA-MP-A4SW	T	LUAD
TCGA-49-4514	T	LUAD
TCGA-05-4250	T	LUAD
TCGA-MN-A4N5	T	LUAD
TCGA-55-6969	T	LUAD
TCGA-93-A4JO	T	LUAD
TCGA-55-8089	T	LUAD
TCGA-55-6982	T	LUAD
TCGA-99-AA5R	T	LUAD
TCGA-44-3919	T	LUAD
TCGA-44-2661	T	LUAD
TCGA-69-7978	T	LUAD
TCGA-49-4507	T	LUAD
TCGA-64-5778	T	LUAD
TCGA-55-6978	T	LUAD
TCGA-86-8076	T	LUAD
TCGA-55-8619	T	LUAD
TCGA-49-6745	T	LUAD

Confidential: For Review Only

Garrido-Martin EM, *et al. J Immunother Cancer* 2020; 8:e000778. doi: 10.1136/jitc-2020-000778

TCGA-50-5941	T	LUAD
TCGA-44-7672	T	LUAD
TCGA-50-5045	T	LUAD
TCGA-MP-A4TJ	T	LUAD
TCGA-55-6971	T	LUAD
TCGA-78-7146	T	LUAD
TCGA-MP-A4TH	T	LUAD
TCGA-05-4389	T	LUAD
TCGA-44-2659	T	LUAD
TCGA-55-6979	T	LUAD
TCGA-69-8255	T	LUAD
TCGA-L9-A743	T	LUAD
TCGA-55-7728	T	LUAD
TCGA-L9-A50W	T	LUAD
TCGA-55-8510	T	LUAD
TCGA-55-A493	T	LUAD
TCGA-49-4487	T	LUAD
TCGA-95-7944	T	LUAD
TCGA-50-6594	T	LUAD
TCGA-93-A4JQ	T	LUAD
TCGA-55-7994	T	LUAD
TCGA-55-8301	T	LUAD
TCGA-73-7499	T	LUAD
TCGA-L9-A8F4	T	LUAD
TCGA-62-A46U	T	LUAD
TCGA-55-7995	T	LUAD
TCGA-35-4123	T	LUAD
TCGA-44-3918	T	LUAD
TCGA-78-8660	T	LUAD
TCGA-99-8028	T	LUAD
TCGA-55-8208	T	LUAD
TCGA-38-7271	T	LUAD
TCGA-MP-A4T4	T	LUAD
TCGA-50-5049	T	LUAD
TCGA-50-6597	T	LUAD
TCGA-91-6835	T	LUAD
TCGA-73-4666	T	LUAD
TCGA-55-8302	T	LUAD
TCGA-97-A4LX	T	LUAD
TCGA-50-5055	T	LUAD
TCGA-97-7552	T	LUAD
TCGA-44-2657	T	LUAD
TCGA-NJ-A4YQ	T	LUAD
TCGA-86-6851	T	LUAD
TCGA-97-A4M6	T	LUAD
TCGA-55-7281	T	LUAD
TCGA-55-6987	T	LUAD
TCGA-55-7907	T	LUAD
TCGA-49-AAR4	T	LUAD

Confidential: For Review Only

Supplemental material BMJ Publishing Group Limited (BMJ) disclaims all liability and responsibility arising from any reliance placed on this supplemental material which has been supplied by the author(s) J Immunother Cancer

TCGA-93-7347	T	LUAD
TCGA-49-AAR3	T	LUAD
TCGA-49-6767	T	LUAD
TCGA-MP-A4TI	T	LUAD
TCGA-L9-A444	T	LUAD
TCGA-91-A4BC	T	LUAD
TCGA-86-8671	T	LUAD
TCGA-49-6761	T	LUAD

Cohort is composed of n=511 but complete data of survival is only available from 495

Confidential: For Review Only

Garrido-Martin EM, *et al. J Immunother Cancer* 2020; 8:e000778. doi: 10.1136/jitc-2020-000778

UAD) retrospective cohort of The Cancer Genome Atlas TCGA

Dead/Alive	Combined survival	CXCL9	CXCR3	CD68
1	119	2.9205	1.9953	9.1914
0	652	9.5368	2.8502	11.3244
1	1454	7.0981	2.9428	9.5031
0	256	4.1324	3.0855	10.685
0	568	5.7571	3.1015	11.1608
0	842	9.1718	3.2581	8.2014
0	1189	6.7766	3.3737	9.163
1	434	7.2706	3.5524	10.7519
0	617	6.406	3.5838	10.0718
0	1617	8.9542	3.653	10.6602
1	62	5.5241	3.8539	12.8749
0	773	10.3887	3.9096	11.469
0	1148	7.8316	3.9374	10.9707
0	1072	7.0047	3.9782	10.3007
1	1528	6.5677	4.0979	11.1552
0	949	7.7417	4.1124	11.3032
0	131	8.3182	4.121	12.2707
1	260	12.3961	4.2402	9.9161
0	705	10.9751	4.2507	12.7836
1	1501	8.389	4.3563	12.4428
0	408	10.4352	4.3927	11.4915
1	711	7.536	4.4096	13.2189
0	889	6.1929	4.425	11.9446
0	186	9.6111	4.483	11.572
0	912	6.9395	4.4958	12.0484
1	1632	6.7891	4.504	8.2884
1	607	5.6019	4.5514	11.3159
1	625	7.8238	4.558	11.354
0	670	8.6038	4.6173	11.5912
0	119	11.2643	4.6354	10.9849
0	724	9.2293	4.643	11.341
0	462	6.6203	4.6686	11.4316
0	435	8.8662	4.6694	11.8339
1	1171	11.3088	4.694	10.2626
1	1379	6.321	4.7246	11.2039
0	1126	8.3916	4.7633	11.482
0	564	8.0276	4.8031	10.9801
0	806	8.2821	4.8632	10.8217
0	442	12.0511	4.8758	12.13
0	440	6.9214	4.9008	11.9612
1	561	9.1234	4.9198	10.2571
1	594	6.3073	4.9224	10.6181
1	1235	7.4283	4.9312	11.9965
1	250	9.0457	4.9336	13.631
1	503	7.7638	4.9442	10.2102
1	666	8.3752	4.9464	11.5677

Confidential: For Review Only

0	1301	8.0277	5.0139	12.2655
1	731	10.2977	5.0261	12.6113
1	275	6.8988	5.0316	11.7861
0	2676	8.0366	5.0365	9.7971
0	541	5.4369	5.0525	13.4242
0	2161	8.7861	5.0531	11.271
0	174	8.1386	5.0778	12.4616
0	1126	8.8521	5.0964	11.7114
1	18	9.8293	5.0973	11.2207
1	179	7.9414	5.1131	11.3156
0	2248	8.7246	5.1305	10.0022
0	62	7.0291	5.1438	13.3434
0	2616	7.5138	5.149	10.5531
0	1750	8.1953	5.1603	12.6569
0	1040	8.3971	5.1773	10.3879
1	4	5.7559	5.1931	12.8648
1	550	4.7756	5.1988	10.3976
0	84	5.1974	5.2222	11.7721
0	882	9.2636	5.228	11.0822
0	417	8.3877	5.2331	10.6311
0	2199	7.2561	5.2374	11.5697
1	354	9.9418	5.2435	13.2569
0	1246	8.5187	5.2459	12.3057
1	1492	4.97	5.2471	12.6929
1	976	9.8661	5.2598	10.3859
0	824	9.2541	5.2658	10.0934
1	593	9.9415	5.267	10.6509
0	14	8.9384	5.2738	11.8162
0	418	7.5355	5.2809	11.907
1	2318	7.1993	5.2818	10.1436
0	1097	9.9432	5.3203	11.3149
1	1046	9.1869	5.327	12.5104
0	910	7.4292	5.3358	12.0693
1	2681	8.4556	5.3407	10.5098
0	414	10.909	5.3501	10.4266
1	97	6.5546	5.3589	11.2558
1	38	8.7724	5.3641	12.048
0	540	6.9995	5.3738	10.7245
0	739	8.7076	5.3766	12.8219
0	1289	4.4221	5.388	11.9635
0	546	7.5537	5.389	11.3609
1	281	7.5852	5.3978	12.4988
1	1498	8.5171	5.4	11.2857
1	807	9.2329	5.4159	13.0062
0	551	8.0076	5.4175	11.8741
1	598	8.8978	5.4302	12.1836
1	1258	7.7289	5.4545	12.3871
0	287	10.4461	5.4592	11.5422
1	340	10.166	5.4638	11.316

Confidential: For Review Only

0	467	9.3883	5.4691	12.0466
1	3361	8.9408	5.4815	10.5866
0	603	8.1417	5.4865	12.7838
1	303	8.8844	5.491	10.9861
1	1073	10.2667	5.509	10.8582
0	230	8.2862	5.5121	11.0744
0	1060	9.071	5.5231	10.7477
0	863	7.0589	5.5305	10.4466
1	922	5.6243	5.5307	12.5618
0	1280	9.8919	5.5645	13.4093
1	0	10.0571	5.568	12.3253
0	448	8.4316	5.5692	12.1457
0	536	10.224	5.5704	10.7605
0	657	8.4556	5.5768	12.3197
0	426	8.0622	5.5806	11.7352
0	3940	8.5545	5.5811	11.015
0	1157	8.6104	5.5818	11.529
0	610	10.12	5.5847	12.5521
1	701	9.4255	5.5878	12.9018
1	760	5.6542	5.6009	11.6
1	124	9.9604	5.6022	12.3999
1	58	8.8829	5.6207	11.6867
0	36	12.2577	5.6277	12.1221
0	977	8.5401	5.6449	12.6651
1	118	7.3243	5.6569	11.4903
0	592	10.3667	5.6618	11.0295
1	321	10.2907	5.6622	11.6757
0	44	7.7687	5.6631	11.2289
1	896	8.3146	5.667	11.4584
0	3674	8.0719	5.6787	14.789
0	653	11.0586	5.6836	10.9084
0	630	9.7618	5.6989	11.0723
0	2696	10.4316	5.7223	12.6253
0	505	7.6496	5.7248	13.8185
0	791	9.8888	5.727	12.03
1	116	9.7038	5.7311	10.7146
0	578	9.8152	5.7512	13.2545
0	448	8.648	5.756	11.6741
1	1622	7.0431	5.7586	11.2899
0	791	7.7058	5.7721	12.6442
1	22	8.1452	5.7772	11.9816
1	257	8.6231	5.7838	12.76
0	426	7.8949	5.791	12.781
0	285	11.0331	5.7931	12.6515
1	1043	11.4816	5.816	10.873
1	488	6.4996	5.8311	12.7799
1	808	8.3214	5.8386	11.818
1	210	8.1918	5.8435	11.2745
1	468	6.6736	5.8452	12.229

Confidential: For Review Only

BMJ Publishing Group Limited (BMJ) disclaims all liability and responsibility arising from any reliance placed on this supplemental material which has been supplied by the author(s)

0	139	8.8852	5.862	11.8702
0	435	9.1252	5.8682	13.1286
0	424	8.9553	5.8717	9.2168
1	87	12.4908	5.8802	11.5743
1	291	7.1766	5.884	9.2
0	1163	8.7813	5.8886	12.9899
0	264	11.3102	5.9026	11.7475
0	2065	9.2741	5.9118	12.2945
0	896	9.9649	5.9245	12.7173
1	694	7.9212	5.9305	12.2129
1	628	11.9746	5.9535	12.5578
0	7248	6.8712	5.9608	8.8924
0	446	6.2454	5.9657	11.0771
1	460	9.2029	5.9674	13.4044
0	704	6.8468	5.9682	11.5843
1	1081	10.3308	5.9685	11.9585
0	610	7.7661	5.9838	11.8697
1	91	10.2025	5.9925	11.5151
1	300	10.7319	6.0221	11.3324
1	949	4.1746	6.0296	11.2834
0	476	7.4017	6.0328	10.905
1	244	12.3307	6.0444	12.072
0	1216	8.5747	6.0521	11.4552
0	690	7.3167	6.0577	12.1712
1	574	11.0805	6.0647	12.602
0	2488	8.9263	6.0943	12.6458
1	193	11.0814	6.0957	10.3467
0	791	8.6362	6.1137	14.0828
0	866	8.6665	6.1157	13.4697
0	691	9.0543	6.1243	11.524
0	323	12.2953	6.1298	12.7026
0	888	7.64	6.1496	12.9769
1	1147	8.3458	6.1507	12.9037
0	596	7.8165	6.1531	11.7211
0	260	7.452	6.1572	11.4641
0	913	10.0079	6.1607	13.1415
0	2067	10.0338	6.1716	11.5869
1	478	4.5992	6.1752	10.1854
0	1324	8.3256	6.1897	11.7617
1	258	9.7379	6.203	10.8977
1	376	8.2623	6.2074	12.3127
0	603	6.9433	6.2104	11.1931
0	3261	8.4385	6.2313	11.8908
0	466	10.2594	6.2328	12.2448
1	282	10.8458	6.2359	12.7854
0	2368	9.1151	6.2368	11.3956
1	1516	8.5056	6.2446	12.4264
1	737	10.3586	6.2452	12.3554
0	1974	8.8744	6.2466	10.7211

Confidential: For Review Only

Garrido-Martin EM, et al. J Immunother Cancer 2020; 8:e000778. doi: 10.1136/jitc-2020-000778

BMJ Publishing Group Limited (BMJ) disclaims all liability and responsibility arising from any reliance placed on this supplemental material which has been supplied by the author(s)

0	1079	10.7568	6.249	11.6474
0	24	11.1578	6.2557	11.7363
0	1847	9.0031	6.2682	10.5348
0	600	11.649	6.2696	12.2341
0	740	8.7457	6.2724	11.9351
1	1167	10.3796	6.2851	11.7555
1	719	8.9421	6.2859	13.1347
0	1965	9.1941	6.2941	11.9116
1	1798	8.6284	6.3036	12.7614
1	336	8.3065	6.3084	13.0838
0	48	9.98	6.3085	11.0031
1	336	10.6842	6.3093	11.3396
0	515	10.9129	6.3097	12.0468
1	139	9.1524	6.3146	11.2031
0	13	9.658	6.3282	13.1639
0	141	8.6029	6.3426	12.5401
0	718	8.6258	6.3584	10.8663
0	1523	8.9358	6.3712	11.9628
0	578	10.0682	6.3801	13.4365
1	905	7.8839	6.3925	12.4804
0	2595	12.2229	6.4123	12.6275
0	1621	10.4027	6.4203	12.6426
1	950	8.5923	6.4302	12.2711
0	610	12.5927	6.4428	12.8148
1	167	9.4642	6.4505	12.4665
0	481	4.7399	6.4508	9.8904
0	4992	8.2141	6.4553	12.4964
1	656	10.8186	6.4612	11.462
0	1013	10.9489	6.4647	10.8069
0	310	11.0909	6.4754	11.8415
0	705	9.1366	6.4812	13.2278
1	999	10.756	6.4814	12.7528
1	1178	10.9292	6.4829	11.9239
1	244	8.1693	6.4884	12.444
0	3305	7.697	6.4899	11.9831
0	775	9.8145	6.4926	12.2656
1	2393	11.6951	6.4966	13.1821
0	7062	10.1022	6.5148	12.0537
0	1175	8.0142	6.5205	11.4543
1	1600	10.4797	6.5311	13.0389
0	864	10.0597	6.5338	9.9836
0	605	9.6388	6.535	11.2576
1	189	11.5047	6.5386	12.4113
0	728	7.8156	6.5401	12.125
0	997	8.3434	6.5419	12.2211
0	930	12.4727	6.557	12.1788
0	422	11.2748	6.5583	11.6555
0	218	10.5463	6.5761	12.2442
0	827	5.4251	6.5872	10.1961

Confidential: For Review Only

Garrido-Martin EM, *et al. J Immunother Cancer* 2020; 8:e000778. doi: 10.1136/jitc-2020-000778

0	670	9.9428	6.5896	12.2405
0	1479	11.139	6.5929	11.5209
1	164	10.9002	6.5931	11.8805
0	1189	11.4578	6.6041	10.5611
0	634	9.1968	6.6414	12.9135
1	274	11.3175	6.6445	13.5822
0	626	8.7409	6.6495	13.1362
0	476	8.9075	6.6764	11.1482
0	520	7.6557	6.6765	11.9872
0	499	8.2604	6.6767	13.532
0	658	9.2343	6.6767	12.1271
0	35	9.7515	6.7066	12.8693
0	845	10.5284	6.7342	11.437
0	60	10.6105	6.7381	12.7416
0	427	11.7554	6.7396	12.2995
0	1728	10.7929	6.7439	11.6045
0	531	9.9049	6.7459	11.3317
1	869	11.0889	6.7469	11.4534
0	761	10.1288	6.7487	12.5596
1	626	9.6859	6.7605	11.6134
0	1285	10.0863	6.7618	11.6006
0	477	10.9749	6.7625	11.5828
0	385	11.0229	6.7696	11.3023
0	944	9.4757	6.7698	11.903
0	601	8.6524	6.7834	12.6116
0	629	10.9764	6.8002	11.9983
1	1421	9.7199	6.8035	11.994
0	151	7.8336	6.8119	13.0466
1	1357	10.3428	6.8156	12.3754
1	464	11.7663	6.8257	12.8689
1	1194	9.785	6.8301	12.5033
0	938	9.9183	6.8302	11.7845
1	1653	9.0761	6.8398	11.9351
1	557	10.8699	6.8493	12.8688
1	777	12.2206	6.8548	13.2534
0	423	10.3169	6.862	11.9328
0	372	11.3045	6.8626	11.3137
0	409	11.0971	6.8722	11.8728
0	761	11.0657	6.8769	12.7046
1	161	5.6356	6.8898	10.5469
0	988	8.9152	6.8903	11.5503
1	1197	7.0756	6.8986	12.7094
0	609	9.8569	6.9105	11.6307
1	1499	11.5811	6.9154	12.043
1	614	11.5982	6.9213	12.2823
0	813	11.2744	6.9348	14.6181
1	74	12.3116	6.9363	12.0534
0	2973	10.2242	6.9484	13.2529
1	1215	9.8545	6.9491	11.4195

Confidential: For Review Only

1	624	10.4482	6.9758	11.4551
1	4961	7.4994	6.98	12.0098
1	1135	8.9557	6.9815	11.0668
1	586	11.09	6.984	11.2942
0	202	5.5803	6.9906	10.2966
0	50	11.7649	7.0032	11.5849
0	224	12.5549	7.0033	12.7611
0	11	10.3191	7.0092	11.9602
0	730	10.9742	7.0123	13.6415
0	607	11.7174	7.0195	13.7568
0	1442	12.6111	7.0215	12.8528
0	1189	9.3568	7.0275	11.4397
0	1272	11.348	7.0413	11.7859
1	307	8.2057	7.0562	11.3343
0	830	9.68	7.0614	11.9395
0	484	9.3512	7.069	11.9219
0	1333	9.3503	7.0724	12.1882
1	582	10.7113	7.0739	12.8021
0	608	10.6142	7.0933	11.1163
0	591	11.6066	7.0972	11.6968
0	573	8.6801	7.1005	12.3226
0	829	9.4586	7.1013	12.4811
0	385	10.9332	7.1118	12.4259
0	184	11.8735	7.1265	13.0351
0	353	12.4824	7.1401	13.0364
0	2449	10.5063	7.1486	11.7711
1	428	11.7994	7.1629	12.7282
0	435	9.9982	7.1693	15.0999
1	3169	10.4294	7.1706	11.7011
0	1130	11.5311	7.1787	12.6729
0	2360	10.6434	7.1901	12.3846
1	1790	9.7277	7.1963	11.6615
1	864	12.1617	7.1968	13.8616
0	3759	10.2748	7.1983	12.4678
0	651	10.5009	7.2037	11.1444
0	862	9.1094	7.2172	11.4578
1	444	9.3733	7.2215	11.2438
0	669	12.0882	7.222	12.5901
0	2515	10.1298	7.2432	12.2548
0	568	11.243	7.2617	11.9188
1	308	11.032	7.2683	11.4584
0	904	12.4804	7.2709	12.1348
0	165	10.1304	7.2836	12.1653
0	4765	10.1464	7.2916	12.6934
1	677	9.5631	7.2929	11.251
0	567	10.8833	7.3169	11.3575
0	415	13.6443	7.3215	12.5289
1	171	12.4604	7.3348	12.6092
0	2823	9.652	7.3398	11.6916

Confidential: For Review Only

0	595	12.11	7.3406	12.4337
1	385	9.0134	7.3565	11.7048
0	552	11.4726	7.3613	12.2841
0	824	14.028	7.3672	12.5915
1	19	12.1883	7.3745	13.1162
0	882	13.0641	7.3764	13.3274
0	2224	10.5797	7.3821	13.0995
1	952	11.3649	7.3944	12.5415
0	3635	7.8225	7.3978	10.9941
1	697	12.0819	7.3994	12.9648
1	154	12.4306	7.4136	11.9766
0	565	9.9574	7.4441	10.0491
0	1683	10.2592	7.4565	12.863
1	409	10.6137	7.4606	10.8125
0	1233	12.6434	7.4746	11.9233
0	186	13.562	7.4845	12.4657
0	537	11.2775	7.5228	12.0538
1	995	10.7618	7.5265	12.0635
1	1293	13.1612	7.5338	12.6792
1	434	8.423	7.5366	12.8506
0	487	11.0386	7.5388	11.5248
0	415	8.6454	7.5392	12.8706
1	2027	12.966	7.5396	12.59
0	492	11.0717	7.5447	12.6715
0	701	12.0637	7.545	12.4052
0	626	10.878	7.5617	12.201
0	1870	10.2729	7.5755	14.0826
0	6732	11.4106	7.5766	9.7187
1	826	7.0875	7.5796	11.947
1	987	10.5151	7.5806	14.1155
0	617	8.0325	7.5915	11.5129
0	486	13.2719	7.6453	12.3892
0	1125	10.8579	7.6459	12.0727
1	187	10.8792	7.6503	10.6218
0	455	11.4632	7.6566	12.2452
1	929	11.2876	7.6594	12.6004
0	1559	11.3175	7.6648	12.1907
0	225	11.1756	7.6796	13.3836
0	411	11.9987	7.6833	12.0848
0	652	10.0534	7.6917	11.9815
1	1288	13.1597	7.7122	13.0899
0	852	9.7286	7.7171	11.8232
0	805	10.4913	7.725	13.728
0	689	10.791	7.7256	11.3715
1	761	11.9335	7.7278	12.82
1	653	11.6777	7.7476	11.6347
0	1431	11.4627	7.7613	12.6063
0	513	11.4941	7.763	11.9562
0	1071	9.0904	7.7745	10.7537

Confidential: For Review Only

Garrido-Martin EM, et al. J Immunother Cancer 2020; 8:e000778. doi: 10.1136/jitc-2020-000778

1	469	11.9603	7.7827	12.8621
0	15	11.4459	7.7899	11.1088
1	457	12.0338	7.7908	13.6438
0	553	12.269	7.8208	12.2123
1	2620	11.7675	7.8222	12.0046
0	515	11.1019	7.8303	13.9348
0	133	11.5681	7.8303	12.7144
0	599	13.2655	7.8343	13.6459
1	375	11.9157	7.8505	11.8804
1	1265	9.544	7.8579	12.6509
0	651	10.9498	7.8737	11.7453
1	500	13.0716	7.893	12.8338
0	578	9.7011	7.8981	10.3646
1	1498	13.5008	7.9006	12.2542
0	947	9.7531	7.9017	11.3985
0	624	9.7821	7.9033	13.6508
0	351	12.1249	7.9159	12.8566
0	1429	12.8015	7.9166	13.4033
0	365	11.1493	7.9176	12.2978
0	1864	13.2291	7.9233	13.9429
0	791	11.3019	7.936	12.2727
0	1119	8.9547	7.9363	13.7355
0	547	10.3487	7.9382	11.4039
0	605	13.2669	7.9526	12.7895
1	243	9.8185	7.9551	13.0956
0	545	10.9304	7.9643	12.4284
0	457	13.5852	7.9699	13.6321
1	1229	11.2058	7.9813	11.1184
0	747	11.8361	7.992	12.2807
1	1209	10.7773	7.9923	14.2326
0	2109	10.0417	8.0079	12.0209
1	1778	8.5326	8.0103	12.4858
0	1700	11.0206	8.0244	11.2703
1	121	11.7447	8.0441	12.5684
0	84	11.1006	8.0444	11.5869
0	1239	11.2979	8.0455	12.4297
1	33	11.5955	8.0562	12.1137
1	702	14.4775	8.0614	13.446
1	995	11.1882	8.0778	12.8441
0	658	10.8769	8.0812	12.6199
1	1026	12.0083	8.0817	12.5384
0	1159	12.0046	8.1444	13.2227
0	134	12.3381	8.1572	13.4948
1	268	12.1871	8.1652	11.8012
0	1305	13.2848	8.1698	12.1472
1	176	14.0599	8.1813	12.9457
0	993	10.7622	8.183	12.1648
0	416	9.6175	8.188	13.8249
0	522	10.8534	8.1991	12.8815

Confidential: For Review Only

0	1474	13.2737	8.2305	12.9023
0	719	11.637	8.2368	13.039
1	2174	11.4475	8.2373	13.4716
1	339	12.4932	8.2523	12.5889
0	1400	11.9454	8.2571	13.2166
1	173	9.8974	8.2713	11.655
0	741	10.3152	8.2742	11.7917
0	1369	11.5938	8.2857	12.4425
0	1367	11.0649	8.3029	11.7747
1	237	13.5438	8.3102	13.3908
0	129	12.7263	8.3443	13.1601
0	664	11.448	8.3501	12.5781
0	704	10.1531	8.3515	14.3938
1	442	9.4556	8.3614	10.9463
0	539	11.4262	8.3754	13.3554
0	28	11.679	8.3797	12.1426
1	855	12.2453	8.408	12.0656
0	377	13.1159	8.4215	12.4752
1	370	7.4391	8.4231	13.1074
0	526	11.6073	8.4465	11.3137
0	603	13.6539	8.4741	13.0853
0	534	13.4036	8.4803	12.8958
1	1531	13.1286	8.5322	12.055
0	476	12.1309	8.5343	12.4998
0	2067	13.9121	8.5349	13.7161
0	889	13.6416	8.5376	13.2541
0	182	11.6711	8.5393	12.5239
0	1036	13.7828	8.5673	13.0003
1	321	12.1068	8.6011	12.7405
0	1118	13.6144	8.6275	13.5001
0	674	13.2	8.6556	13.4689
1	800	11.9427	8.6589	12.7025
1	2617	12.9757	8.674	12.7578
0	3094	14.2278	8.7088	13.6345
1	1268	11.3949	8.7253	11.2483
0	79	12.6019	8.7326	13.3513
0	800	14.0222	8.7686	13.8397
0	478	10.476	8.8301	12.4776
0	614	12.7517	8.8436	13.1829
1	1830	11.3739	8.862	12.3021
0	1932	12.1067	8.8643	12.6786
0	1351	12.4645	9.0049	12.8262
0	1432	12.7723	9.0053	12.7439
0	179	14.0864	9.0079	12.5594
0	568	11.2017	9.0488	12.4617
0	872	11.2986	9.0647	12.6282
0	2137	12.3636	9.0773	13.1273
1	343	12.8134	9.1273	12.3212
1	879	11.4335	9.1509	11.7011

Confidential: For Review Only

0	683	12.1851	9.2893	12.7996
0	1893	13.5448	9.4085	13.1619
0	677	12.6369	9.4088	11.8166
1	429	12.6744	9.414	13.4081
0	307	14.1091	9.4721	12.346
0	44	12.4008	9.5003	12.2931
0	839	13.5499	9.9864	13.263
0	354	11.675	10.9578	11.708

Confidential: For Review Only

CD8A	CD8B
1.9953	4.1478
6.7479	3.0177
5.6129	4.0045
2.8561	1.3562
3.7696	2.2176
7.4937	3.035
7.3736	5.1175
5.8216	7.2104
5.3025	2.8897
6.8598	6.8438
5.9055	4.1463
7.5953	4.2507
6.391	4.5837
6.6057	3.4908
4.9649	2.8746
5.2605	3.3058
6.4401	4.2301
7.2992	4.9027
8.6359	6.1526
5.7028	3.3351
6.222	4.0327
6.2933	4.6099
5.7947	3.924
6.2837	3.93
6.6382	6.2716
5.6789	4.1798
7.1723	7.6232
6.4507	4.237
7.5246	6.2806
8.319	6.0474
6.1273	3.5732
5.4799	3.9032
6.7354	4.9486
7.6855	5.5459
5.3698	2.836
6.7127	4.6817
5.3063	3.1756
5.689	3.8
8.6707	6.5131
5.4009	3.6084
6.7592	4.4748
6.5923	4.6473
5.8251	3.4562
6.5162	4.9553
7.4642	4.2501
7.0977	4.973

Confidential: For Review Only

Garrido-Martin EM, et al. J Immunother Cancer 2020; 8:e000778. doi: 10.1136/jitc-2020-000778

6.7577	4.542
6.9364	5.2351
6.5155	5.5191
6.8882	9.0682
5.3761	3.6916
6.3963	4.3099
5.9031	3.2006
6.3023	6.653
8.1363	6.0889
5.7015	4.2673
4.6478	2.4763
6.9494	4.5633
6.5931	5.0894
6.5499	4.6664
5.6724	4.2167
5.0831	3.3784
6.6507	4.4401
5.7351	3.781
7.5309	7.7056
7.7654	5.2666
4.2377	2.576
7.5827	6.6193
6.0503	4.8122
5.1348	8.9957
6.5778	4.3855
8.1794	8.7821
7.2889	4.7006
7.4308	5.9636
6.9648	4.7822
6.9813	5.1394
6.402	3.5009
7.9031	5.8082
7.8271	5.5078
6.6561	4.7942
8.6253	6.1286
7.9365	5.4716
8.1632	5.7982
6.3384	3.7703
7.5743	5.4894
5.6621	3.8216
6.4896	4.6827
7.9386	5.4683
7.8585	5.1769
8.0585	7.0016
6.1898	4.356
6.7361	4.5612
7.7464	5.4201
9.0348	6.2941
7.9665	5.213

Confidential: For Review Only

7.3968	4.8105
6.6092	4.5135
6.7543	4.8606
6.1447	3.7958
7.6893	5.963
7.5688	8.1499
6.3394	3.9382
6.8251	5.5305
6.1437	4.7561
6.2421	4.1308
7.649	5.3855
8.0275	6.0903
7.7422	4.9911
5.2965	3.1144
6.4477	4.7851
6.702	4.7504
5.5525	4.4701
7.6114	6.2155
6.9209	4.7247
6.4717	4.5197
7.0368	5.1928
7.95	5.7672
7.8468	5.4827
7.9521	5.6862
7.617	5.0379
6.7412	7.9729
7.7211	5.6382
7.2177	5.6293
6.9828	6.6481
7.8871	5.735
7.8811	6.9886
7.0817	5.1775
7.3698	5.0236
7.7095	6.0455
8.2053	6.2125
7.2501	4.3606
7.0287	4.3276
6.3038	3.834
7.898	5.7961
6.7589	4.8658
6.021	3.4719
6.5963	4.0944
6.856	4.4032
7.9341	5.7158
7.4323	6.5013
6.7797	4.4515
8.0708	6.1473
6.0734	4.3377
7.0227	4.8553

Confidential: For Review Only

7.1603	5.3272
6.647	4.3185
9.2268	7.5471
7.935	5.3074
6.8396	4.5492
8.1523	6.7953
9.3886	7.4533
7.5734	5.5048
8.0095	5.0417
5.7042	4.1113
8.7868	7.7488
7.6334	5.3835
4.5846	2.9913
6.0884	4.2833
7.2125	8.4435
8.7417	7.171
7.9969	5.2267
8.7567	5.9824
7.1542	5.632
6.4312	4.0868
6.8182	5.1909
7.8977	5.3992
6.9314	4.9866
7.1645	4.7234
8.3189	5.7149
5.1273	3.553
8.6529	5.8082
6.867	4.5848
7.1617	4.5233
7.1749	4.4546
8.9467	6.2935
6.351	4.6222
7.306	5.762
6.2272	4.8771
7.1094	4.0857
8.3257	6.639
7.4544	5.552
7.1495	5.1005
7.0921	4.8172
6.8966	4.5959
6.1432	3.9007
6.0417	3.3407
7.3428	4.7636
7.2718	5.3181
9.1193	6.5743
7.9807	6.647
6.2594	3.833
8.1811	6.0175
7.5781	7.743

Confidential: For Review Only

8.3411	6.0829
7.3542	4.6612
8.0742	5.722
7.1038	4.4099
7.4015	5.1995
5.9811	3.6514
7.565	5.8751
6.9075	5.0423
6.6306	4.6394
6.3544	4.1953
7.648	5.9026
7.449	5.467
7.993	5.729
6.6672	7.5556
6.8426	4.6393
8.1905	6.3215
6.6957	4.445
6.9276	5.3773
7.1322	5.3291
5.5345	2.9851
7.0922	5.261
7.8856	5.5247
8.8286	6.824
8.5329	6.4643
7.2508	4.9945
4.8768	7.2996
8.5877	6.9539
9.3404	7.1219
8.2214	5.9637
6.8579	7.9982
7.1936	4.9202
10.3526	9.1142
7.5941	9.3607
6.9463	4.8812
8.5539	6.1568
7.9549	5.9196
7.5837	5.0728
7.9667	5.4267
7.7501	5.6715
8.4664	6.0194
8.6598	6.3433
8.634	7.1065
8.169	5.8316
6.7781	4.5858
7.8545	5.5663
8.209	6.1673
9.129	6.9783
7.9429	5.7933
4.9695	2.8545

Confidential: For Review Only

Garrido-Martin EM, *et al. J Immunother Cancer* 2020; 8:e000778. doi: 10.1136/jitc-2020-000778

8.4702	6.0867
8.4188	6.4741
7.7284	5.4039
8.4603	6.7284
7.1122	4.9417
8.9393	7.0798
7.5037	4.9485
6.9669	5.2717
5.8362	3.3266
8.3033	6.1509
6.826	4.2919
8.4011	5.9584
8.2178	5.9589
8.8622	6.562
8.7083	6.9817
8.9349	6.7308
7.8299	4.9411
9.0679	7.2564
8.2556	6.1789
8.8928	6.4629
8.776	6.3649
8.0649	5.9498
7.4843	4.6434
6.7491	4.8321
7.5355	5.9471
8.2559	6.0974
7.9818	6.53
7.9407	6.6211
8.5996	6.8351
8.5305	5.9095
8.7866	6.1121
7.7624	5.4544
8.5005	7.2721
8.0978	5.6448
9.8472	7.187
7.383	5.2556
8.9322	6.9455
7.9095	5.1377
8.5163	7.1734
5.4119	2.6914
8.3625	5.5764
6.4995	4.2651
8.4172	5.8984
9.7169	8.4222
8.8928	7.4912
8.6337	6.3122
8.0955	5.5808
8.5807	5.7758
8.7102	6.2163

Confidential: For Review Only

8.9701	6.4082
6.7614	5.0717
7.667	5.9587
8.2209	6.7576
5.312	2.0437
8.0175	6.1727
9.1729	6.5149
7.5058	5.296
8.7479	6.2828
7.411	5.2694
10.3138	9.6245
7.0778	4.9533
8.0745	5.9805
7.1392	5.0884
7.361	4.9022
7.7404	5.4061
7.9549	6.0091
7.8782	6.2903
8.7333	6.5108
8.7898	6.0486
7.3361	5.8839
7.9626	6.3495
7.9773	5.6118
8.1152	5.9017
9.2124	7.1625
8.8991	7.1285
9.1952	7.5838
7.7528	5.5425
8.6246	6.531
9.4379	7.1876
8.6665	6.8052
7.7512	5.4534
9.4195	7.3781
8.2409	6.4509
8.4828	6.175
8.5033	6.4368
10.5685	8.1534
7.6333	5.6019
8.5477	6.5266
8.4446	6.0065
8.8716	6.2605
9.2834	7.0501
8.0837	5.5021
8.1094	5.9767
7.8898	6.1996
8.7632	6.0825
9.2294	6.9211
10.2819	8.2561
8.9082	6.5802

Confidential: For Review Only

10.4305	7.791
6.848	5.789
8.8618	6.4394
10.6927	8.759
9.4884	7.2747
9.807	8.9088
8.094	5.856
8.0979	5.9173
7.9462	5.5934
9.8597	7.3994
10.1065	8.056
7.0799	4.6081
9.4185	7.25
7.0698	4.9782
8.7783	6.4023
9.9294	7.1149
9.9155	7.1935
9.6388	7.4168
8.7796	6.6525
7.636	5.6623
7.9796	5.3303
9.0276	6.936
8.9893	6.0545
9.5143	6.8774
8.2895	6.2036
7.7473	7.6055
9.4124	7.3806
7.8816	8.3173
5.1897	2.343
7.7095	5.3193
7.3073	5.47
9.968	7.1472
8.8824	7.0534
8.8211	6.6997
8.8367	6.858
9.651	7.0709
10.4882	8.6451
9.7812	8.3269
8.8397	6.6021
9.2011	7.0436
9.2312	6.9958
7.8521	5.6536
9.6005	7.2893
8.5985	6.3075
9.5757	6.9458
7.9981	5.5883
8.3339	5.8949
8.7748	6.585
7.3565	5.3758

Confidential: For Review Only

10.4172	8.273
8.5977	6.2514
8.4747	6.6475
9.6505	7.5662
9.3126	7.0094
8.517	5.8391
8.3401	6.4833
10.1244	7.9661
8.6605	6.6327
7.7868	5.6775
8.1717	6.4028
9.9315	7.6456
7.5159	4.9768
9.1259	9.1931
9.3304	6.4145
9.1893	6.9799
9.829	7.1494
10.496	8.8024
10.1199	8.2226
9.8727	7.2176
9.4463	7.5436
7.9849	5.9539
8.7978	6.9722
8.5377	7.1769
8.0965	6.2081
8.6733	6.6811
9.741	8.2242
7.9365	5.7373
9.575	7.327
10.1817	8.0137
7.6306	5.6586
7.4089	5.3278
8.7819	7.1
9.9113	7.5674
9.329	6.9384
9.1315	6.8039
9.2272	7.3219
10.5624	7.8975
7.4147	5.7674
9.6293	7.7068
9.1099	7.4511
9.7907	8.3259
9.773	7.4774
10.6705	8.2868
10.7756	8.8641
10.6161	8.4426
10.0969	8.2022
9.0462	7.3886
9.4148	6.7368

Confidential: For Review Only

10.0181	7.4803
8.868	6.5048
9.481	7.7513
10.1335	8.0197
10.3594	7.6881
7.8162	4.8757
8.8597	6.956
10.2236	8.4345
9.5117	7.4772
9.8298	7.7544
10.5609	8.1381
9.2166	7.2754
8.2088	6.2447
6.0827	3.8953
9.2203	7.0373
8.8862	6.6282
10.0429	7.1972
10.8339	8.2448
6.1865	2.5679
8.2785	6.0623
9.7072	7.7964
9.4844	7.6541
10.438	8.6894
9.4918	7.4303
9.0884	6.2828
10.0194	8.4575
11.6923	9.989
10.0747	8.4112
9.2443	6.9068
10.6883	8.1841
9.5071	7.7275
10.4978	8.2833
10.1841	7.7062
10.9426	9.2025
7.8037	6.6569
10.3606	8.3864
10.1458	8.24
8.1994	5.6361
9.6727	7.088
10.0987	8.176
10.9796	7.8817
10.4646	8.6108
10.1734	8.3299
9.8722	7.4793
7.842	6.3792
8.4095	5.8856
10.7359	8.1703
8.6764	6.8376
11.6511	9.3425

Confidential: For Review Only

Supplemental material
BMJ Publishing Group Limited (BMJ) disclaims all liability and responsibility arising from any reliance placed on this supplemental material which has been supplied by the author(s)

10.0839	7.9319
10.5799	8.2236
9.3511	6.6153
9.6733	7.3798
10.9855	8.7147
11.6524	9.7272
10.2771	8.2742
9.035	6.1725

Confidential: For Review Only

Garrido-Martin EM, et al. J Immunother Cancer 2020; 8:e000778. doi: 10.1136/jitc-2020-000778

Supplemental material BMJ Publishing Group Limited (BMJ) disclaims all liability and responsibility arising from any reliance placed on this supplemental material which has been supplied by the author(s) J Immunother Cancer

Supplementary Table 6: Proportion of CD103 presence in M1 hot, M1 interm, M1

Anonimized ID	CD103 score	CXCL9 score
LUAD-2011-365	3	1
LUAD-2011-422	3	1
LUAD-2011-455	3	1
LUAD-2007-030	3	1
LUAD-2011-379	3	1
LUAD-2009-151	2	1
LUAD-2010-258	2	1
LUAD-2011-366	2	1
LUAD-2007-017	2	1
LUAD-2007-051	2	1
LUAD-2008-080	2	1
LUAD-2009-222	2	1
LUAD-2010-259	2	1
LUAD-2011-418	2	1
LUAD-2011-452	2	1
LUAD-2009-205	2	1
LUAD-2010-261	2	1
LUAD-2010-297	2	1
LUAD-2007-021	2	1
LUAD-2007-060	1	1
LUAD-2008-071	1	1
LUAD-2009-232	1	1
LUAD-2010-250	1	1
LUAD-2010-274	1	1
LUAD-2010-343	1	1
LUAD-2011-368	1	1
LUAD-2007-001	1	1
LUAD-2007-031	1	1
LUAD-2007-041	1	1
LUAD-2008-062	1	1
LUAD-2008-067	1	1
LUAD-2008-078	1	1
LUAD-2008-089	1	1
LUAD-2008-102	1	1
LUAD-2008-115	1	1
LUAD-2008-116	1	1
LUAD-2008-125	1	1
LUAD-2008-126	1	1
LUAD-2008-127	1	1
LUAD-2008-138	1	1
LUAD-2009-148	1	1
LUAD-2009-152	1	1
LUAD-2009-160	1	1
LUAD-2009-161	1	1
LUAD-2009-165	1	1
LUAD-2009-168	1	1

Garrido-Martin EM, *et al. J Immunother Cancer* 2020; 8:e000778. doi: 10.1136/jitc-2020-000778

LUAD-2009-177	1	1
LUAD-2009-182	1	1
LUAD-2009-192	1	1
LUAD-2009-193	1	1
LUAD-2009-197	1	1
LUAD-2009-201	1	1
LUAD-2009-210	1	1
LUAD-2009-212	1	1
LUAD-2009-214	1	1
LUAD-2009-215	1	1
LUAD-2009-223	1	1
LUAD-2009-224	1	1
LUAD-2010-237	1	1
LUAD-2010-238	1	1
LUAD-2010-243	1	1
LUAD-2010-249	1	1
LUAD-2010-254	1	1
LUAD-2010-266	1	1
LUAD-2010-268	1	1
LUAD-2010-275	1	1
LUAD-2010-295	1	1
LUAD-2010-301	1	1
LUAD-2010-309	1	1
LUAD-2010-319	1	1
LUAD-2010-326	1	1
LUAD-2010-328	1	1
LUAD-2010-329	1	1
LUAD-2010-337	1	1
LUAD-2010-339	1	1
LUAD-2010-340	1	1
LUAD-2010-342	1	1
LUAD-2011-364	1	1
LUAD-2011-369	1	1
LUAD-2011-372	1	1
LUAD-2011-380	1	1
LUAD-2011-381	1	1
LUAD-2011-382	1	1
LUAD-2011-384	1	1
LUAD-2011-393	1	1
LUAD-2011-394	1	1
LUAD-2011-397	1	1
LUAD-2011-403	1	1
LUAD-2011-405	1	1
LUAD-2011-411	1	1
LUAD-2011-412	1	1
LUAD-2011-416	1	1
LUAD-2011-445	1	1
LUAD-2011-449	1	1
LUAD-2011-456	1	1

LUAD-2011-459	1	1
LUAD-2007-010	3	2
LUAD-2007-013	3	2
LUAD-2007-018	3	2
LUAD-2007-020	3	2
LUAD-2007-052	3	2
LUAD-2008-139	3	2
LUAD-2009-155	3	2
LUAD-2009-172	3	2
LUAD-2009-176	3	2
LUAD-2009-184	3	2
LUAD-2010-255	3	2
LUAD-2010-260	3	2
LUAD-2010-278	3	2
LUAD-2010-341	3	2
LUAD-2011-344	3	2
LUAD-2011-355	3	2
LUAD-2011-402	3	2
LUAD-2011-417	3	2
LUAD-2011-419	3	2
LUAD-2011-430	3	2
LUAD-2011-457	3	2
LUAD-2007-019	3	2
LUAD-2008-087	3	2
LUAD-2008-112	3	2
LUAD-2009-187	3	2
LUAD-2009-191	3	2
LUAD-2011-370	3	2
LUAD-2011-392	3	2
LUAD-2011-436	3	2
LUAD-2007-002	2	2
LUAD-2008-066	2	2
LUAD-2008-117	2	2
LUAD-2009-175	2	2
LUAD-2010-304	2	2
LUAD-2010-311	2	2
LUAD-2010-324	2	2
LUAD-2011-363	2	2
LUAD-2011-371	2	2
LUAD-2011-426	2	2
LUAD-2011-453	2	2
LUAD-2011-458	2	2
LUAD-2007-005	2	2
LUAD-2007-022	2	2
LUAD-2007-026	2	2
LUAD-2007-028	2	2
LUAD-2007-029	2	2
LUAD-2007-042	2	2
LUAD-2007-044	2	2

For peer review Only

LUAD-2007-053	2	2
LUAD-2007-054	2	2
LUAD-2007-056	2	2
LUAD-2007-057	2	2
LUAD-2007-059	2	2
LUAD-2008-064	2	2
LUAD-2008-069	2	2
LUAD-2008-073	2	2
LUAD-2008-074	2	2
LUAD-2008-075	2	2
LUAD-2008-099	2	2
LUAD-2008-106	2	2
LUAD-2008-109	2	2
LUAD-2008-119	2	2
LUAD-2008-123	2	2
LUAD-2008-124	2	2
LUAD-2008-141	2	2
LUAD-2009-144	2	2
LUAD-2009-154	2	2
LUAD-2009-164	2	2
LUAD-2009-173	2	2
LUAD-2009-174	2	2
LUAD-2009-213	2	2
LUAD-2009-219	2	2
LUAD-2010-244	2	2
LUAD-2010-246	2	2
LUAD-2010-269	2	2
LUAD-2010-271	2	2
LUAD-2010-298	2	2
LUAD-2010-336	2	2
LUAD-2011-356	2	2
LUAD-2011-362	2	2
LUAD-2011-378	2	2
LUAD-2011-404	2	2
LUAD-2011-410	2	2
LUAD-2011-414	2	2
LUAD-2011-423	2	2
LUAD-2011-427	2	2
LUAD-2011-440	2	2
LUAD-2011-441	2	2
LUAD-2011-443	2	2
LUAD-2007-014	2	2
LUAD-2007-015	2	2
LUAD-2007-036	2	2
LUAD-2007-045	2	2
LUAD-2007-055	2	2
LUAD-2008-081	2	2
LUAD-2008-113	2	2
LUAD-2009-149	2	2

Garrido-Martin EM, et al. J Immunother Cancer 2020; 8:e000778. doi: 10.1136/jitc-2020-000778

LUAD-2009-167	2	2
LUAD-2009-198	2	2
LUAD-2009-208	2	2
LUAD-2009-226	2	2
LUAD-2009-230	2	2
LUAD-2010-242	2	2
LUAD-2010-252	2	2
LUAD-2010-270	2	2
LUAD-2010-290	2	2
LUAD-2010-292	2	2
LUAD-2010-302	2	2
LUAD-2010-305	2	2
LUAD-2010-333	2	2
LUAD-2011-350	2	2
LUAD-2011-351	2	2
LUAD-2011-357	2	2
LUAD-2011-361	2	2
LUAD-2011-373	2	2
LUAD-2011-398	2	2
LUAD-2011-421	2	2
LUAD-2011-433	2	2
LUAD-2007-008	2	2
LUAD-2008-128	2	2
LUAD-2009-153	2	2
LUAD-2009-171	2	2
LUAD-2009-203	2	2
LUAD-2010-253	2	2
LUAD-2011-439	2	2
LUAD-2011-460	2	2
LUAD-2007-007	1	2
LUAD-2007-011	1	2
LUAD-2007-016	1	2
LUAD-2007-027	1	2
LUAD-2007-034	1	2
LUAD-2007-038	1	2
LUAD-2007-039	1	2
LUAD-2007-046	1	2
LUAD-2007-047	1	2
LUAD-2008-065	1	2
LUAD-2008-079	1	2
LUAD-2008-083	1	2
LUAD-2008-084	1	2
LUAD-2008-094	1	2
LUAD-2008-097	1	2
LUAD-2008-105	1	2
LUAD-2008-133	1	2
LUAD-2008-136	1	2
LUAD-2009-169	1	2
LUAD-2009-189	1	2

Garrido-Martin EM, *et al. J Immunother Cancer* 2020; 8:e000778. doi: 10.1136/jitc-2020-000778

LUAD-2009-194	1	2
LUAD-2009-218	1	2
LUAD-2009-227	1	2
LUAD-2010-247	1	2
LUAD-2010-280	1	2
LUAD-2010-288	1	2
LUAD-2010-294	1	2
LUAD-2010-307	1	2
LUAD-2010-308	1	2
LUAD-2010-331	1	2
LUAD-2011-345	1	2
LUAD-2011-376	1	2
LUAD-2011-377	1	2
LUAD-2011-383	1	2
LUAD-2011-408	1	2
LUAD-2011-420	1	2
LUAD-2011-451	1	2
LUAD-2007-003	1	2
LUAD-2007-004	1	2
LUAD-2007-006	1	2
LUAD-2007-009	1	2
LUAD-2007-012	1	2
LUAD-2007-024	1	2
LUAD-2007-025	1	2
LUAD-2007-035	1	2
LUAD-2007-037	1	2
LUAD-2007-040	1	2
LUAD-2007-048	1	2
LUAD-2007-049	1	2
LUAD-2007-058	1	2
LUAD-2008-061	1	2
LUAD-2008-063	1	2
LUAD-2008-070	1	2
LUAD-2008-072	1	2
LUAD-2008-077	1	2
LUAD-2008-082	1	2
LUAD-2008-085	1	2
LUAD-2008-088	1	2
LUAD-2008-090	1	2
LUAD-2008-091	1	2
LUAD-2008-092	1	2
LUAD-2008-093	1	2
LUAD-2008-095	1	2
LUAD-2008-096	1	2
LUAD-2008-098	1	2
LUAD-2008-101	1	2
LUAD-2008-103	1	2
LUAD-2008-104	1	2
LUAD-2008-107	1	2

For Peer Review Only

LUAD-2008-111	1	2
LUAD-2008-114	1	2
LUAD-2008-120	1	2
LUAD-2008-121	1	2
LUAD-2008-130	1	2
LUAD-2008-132	1	2
LUAD-2008-135	1	2
LUAD-2008-140	1	2
LUAD-2009-145	1	2
LUAD-2009-147	1	2
LUAD-2009-156	1	2
LUAD-2009-157	1	2
LUAD-2009-162	1	2
LUAD-2009-163	1	2
LUAD-2009-166	1	2
LUAD-2009-178	1	2
LUAD-2009-179	1	2
LUAD-2009-180	1	2
LUAD-2009-181	1	2
LUAD-2009-183	1	2
LUAD-2009-195	1	2
LUAD-2009-196	1	2
LUAD-2009-199	1	2
LUAD-2009-200	1	2
LUAD-2009-202	1	2
LUAD-2009-204	1	2
LUAD-2009-217	1	2
LUAD-2009-225	1	2
LUAD-2009-229	1	2
LUAD-2009-231	1	2
LUAD-2009-233	1	2
LUAD-2009-234	1	2
LUAD-2009-235	1	2
LUAD-2010-239	1	2
LUAD-2010-240	1	2
LUAD-2010-248	1	2
LUAD-2010-256	1	2
LUAD-2010-257	1	2
LUAD-2010-262	1	2
LUAD-2010-263	1	2
LUAD-2010-267	1	2
LUAD-2010-272	1	2
LUAD-2010-276	1	2
LUAD-2010-277	1	2
LUAD-2010-281	1	2
LUAD-2010-283	1	2
LUAD-2010-284	1	2
LUAD-2010-285	1	2
LUAD-2010-286	1	2

For peer review only

LUAD-2010-291	1	2
LUAD-2010-293	1	2
LUAD-2010-300	1	2
LUAD-2010-306	1	2
LUAD-2010-310	1	2
LUAD-2010-316	1	2
LUAD-2010-317	1	2
LUAD-2010-318	1	2
LUAD-2010-321	1	2
LUAD-2010-325	1	2
LUAD-2010-330	1	2
LUAD-2010-332	1	2
LUAD-2010-335	1	2
LUAD-2010-338	1	2
LUAD-2011-348	1	2
LUAD-2011-349	1	2
LUAD-2011-352	1	2
LUAD-2011-353	1	2
LUAD-2011-354	1	2
LUAD-2011-358	1	2
LUAD-2011-374	1	2
LUAD-2011-375	1	2
LUAD-2011-385	1	2
LUAD-2011-387	1	2
LUAD-2011-388	1	2
LUAD-2011-399	1	2
LUAD-2011-401	1	2
LUAD-2011-409	1	2
LUAD-2011-413	1	2
LUAD-2011-415	1	2
LUAD-2011-428	1	2
LUAD-2011-429	1	2
LUAD-2011-434	1	2
LUAD-2011-435	1	2
LUAD-2011-446	1	2
LUAD-2011-447	1	2
LUAD-2008-118	3	3
LUAD-2008-137	3	3
LUAD-2009-159	3	3
LUAD-2009-206	3	3
LUAD-2009-221	3	3
LUAD-2010-265	3	3
LUAD-2010-273	3	3
LUAD-2010-287	3	3
LUAD-2010-315	3	3
LUAD-2011-367	3	3
LUAD-2011-424	3	3
LUAD-2011-431	3	3
LUAD-2011-448	3	3

For peer review only

LUAD-2011-454	3	3
LUAD-2010-289	3	3
LUAD-2010-320	3	3
LUAD-2010-323	3	3
LUAD-2011-395	3	3
LUAD-2007-023	3	3
LUAD-2010-296	2	3
LUAD-2010-334	2	3
LUAD-2011-400	2	3
LUAD-2009-146	2	3
LUAD-2010-279	2	3
LUAD-2011-346	2	3
LUAD-2011-391	2	3
LUAD-2011-406	2	3
LUAD-2011-442	2	3
LUAD-2008-110	2	3
LUAD-2008-129	2	3
LUAD-2008-134	2	3
LUAD-2009-209	2	3
LUAD-2010-251	2	3
LUAD-2010-303	2	3
LUAD-2010-314	2	3
LUAD-2010-327	2	3
LUAD-2011-386	2	3
LUAD-2011-390	2	3
LUAD-2009-211	2	3
LUAD-2011-407	2	3
LUAD-2007-050	1	3
LUAD-2008-108	1	3
LUAD-2009-188	1	3
LUAD-2010-241	1	3
LUAD-2010-264	1	3
LUAD-2010-299	1	3
LUAD-2010-313	1	3
LUAD-2010-322	1	3
LUAD-2007-033	1	3
LUAD-2008-068	1	3
LUAD-2008-086	1	3
LUAD-2008-122	1	3
LUAD-2009-150	1	3
LUAD-2009-186	1	3
LUAD-2009-190	1	3
LUAD-2009-220	1	3

For peer review only

BMJ Publishing Group Limited (BMJ) disclaims all liability and responsibility arising from any reliance placed on this supplemental material which has been supplied by the author(s)

cold tumours

Confidential: For Review Only

Garrido-Martin EM, *et al. J Immunother Cancer* 2020; 8:e000778. doi: 10.1136/jitc-2020-000778

Univariate a

	Factor	N(%)	Mean OS (Months)	95% CI	P value
CD103	Low	72 (57)	1861 (61.1)	1658 to 2063	0.230
	Moderate	39 (31)	2017 (66.3)	1770 to 2263	
	High	15 (12)	2213 (72.8)	1876 to 2549	
	Overall	126	1974 (64.9)	1829 to 2118	
CD103	Low	72 (83)	1861 (61.1)	1658 to 2063	0.109
	High	15 (17)	2213 (72.8)	1876 to 2549	
	Overall	87	1943 (63.9)	1766 to 2119	
CD8	Low	60 (48)	1807 (59.4)	1581 to 2034	0.192
	Moderate	46 (36)	2076 (68.3)	1857 to 2295	
	High	20 (16)	2028 (66.7)	1692 to 2365	
	Overall	126	1942 (63.8)	1766 to 2119	
CD8	Low	60 (75)	1807 (59.4)	1581 to 2034	0.293
	High	20 (25)	2028 (66.7)	1692 to 2365	
	Overall	80	1878 (61.7)	1688 to 2068	
CXCL9	Low	32 (25)	1600 (52.6)	1281 to 1918	**0.028**
	Moderate	83 (65)	1981 (65.1)	1803 to 2158	
	High	13 (10)	2040 (67.1)	1738 to 2342	
	Overall	128	1961 (64.4)	1817 to 2105	
CXCL9	Low	32 (71)	1600 (52.6)	1281 to 1918	**0.01**
	High	13 (29)	2040 (67.1)	1738 to 2342	
	Overall	45	1848 (60.8)	1603 to 2093	
	Male	60 (47)	1883	1662 to 2103	0.377

Confidential: For Review Only

Gender	Male	60 (47)	[illegible] (61.9)	1662 to 2103	0.377
	Female	68 (53)	2006 (65.9)	1817 to 2193	
	Overall	128			
Age	<65	57 (45)	1922 (63.2)	1708 to 2136	0.783
	>65	71 (55)	1975 (64.9)	1783 to 2167	
	Overall	128	1961 (64.5)	1817 to 2105	
Staging		119	1975 (64.9)	1828 to 2124	**8.9x10-8**

Confidential: For Review Only

Garrido-Martin EM, *et al. J Immunother Cancer* 2020; 8:e000778. doi: 10.1136/jitc-2020-000778

analysis using cancer specific death

Confidential: For Review Only

BMJ Publishing Group Limited (BMJ) disclaims all liability and responsibility arising from any reliance placed on this supplemental material which has been supplied by the author(s)

Variable	HR	95% CI	p-value
CD103	0.863	0.643 to 1.171	0.352
CD8	1,023	0.749 to 1.399	0.885
CXCL9	0.713	0.512 to 0.994	**0.046**
Age	0.950	0.665 to 1.358	0.780
Gender	0.919	0.654 to 1.314	0.643

Multivariate analysis using cancer specific death

(Without staging: N=124)

Confidential: For Review Only

Garrido-Martin EM, *et al. J Immunother Cancer* 2020; 8:e000778. doi: 10.1136/jitc-2020-000778
