# Supplementary figures and images for: M1hot tumor-associated macrophages boost tissue-resident memory T cells infiltration and survival in human lung cancer
Source: J Immunother Cancer. 2020 Jul 21;8(2):e000778. doi: 10.1136/jitc-2020-000778 (PMC7375465; doi:10.1136/jitc-2020-000778)

**Figure S5.**

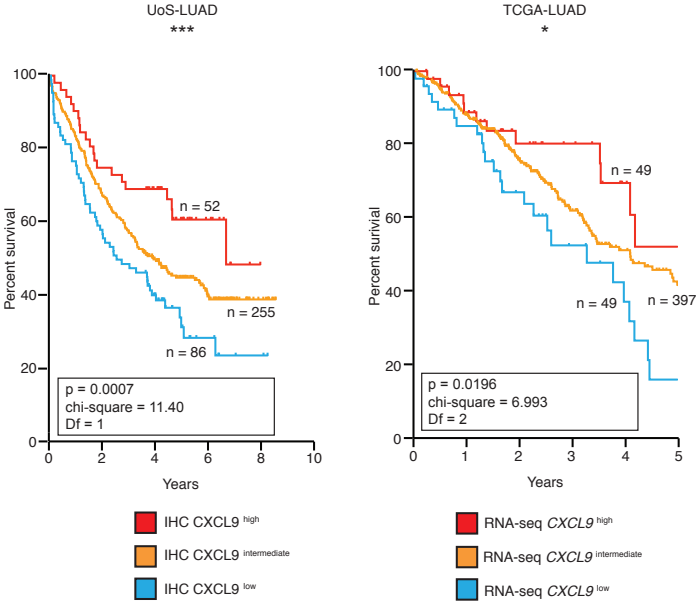

Supplement: Supplementary data [file jitc-2020-000778supp004.pdf]
